# Optimal Synthetic Glycosylation of a Therapeutic Antibody

**DOI:** 10.1002/ange.201508723

**Published:** 2016-01-12

**Authors:** Thomas B. Parsons, Weston B. Struwe, Joseph Gault, Keisuke Yamamoto, Thomas A. Taylor, Ritu Raj, Kim Wals, Shabaz Mohammed, Carol V. Robinson, Justin L. P. Benesch, Benjamin G. Davis

**Affiliations:** ^1^ Department of ChemistryUniversity of OxfordChemistry Research LaboratoryMansfield RoadOxfordOX1 3TAUK; ^2^ Department of PharmacologyUniversity of OxfordOxfordOX1 3QTUK

**Keywords:** Antikörper, Endoglykosidasen, Glyko-Engineering, Glykosylierungen, Native Massenspektrometrie

## Abstract

Glycosylation patterns in antibodies critically determine biological and physical properties but their precise control is a significant challenge in biology and biotechnology. We describe herein the optimization of an endoglycosidase‐catalyzed glycosylation of the best‐selling biotherapeutic Herceptin, an anti‐HER2 antibody. Precise MS analysis of the intact four‐chain Ab heteromultimer reveals nonspecific, non‐enzymatic reactions (glycation), which are not detected under standard denaturing conditions. This competing reaction, which has hitherto been underestimated as a source of side products, can now be minimized. Optimization allowed access to the purest natural form of Herceptin to date (≥90 %). Moreover, through the use of a small library of sugars containing non‐natural functional groups, Ab variants containing defined numbers of selectively addressable chemical tags (reaction handles at Sia C1) in specific positions (for attachment of cargo molecules or “glycorandomization”) were readily generated.

## Graphical abstract

**Der Blockbuster‐Antikörper** Herceptin ist durch natürliche Glykosylierung mittels chemoenzymatischem Aufbau gekoppelt mit Massenspektrometrie des intakten Antikörpers zugänglich (siehe Bild). Herceptin wurde in hoher Reinheit (>90 %) erhalten, wenn nichtspezifische, nichtenzymatische Reaktionen (Glykierung) zurückgedrängt wurden. Glykosylierung mit nichtnatürlichen Zuckern ermöglichte ferner das regioselektive Anbringen einer Fracht.

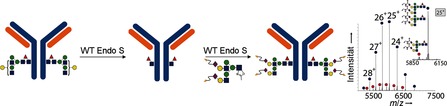

Protein glycosylation is the most common and varied post‐translational modification, critically influencing protein function,^1^ and yet is currently difficult to control.^2^ Endoglycosidase (ENGase or “Endo”)‐catalyzed glycosylation is an attractive strategy to access homogeneous glycoproteins (Figure 1 a);^3^ the initially limited scope of glycoproteins was expanded to antibodies (Abs) by the use of EndoS,^4^ a family 18 glycoside hydrolase (GH) from *Streptococcus pyogenes* capable of glycosylating immunoglobulins (Igs).^5^ Monoclonal Abs (mAbs) and antibody–drug conjugates (ADCs) are a rapidly growing class of therapeutics.^6^ Glycans in Abs^7^ modulate stability, the rate of clearance, and the pharmacokinetic profile;^8^ aggregation, folding, and immunogenicity;^9^ complement activation;^10^ binding to Fc receptors and Ab‐dependent cell‐mediated cytotoxicity;^11^ and Ab‐mediated inflammation.^12^ They are therefore vital functional “switches” that cannot yet be controlled cleanly (see the Supporting Information for an extended discussion).

**Figure 1:**
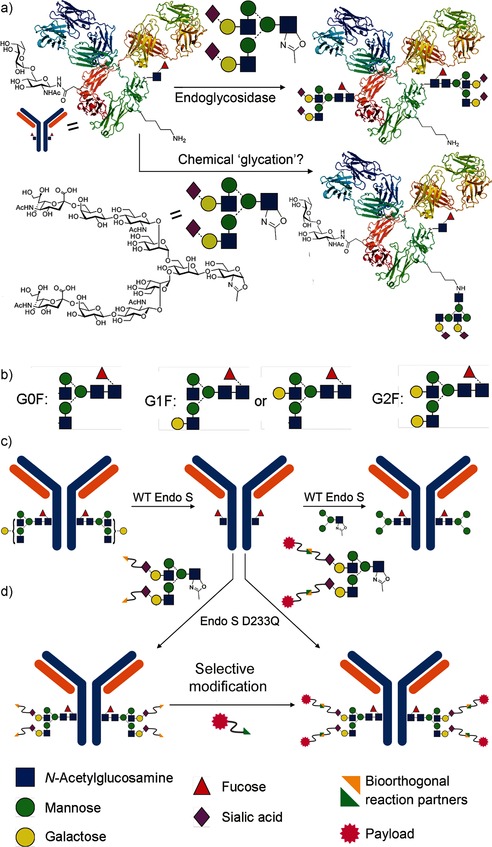
a) Endoglycosidase‐catalyzed glycosylation with activated sugar donors may lead to competing chemical glycation. b) Current mAbs are formed as mixtures of glycoforms; G0F, G1F, and G2F predominate. c) EndoS‐WT cleaves the core of mixed N‐glycans and can subsequently catalyze glycosylation with oxazoline donors,5a to give, in principle, purer glycoform distributions. d) Payload molecules may also, in principle, be introduced directly through glycosylation or indirectly by the incorporation of reactive handles in sugars and a subsequent selective reaction.

Antibodies are all N‐glycosylated in the Fc region of each of two heavy chains. All therapeutic Abs are currently produced from cells as mixtures (Figure 1 b); more than 20 different glycoforms are typically identified.^13^ By contrast, the chemoenzymatic ENGase method could potentially be used to access pure Abs. However, until now it has been assumed that this method will necessarily give rise to homogeneous glycoforms by virtue of the direct reversal of selective enzymatic hydrolytic activity (Figure 1). Herein we demonstrate that this assumption is incorrect: not only do nonspecific background chemical modifications compete, but we now reveal optimized methods that allow access to essentially homogenous (≥90 % pure) glycoforms of a key therapeutic mAb.

Our preliminary studies5a had indicated that wild‐type (WT) EndoS could be successfully used to trim glycans from mixtures of glycoforms of human IgG to reveal single GlcNAc moieties (Figure 1 c, left). Subsequent treatment of the resulting IgG‐GlcNAc with WT EndoS and an appropriately activated sugar oxazoline donor led to the formation of a new glycosidic linkage (Figure 1 c, right).5a However, the inherent hydrolytic activity of EndoS prevented fully efficient reactions. To overcome this limitation, we explored the use of mutated variants of EndoS to access enzymes with enhanced transglycosylation:hydrolysis (T:H) activity ratios. Similar strategies^14^, ^15^ have proven successful in other ENGase systems, by partial analogy with synthases described by Withers and co‐workers.^16^, ^17^ Sequence alignment (see the Supporting Information) with other family 18 and 85 GHs^18^ suggested residues D233, E235, Q303, and Y305, which enhance the role of the C2 amide in reactions involving oxazolinium intermediates (D233), act as a general acid/base (E235), or assist substrate binding (Q303, Y305).^19^ We generated EndoS mutants and assessed combined T:H activities (100:1 [Ab]:[EndoS]; T:H=35:30 (D233A), 65:25 (D233A/Q303E), nd:100 (Y305F), 75:55 (D233E), 80:20 (D233Q), 10:100 (WT); nd=not determined; see the Supporting Information). Although, in our hands, none displayed completely abolished hydrolytic activity, it was substantially decreased in EndoS‐D233Q as compared to EndoS‐WT, thus giving rise to a T:H activity of 80:20. We therefore selected EndoS‐D233Q. During the course of this study, Wang and co‐workers also suggested that EndoS‐D233Q and EndoS‐D233A mutants possess useful “synthase” activity.5b The mutant EndoS‐D233Q is sufficiently stable to be produced on scale.

We chose the therapeutic mAb Herceptin as a highly representative substrate (see the Supporting Information). Our analysis of Herceptin (see Figure S4 in the Supporting Information) suggested at least seven major glycoforms with many other minor species, dominated by complex biantennary structures, consistent with prior observations.^20^ We estimate the most prevalent (asymmetric G0F/G1F) to account for less than 35 %; Herceptin is therefore highly heterogeneous.

We set out to create a pure, single, symmetric glycoform of Herceptin bearing a relevant complex biantennary glycan at each Fc Asn300 position.^21^ A corresponding activated sugar oxazoline **2** was created on a tens‐of‐milligrams scale^22^ to enable the creation of a fully sialylated G2F/G2F (S2G2F/S2G2F) glycoform (S2G2F/S2G2F‐Herceptin). In principle, this glycan would convey designed anti‐inflammatory properties,12a but at levels of incorporation not accessible in previous studies. Its incorporation would, in turn, enable the ready creation, advantageously, of ADCs with reduced inflammatory properties in a manner not previously possible.

First, Herceptin was converted cleanly into Herceptin‐GlcNAc (**1**) by the use of EndoS‐WT. Then, glycosylation at 30 °C in phosphate buffer (pH 6.5) with oxazoline donor **2** (2×70 equivalents, second addition after 40 min) in the presence of EndoS‐D233Q gave the desired glycosylated Ab **3**, essentially as a single glycoform (Figure 2 a,b), as judged by SDS‐PAGE and reducing LC–MS (rMS, after the reduction of inter‐ and intrachain disulfides to give two heavy and two light chains). This method of analysis has been the dominant and most successful approach to assess the chemistry of monomeric proteins by us and others.^23^ However, the mAb products of these reactions are heteromultimeric, and it occurred to us that the true details of these reactions (at, for example, each of the two Asn300 sites simultaneously) would only be revealed in this relevant protein complex. We elucidated conditions for evaluation by high‐resolution native MS (nMS; see the Supporting Information). Strikingly, such nMS analysis without reduction revealed heterogeneity not detected in the monomeric state by rMS. Although the major reaction product was the expected diglycosylated Ab **3**, we also identified peaks corresponding to the attachment of only one and even three glycans (Figure 2 c,d), along with small quantities of glycoforms containing only one core fucosyl moiety (see the Supporting Information). The estimated purity of **3** in this product mixture was below 75 %.^24^

**Figure 2:**
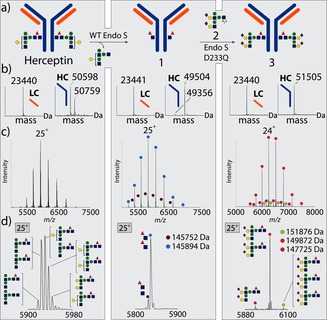
a) EndoS‐WT‐catalyzed deglycosylation of Herceptin gives trimmed Herceptin **1**. Subsequent glycosylation with the sugar oxazoline **2** gives remodeled Ab **3**. b) Deconvoluted rMS data for samples of reduced commercial, trimmed, and remodeled Herceptin (see Figures S8–S10). c) Native MS for intact commercial, trimmed, and remodeled Herceptin. d) Single‐charge‐state peaks. Colored circles indicate major species present. A minor, non‐glycan +176 Da component (EndoS‐ and PNGaseF‐resistant) was present in all Herceptin (including commercial) samples analyzed.

The detection of unexpected Ab products bearing three glycans had a striking implication: nonselective chemical reaction(s) occur(s) alongside the desired selective enzymatic reaction. Treatment of the glycosylation products with PNGaseF, which removes N‐linked glycans from Herceptin, left these additional glycans attached (see Figures S25–S27). Tryptic MS/MS mapping confirmed their non‐Asn‐linked nature (see the Supporting Information). Notably, Wang and co‐workers have suggested that other endoglycosidases (i.e., EndoA) have broad acceptor‐substrate specificity, thus potentially glycosylating acceptors other than Asn‐linked GlcNAc.^25^ Finally, we tested the direct reaction of **2** with Herceptin‐GlcNAc (**1**; Figure 3; see also Figures S15 and S16); after 2 h in the absence of an enzyme, chemical addition products (“glycation”) were observed. At pH 7.4, around 20 % of the mAb carried one glycan, 53 % carried two glycans, and 27 % carried three glycans (Figure 3 a).5c, 26, 27 When the pH value was lowered to 6.5, around 69 % of the mAb had not undergone any glycation after 2 h: 26 % carried one glycan, and 5 % carried two glycans. Direct high‐resolution rMS of glycated samples indicated that most glycation occurred on the heavy chain, with small amounts on the light chain (Figure 3 b). LC–MS/MS following tryptic digestion revealed several possible sites (including heavy‐chain site K30 and light‐chain sites K188, H189, and K190; see Figure S41). Importantly, the direct treatment of Herceptin with lactol **4** did not result in glycation, thus indicating that an excess of a reducing sugar (e.g., from the hydrolysis of **2** to **4**) was not the cause of the “glycation” process (see Figures S17 and S18).

**Figure 3:**
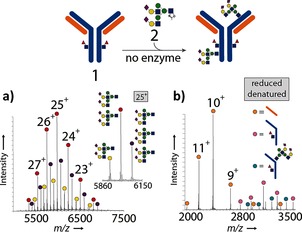
Treatment of **1** with donor **2** at pH 7.4 in the absence of an enzyme leads to substantial glycation. a) nMS of an intact glycated antibody. The inset is an expansion of the +25 charge state. b) rMS showing that the majority of glycation occurs on the heavy chain.

Having discovered this previously unreported, but critical, aspect of the ENGase method, we set out to devise newly optimized methods to solve this apparent problem. Any solution would need to address three issues simultaneously: the optimization of transglycosylation (T) and minimization of two competing reactions, hydrolysis (H) and “glycation” (G). Through experimentation, we logically optimized the T:H+G ratio with EndoS‐D233Q; we thus used a higher enzyme loading (10 % w/w rather than 2–5 %) and lower peak concentrations of oxazoline **2** ([Ab]=13.3 μm; [**2**]_init_<0.2 mm, multiple additions (7×) of fewer (15) equivalents at 15 min intervals). nMS revealed an efficient reaction with virtually no discernible “glycation” and at least 90 % desired bisglycosylation (fully sialylated mAb/mAb bearing four sialic acid residues, giving >75 %^28^ **3**, S2G2F/S2G2F‐Herceptin; Figure 4).

**Figure 4:**
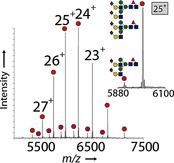
Native MS of glycosylation products after optimization of the reaction conditions with the native decasaccharide donor **2**. The inset shows an expansion of the +25 charge‐state peaks.

Herceptin is also known to internalize after binding to the HER2/neu receptor,^29^ thereby valuably raising the potential of this mAb as a platform for intracellular drug/toxin/diagnostic targeting; this potential has been recognized in the development of the ADC Kadcyla. We envisaged that judiciously selected sugar donors would therefore not only enable efficient access to Ab constructs with defined natural glycosylation but also modified, unnatural glycans bearing precise numbers of attachment sites (handles or tags) at set positions. These sites would enable the subsequent attachment of “cargo”. Alternatively, such “cargo” could be attached to the glycan prior to enzymatic glycosylation (Figure 1 d). Various appropriate representative (potentially selectively addressable) “handles” were incorporated into **4** to create activated sugar donors **6 a**–**e** (via **5 a**–**e**) as test substrates for EndoS (Table 1). Our chemoselective synthetic strategy avoided the use of protecting groups and allowed direct conversion from free reducing sugars. Thus, the two C2 carboxylate groups found in the nonreducing terminal sialic acids of the decasaccharide lactol **4** 22c were converted into amides with 4‐(4,6‐dimethoxy‐1,3,5‐triazin‐2‐yl)‐4‐methylmorpholinium chloride (DMTMM).^30^ Through the use of an elevated temperature (37–50 °C) and an excess of the ammonium salt and coupling agent we were able to drive amidation to completion to give **5 a**–**e**. In each case, the product was readily purified by filtration and size exclusion chromatography. Subsequent dehydration of lactols **5 a**–**e** to the oxazolines **6 a**–**e** was carried out with 2‐chloro‐1,3‐dimethyl‐1*H*‐benzimidazol‐3‐ium chloride (CDMBI) under mildly basic conditions.22c Given the ready access to **4**,22a,22b this route provides a rapid method to attach reaction handles to biologically important glycans (and in turn to proteins, see below) with minimal operational manipulation and/or purification, thereby allowing its ready application and scale‐up. It could also, in principle, be applied to any neuraminyl‐containing sugar extracted from a natural source, even mixed samples.

**Table 1:** Functionalization of the carboxylate moiety of **4** and subsequent oxazoline formation.

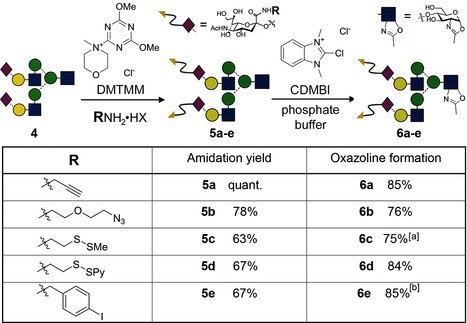

[a] The disulfide linkage was reduced by the addition of dithiothreitol immediately following oxazoline formation. [b] The product was composed of around 70–80 % of the target **6 e** and 20–30 % unidentified material, which had undergone further dehydration.

Pleasingly, in every case EndoS‐D233Q catalyzed the glycosylation of Herceptin‐GlcNAc (**1**) with the functionalized, non‐natural glycans **6 a**–**e** to give glycosylated mAbs **7 a**–**e** (see Table S1) carrying two glycans each functionalized with two reaction handles as the major products, as judged by rMS and reducing SDS‐PAGE (see Figures S20–S24). However, as before, nMS analysis of these Abs under native conditions indicated varying levels of purity. Significant amounts of Ab carrying one or three glycans were observed (heterogeneity was dependent upon the sugar used; see Table S1). Larger and more “unnatural” tags led to a decrease in efficiency (from ≥90 % desired bisglycosylation with “natural” **2** to 74 % bis‐ and 22 % monoglycosylation with propargyl **6 a**, 65 % bis‐ and 27 % monoglycosylation with azide **6 b**, 70 % bisglycosylation with thiol **6 c**, and only 59 % bisglycosylation with **6 e**, which contains a bulkier iodoaryl group; see Figures S20–S24). The presence of a labile disulfide in **6 d** apparently contributed to the high heterogeneity observed in its Ab product (<40 % of **7 d**).

We chose to optimize the transglycosylation reaction by using the non‐natural oxazoline donor **6 b** bearing an azide group to create **7 b** (Figure 5). Use of the previously optimized conditions (7×15 equiv) led to only small amounts of glycation (ca. 2 %). Glycation was almost entirely eliminated by further decreasing the rate of addition (20×5 equiv, 5 min intervals); under these conditions, 90 % bisglycosylation was observed (Figure 5 b). Notably, circular dichroism analysis (Figure 5 a) showed that the gross secondary structural elements in Her were preserved during this optimized glycosylation to give azido‐Herceptin Ab **7 b**.

**Figure 5:**
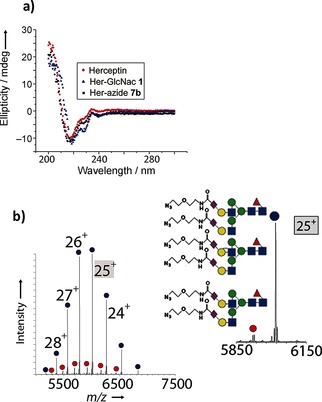
a) Circular dichroism analysis shows no gross structural changes during remodeling with unnatural sugar **6 b** to form **7 b**. b) Native MS of the glycosylation product **7 b** after optimization of the reaction conditions with the azide‐tagged decasaccharide donor **6 b**. The inset shows an expansion of +25 charge‐state peaks.

The azide “tag” sites in the glycans in azido‐Herceptin Ab **7 b** were then used for the attachment of cargo by using a rhodamine alkyne and a cemadotin alkyne variant (generated by the use of standard solid‐phase peptide synthesis techniques; see the Supporting Information). These compounds allowed us to demonstrate the functional integrity of a synthetic Her with optimal glycosylation bearing cargo. Rhodamine–Herceptin generated in this way bound metastatic breast adenocarcinoma HER2(+) cells (SK‐BR‐3) selectively (no binding to HER2(−) MCF‐7 cells), as shown by fluorescence‐activated cell sorting (Figure 6). We were also able to generate Herceptin “ADC” variants with different loadings of the cemadotin toxin (ca. 2, 3, or 4; see the Supporting Information). Herceptin bearing approximately three cemadotin units showed enhanced killing of HER2(+) cells (SK‐BR‐3) over Her (EC_50_≈800 pm; see the Supporting Information).

**Figure 6:**
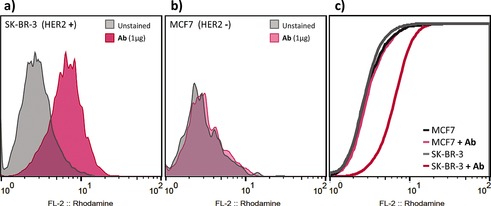
Cell‐surface staining of HER2 with **7 b** modified with rhodamine cargo. Flow cytometry histograms of a) SK‐BR‐3 cells (HER2(+)) and b) MCF‐7 cells (HER2(−)), unstained (gray) or stained with the synthetic Ab (pink). c) A cumulative distribution function plot of the summed fluorescence intensity shows a 2.3‐fold change in geometric mean intensity for SK‐BR‐3.

We have shown herein the first examples, to the best of our knowledge, of the use of nMS to direct the optimization of protein chemistry. This approach has vitally enabled the precise and detailed remodeling of Abs, by revealing reactions that were not previously appreciated. Although rMS is an essential and useful tool for the characterization^23^ and monitoring of chemical modification,^31^ labile heteromultimers, such as the Ab Herceptin used in this study, may require such precision. The ionization of native protein complexes is dependent on the surface composition, which is largely invariant between, for example, WT, cleaved, and remodeled IgG Herceptin. The relative glycoform intensities detected and assigned by native MS can therefore be taken as proxy for the relative abundances, in line with the well‐known minor effects of post‐translational modification on intact‐protein analysis.^32^

Our increased control of the natural glycosylation pattern of Abs may allow the construction of new optimally efficacious Abs. In this study, highly pure sialylated Ab glycoforms have been created; Fc glycan sialylation imparts anti‐inflammatory properties to IgGs,12a and has led to the use of IVIg as an anti‐inflammatory drug. However, only approximately 10 % of the total IgG content of IVIg carries sialylation.^33^ The production of pure sialylated Abs (>90 % sialylated, as in this study) should improve anti‐inflammatory properties and reduce dosage. Interestingly, aberrant Ab glycation has also been linked to disease (see the Supporting Information).^34^

The incorporation of unnatural glycans into Abs also allowed dual simultaneous access to defined glycosylation and specifically positioned reaction handles. Current approaches to ADCs in the clinic use backbone Lys and Cys residues and give rise to heterogeneity. Recently, other complementary approaches have been suggested that are based on the use of unnatural amino acids^35^ or Fc N‐glycosylation.^36^ Some require either complete removal36a or significant truncation5b, 36b of the glycans, which may adversely affect Ab stability and immunogenicity^8^, ^9^ as well as impacting on FcγR binding.^37^ Alternatively, remodeling at the nonreducing termini36c,36d (“tip sugars”), although in principle capable of partially reducing heterogeneity, is limited to certain residues (i.e. Gal); it also does not remove the variation arising from bisecting sugars^38^ and/or hybrid/triantennary/other branching glycans.^39^ The use of unnatural AAs does not avoid the desire for or solve the problem of defined glycosylation. The approach reported herein now enables the attachment of a defined number of reaction handles at specified positions, while removing virtually all glycan heterogeneity. In this way, it allows access to a single key glycan motif with the potential for both the attachment of cargo and reduction of inflammation. Thus, ADCs can be created with improved anti‐inflammatory properties by a general optimized strategy. We note too that the in vivo attachment of cargo may have distinct advantages.^40^ Other “pure and tagged” glycoproteins (created by the use of **6 a**–**e**, for example), besides Abs, are anticipated to be of general utility as therapeutics, for diagnostic purposes, and as probes of organismal biology.

## Supporting information

As a service to our authors and readers, this journal provides supporting information supplied by the authors. Such materials are peer reviewed and may be re‐organized for online delivery, but are not copy‐edited or typeset. Technical support issues arising from supporting information (other than missing files) should be addressed to the authors.

Supplementary
