# Supplementary material for: Optimal Synthetic Glycosylation of a Therapeutic Antibody
Source: Angew Chem Weinheim Bergstr Ger. 2016 Jan 12;128(7):2407–13. doi: 10.1002/ange.201508723 (PMC4974915; doi:10.1002/ange.201508723)
Supplement: Supplementary file 1 — Supplementary [file ANGE-128-2407-s001.pdf]

Angewandte Chemie
Eine Zeitschrift der Gesellschaft Deutscher Chemiker

Supporting Information

Optimal Synthetic Glycosylation of a Therapeutic Antibody

Thomas B. Parsons, Weston B. Struwe[+], Joseph Gault[+], Keisuke Yamamoto, Thomas A. Taylor, Ritu Raj, Kim Wals, Shabaz Mohammed, Carol V. Robinson, Justin L. P. Benesch, and Benjamin G. Davis**

ange_201508723_sm_miscellaneous_information.pdf

1.1 Sequence alignment between glycoside hydrolases 1
1.2 Overexpression and purification of EndoS mutants 5
1.3 Hydrolytic activity of EndoS mutants 5
2.1 Materials and general methods: chemical 9
2.2 Chemical Synthesis 9
2.2.1 Prior Methods 9
2.2.2 Synthesis of sugars 10
2.2.3 NMR data 27
2.2.4 Synthesis of Rhodamine Cargo Molecule 8a 40
2.2.5 Synthesis of Cemadotin Cargo Molecule 8b 41
3.1 Antibody remodelling 44
3.1.1 General methods: biological 44
3.1.2 Trimming of Herceptin to produce 1 45
3.1.3 Transglycosylation activity of EndoS mutants 46
3.1.4 LC-MS analysis of commercial, trimmed and remodelled Herceptin 49
3.2 Analysis of antibodies under native/non-denaturing MS conditions 51
3.2.1 Sample preparation 51
3.2.2 Sample analysis 51
3.3 PNGase F treatment of Herceptin 56
3.3.1 Typical procedure for PNGase F digestion of antibodies 56
3.3.2 Deglycosylated herceptin 56
3.4 Glycation control reactions 58
3.4.1 Glycation control reaction using activated sugar donor 2 58
3.4.2 Glycation control reaction using non-activated sugar 4 60
3.5 Optimized glycosylation 63
3.6 Glycosylation using 'tagged' non-natural sugars 65
3.6.1 Typical glycosylation reaction using tagged sugar oxazoline donors 65
3.6.2 Glycosylation using alkyne-tagged glycan 6a 65
3.6.3 Glycosylation using azide-tagged glycan 6b 67
3.6.4 Glycosylation using thiol-tagged glycan 6c 69
3.6.5 Glycosylation using pyridyl disulfide-tagged glycan 6d 71
3.6.6 Glycosylation using iodoaryl-tagged glycan 6e 73
3.7 PNGase F treatment of glycated antibodies 76
3.7.1 Native MS analysis of PNGase-treated glycation products 76
3.7.2 Denaturing MS analysis of PNGase F-treated glycation products 78
3.8 Loading of Cargo Molecules 85
3.8.1 Loading of Rhodamine 85
3.8.1 Loading of Cemadotin 86
4.1 Elucidation of MS technique 90

4.2 Tryptic mapping of Herceptin **92**

4.3 LC-MS/MS analysis **98**

5.1 Extended Introduction **100**

5.2 Extended Conclusions **101**

6.1 References **102**

1.1 Sequence alignment between glycoside hydrolases

Endo M (line 1, Q9C1S6), Endo A (line 2, Q9ZB22), Endo D (line 3, Q93HW0), Endo F1 (line 4, P36911), Endo H (line 5, P04067), Endo F3 (line 6, P36913), Endo S (line 7, Q9APG4) and Endo F2 (line 8, P36912) sequences were aligned to allow identification of conserved residues invovled in catalysis and substrate binding. Residues in the Endo S sequence which were mutated during this work and highlighted in yellow.

```
                      *        20         *        40         *        60         *        80         *       100         *       120
tr|Q9C1S6| : ------------------------------------------------------------------------------------------------------------------------ :    -
tr|Q9ZB22| : ------------------------------------------------------------------------------------------------------------------------ :    -
tr|Q93HW0| : MKNPFF------------------------ERRCRYSIRKLSVGACSLMIGAVLFVGPALAEETAVPENSGANTELVSGESEHSTNEADKQNEGEHTRENKLEKAEGVATASETAEAASA :   96
sp|P36911| : --------------------------MKKFINQFSASLKNNILV---FL-AFPFVWTSCARDNP------------LSSENSN------------------I------------------ :   42
sp|P04067| : -----------------------------MFTPVRRRVRTAALA---LSAAAALVLGSTAAS----------------GASAT------------------P------------------ :   36
sp|P36913| : -------------------------------------MKKIFFAQCSI---LLLMLGSCSKMT----------ED-MTPES--------------------------------------- :   30
tr|Q9APG4| : MDKHLLVKRTLGCVCAATLMGAALATHHDSLNTVKAEEKTVQVQKGLPSIDSLHYLSENSKKEFK--------EE-LSKAGQE------------------S------------------ :   75
sp|P36912| : ---------------------------------MKTAN--FSFALCLSVVI-MLFIK-C------------------TRSEQD------------------L------------------ :   29

                      *       140         *       160         *       180         *       200         *       220         *       240
tr|Q9C1S6| : -----------------------------------------------------MPSLQLQPDDKLAPVSFALKSMNELRDWTPDEK--IKFNVSSVALQPRVKNALK------PQLLLTH :   59
tr|Q9ZB22| : -----------------------------MRKAFLVGLV--------CTACVLLHDDPVAASTYNGPL-SSHWFPEELAQWEPDSDPDAPFNRSHVPLEPGRVANRVNANADKDAHLVSL :   82
tr|Q93HW0| : AKPEEKAGEVVAETPSAEAKPKSDKETEAKPEATNQGDESKPAAEANKTEKEVQPDVPKNTEKTLKPKEIKFNSWEELLKWEPGAREDDAINRGSVVLASRRTGHLVNEKASKEAKVQAL :  216
sp|P36911| : ----SP-----N------------AAARAAVTGT----------------------------TKANIKLFSF-----TEVND-----TNPLNNLNFTLKNSGKPL-------VDMVVLFS :   96
sp|P04067| : ----SP-----A---------------PAPAPAP----------------------------VKQGPTSVAY-----VEVNN-----NSMLNVGKYTLADGGGNA-------FDVAVIFA :   87
sp|P36913| : ----------VN------------KEA-SVKSAT-------------------------ALAGSNGV-CIAY-----YITD----------GRNPTFKLKDIPDK-------VDMVILFG :   79
tr|Q9APG4| : ----QKVKEILA------------KAQQADKQAQ-------------ELAKMKIP-EKIPMKPLHGPLYGGY-----FRTWHDKT--SDPTEKDKVNSMGELPKE-------VDLAFIFH :  151
sp|P36912| : ----S-----VT------------KDAIAQKSGV-------------TVSAVNLS-NLIAYKNSDHQISAGY-----YRTWRDSA--TA---SGNLPSMRWLPDS-------LDMVMVFP :   97
                                                                                                                              d

                      *       260         *       280         *       300         *       320         *       340         *       360
tr|Q9C1S6| : DMAGGYKEDKNIQGNNYKDIYNIQYWHLADTFVYFSHE------RVSIPPVNWTNACHRNGVKCLGTFLVEGNNQMHEMEALLHGPPL-LNNTDDPMRLWSPYYADQLV-AIAKHYGFDG :  171
tr|Q9ZB22| : SALNRHTSGVPSQGAPVFYENTFSYWHYTDLMVYWAGSA--GEGIIVPPSADVIDASHRNGVPILGNVFFPPTVYGGQLEWLEQML---EQEEDG-----SFPLADKLL-EVADYYGFDG :  191
tr|Q93HW0| : SNTNSKAKDHASVGGEEFKAYAFDYWQYLDSMVFWEGL---------VPTPDVIDAGHRNGVPVYGTLFFNWSNSIADQERFAEAL---KQDADG-----SFPIARKLV-DMAKYYGYDG :  318
sp|P36911| : AN----------------INY----DAANDKVFVSNNPN---VQHLLTNRAKYLKPLQDKGIKVILSILGNH----D-RSGIANLS-----TA-R-----AKAFAQELK-NTCDLYNLDG :  176
sp|P04067| : AN----------------INY----DTGTKIAYLHFNEN---VQRVLDNAVTQIRPLQQQGIKVLLSVLGNH----Q-GAGFANFP-----SQQA-----ASAFAKQLS-DAVAKYGLDG :  168
sp|P36913| : L----------------------KYWSLQDTTKLPGGTGMMGSFKSYKDLDTQIRSLQSRGIKVIQNIDDDVSWQSS-K----------PGGFAS-----AAAYGDAIKSIVIDKWKLDG :  161
tr|Q9APG4| : DW----------------TKDYSLFWKELATKH--------------------VPKLNKQGTRVIRTIPWRFLAGGD-NSGIAEDTSKYPNTPEG-----NKALAKAIVDEYVYKYNLDG :  229
sp|P36912| : DY----------------TPPENAYWNTLKTNY--------------------VPYLHKRGTKVIITLGDLNS-----------ATTTGGQDSIG-----YSSWAKGIYDKWVGEYNLDG :  165
                                      w                                  G                                           a  6       5  DG

                      *       380         *       400         *       420         *       440         *       460         *       480
tr|Q9C1S6| : WLFNIECEFFPFPTNPKFKAE--------------ELAKFLHYFKE----KLHNEIPGSQLIWYDSMTN------EGEIHWQNQLTWKNELFFK------NTDGIFLNYWWKKEYPEMAR :  261
tr|Q9ZB22| : WFINQETEGAD----E-GTAE--------------AMQAFLVYLQE----QKP---EGMHIMWYDSMID------TGAIAWQNHLTDRNKMYLQ-NGSTRVADSMFLNFWWRDQRQSNEL :  278
tr|Q93HW0| : YFINQETTGDL----VKPLGE--------------KMRQFMLYSKE----YAAKVNHPIKYSWYDAMTY------NYGRYHQDGLGEYNYQFMQPEGDKVPADNFFANFNWDKAKNDYTI :  410
sp|P36911| : VFFDDEYSAYQTPPPSGFVTPSNNAAARLAYETKQAMPNKLVTVYV----YSRTS---SFPTAVDGVNAGSYVDYAIHDYGGSYDLATNYPGLAKSGMVMSS------------------ :  271
sp|P04067| : VDFDDEYAEYGNN---GTAQPNDSSFVHLVTALRANMPDKIISLYN----IGPAA---S-RLSYGGVDVSDKFDYAWNPYYGTWQV----PGIALPKAQLSP------------------ :  255
sp|P36913| : ISLDIEHSGAKPNPIPTFPGYAATGYNGWYSGSMAATPAFLNVISELTKYFGTTAPNNKQLQIASG-----IDVYAWNKIMENFRNNFNYIQLQSYGANVSRTQ---------------- :  260
tr|Q9APG4| : LDVDVEHDSIPKVDKK---------------EDTAGVERSIQVFEEIGKLIGPKGVDKSRLFIMDS-----TYMADKNPLIERGAPYINLLLVQVYGSQGEKGG---------------- :  313
sp|P36912| : IDIDIESSPSGAT-----------------------LTKFVAATKALSKYFGPKSGTGK-TFVYDT-----NQ--NPTNFFIQTAPRYNYVFLQAYGRSTT------------------- :  235
                1 E                                  6                       d                       n       g
```

```
                      *        500        *        520        *        540        *        560        *        580        *       600
tr|Q9C1S6| : RVAEGIGRSGLEVYFGTDVWGRHTYGGGGFKSYKGV-KTAYSAMTS-SALFGMAWTYEHFEK-SEFEKM-------------DRLFWCGGKYSDYPPPPPKNPDDEKEVESDDSEDELMY :  365
tr|Q9ZB22| : --AQALGRSPYDLYAGVDVEARGT-STPV--QWEGLFPEGEKAHTS-LGLYRPDWAFQSSETMEAFYEK-------------ELQFWVGSTGN-----PAE-----TD------GQSNW- :  362
tr|Q93HW0| : ATANWIGRNPYDVFAGLELQQGGSYKTKV--KWNDILDENGKLRLS-LGLFAPDTITSLGKTGEDYHKN-------------EDIFFTGYQGD-----PTG----QKP------GDKDW- :  498
sp|P36911| : -------------------------------------------------------------------------------------------------------------------QEFNQ :  276
sp|P04067| : -------------------------------------------------------------------------------------------------------------------AAVEI :  260
sp|P36913| : --------------------------------L-----------------------MMNYATGTNKIPASKMVFGAYAEGGT------------------------------------NQ :  289
tr|Q9APG4| : --------------------------------WEPVSNRPEKTMEERWQG------YSKYIRPEQYM----IGFSFYEENAQEGNLWYDINSR-----KDE--DKANG-----INTDITG :  379
sp|P36912| : ------------------------------------------NLTTVSGL------YAPYISMKQFL----PGFSFYEENGYPGNYWNDVRYP-----QNG------------------T :  280

                      *        620        *        640        *        660        *        680        *        700        *       720
tr|Q9C1S6| : GHKKGIADTVESIPVP---GTDWFVTNFDRGFGN--------------RFYYRGKRLLSQPWSHLSHQAILPNKSYRNP--EIYPTDQ-NIKITSSL---DCDHGAFLGGTSLIIKGQRF :  462
tr|Q9ZB22| : ---PGMAHWFPAKSTA---TSVPFFVTHFNTGSGA-------------QFSAEGKTVSEQEWNNRSLQDVLPTWRWIQH----------GGDLEATF---S-WEEAFEGGSSLQWHGSLA :  448
tr|Q93HW0| : ---YGIANLVADRTPA---VGNTFTTSFNTGHGK--------------KWFVDGKVSKDSEWNYRSVSGVLPTWRWWQT--ST------GEKLRAEY---D-FTDAYNGGNSLKFSGDVA :  586
sp|P36911| : GR---YATAQALRNIVTKGYGGHMIFAMDPNRSNFTSGQLPALKLIAKELYGDELVYSNTPYSKDW------------------------------------------------------ :  339
sp|P04067| : GRTSRSTVADLARRTVDEGYGVYLTYNLDGGDRT------ADVSAFTRELYGSEAVRTP------------------------------------------------------------- :  313
sp|P36913| : ANDVEVAKWTPTQ---G-AKGGMMIYTYNSNVSYANAVRDAVKN---------------------------------------------------------------------------- :  329
tr|Q9APG4| : TRAERYARWQPKT---GGVKGGIFSYAIDRDGVAHQPKKYAKQKEFKDA--TDNIFHSDYSVSKALKTVMLKDKSYDLIDEKDFPDKALREAVMAQVGTRKGDLERFNGTLRLDNP--AI :  492
sp|P36912| : GRAYDYARWQPAT---G-KKGGVFSYAIERDAPLTSSNDNTL-------------RAPNFRVTKDLIKIMNP------------------------------------------------ :  335
                   a             g

                      *        740        *        760        *        780        *        800        *        820        *       840
tr|Q9C1S6| : NHR-ESHDVETEISIPLYKLSLDASKGCSLRYIYRTLLMKDVKLTVACHFSLKTNDSVNFFKVWQPDENFSFEYDDGMRATVTTENSTESRCFLLRTTEEDTGENDWITKTINVPAVPEG :  581
tr|Q9ZB22| : E--------GEHAQIELYQTELPISEGTSLTWTFKSEHGNDLNV----GFRL------------DGEEDF--RYVEGEQR---------------------ESINGWTQWTLPLDAFAG- :  520
tr|Q93HW0| : G--------KTDQDVRLYSTKLEVTEKTKLRVAHKGGKGSKVYM----AFST------------TP--DY--KFDDADAWK------------------ELTLSDNWTNEEFDLSSLAG- :  659
sp|P36911| : ------------------------------------------------------------------------------------------------------------------------ :    -
sp|P04067| : ------------------------------------------------------------------------------------------------------------------------ :    -
sp|P36913| : ------------------------------------------------------------------------------------------------------------------------ :    -
tr|Q9APG4| : QSLEGLNKFKKLAQLDLIGL----SRITKL---DRSVLPANMKP--------GKDTLETVL------ETY--KKDNKEEPA------------------------TIPPVSLKVSGLTGL :  565
sp|P36912| : ------------------------------------------------------------------------------------------------------------------------ :    -

                      *        860        *        880        *        900        *        920        *        940        *       960
tr|Q9C1S6| : SQLYITRLEVSVVLDTAGLVGLVNQVIACLGYISI--------IPTINSG--------IKTDSSRIIQDLFWKDQKY-----TKIGKESLDDIAQEEVHRYYGTLNWEN---TANVVNAW :  677
tr|Q9ZB22| : QTITG----LAFAAE-GNETGLAEFY---IGQLAVGADSEKPAAPNVNVR--------Q-------------------------------YDPDP-----SGIQLVWEK---Q------- :  578
tr|Q93HW0| : KTIYA----VKLFFEHEGAVKDYQFN---LGQLTISDNHQEPQSPTSFSV--------VK-------QSL--------------------KNAQE-----AEAVVQFKG---N------- :  722
sp|P36911| : ------------------------------------------------------------------------------------------------------------------------ :    -
sp|P04067| : ------------------------------------------------------------------------------------------------------------------------ :    -
sp|P36913| : ------------------------------------------------------------------------------------------------------------------------ :    -
tr|Q9APG4| : KELDL------SGFDRETLAGLDAATLTSLEKVDISGNKLDLAPGTENRQIFDTMLSTISNHVGSNEQTVKFDKQKPTGHYPDTYGKTSLRLPVANEKVDLQSQLLFGTVTNQGTLINS- :  678
sp|P36912| : ------------------------------------------------------------------------------------------------------------------------ :    -
```

```
                         *       980         *       1000        *       1020        *       1040        *       1060        *       1080
tr|Q9C1S6| : EEIDYYNVFYKESDD-------SATRIFLGTAFCNQ---FRVSGLDIILSKLPKIVIEAVNKEGY----ISSSGSIDLSLN--------------------------------------- :  744
tr|Q9ZB22| : SNVHHYRVYKETKHGKELIGTSAGDRIYLEGLVEES----KQN------DVR--LHIEALSETFV----PSDARMIDI------KSGS-------------------F------------ :  645
tr|Q93HW0| : KDADFYEVYEKDGDSWKLLTGSSSTTIYLPKVSRSA----SAQ------GTTQELKVVAVGKNGV----RSEAATTTFDWGMTVKDTSLPKPLAENIVPGATVIDSTFPKTEGGEGIEGM :  828
sp|P36911| : ------------------------------------------------------------------------------------------------------------------------ :    -
sp|P04067| : ------------------------------------------------------------------------------------------------------------------------ :    -
sp|P36913| : ------------------------------------------------------------------------------------------------------------------------ :    -
tr|Q9APG4| : -EA-DYKAYQN----HKIAGRSFVDSNYHYNNFKVSYENYTVKVTDSTLGTTTDKTLATDKEETYKVDFFSPADKTKAVHTAKVI-VGDEKTMMVNLAEGATVIGGSADPVNARKVFDGQ :  791
sp|P36912| : ------------------------------------------------------------------------------------------------------------------------ :    -

                         *      1100         *       1120        *       1140        *       1160        *       1180        *       1200
tr|Q9C1S6| : ------------------------------------------------------------------------------------------------------------------------ :    -
tr|Q9ZB22| : ------------------------------------------------------------------------------------------------------------------------ :    -
tr|Q93HW0| : LNGTITSLSDKWSSAQLSGSVDIRLTKPRTVVRWVMDHAGAGGESVND--------GLMNTKDFDLYY-------KDADGEWKLAKEV--RGNKAHVTDITLDKPITAQDWRLNVVTSDN :  931
sp|P36911| : ------------------------------------------------------------------------------------------------------------------------ :    -
sp|P04067| : ------------------------------------------------------------------------------------------------------------------------ :    -
sp|P36913| : ------------------------------------------------------------------------------------------------------------------------ :    -
tr|Q9APG4| : LGSETDNISLGWDSKQS---IIFKLKEDGLIKHWRFFNDSARNPETTNKPIQEASLQIFNIKDYNLDNLLENPNKFDDEKYWITVDTYSAQGERATAFSNT-LNNITSKYWRVVFDTKG- :  906
sp|P36912| : ------------------------------------------------------------------------------------------------------------------------ :    -

                         *      1220         *       1240        *       1260        *       1280        *       1300        *       1320
tr|Q9C1S6| : ------------------------------------------------------------------------------------------------------------------------ :    -
tr|Q9ZB22| : ------------------------------------------------------------------------------------------------------------------------ :    -
tr|Q93HW0| : GTPWKAIRIYNWKMYEKLDTESVNIPMAKAAARSLGNNKVQVGFADVQAGATITVYDNPNSQTPLATLKSEVGGDLASAPLDLTNQSGLLYYRTQLPGKEISNVLAVSVPKDDRRIKSVS : 1051
sp|P36911| : ------------------------------------------------------------------------------------------------------------------------ :    -
sp|P04067| : ------------------------------------------------------------------------------------------------------------------------ :    -
sp|P36913| : ------------------------------------------------------------------------------------------------------------------------ :    -
tr|Q9APG4| : -DRYS-------------------SPV--------------------VPELQILGYPLPNADTIMKTVTTAK---------ELSQQKD------KFSQK----------MLDELKIKEMA :  961
sp|P36912| : ------------------------------------------------------------------------------------------------------------------------ :    -

                         *      1340         *       1360        *       1380        *       1400        *       1420        *       1440
tr|Q9C1S6| : ------------------------------------------------------------------------------------------------------------------------ :    -
tr|Q9ZB22| : ------------------------------------------------------------------------------------------------------------------------ :    -
tr|Q93HW0| : LETGPKKTSYAEGEDLDLRGGVLRVQYEGGTEDELIRLTHAGVSVSGFDTHHKGEQNLTLQYLGQPVNANLSVTVTGQDEASPKTILGIEVSQKPKKDYLVGDSLDLSEGRFAVAYSNDT : 1171
sp|P36911| : ------------------------------------------------------------------------------------------------------------------------ :    -
sp|P04067| : ------------------------------------------------------------------------------------------------------------------------ :    -
sp|P36913| : ------------------------------------------------------------------------------------------------------------------------ :    -
tr|Q9APG4| : LETSLNSKIFD-VTAINANAGVLKDCIEK---RQLLKK---------------------------------------------------------------------------------- :  995
sp|P36912| : ------------------------------------------------------------------------------------------------------------------------ :    -
```

```
                         *      1460         *      1480         *      1500         *      1520         *      1540         *      1560
tr|Q9C1S6| : ------------------------------------------------------------------------------------------------------------------------ :    -
tr|Q9ZB22| : ------------------------------------------------------------------------------------------------------------------------ :    -
tr|Q93HW0| : MEEHSFTDEGVEISGYDAQKTGRQTLTLRYQGHEVNFDVLVSPKAALNDEYLKQKLAEVEAAKNKVVYNFASPEVKEAFLKAIEAAEQVLKDHEISTQDQVNDRLNKLTEAHKALNGQEK : 1291
sp|P36911| : ------------------------------------------------------------------------------------------------------------------------ :    -
sp|P04067| : ------------------------------------------------------------------------------------------------------------------------ :    -
sp|P36913| : ------------------------------------------------------------------------------------------------------------------------ :    -
tr|Q9APG4| : ------------------------------------------------------------------------------------------------------------------------ :    -
sp|P36912| : ------------------------------------------------------------------------------------------------------------------------ :    -

                         *      1580         *      1600         *      1620         *      1640         *      1660         *      1680
tr|Q9C1S6| : ------------------------------------------------------------------------------------------------------------------------ :    -
tr|Q9ZB22| : ------------------------------------------------------------------------------------------------------------------------ :    -
tr|Q93HW0| : FKEEKTELDRLTGEVQELLDAKPNHPSGSALAPLLEKNKVLVEKVDLSPEELATAKQSLKDLVALLKEDKPAVFSDSKTGVEVHFSNKEKTVIKGLKVERVQASAEEKKYFAGEDAHVFE : 1411
sp|P36911| : ------------------------------------------------------------------------------------------------------------------------ :    -
sp|P04067| : ------------------------------------------------------------------------------------------------------------------------ :    -
sp|P36913| : ------------------------------------------------------------------------------------------------------------------------ :    -
tr|Q9APG4| : ------------------------------------------------------------------------------------------------------------------------ :    -
sp|P36912| : ------------------------------------------------------------------------------------------------------------------------ :    -

                         *      1700         *      1720         *      1740         *      1760         *      1780         *      1800
tr|Q9C1S6| : ------------------------------------------------------------------------------------------------------------------------ :    -
tr|Q9ZB22| : ------------------------------------------------------------------------------------------------------------------------ :    -
tr|Q93HW0| : IEGLDEKGQDVDLSYASIVKIPIEKDKKVKKVFFLPEGKEAVELAFEQTDSHVIFTAPHFTHYAFVYESAEKPQPAKPAPQNKVLPKPTYQPASDQQKAPKLEVQEEKVAFHRQEHENAE : 1531
sp|P36911| : ------------------------------------------------------------------------------------------------------------------------ :    -
sp|P04067| : ------------------------------------------------------------------------------------------------------------------------ :    -
sp|P36913| : ------------------------------------------------------------------------------------------------------------------------ :    -
tr|Q9APG4| : ------------------------------------------------------------------------------------------------------------------------ :    -
sp|P36912| : ------------------------------------------------------------------------------------------------------------------------ :    -

                         *      1820         *      1840         *      1860         *      1880         *      1900         *
tr|Q9C1S6| : ------------------------------------------------------------------------------------------------------------------- :    -
tr|Q9ZB22| : ------------------------------------------------------------------------------------------------------------------- :    -
tr|Q93HW0| : MLVGEQRVIIQGRDGLLRHVFEVDENGQRRLRSTEVIQEAIPEIVEIGTKVKTVPAVVATQEKPAQNTAVKSEEASKQLPNTGTADANEALIAGLASLGLASLALTLRRKREDKD : 1646
sp|P36911| : ------------------------------------------------------------------------------------------------------------------- :    -
sp|P04067| : ------------------------------------------------------------------------------------------------------------------- :    -
sp|P36913| : ------------------------------------------------------------------------------------------------------------------- :    -
tr|Q9APG4| : ------------------------------------------------------------------------------------------------------------------- :    -
sp|P36912| : ------------------------------------------------------------------------------------------------------------------- :    -
```

1.2 Overexpression and purification of EndoS mutants

Site directed mutagenesis was carried out using a Stratagene Quikchange II site-directed mutagenesis kit according to the manufacturer's instructions. The pGEX-4T-1-*ndoS* plasmid, which codes for GST-Tagged Endo S, was used as the template, and Pfa Ultra HF DNA Polymerase was used for PCR. Primers were designed using the primerX website and purchased from Sigma. In each case the DNA was transformed into XL-Gold Ultracompetent cells which were cultured on agar at 37 °C for 14 hours. Single colonies of cells were cultured in LB media containing ampicillin. After mini-prep, incorporation of the mutation was confirmed by DNA sequencing. The plasmids were each transformed into *E. coli* BL21(DE3) cells and the transformants subsequently cultured in LB medium containing ampicillin. Overexpression in LB medium containing ampicillin and ZYM-5052 media followed by lysis of the cell and purification by glutathione affinity chromatography gave the desired variants. If required, the GST tag was removed by treatment of the variant with Tev protease. The GST tag and the protease were removed by repeated dialysis using 30000 MWCO vivaspin columns.

1.3 Hydrolytic activity of EndoS mutants

The hydrolytic activity of the mutants of Endo S was investigated at low and high enzyme loading. Typically a mixture of Herceptin (2 mg/mL in PBS, 50 µg) and Endo S mutant were combined in PBS buffer to give a final concentration of 1 mg/mL Ab and a ratio of 10:1 or 100:1 Ab:Endo S. The reaction mixture was incubated at room temperature, aliquots (5 µL) were removed at various time points, snap-frozen and stored at -20 °C until analysis by SDS-PAGE under reducing conditions.

Densitometry analysis

Attempts to quantify the hydrolytic activity of EndoS mutants were carried out using densitometry. Commercial Herceptin gives a relatively broad band upon SDS-PAGE analysis, presumably due to the presence of multiple glycoforms. Complete separation of bands arising from glycosylated and trimmed Herceptin was not possible, so conversion levels calculated by densitometry are necessarily somewhat approximate. Densitometry analysis was carried out using Image Lab software. Destained gels were scanned and lanes were detected automatically. The detected lanes were adjusted to the observed bands as appropriate. Heavy chain bands were selected manually and adjusted to obtain consistent band widths. Light chain bands and those arising from Endo S were not selected. Normalisation was carried out against an internal standard (trimmed Herceptin, usually in band 1 or 2). The relative proportion of trimmed and glycosylated antibody were calculated as percentages.

Endo S Y305F At 100:1 Ab:enzyme the deglycosylation was complete after around 4.5 hours (figure S1).

A)

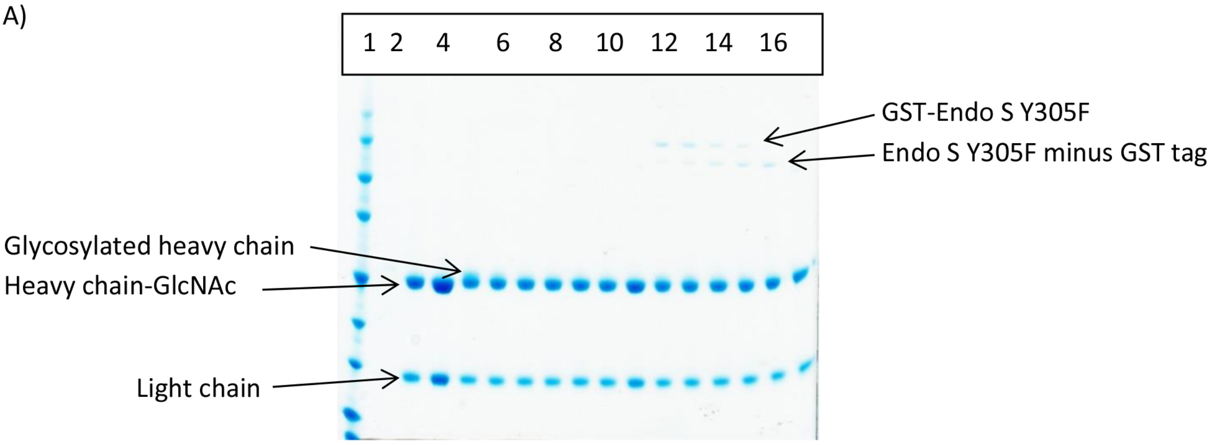

B)

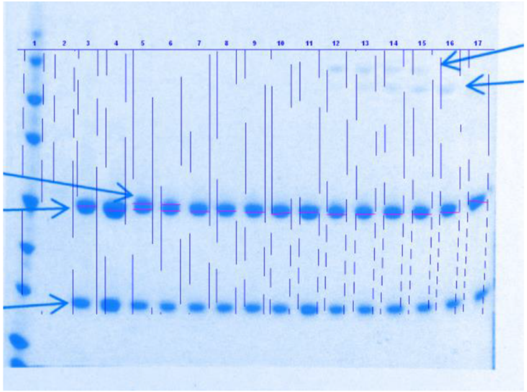

	Band No.	Band %
Lane 1		
Lane 2		
Lane 3	1	100
Lane 4	1	100
Lane 5	1	41.82521
	2	58.17479
Lane 6	1	35.16088
	2	64.83912
Lane 7	1	100
Lane 8	1	100
Lane 9	1	100
Lane 10	1	100
Lane 11	1	100
Lane 12	1	100
Lane 13	1	100
Lane 14	1	100
Lane 15	1	100
Lane 16	1	100
Lane 17	1	100

Figure S1. A) SDS-PAGE and B) densitometry analysis. Lane 1: marker. Lanes 3 and 11: IgG-GlcNAc. Lane 4: aglucosylated IgG (after PNGase F treatment). Lanes 5-10: 100:1 herceptin:Endo S Y305F after 1, 2, 4.5, 6.5, 21.5 and 46 hours. Lanes 12-17: 10:1: herceptin:Endo S Y305F after 1, 2, 4.5, 6.5, 21.5 and 46 hours.

Endo S D233E At 100:1 Ab:enzyme the deglycosylation was incomplete after 21 hours; at 10:1 Ab:enzyme deglycoslyation was complete after around five hours (figure S2).

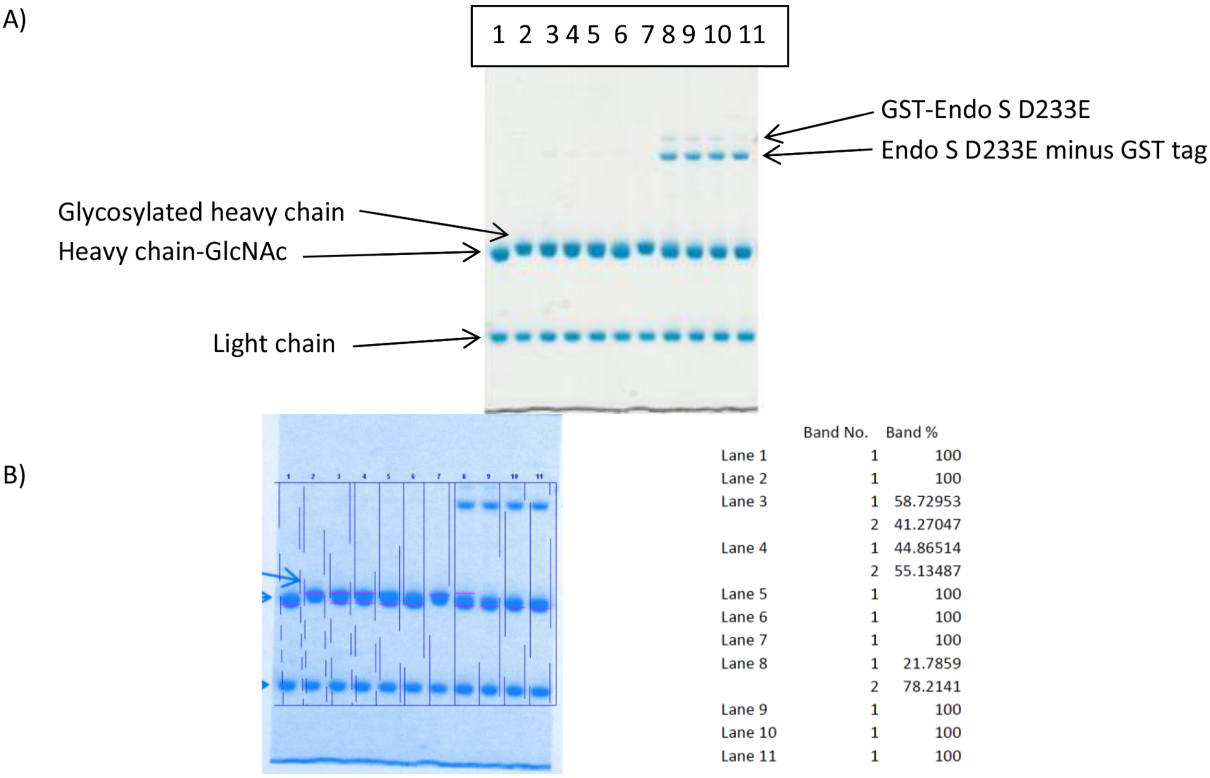

	Band No.	Band %
Lane 1	1	100
Lane 2	1	100
Lane 3	1	58.72953
	2	41.27047
Lane 4	1	44.86514
	2	55.13487
Lane 5	1	100
Lane 6	1	100
Lane 7	1	100
Lane 8	1	21.7859
	2	78.2141
Lane 9	1	100
Lane 10	1	100
Lane 11	1	100

Figure S2. A) SDS-PAGE and B) densitometry analysis. Lane 1: IgG-GlcNAc. Lane 2: Herceptin. Lanes 3-6: 100:1 herceptin:Endo S D233E after 1, 2, 5.5 and 21 hours. Lane 7: Herceptin. Lanes 8-11: 10:1: herceptin:Endo S D233E after 1, 2, 5.5 and 21 hours.

Endo S D233Q At 100:1 the level of deglycosylation was around 27% after 3.5 hours. Deglycosylation was incomplete after 21 hours at 10:1 Ab:enzyme (figure S3).

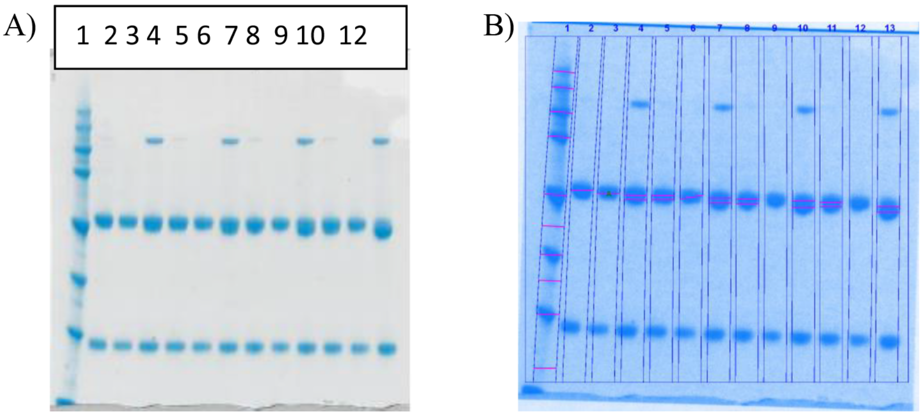

	Band No.	Band %
Lane 1		
Lane 2	1	100
Lane 3	1	64.88702
	2	35.11298
Lane 4	1	67.75668
	2	32.24332
Lane 5	1	72.05295
	2	27.94705
Lane 6	1	80.60482
	2	19.39519
Lane 7	1	78.15362
	2	21.84639
Lane 8	1	62.10683
	2	37.89317
Lane 9	1	59.79548
	2	40.20452
Lane 10	1	100

Figure S3. A) SDS-PAGE and B) densitometry analysis. Lane 1: Marker. Lanes 2, 5, 8 and 11: 100:1 herceptin:Endo S D233Q after 1, 3.5, 7.5 and 21 hours. Lanes 3, 6, 9, 12: Herceptin. Lanes 4, 7, 10, 13: 10:1: herceptin:Endo S D233Q after 1, 3.5, 7.5 and 21 hours.

Endo S D233A Deglycosylation was incomplete after 23 hours at 100:1 Ab:enzyme, but around 85% complete after 3 hours at 10:1 Ab:enzyme (figure S4).

Endo S D233A:Q303E Deglycosylation was incomplete after 23 hours at 100:1 Ab:enzyme, but complete after 23 hours at 10:1 Ab:enzyme (figure S4).

A)

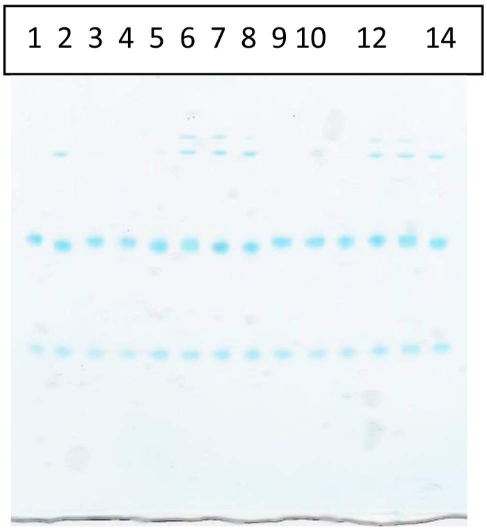

D233A

	Band No.	Band %
Lane 1	1	100
Lane 2	1	100
Lane 3	1	69.33661
	2	30.66339
Lane 4	1	71.84319
	2	28.15681
Lane 5	1	54.5628
	2	45.4372
Lane 6	1	43.93748
	2	56.06252
Lane 7	1	16.24928
	2	83.75072
Lane 8	1	100

D233A:E303Q

	Band No.	Band %
Lane 9	1	100
Lane 10	1	68.7078
	2	31.2922
Lane 11	1	63.25417
	2	36.74583
Lane 12	1	66.72037
	2	33.27963
Lane 13	1	61.19535
	2	38.80465
Lane 14	1	100

Figure S4. A) SDS-PAGE and B) densitometry analysis. Lane 1: Herceptin. Lane 2: IgG-GlcNAc. Lanes 3, 4, 5: 100:1 herceptin:Endo S D233A after 1, 3, and 23 hours. Lanes 6, 7, 8: 10:1 herceptin:Endo S D233A after 1, 3, and 23 hours. Lanes 9, 10, 11: 100:1: herceptin:Endo S D233A:Q303E after 1, 3 and 23 hours. Lanes 12, 13, 14: 10:1: herceptin:Endo S D233A:Q303E after 1, 3 and 23 hours.

The brief study of hydrolytic activities indicated that all the mutants of Endo S investigated retain some activity. Qualitatively the D233Q and the D233A:E303E mutants demonstrated the lowest activity and the Y305F mutant displayed the highest hydrolytic activity.

Semi-quantitatively, the hydrolytic activity of the various mutants (at 100:1 Ab:enzyme, after 5 hours reaction time) are given by the approximate levels of hydrolysis (‘H’ in manuscript):

WT: 100%

Y305F: 100%

D233E: 55%

D233A: 30%

D233A/Q303E: 25%

D233Q: 20%

The activity of endohexosaminidases has been reported to be influenced by the use of organic co-solvents such as DMSO and acetone.[1] We investigated the use of acetone or DMSO as

co-solvents but did not observe any change in the hydrolytic activity of Endo S, at levels of up to 30% DMSO. This indicates that Endo S is robust in terms of solvent tolerance.

2.1 Materials and general methods: chemical

Chemicals were purchased from Sigma-Aldrich and used as supplied, unless otherwise stated. Proton nuclear magnetic resonance (^{1}H NMR) spectra were recorded on a Bruker AVIII 700 (700 MHz), a Bruker AVII500 (500 MHz) or a Varain Mercury VX 400 (400 MHz) spectrometer, as indicated. Carbon nuclear magnetic resonance (^{13}C NMR) spectra were recorded on a Bruker AVIII 700 (176 MHz), Bruker AVII500 (125.8 MHz) or a Varain Mercury VX 400 (100.6 MHz) spectrometer, as indicated. NMR spectra were assigned using COSY, HSQC, HMBC and TOCSY correlation experiments. All chemical shifs are quoted on δ scale in ppm using residual solvent as the internal standard. Coupling constants (*J*) are reported in Hz with the following splitting abbreviations: s = singlet, d = doublet, t = triplet, q = quarter, app = apparent.

Low resolution mass spectra (LRMS) were recorded on a Waters Micromass LCT premier TOF spectrometer using electrospray ionization (ESI-MS) and high resolution mass spectra (HRMS) were recorded on a Bruker MicroTOF ESI mass spectrometer.

Melting points (m.p.) were recorded on a Leica Galen III hot stage microscope equipped with a Testo 720 thermocouple probe and are uncorrected. Optical rotations were measured on a Perkin-Elmer 241 polarimeter or a Schmidt-Haensch Unipol L series polarimeter with a path length of 1.0 dm and are reported with implied units of 10^{-1} deg cm^{2} g^{-1}. Concentrations (c) are given in g/100 ml. Thin layer chromatography (TLC) was carried out using Merck aluminium backed sheets coated with 60F254 silica gel. Visualization of the silica gel plates was achieved using a UV lamp (λ_{max} = 254 nm) and/or by dipping the plate in sulphuric acid stain followed by heating.

2.2 Chemical Synthesis

2.2.1 Prior Methods

2-Chloro-1,3-dimethyl-1*H*-benzimidazol-3-ium chloride (CDMBI) was prepared from 2-hydroxybenzimidazole as described previously.[2]

2-(2-Azidoethoxy)ethanaminium 2,2,2-trifluoroacetate was prepared from 2-(2-aminoethoxy)ethanol as described previously.[3] Following solvent removal the residue was dissolved in water and the aqueous solution was frozen and lyophilized to remove residual trifluoroacetic acid. The salt was isolated as the monohydrate, as a very pale yellow oil, ν_{max} (neat): 2884, 2110, 1780, 1672 cm^{-1}; δ_H (400 MHz, CD_3OD) 3.14 (2H, t, J_{H1-H2} 5.2Hz, H_4), 3.46 (2H, t, J_{H1-H2} 5.2Hz, H_1), 3.71 (2H, t, J_{H3-H4} 5.2Hz, H_3), 3.72 (2H, t, J_{H2-H1} 5.2Hz, H_2); δ_C (125.8 MHz, CD_3OD) 39.2 (CH_2NH_2), 50.2 (CH_2N_3), 66.4, 69.6 (2 x CH_2O), 118.0 (CF_3), 160.9 (C=O); *m/z/* (ES^+) 228.1 (MNa^+, 100%).

2-(Pyridyldithio)-ethylamine hydrochloride was prepared as described previously.[4]

2.2.2 Synthesis of sugars

The extraction and purification of the complex biantennary glycan from hen egg yolk was carried out using minor modifications to the previously described procedures.[5]

Extraction of sialylglycopeptide (SGP) from egg

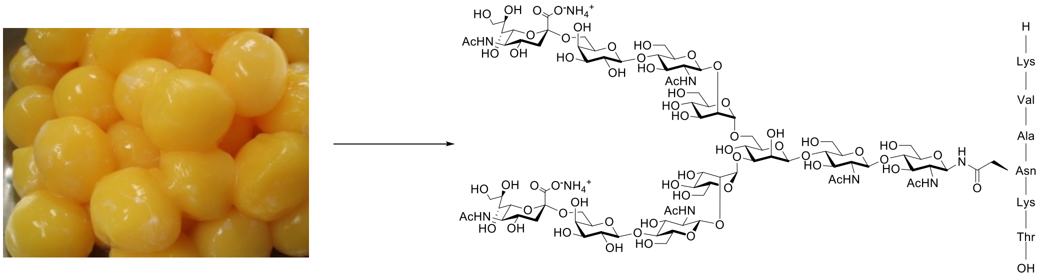

The yolks from 30 hen eggs (520 g yolk, Tesco large size eggs) were suspended in EtOH (800 mL) and mixed thoroughly. The suspension was centrifuged (J500, 8000 rpm, 4 °C, 10 mins in polypropylene centrifuge tubes) and the supernatant discarded. The pellet was re-suspended in EtOH (800 mL), mixed thoroughly and the resulting suspension centrifuged (J500, 8000 rpm, 4 °C, 10 mins). The procedure was repeated once more (i.e. total of three times, 2400 mL EtOH).

The resultant pellet was suspended in water (500 mL), mixed thoroughly and centrifuged (J500, 8000 rpm, 4 °C, 10 mins). The supernatant was collected and the pellet was re-suspended in water (500 mL), mixed thoroughly and centrifuged (J500, 8000 rpm, 4 °C, 10 mins). The procedure was repeated thrice more (i.e. total of five times, 2500 mL water).

The oil phase was separated from the aqueous and discarded; the aqueous phase was filtered through celite and stored at 4 °C overnight.

The aqueous fraction was warmed to rt and concentrated *in vacuo* to around 200 mL. During this concentration step the solution is prone to bumping and bubbling so an oversized flask and a splash head were utilised. The small amount of precipitate formed upon concentration was removed by filtration through celite. Ethanol (1400 mL) was added to the supernatant to precipitate the SGP, which was collected by centrifugation (J500, 8000 rpm, 4 °C, 10 mins). The pellet was collected, suspended fully in water (200 mL) with thorough mixing, and the mixture centrifuged (JA-25, 14000 rpm, 4 °C, 10 mins) to remove small quantities of insoluble material (around 500 mg solid was removed). Ethanol (1400 mL) was added to the supernatant, the mixture centrifuged (JLA 16.250, 10000 rpm, 4 °C, 6 mins) and the supernatant discarded. The pellet was again suspended in water (200 mL) and the procedure above repeated once more (i.e. total of three times, 4200 mL EtOH, 600 mL water). The pellet was collected and dried under high vacuum (~20 h) to afford target sialylglycopeptide contaminated with amino acid and peptide impurities (7.26 g) as a pale yellow foam.

Preparation of glycosyl asparagine

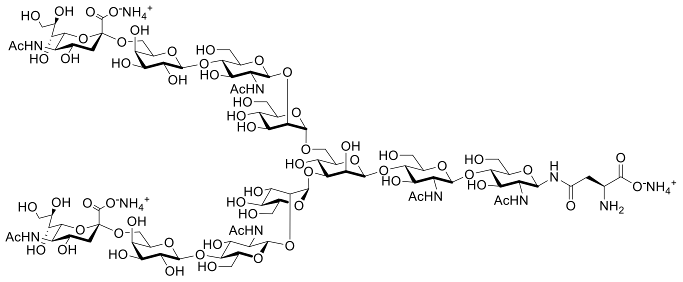

The SGP (6.6 g) isolated from hen egg yolk was dissolved in buffer solution (280 mL total, 40 mL per gram of crude SGP, 200 mM Tris·HCl, 10 mM $CaCl_2$, 0.01% NaN_3, pH 8.0) in six falcon tubes. The crude SGP was only partially soluble at room temperature but upon warming to 37 °C the material dissolved readily. After the SGP was dissolved the pH of the solution was 7.85. Actinase E (660 mg total, 10 wt%, protease from *Streptomyces griseus*, Sigma) was added and the mixture incubated at 37 °C for two days. Reaction progress was monitored by t.l.c. analysis (1 M ammonium acetate: *iso*-propanol, 1:1); starting glycopeptide was visible after sulphuric acid staining as a streak from the baseline to R_f 0.4; glycosyl asparagine R_f 0.64. Further actinase E (30 mg to each falcon tube) was added and the mixture incubated at 37 °C for a further two days. After this time the mixture was cooled to rt and centrifuged (JLA 16.250, 10000 rpm, 4 °C, 6 mins). The supernatant was retained and the pellet was re-suspended in water (10 mL) and centrifuged (JLA 16.250, 20000 rpm, 4 °C, 5 mins). This procedure was repeated once more. The aqueous fractions were analysed by t.l.c. (1 M ammonium acetate: *iso*-propanol, 1:1) and those containing target material were pooled, concentrated to around 60 mL and filtered (0.8 then 0.45 then 0.2 µm membranes) and subjected to FPLC purification.

[If necessary the filtrate may be stored at 4 °C overnight. Further precipitation may be observed in which case it may be removed by centrifugation, the pellet re-extracted and analysed as above and the aqueous fraction containing target material subsequently filtered (0.2 µm membrane) prior to FPLC purification.]

FPLC purification was carried out in batches using a HiPrep 26/10 desalting column (GE Healthcare Life Sciences) connected to an AKTA FPLC system. Samples (7 mL) were loaded onto the column and eluted with ammonium bicarbonate (5 mM aqueous solution). Sample injection was carried out *via* a Superloop, at a flow rate of 2 mL/min. Column equilibration and sample elution was carried out at a flow rate of 4 mL/min. Prior to sample injection the column was equilibrated with one column volume of buffer. After sample injection the samples were eluted with two column volumes of buffer, and collected as 6 mL fractions. Fractions were analysed by t.l.c. (1 M ammonium acetate: *iso*-propanol, 1:1); those containing target glycosyl asparagine (generally fractions 2-8) were combined, concentrated *in vacuo* to around 20 mL and lyophilised to afford the target (992 mg) as a white solid.

Fmoc protection of SGAsn

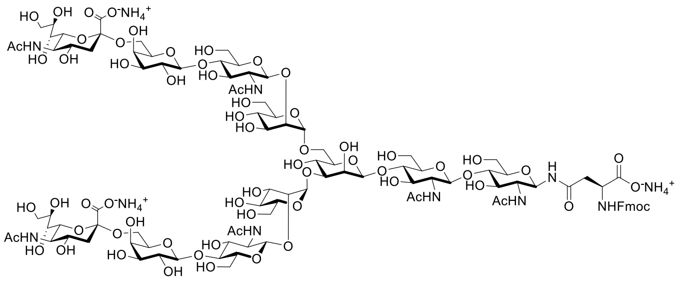

Glycosyl asparagine (950 mg) was dissolved in buffer (9.5 mL of 1 M $Na_2CO_3/NaHCO_3$, pH 9). 9-Fluorenylmethyl *N*-succinimidyl carbonate (Fmoc-succinimide) (2.85 g, 300 wt%) was dissolved in dioxane (9.5 mL) and added to the solution of glycosyl asparagine. The mixture was stirred at rt for 2 hours before the addition of further Fmoc-succinimide (600 mg). After a further 40 minutes the reaction mixture was filtered (gravity filtration through paper), the white precipitate thus removed was washed twice with water (5 mL). The combined aqueous fractions were washed with ethyl acetate (3 x 5 mL). The combined organics were re-extracted with water (2 x 4 mL) until t.lc. analysis (1 M ammonium acetate: *iso*-propanol, 2:3) indicated that no target material remained in the organic fraction. The aqueous fractions were pooled, filtered (0.45 µm polyamide syringe filter) and purified in batches by FPLC using a HiPrep 26/10 desalting column (GE Healthcare Life Sciences) connected to an AKTA FPLC system. Samples (5 mL) were loaded onto the column and eluted with ammonium bicarbonate (5 mM aqueous solution). Sample injection was carried out *via* a Superloop, at a flow rate of 2 mL/min. Column equilibration and sample elution was carried out at a flow rate of 4 mL/min. Prior to sample injection the column was equilibrated with one column volume of buffer. After sample injection the samples were eluted with 1.5 column volumes of buffer, and collected as 3 mL fractions. Fractions were analysed by t.l.c. (1 M ammonium acetate: *iso*-propanol, 1:1); those containing target glycosyl asparagine (generally fractions 4-12) were combined, concentrated *in vacuo* to around 5 mL and lyophilised to afford the target (710 mg) as an off-white solid. The solid was dissolved in water (4 mL), filtered (0.8 µm membrane) and subjected to HPLC purification using a Synergi 4u, Hydro-RP 80Å, 100 x 21.2 mm, 4 micron column (Phenomenex). Buffer A : 5 mM ammonium acetate. Buffer B: 5 mM ammonium acetate in 80% acetonitrile. A gradient elution was used comprising the following stages: 2 minute equilibration with 3% B, gradual gradient to 10% B over 13 minutes and maintain at 10% B for 2 minutes. Increase to 100% B over 1 minute, maintained at 100% B for 2 minutes then re-equilibrate at 3% B for 2 minutes, all at a flow rate of 15 mL/min. Detection was by UV absorption, with monitoring at 214, 254, 280 and 300 nm. Injections were typically between 400 and 800 µL. The product eluted as a broad peak with T_R around 6.5 mins. Fractions containing target compound were combined, concentrated *in vacuo* to around 5 mL, transferred to two falcon tubes and lyophilized to afford the triammonium salt of Fmoc protected SGAsn (210 mg) as white foam. Also isolated was a mixture of monosialylated and monogalactosylated Fmoc-protected SGAsn (52.5 mg) T_R around 19 minutes, as a white foam.

Disialylated material (triacid form): *m/z* (ESI$^+$) 2577 ($M.NH_4^+$, 3), 1299 ($M.NH_4^+.Na^+$, 55), 1290 ($M.NH_4^+.H^+$, 100%).

Monosialylated material (diacid form): *m/z* (ESI$^+$) 2286 ($M.NH_4^+$, 28), 1154 ($M.NH_4^+.Na^+$, 22), 1144 ($M.NH_4^+.H^+$, 100%).

Monogalactosylated material (diacid form): *m/z* (ESI$^+$) 2125 ($M.NH_4^+$, 63), 1063 ($M.NH_4^+.H^+$, 100%).

Ion-exchange of triammonium salt to trisodium salt

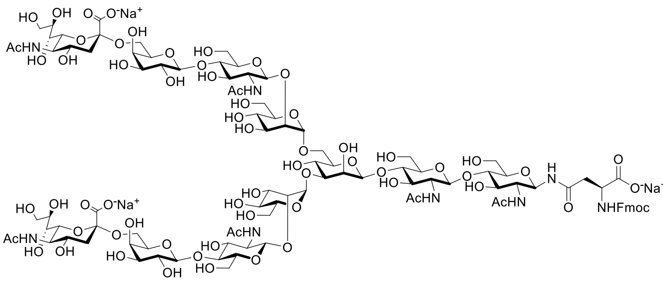

Dowex 50WX8 100-200 mesh, hydrogen form (22 mL wet volume in water) was loaded onto a syringe column and activated with sodium hydroxide (10 column volumes of a 1 M aqueous solution). The column was washed sequentially with water (4 column volumes), methanol (4 column volumes) and water (8 column volumes). The triammonium salt (210 mg) was applied as a solution in water and the product eluted with water (10 x 8 mL fractions). Fractions were analysed by t.l.c. (1 M ammonium acetate: *iso*-propanol, 2:3) and those containing target material (fractions 1 and 2) were combined and lyophilised to afford the trisodium salt (204 mg) as a white powder; $[\alpha]_D^{20}$= -10.2° (c= 0.50, H_2O); ν_{max} (single crystal): 3294, 2930, 1611, 1556, 1377 cm^{-1}; ^1H NMR[6] (700 MHz, D_2O, δ (acetone)= 2.220): 1.72 (2H, dd, $J_{H3ax\text{-}H3eq}$ 12.6Hz, $J_{H3\text{-}H4}$ 12.6Hz, $H3g_{ax}$, $H3g'_{ax}$), 1.89 (2.25H, s, $COCH_3$a (major conformer)), 1.98 (0.75H, s, $COCH_3$a (minor conformer)), 2.03 (6H, s, $COCH_3$ x 2), 2.067, 2.071 (9H, s x 2, $COCH_3$ x 3), 2.27-2.34 (0.25H, m, $^{Asn}H_\beta$ (minor conformer)), 2.48-2.54 (0.25H, m, $^{Asn}H_\beta$' (minor conformer)), 2.53 (0.75H, dd, $J_{H\beta\text{-}H\beta'}$ 15.4Hz, $J_{H\beta\text{-}H\alpha}$ 9.1Hz, $^{Asn}H_\beta$ (major conformer)), 2.669 (1H, dd, $J_{H3eq\text{-}H3ax}$ 12.6Hz, $J_{H3eq\text{-}H4}$ 4.2Hz, $H3g_{eq}$ or $H3g'_{eq}$), 2.675 (1H, dd, $J_{H3eq\text{-}H3ax}$ 12.6Hz, $J_{H3eq\text{-}H4}$ 4.2Hz, $H3g_{eq}$ or $H3g'_{eq}$), 2.72 (0.75H, dd, $J_{H\beta'\text{-}H\beta'}$ 15.4Hz, $J_{H\beta'\text{-}H\alpha}$ 4.2Hz, $^{Asn}H_\beta$' (major conformer)), 3.41-3.46 (0.75H, m, H1a (major conformer)), 3.46-3.58 (9.25H, m, H5a (minor conformer), H6a, H4d, H4d', H2f, H2f', H6f, H6f', H7g, H7g'), 3.58-3.68 (17H, m, H4a, H6'a, H5b, H5c, H5d', H6d, H6d', H4e, H4e', H5e, H5e', H3f, H3f', H4g, H4g', H9g, H9g'), 3.68-3.84 (20H, m, H2a, H3a, H2b, H3b, H4b, H6b, H3c, H4c, H6c, H5d, $^eH_2$, H2e', H3e, H3e', H5f, H5f', H5g, H5g', H6g, H6g'), 3.84-3.94 (13H, m, H6'b, H3d, H3d', H6'd, H6'd', H6e, H6e', H4f, H4f', H8g, H8g', H9'g, H9'g'), 3.94-4.02 (5H, m, H6'c, H6'e, H6'e', H6'f, H6'f'), 4.05-4.10 (0.25H, m, $^{Asn}H_\alpha$ (minor conformer)), 4.12 (1H, d, $J_{H2\text{-}H3}$ 3.5Hz, H2d'), 4.20 (1H, d, $J_{H2\text{-}H3}$ 3.5Hz, H2d), 4.25 (1H, s, H2d), 4.25-4.28 (0.75H, m, $^{Asn}H_\alpha$ (major conformer)), 4.31 (1H, t, $J_{H3\text{-}H2}$ 6.3Hz,

^{Fmoc}H3), 4.439 (1H, d, J_{H1-H2} 8.4Hz, H1f or H1f'), 4.425 (1H, d, J_{H1-H2} 7.7Hz, H1f' or H1f), 4.48 (0.75H, dd, $J_{H2-H2'}$ 11.2Hz, J_{H2-H3} 6.3Hz, ^{Fmoc}H2 (major conformer)), 4.48-4.53 (0.50H, m, ^{Fmoc}H2 (minor conformer)), 4.54 (0.75H, dd, $J_{H2'-H2}$ 11.2Hz, $J_{H2'-H3}$ 6.3Hz, ^{Fmoc}H2' (major conformer)), 4.57 (1H, d, J_{H1-H2} 7.7Hz, H1b), 4.60 (2H, d, J_{H1-H2} 6.3Hz, H1e, H1e'), 4.76 (1H, s, H1c), 4.95 (1H, s, H1d'), 5.00 (1H, d, J_{H1-H2} 9.1Hz, H1a), 5.14 (1H, s, H1d), 7.43 (1H, td, $J_{H7-H6,8}$ 7.7Hz, J_{H7-H5} 4.9Hz, ^{Fmoc}H7), 7.50 (1H, t, $J_{H6-H5,7}$ 7.7Hz, ^{Fmoc}H6), 7.70 (1H, dd, J_{H5-H6} 7.0Hz, J_{H5-H7} 4.9Hz, ^{Fmoc}H5), 7.90 (1H, d, J_{H8-H7} 7.7Hz, ^{Fmoc}H8); ^{13}C NMR (176 MHz, D_2O): 22.6 (^{a}CO*C*H_3), 22.7, 22.7, 22.9, 23.1, 23.1 (CO*C*H_3 x 5), 39.2 ($^{Asn}C_\beta$), 40.7, 40.7 (C3g, C3g'), 47.5 (^{Fmoc}C3, minor conformer), 47.6 (^{Fmoc}C3, major conformer), 52.5, 52.5 (C5g, C5g'), 53.4 ($^{Asn}C_\alpha$, major conformer), 54.0 ($^{Asn}C_\alpha$, minor conformer), 54.4 (C2a), 55.3, 55.3 (C2e, C2e'), 55.5 (C2b), 60.4 (C6a), 60.6 (C6b), 60.9, 60.9 (C6e, C6e'), 62.3 (C6d'), 62.4 (C6d), 63.3, 63.3 (C9g, C9g'), 64.0, 64.0 (C6f, C6f'), 66.4 (C4c), 66.5 (C6c), 67.1 (^{Fmoc}C2, major conformer), 67.7 (^{Fmoc}C2, minor conformer), 67.9 (C4d), 68.0 (C4d'), 68.9, 68.9 (C4g, C4g'), 69.0, 69.0 (C4f, C4f'), 69.1, 69.1 (C7g, C7g'), 70.1 (C3d), 70.1 (C3d'), 70.9 (C2c), 71.4, 71.4 (C2f, C2f'), 72.4, 72.4 (C8g, C8g'), 72.6 (C3b), 72.7, 72.8 (C3e, C3e'), 73.1, 73.1 (C3f, C3f'), 73.2, 73.2 (C6g, C6g'), 73.4 (C3a), 73.5 (C5d'), 74.2 (C5d), 74.4, 74.4 (C5f, C5f'), 75.0, 75.0 (C5b, C5c), 75.1, 75.1 (C5e, C5e'), 76.8 (C5a), 76.9 (C2d'), 77.1 (C2d), 78.8 (C1a), 79.3 (C4a), 80.3 (C4b), 81.1 (C3c), 81.3, 81.4 (C4e, C4e'), 97.6 (C1d'), 99.9, 100.0 (C1e, C1e'), 100.2 (C1d), 100.8, 100.8 (C2g, C2g'), 101.1 (C1c), 102.0 (C1b), 104.2, 104.2 (C1f, C1f'), 120.9 (^{Fmoc}C8), 125.8 (^{Fmoc}C5, major conformer), 125.9 (^{Fmoc}C5, minor conformer), 128.2, 128.2 (^{Fmoc}C6, two conformers), 128.7, 128.7 (^{Fmoc}C7, two conformers), 141.6 (^{Fmoc}C9), 144.4 (^{Fmoc}C4, minor conformer), 144.5 (^{Fmoc}C4, major conformer), 158.3 (^{Fmoc}C1), 174.1 ($^{Asn}C_\gamma$), 174.2, 174.2 (C1g, C1g'), 175.3, 175.4, 175.4, 175.6, 175.6 (*C*OCH_3 x 6), 178.0 (^{Asn}C1); coupled HSQC: *J*(H1a-C1a) 156Hz, *J*(H1b-C1b) 164Hz, *J*(H1c-C1c) 160Hz, *J*(H1d-C1d) 171Hz, *J*(H1d'-C1d') 170Hz, *J*(H1e-C1e) 162Hz, *J*(H1f-C1f) 162Hz; MALDI-TOF-MS (matrix: DHB): [trisodium form + Na]$^+$ 2647.73.

Decasaccharide lactol ***4***

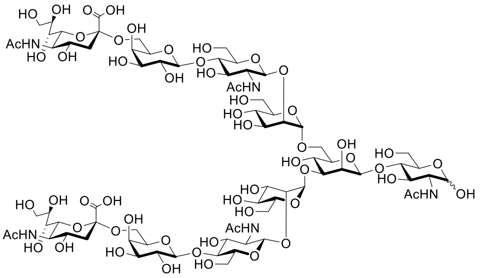

The trisodium salt of Fmoc-SGAsn (22.0 mg, 8.59 μmol) was dissolved in 150 μL of PBS. GST-EndoS (150 μL of a 14.7 mg/mL solution) was added. The pH of the reaction mixture was measured and if necessary adjusted by addition of phosphate buffer (pH 6.5, 500 mM) to give a final pH around 7. The mixture was incubated at 37 ºC and reaction progress monitored by t.l.c. (3:2 *iso*-propanol-1 M ammonium acetate) and ESI-MS. After 3 days the

reaction appeared complete; the mixture was filtered through a 0.2 μm centrifugal filter and applied to a PD Miditrap G25 column. The product was eluted with water; pure fractions containing glycan were collected and lyophilised to afford the target lactol as a white fluffy solid, 17.5 mg (quant.); ν_{max} (single crystal) 3285, 1635, 1377 cm^{-1}; δ_H (500 MHz, D_2O) 1.63 (2H, t, *J* 12.1 Hz, H3f_{ax}, H3f'$_{ax}$), 1.94, 1.98, 1.98 (15H, 3 x s, 5 x CH_3), 2.58 (2H, dd, J_{3eq-4} 4.4 Hz, $J_{3eq-3ax}$ 12.3 Hz, H3f_{eq}, H3f'$_{eq}$), 3.39-3.48 (8H, m, H4c, H4c', H2e, H2e', H6e, H6e', H7f, H7f'), 3.48-3.61 (17H, m, H5a, H5b, H5c', H6c, H6c', H4d, H4d', H5d, H5d', H3e, H3e', H4f, H4f', H6f, H6f', H9f, H9f'), 3.61-3.75 (16H, m, H3a, H4a, H6a, H6'a, H3b, H4b, H6b, H5c, H2d, H2d', H3d, H3d', H5e, H5e', H5f, H5f'), 3.75-3.92 (18H, m, H2a, H6'b, H3c, H3c', H6'c, H6'c', H6d, H6d', H6'd, H6'd', H4e, H4e', H6'e, H6'e', H8f, H8f', H9'f, H9'f'), 4.03 (1H, d, *J* 2.2 Hz, H2c'), 4.11 (1H, d, *J* 2.0 Hz, H2c), 4.18 (1H, d, *J* 4.4 Hz, H2b), 4.35 (2H, d, J_{1-2} 7.9 Hz, H1e, H1e'), 4.52 (2H, d, J_{1-2} 7.8 Hz, H1d, H1d'), 4.63 (0.3H, d, J_{1-2} 7.9 Hz, H1a(β)), 4.70 (1H, d, H1b), 4.87 (1H, s, H1c'), 5.05 (1H, s, H1c), 5.13 (0.7H, d, J_{1-2} 3.3 Hz, H1a(α)); δ_C (125.8 MHz, D_2O) 21.9, 22.0, 22.3, 22.4, 22.4 (5 x CH_3), 40.0 (C3f, C3f'), 51.8 (C5f, C5f'), 54.1 (C2a_α), 54.6 (C2d, C2d'), 56.0 (C2a), 60.4 (C6a_β), 60.2 (C6a), 60.4, (C6d, C6d'), 61.6, 61.7 (C6c, C6c'), 62.4, 62.6 (C9f, C9f'), 63.3 (C6e, C6e'), 65.7 (C4b), 65.9 (C6b), 67.2, 67.3 (C4c, C4c'), 68.2 (C4f, C4f'), 68.3, 68.4 (C4e, C4e'), 69.3, 69.4 (C7f, C7f'), 69.4, 69.5 (C3c, C3c'), 70.2 (C2b), 70.7 (C2e, C2e'), 71.7 (C8f, C8f'), 72.0 (C3d, C3d'), 72.1 (C3a), 72.4 (C3e, C3e'), 72.5 (C6f, C6f'), 72.8 (C5c'), 73.5 (C5c), 73.7 (C5e, C5e'), 74.3, 74.3 (C5a, C5b), 74.4 (C5d, C5d'), 76.2 (C2c'), 76.4 (C2c), 80.2 (C4a), 80.5 (C2b), 80.6, 80.7 (C4d, C4d'), 90.4 (C1a_α), 94.9 (C1a_β), 96.9 (C1c'), 99.2, 99.3 (C1d, C1d'), 99.5 (C1c), 100.1, 100.1 (C2f, C2f'), 100.4 (C1b), 103.5 (C1e, C1e'), 173.5 (C1f, C1f'), 174.5, 174.7, 174.7, 174.8, 174.9 (5 x C=O); LRMS (ESI$^+$): *m/z* 2022 (MH^+, 5), 1030 ($M{\cdot}2Na^+$, 55), 1022 ($M{\cdot}Na^+{\cdot}H^+$, 100), 1011 ($M{\cdot}2H^+$, 61%); HRMS (ESI$^+$) ion series calculated for $C_{76}H_{125}N_5O_{57}$: 1032.84104, 1033.34272, 1033.84440, 1034.34487, 1034.84654, observed: 1032.84435, 1033.34590, 1033.84768, 1034.34979, 1034.85252.

Decasaccharide oxazoline ***2***[2, 7]

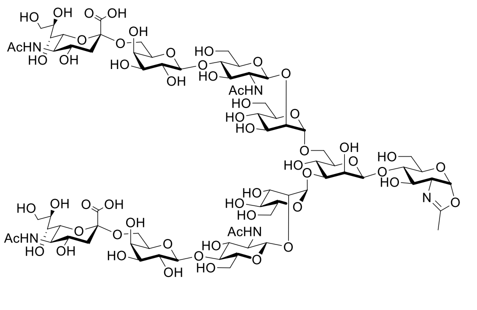

Lactol **4** (1.4 mg, 0.693 μmol) was dissolved in water (28 μL). CDMBI (1.5 mg, 6.93 μmol) was added, followed by potassium phosphate tribasic (28 μL of a 0.75 M solution). The mixture was vortexed and the shaken at 4 °C for 4 hours. After this time ESI-MS analysis indicated complete consumption of lactol and formation of oxazoline product. The mixture was filtered through a centrifugal spin filter (0.2 μm membrane) and applied to a PD Minitrap

G25 column. The product was eluted with 0.01% ammonia solution; pure fractions containing glycan were collected and lyophilised to afford **2** as a white fluffy solid, 1.0 mg (72%); $[\alpha]_D^{25}$ = + 2.5° (c = 0.16, H_2O); ν_{max} (single crystal) 3270, 1608 cm^{-1}; δ_H (700 MHz, D_2O) 1.58 (2H, t, *J* 10.7 Hz, $H3f_{ax}$, $H3f'_{ax}$), 1.88, 1.92 (15H, 2 x s, 5 x CH_3), 2.52 (2H, br d, *J* 9.8 Hz, $H3f_{eq}$, $H3f'_{eq}$), 3.28-3.43 (9H, m, H5a, H4c, H4c', H2e, H2e', H7f, H7f', H9f, H9f'), 3.43-3.58 (14H, m, H5b, H5c', H6c, H6c', H4d, H4d', H5d, H5d', H3e, H3e', H6e, H6e', H4f, H4f'), 3.58-3.70 (17H, m, H3a, H4a, H6a, H6'a, H3b, H4b, H5c, H2d, H2d', H3d, H3d', H6d, H6d', H5f, H5f', H6f, H6f'), 3.70-3.88 (18H, m, H6b, H6'b, H3c, H3c', H6'c, H6'c', H6'd, H6'd', H4e, H4e', H5e, H5e', H6'e, H6'e', H8f, H8f', H9'f, H9'f'), 3.98-4.12 (4H, m, H2a, H2b, H2c, H2c'), 4.30 (2H, br s, H1e, H1e'), 4.46-4.48 (2H, m, H1d, H1d'), 4.60 (1H, s, H1b), 4.80 (1H, s, H1c'), 4.98 (1H, s, H1c), 5.94 (1H, d, *J* 7.4 Hz, H1); δ_C (125.8 MHz, D_2O) 12.9 (C=N($\underline{C}H_3$)) 22.0, 22.4 (CO*C*H_3 x 4), 40.0 (C3f, C3f'), 51.8 (C5f, C5f'), 54.6 (C2d, C2d'), 60.2 (C6d, C6d'), 61.6 (C6a, C6c, C6c'), 62.6 (C6e, C6e'), 63.3 (C9f, C9f'), 65.1 (C2a), 65.8 (C6b), 67.3, 68.2, 68.4 (C4c, C4c', C4e, C4e', C4f, Cf4', C7f, C7f'), 69.1 (C3a), 69.4 (C3c, C3c'), 69.6 (C8f, C8f'), 70.2 (C2b), 70.7 (C2e, C2e'), 70.9 (C5a), 71.7 (C5e, C5e'), 72.0, 72.1 (C3d, C3d'), 72.4, 72.5 (C4b, C5e, C5e'), 73.7 (C6f, C6f'), 74.2 (C5b), 74.4 (C5d, C5d'), 76.0, 76.4 (C2c, C2c'), 77.9 (C4a), 80.5 (C3b), 80.7 (C4d, C4d'), 96.5 (C1c'), 99.3, 99.5 (C1d, C1d'), 100.0 (C1a), 100.1 (C1c, C2f, C2f'), 101.3 (C1b), 103.5 (C1e, C1e'), 168.6 (C1f, C1f'), 173.5, 174.7, 174.9, 175.0 (4 x *C*OCH_3); LRMS (ESI^+): *m/z* 2002 (MH^+, 2), 1012 ($M{\cdot}2H^+$, 50%).

Decasaccharide propargyl amide lactol ***5a***

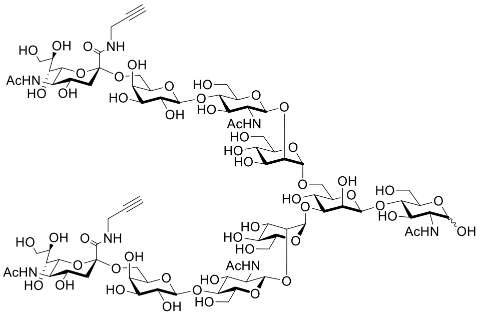

Lactol **4** (2.0 mg, 0.955 μmol) was dissolved in water (48 μL). An aqueous solution of propargly amine hydrochloride salt (1.75 mg, 0.4 M solution) was added, followed by 4-(4,6-dimethoxy-1,3,5-triazin-2-yl)-4-methylmorpholinium chloride (4.66 mg, 85% purity from Sigma, 17 μmol) and the reaction warmed to 50 ºC. After 16 h t.l.c. analysis (IPA:1 M ammonium acetate, 3:2) indicated consumption of the diacid starting material (R_f 0.60) and formation of a major product (R_f 0.67). The reaction mixture was cooled and the reaction mixture placed in a centrifugal filter unit (0.22 μm membrane) and subjected to centrifugation (13,000 g, 1 min.) to remove the white precipitate. The cake was twice washed with water (20 μL) and centrifuged. The reaction mixture was subjected to PD MiniTrap G25 purification, eluting with water. Fractions containing glycan product were collected and lyophilised to afford **5a** as a white foam, 2.1 mg, quant.; $[\alpha]_D^{20}$ = - 2.8° (c = 0.47, H_2O); IR

(ν_{max}, single crystal) 3292, 2921, 2853, 1738, 1643, 1547; ^{1}H NMR (700MHz, D_2O) 1.84 (2H, dd, $J_{3ax\text{-}3eq}$ 12.6 Hz, $J_{3\text{-}4}$ 12.6 Hz, $H3f_{ax}$, $H3f'_{ax}$), 2.03 (6H, s, $COCH_3$ x 2), 2.058, 2.061, 2.062 (9H, s x 3, $COCH_3$ x 3), 2.64 (2H, t, $J_{1\text{-}3}$ 2.8 Hz, 2 x C≡CH), 2.69 (2H, dd, $J_{3eq\text{-}3ax}$ 12.6 Hz, $J_{3eq\text{-}4}$ 4.9 Hz, $H3f_{eq}$, $H3f'_{eq}$), 3.47-3.55 (4H, m, H4c, H4c', H2e, H2e'), 3.55-3.69 (16.4H, m, β-H5a, H5b, H5c', H6c, H6c', H4d, H4d', H5d, H5d', H3e, H3e', H6e, H6e', H7f, H7f', H9f, H9f'), 3.69-3.82 (15.8H, m, α-H4a, α-H6a, α-H6'a, β-H2a, β-H3a, β-H4a, β-H6a, β-H6'a, H3b, H4b, H6b, H5c, H2d, H2d', H3d, H3d', H4f, H4f', H6f, H6f'), 3.82-3.99 (22.8H, m, α-H2a, α-H3a, α-H5a, H6'b, H3c, H3c', H6'c, H6'c', H6d, H6d', H6'd, H6'd', H4e, H4e', H5e, H5e', H6'e, H6'e', H5f, H5f', H8f, H8f', H9'f, H9'f'), 3.99 (2H, dd, $J_{1\text{-}1'}$ 18.2 Hz, $J_{1\text{-}3}$ 2.8 Hz, 2 x CH_2C≡CH), 4.06 (2H, dd, $J_{1'\text{-}1}$ 18.2 Hz, $J_{1'\text{-}3}$ 2.8 Hz, 2 x CH_2C≡CH), 4.12 (1H, d, $J_{2\text{-}3}$ 2.1 Hz, H2c'), 4.19 (1H, d, $J_{2\text{-}3}$ 2.8 Hz, H2c), 4.24-4.27 (1H, m, H2b), 4.44 (2H, d, $J_{1\text{-}2}$ 7.7 Hz, H1e, H1e'), 4.59 (2H, d, $J_{1\text{-}2}$ 7.7 Hz, H1d, H1d'), 4.71 (0.4H, d, $J_{1\text{-}2}$ 7.7 Hz, β-H1a), 4.77 (1H, s, H1b), 4.94 (1H, s, H1c'), 5.13 (1H, s, H1c), 5.21 (0.6H, d, $J_{1\text{-}2}$ 2.8 Hz, α-H1a); ^{13}C NMR (176 MHz, D_2O) 22.6, 22.7, 23.0, 23.1 ($COCH_3$ x 5), 29.5 (2 x CH_2C≡CH), 38.6, 38.6 (C3f, C3f'), 52.3 (C5f, C5f'), 54.2 (α-C2a), 55.2, 55.3 (C2d, C2d'), 56.7 (β-C2a), 60.7 (α-C6a), 60.8, 60.8 (β-C6a, C6d, C6d'), 62.3 (C6c'), 62.4 (C6c), 63.4, 63.4 (C6e, C6e'), 63.7 (C9f, C9f'), 66.4 (C4b), 66.6 (C6b), 67.6, 67.6 (C4f, C4f'), 68.0 (C4c), 68.0 (C4c'), 68.4 (C7f, C7f'), 69.0 (C4e, C4e'), 69.8 (α-C3a), 70.0, 70.1 (C3c,C3c'), 70.6 (α-C5a), 70.6 (C2b), 71.3, 71.3 (C2e, C2e'), 71.8 (C8f, C8f'), 72.7, 72.8 (C3d, C3d'), 73.0 (2 x CH_2C≡*C*H), 73.0 (β-C3a), 73.1 (C3e, C3e'), 73.5 (C5c'), 74.1 (C6f, C6f'), 74.2 (C5c), 74.2 (C5e, C5e'), 75.0, 75.0 (C5b, C5d, C5d'), 75.1 (β-C5a), 76.9 (C2c'), 77.1 (C2c), 79.4, 79.5 (2 x CH_2*C*≡CH), 80.5 (β-C4a), 80.9 (α-C4a), 81.1 (C3b), 81.3, 81.4 (C4d, C4d'), 91.2 (α-C1a), 95.6 (β-C1a), 97.7 (C1c'), 99.9, 100.0, 100.0 (C1d, C1d', C2f, C2f'), 100.2 (C1c), 101.1 (C1b), 104.3 (C1e, C1e'), 169.8, 169.9 (C1f, C1f'), 175.2, 175.3, 175.4, 175.7 (5 x $COCH_3$); coupled HSQC: *J*(α-H1a-α-C1a) 172 Hz, *J*(β-H1a-β-C1a) 162 Hz, *J*(H1b-C1b) 161 Hz, *J*(H1c-C1c) 171 Hz, *J*(H1c'-C1c') 170 Hz, *J*(H1d-C1d) 161 Hz, *J*(H1e-C1e) 161 Hz; LRMS (ESI^+): *m/z* 1070.1 (M M·$2Na^+$, 100%); HRMS (ESI^+) calculated for $C_{82}H_{131}N_7Na_2O_{55}$ 1069.8728; observed 1069.8763.

Decasaccharide propargyl amide oxazoline ***6a***

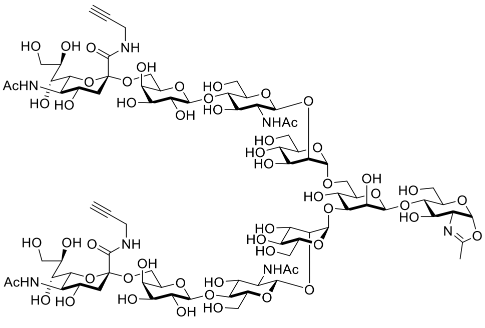

Lactol **5a** (8 mg, 3.82 mmol) was dissolved in water (150 µL). CDMBI (8.3 mg, 38.2 mmol) was added, followed by potassium phosphate tribasic (153 µL of a 0.75 M solution). The mixture was vortexed and the shaken at 4 °C for 18 hours. After this time ESI-MS analysis

indicated complete consumption of lactol and formation of oxazoline product. The mixture was filtered through a centrifugal spin filter (0.2 μm membrane) and applied to a PD Miditrap G25 column. The product was eluted with 0.01% ammonia solution; pure fractions containing glycan were collected and lyophilised to afford **6a** as a white fluffy solid, 6.8 mg (85%); ν_{max} (single crystal) 3205, 2439, 1655, 1557 cm^{-1}; δ_H (500 MHz, D_2O) 1.77 (2H, t, *J* 12.4 Hz, H3f$_{ax}$, H3f'$_{ax}$), 1.96, 1.99, 1.99, 2.00, 2.00 (15H, 3 x s, 5 x CH_3), 2.57 (2H, t, *J* 2.3 Hz, 2 x C≡CH), 2.63 (2H, dd, $J_{3eq\text{-}4}$ 4.5 Hz, $J_{3eq\text{-}3ax}$ 12.3 Hz, H3f$_{eq}$, H3f'$_{eq}$), 3.34-3.90 (51H, m, H2a, H3a, H4a, H5a, H6a, H6'a, H3b, H4b, H5b, H6b, H6'b, H3c, H3c', H4c, H4c', H5c, H5c', H6c, H6c', H6'c, H6'c', H2d, H2d', H3d, H3d', H4d, H4d', H5d, H5d', H6d, H6d', H6'd, H6'd', H2e, H2e', H3e, H3e', H4e, H4e', H5e, H5e', H6e, H6e', H6'e, H6'e', H4f, H4f', H5f, H5f', H6f, H6f', H7f, H7f', H8f, H8f', H9f, H9f', H9'f, H9'f'), 3.92 (2H, dd, *J* 2.3 Hz, 17.5 Hz, 2 x C*H*$_2$C≡CH), 4.00 (2H, dd, *J* 2.3 Hz, 17.6 Hz, 2 x C*H*$_2$C≡CH), 4.09 (1H, d, $J_{2\text{-}3}$ 3.3 Hz, H2c'), 4.12 (1H, br s, H2c), 4.31 (1H, d, $J_{1,2}$ 2.0 Hz, H2b), 4.37 (2H, d, $J_{1\text{-}2}$ 7.9 Hz, H1e, H1e'), 4.52-4.56 (2H, m, H1d, H1d'), 4.69 (1H, s, H1b), 4.89 (1H, s, H1c'), 5.06 (1H, s, H1c), 6.02 (1H, d, *J* 7.2 Hz, H1a); ^{13}C NMR (125.8 MHz, D_2O) 13.6 (C=N($\underline{C}H_3$)) 22.7, 23.0 (CO*C*H$_3$ x 4), 29.4 (2 x *C*H$_2$C≡CH), 38.6 (C3f, C3f'), 52.3 (C5f, C5f'), 55.2, 55.2 (C2d, C2d'), 60.7, 60.8 (C6a, C6d, C6d'), 62.2 (C6c'), 62.3 (C6c), 63.3 (C6e, C6e'), 63.6 (C9f, C9f'), 65.8 (C2a), 66.3 (C4b), 66.4 (C6b), 67.6 (C4f, C4f'), 67.9, 67.9 (C4c, C4c'), 68.3 (C7f, C7f'), 68.9, 68.9 (C4e, C4e'), 70.0, 70.1 (C3c, C3c'), 70.9 (C2b), 71.2, 71.5 (C2e, C2e'), 71.7 (C8f, C8f'), 72.7 (C3d, C3d'), 73.0 (2 x CH$_2$C≡*C*H), 73.1 (C3e, C3e'), 73.4 (β-C3a), 73.5 (C5c'), 74.1 (C6f, C6f'), 74.1 (C5c), 74.2 (C5e, C5e'), 75.0 (C5b, C5d, C5d'), 75.5 (C5a), 77.0 (C2c', C2c), 78.9 (2 x CH$_2$*C*≡CH), 81.0 (C4a), 81.2 (C3b), 81.4, 81.4 (C4d, C4d'), 97.2 (C1c'), 99.9, 99.9, 100.0, 100.1 (C1a, C1d, C1d', C2f, C2f'), 100.6 (C1c), 102.0 (C1b), 104.2 (C1e, C1e'), 169.8 (C1f, C1f'), 175.3, 175.3, 175.6, 175.67 (4 x *C*OCH$_3$); LRMS (ESI$^+$): *m/z* 2077 (MH$^+$, 3), 1061 (M·2Na$^+$, 20), 1048 (M·Na$^+$·H$^+$, 28), 1039 (M·2H$^+$, 100%); HRMS (ESI$^+$) ion series calculated for $C_{82}H_{129}N_7O_{54}Na$ 2098.74665, 2099.75002, 2100.75337, 2101.75671, 2103.76100; observed: 2098.75008, 2099.75406, 2100.75743.

*Decasaccharide azide lactol **5b***

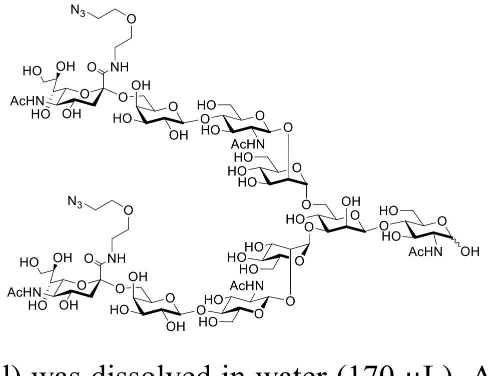

Lactol 4 (5.1 mg, 2.52 μmol) was dissolved in water (170 μL). An aqueous solution of 2-(2-azidoethoxy)ethan-1-amine trifluoroacetate (14.2 mg, 0.4 M solution) was added, followed by 4-(4,6-dimethoxy-1,3,5-triazin-2-yl)-4-methylmorpholinium chloride (16.4 mg, 85% purity from Sigma, 59 μmol) and the reaction warmed to 37 ºC. After 16 h t.l.c. analysis

(IPA:1 M ammonium acetate, 3:2) indicated consumption of the diacid starting material (R_f 0.60) and formation of a major product (R_f 0.67). The reaction mixture was cooled and the reaction mixture placed in a centrifugal filter unit (0.22 μm membrane) and subjected to centrifugation (13,000 g, 1 min.) to remove the white precipitate. The cake was twice washed with water (30 μL) and centrifuged. The reaction mixture was subjected to PD MiniTrap G10 purification, eluting with water. Fractions containing glycan product were collected and lyophilised to afford the target compound as a white solid, 4.1 mg, 72%. Analysis indicated that the amidation reaction had not proceeded to completion and so this product was re-subjected to the reaction conditions. After this second round of reaction and purification the target 5b was obtained as a white foam, 3.4 mg, 66%; ν_{max} (single crystal) 3297, 2108, 1647, 1549 cm^{-1}; δ_H (500 MHz, D_2O) 1.76 (2H, t, *J* 12.3 Hz, $H3f_{ax}$, $H3f'_{ax}$), 1.90, 1.95, 1.97 (15H, 3 x s, 5 x CH_3), 2.62 (2H, dd, J_{3eq-4} 4.3 Hz, $J_{3eq-3ax}$ 12.7 Hz, $H3f_{eq}$, $H3f'_{eq}$), 3.39-3.47 (12H, m, H4c, H4c', H2e, H2e', 2 x CH_2NH, 2 x CH_2N_3), 3.48-3.63 (27H, m, H4a, H5a(β), H5b, H5c', H6c, H6c', H4d, H4d', H5d, H5d', H3e, H3e', H6e, H6e', H4f, H4f', H7f, H7f', H9f, H9f', 2 x CH_2OCH_2), 3.61-3.75 (10H, m, H3a, H6a, H3b, H5c, H2d, H2d', H3d, H3d', H6f, H6f'), 3.76-3.93 (25H, m, H2a, H6'a, H4b, H6b, H6'b, H3c, H3c', H6'c, H6'c', H6d, H6d', H6'd, H6'd', H4e, H4e', H5e, H5e', H6'e, H6'e', H5f, H5f', H8f, H8f', H9'f, H9'f'), 4.03 (1H, br s, H2c'), 4.11 (1H, br s, H2c), 4.18 (1H, br s, H2b), 4.36 (2H, d, J_{1-2} 7.8 Hz, H1e, H1e'), 4.51 (2H, d, J_{1-2} 6.3 Hz, H1d, H1d'), 4.53 (0.3H, d, H1a(β)), 4.70 (1H, d, H1b), 4.86 (1H, s, H1c'), 5.05 (1H, s, H1c), 5.13 (0.7H, d, J_{1-2} 2.7 Hz, H1a(α)); δ_C (125.8 MHz, D_2O) 22.9, 23.0, 23.3 (5 x CH_3), 39.0 (C-3f, C-3f'), 39.8 ($NHCH_2$), 51.3 (CH_2N_3), 52.6 (C-5f, C-5f'), 54.5 (C-2a(α)), 55.5 (C-2d, C-2d'), 61.0, 61.0 (C-6a, C-6d, C-6d'), 62.6, 62.6 (C-6c, C-6c'), 63.5, 63.8 (C-6e, C-6e', C-9f, C-9f'), 66.6 (C-6b), 67.9 (C-4f, C-4f'), 68.3 (C-4c, C-4c'), 68.7 (C-7f, C-7f'), 69.2 (C-4e, C-4e'), 69.4, 69.7 (4 x OCH_2), 70.1 (C-3a(α)), 70.3 (C-5a(α)), 70.5 (C-3c, C-3c'), 71.2 (C-2b), 71.6 (C-2e, C-2e'), 72.0 (C-8f, C-8f'), 73.0 (C-3d), 73.1 (C-3a), 73.4 (C-3e, C-3e'), 73.8 (C-5c'), 74.4 (C-5c, C-5e, C-5e', C-6f, C-6f'), 75.3 (C-5d, C-5d, C-5d'), 77.1, 77.3 (C-2c, C-2c'), 81.1 (C-4c), 81.4 (C-3c), 81.6, 81.7 (C-4d, C-4d'), 91.4 (C-1a(α)), 95.9 (C-1a(β)), 97.9 (C1c'), 100.2, 100.3 (C1d, C1d'), 100.5 (C1c), 101.4 (C1b), 104.6 (C1e, C1e'), 170.0, 175.6, 176.0 (5 x C=O); LRMS (ESI^+): *m/z* 2244 ($M{\cdot}H^+$, 2), 2268 ($M{\cdot}Na^+$, 12), 1145 ($M{\cdot}2Na^+$, 10), 1134 ($M{\cdot}Na^+{\cdot}H^+$, 60), 1123 ($M{\cdot}2H^+$, 58%); HRMS (ESI^+) ion series calculated for $C_{84}H_{141}N_{13}O_{57}Na$ 2266.8426, 2267.8459, 2268.8492, 2269.8526, 2270.8536, 2271.8570; observed: 2266.8538, 2267.8515, 2268.8530, 2269.8561, 2270.8354.

*Decasaccharide azide oxazoline **6b***

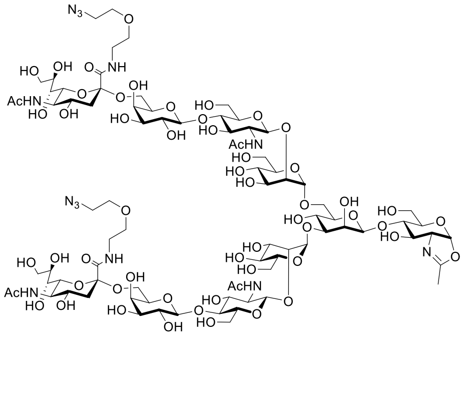

Lactol 5b (1.4 mg, 624 nmol) was dissolved in water (25 μL). CDMBI (1.36 mg, 6.24 μmol) was added, followed by potassium phosphate tribasic (25 μL of a 0.75 M solution). The mixture was vortexed and the shaken at 4 °C for 4 hours. After this time ESI-MS analysis indicated complete consumption of lactol and formation of oxazoline product. The mixture was filtered through a centrifugal spin filter (0.2 μm membrane) and applied to a PD Minitrap G25 column. The product was eluted with 0.01% ammonia solution; pure fractions containing glycan were collected and lyophilised to afford 6b as a white fluffy solid, 1.06 mg (76%); $[\alpha]_D^{25}$ = + 0.45° (c = 0.22, H_2O); ν_{max} (single crystal) 3300, 2115, 1648, 1545 cm^{-1}; δ_H (500 MHz, D_2O) 1.76 (2H, t, *J* 12.3 Hz, H3f_{ax}, H3f'$_{ax}$), 1.96, 1.98, 1.99 (15H, 3 x s, 5 x CH_3), 2.63 (2H, dd, $J_{3eq\text{-}4}$ 4.3 Hz, $J_{3eq\text{-}3ax}$ 12.8 Hz, H3f_{eq}, H3f'$_{eq}$), 3.39-3.91 (51H, m, H2a, H3a, H4a, H5a, H6a, H6'a, H3b, H4b, H5b, H6b, H6'b, H3c, H3c', H4c, H4c', H5c, H5c', H6c, H6c', H6'c, H6'c', H2d, H2d', H3d, H3d', H4d, H4d', H5d, H5d', H6d, H6d', H6'd, H6'd', H2e, H2e', H3e, H3e', H4e, H4e', H5e, H5e', H6e, H6e', H6'e, H6'e', H4f, H4f', H5f, H5f', H6f, H6f', H7f, H7f', H8f, H8f', H9f, H9f', H9'f, H9'f', 2 x C*H*$_2$OC*H*$_2$), 4.08 (1H, at, *J* 2.7 Hz, H2c'), 4.12 (1H, br s, H2c), 4.31 (1H, d, $J_{1,2}$ 2.0 Hz, H2b), 4.37 (2H, d, $J_{1\text{-}2}$ 7.7 Hz, H1e, H1e'), 4.51 (2H, d, $J_{1\text{-}2}$ 7.0 Hz, H1d, H1d'), 4.67 (1H, s, H1b), 4.89 (1H, s, H1c'), 5.04 (1H, s, H1c), 6.01 (1H, d, $J_{1\text{-}2}$ 7.3 Hz, H1a); ^{13}C NMR (125.8 MHz, D_2O, selected resonances only) 12.9 (C=N($\underline{C}H_3$)) 22.0, 22.4 (CO*C*H_3 x 4), 38.1, 38.6 (C3f, C3f'), 50.3, 51.7 (C5f, C5f'), 54.6, 56.9 (C2d, C2d'), 60.1, 61.6, 62.5, 62.7, 62.9 (C6a, C6c, C6c', C6d, C6d', C6e, C6e', C9f, C9f'), 65.2 (C2a), 65.9 (C4b), 80.7 (C4d, C4d'), 96.5 (C1c'), 99.3, 99.4, 99.5 (C1a, C1d, C1d', C2f, C2f'), 101.4 (C1c), 101.9 (C1b), 103.6 (C1e, C1e'), 168.5, 169.1 (C1f, C1f'), 174.5, 175.0 (4 x *C*OCH_3); LRMS (ESI^+): *m/z* 2228 (M·H^+, 3), 1134 (M·2Na^+, 54), 1123 (M·Na^+·H^+, 61), 1114 (M·2H^+, 100%); HRMS (ESI^+) ion series calculated for $C_{84}H_{139}N_{13}O_{56}Na_2$ 1135.91065, 1136.41234, 1136.91401, 1137.41566, 1137.91617, 1138.41782; observed: 1135.91434, 1136.41585, 1136.91712, 1137.41824, 1137.91969.

Decasaccharide lactol bearing SSMe groups ***5c***

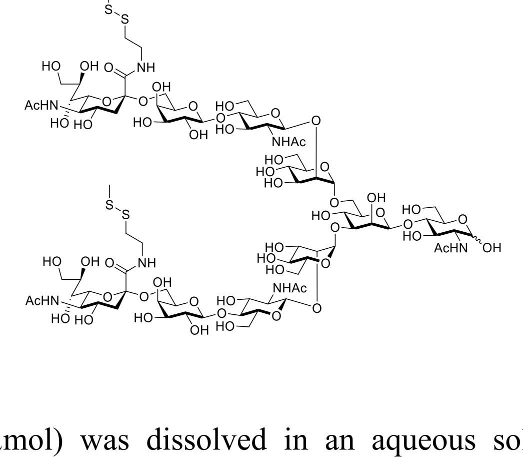

Lactol 4 (2.0 mg, 0.990 μmol) was dissolved in an aqueous solution of 2-(pyridine-2-yldisulfanyl)ethan-1-amine hydrochloride (3.2 mg, 0.2 M solution). 4-(4,6-Dimethoxy-1,3,5-triazin-2-yl)-4-methylmorpholinium chloride (4.8 mg, 17.5 μmol) was added and the reaction warmed to 50 ºC. After 16 h t.l.c. analysis (IPA:1 M ammonium acetate, 3:2) indicated

complete consumption of the diacid starting material (R_f 0.60) and formation of a major product (R_f 0.67). The reaction mixture was cooled and the reaction mixture placed in a centrifugal filter unit (0.22 μm membrane) and subjected to centrifugation (13,000 g, 1 min.) to remove the white precipitate. The cake was twice washed with water (30 μL) and centrifuged. The reaction mixture was subjected to PD MiniTrap G10 purification, eluting with water. Fractions containing glycan product were collected and lyophilised to afford 5c as a white solid, 1.4 mg, 63%; ν_{max} (single crystal) 3283, 1645, 1549 cm^{-1}; δ_H (500 MHz, D_2O) 1.75 (2H, t, *J* 12.3 Hz, H3f_{ax}, H3f'$_{ax}$), 1.95, 1.98 (15H, 2 x s, 5 x CH_3), 2.35 (6H, s, 2 x SCH_3), 2.62 (2H, dd, $J_{3eq\text{-}4}$ 4.3 Hz, $J_{3eq\text{-}3ax}$ 12.8 Hz, H3f_{eq}, H3f'$_{eq}$), 2.84 (4H, t, *J* 5.7 Hz, 2 x CH_2S), 3.40-3.90 (67H, m, H2a, H3a, H4a, H5a, H6a, H6'a, H3b, H4b, H5b, H6b, H6'b, H3c, H3c', H4c, H4c', H5c, H5c', H6c, H6c', H6'c, H6'c', H2d, H2d', H3d, H3d', H4d, H4d', H5d, H5d', H6d, H6d', H6'd, H6'd', H2e, H2e', H3e, H3e', H4e, H4e', H5e, H5e', H6e, H6e', H6'e, H6'e', H4f, H4f', H5f, H5f', H6f, H6f', H7f, H7f', H8f, H8f', H9f, H9f', H9'f, H9'f', 2 x C*H*$_2$NH, 2 x CH_2S), 4.03 (1H, br s, H2c'), 4.11 (1H, br s, H2c), 4.18 (1H, br s, H2b), 4.36 (2H, d, $J_{1\text{-}2}$ 7.9 Hz, H1e, H1e'), 4.51 (2H, d, $J_{1\text{-}2}$ 7.5 Hz, H1d, H1d'), 4.36 (2H, d, $J_{1\text{-}2}$ 7.9 Hz, H1e, H1e'), 4.51 (2H, d, $J_{1\text{-}2}$ 7.5 Hz, H1d, H1d'), H1b obscured by solvent peak, 4.86 (1H, s, H1c'), 5.12 (0.7H, d, $J_{1\text{-}2}$ 3.2 Hz, H1a(α)); δ_C (125.8 MHz, D_2O) 21.9, 22.0, 22.4 (7 x CH_3), 35.4 (2 x CH_2S), 37.6 (2 x $\underline{C}H_2CH_2S$), 38.2 (C3f, C3f'), 51.7 (C-5f, C-5f'), 53.5 (C-2a(α)), 54.5 (C-2d, C-2d'), 60.0, 60.1 (C-6a, C-6d, C-6d'), 61.6, 61.7 (C-6c, C-6c'), 62.7, 62.9 (C-6e, C-6e', C-9f, C-9f'), 67.6 (C-6b), 66.9 (C-4f, C-4f'), 67.2, 67.3 (C-4c, C-4c'), 67.7 (C-7f, C-7f'), 68.3 (C-4e, C-4e'), 69.1 (C-3a(α)), 69.4, 69.4 (C-5a(α)), 69.9 (C-3c, C-3c'), 70.2 (C-2b), 70.6 (C-2e, C-2e'), 71.1 (C-8f, C-8f'), 72.0 (C-3d), 72.1 (C-3a), 72.4 (C-3e, C-3e'), 72.8 (C-5c'), 73.5, 73.5, 73.6 (C-5c, C-5e, C-5e', C-6f, C-6f'), 74.3 (C-5d, C-5d, C-5d'), 76.2, 76.3 (C-2c, C-2c'), 80.1 (C-4c), 80.5 (C-3c), 80.7, 80.7 (C-4d, C-4d'), 90.5 (C1a(α)), 94.9 (C1a(β)), 96.9 (C1c'), 99.3, 99.4 (C1d, C1d'), 99.5 (C1c), 100.1, 100.1 (2 x s, C2f, C2f'), 100.4 (C1b), 103.6 (C1e, C1e'), 169.2 (C1f, C1f'), 174.6, 175.1 (5 x C=O); LRMS (ESI^+): *m/z* 1116 (M·$2Na^+$, 100%); HRMS (ESI^+) ion series calculated for $C_{82}H_{140}N_7NaO_{55}S_4$ 1126.85714, 1127.35882, 1127.86050, 1128.35673, 1128.85840, 1129.36005 observed: 1126.85217, 1127.35315, 1127.85406, 1128.35318, 1128.85495, 1129.35529.

Decasaccharide oxazoline bearing SH groups 6c

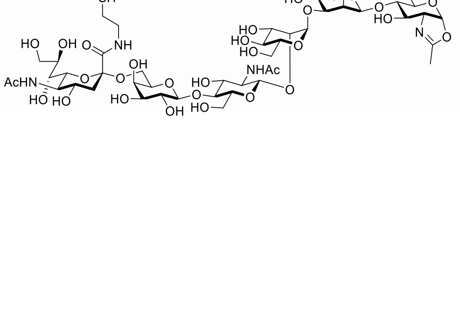

Lactol **4** (1.4 mg, 583 nmol) was dissolved in water (25 μL). CDMBI (1.4 mg, 6.27 μmol) was added, followed by potassium phosphate tribasic (25 μL of a 0.75 M solution). The mixture was vortexed and the shaken at 4 °C for 4 hours. After this time ESI-MS analysis indicated complete consumption of lactol and formation of oxazoline product. A solution of DTT (73 μL of an 80 mM aqueous solution) was added and the mixture shaken at RT for a further 30 mins. ESI-MS analysis indicated complete reduction of the disulfide bonds. The mixture was filtered through a centrifugal spin filter (0.2 μm membrane) and applied to a PD Minitrap G25 column. The product was eluted with 0.01% ammonia solution; pure fractions containing glycan were collected and lyophilised to afford **6c** as a white fluffy solid, 1.2 mg (90%); $[\alpha]_D^{25}$ = + 1.3° (c = 0.06, H_2O); ν_{max} (single crystal) 3208, 1647, 1562, 1449 cm^{-1}; δ_H (700 MHz, D_2O) 1.70 (2H, t, *J* 12.4 Hz, $H3f_{ax}$, $H3f'_{ax}$), 1.89, 1.91, 1.91, 1.92, 1.92 (15H, 5 x s, 5 x CH_3), 2.52-2.62 (6H, m, $H3f_{eq}$, $H3f'_{eq}$, 2 x NCH_2), 3.27-3.40 (9H, m, H5a, H4c, H4c', H2e, H2e', 2 x SCH_2), 3.42-3.60 (16H, m, H5b, H5c', H6c, H6c', H4d, H4d', H5d, H5d', H3e, H3e', H6e, H6e', H7f, H7f', H9f, H9f'), 3.61-3.87 (36H, m, H4a, H6a, H6'a, H3b, H4b, H6b, H6'b, H3c, H3c', H5c, H6'c, H6'c', H2d, H2d', H3d, H3d', H6d, H6d', H6'd, H6'd', H4e, H4e', H5e, H5e', H6'e, H6'e', H4f, H4f', H5f, H5f', H6f, H6f', H8f, H8f', H9'f, H9'f'), 3.99 (1H, d, *J* 2.7 Hz, H2c'), 4.04 (2H, m, H2a, H2c), 4.11 (1H, d, H2b), 4.25 (1H, at, *J* 3.3 Hz, H3a), 4.29 (2H, d, J_{1-2} 7.9 Hz, H1e, H1e'), 4.45 (2H, d, J_{1-2} 6.2 Hz, H1d, H1d'), 4.63 (1H, d, H1b), 4.80 (1H, s, H1c'), 4.98 (1H, s, H1c), 5.94 (1H, d, J_{1-2} 7.2 Hz, H1a); δ_C (125.8 MHz, D_2O) 12.9 (C=N(<u>C</u>H_3)), 22.0, 22.4, 23.0 (5 x CH_3), 38.0 (C3f, C3f'), 42.1 (2 x <u>C</u>H_2CH_2S), 51.8 (C5f, C5f'), 54.5 (C2d, C2d'), 60.1 (C6a, C6d, C6d'), 61.6, 61.7 (C6c, C6c'), 62.7 (C6e, C6e'), 63.0 (C9f, C9f'), 65.2 (C2a), 65.8 (C6b), 66.9 (C4b, C4f, C4f'), 67.3, 67.4 (C4c, C4c'), 67.7 (C7f, C7f'), 68.3 (C4e, C4e'), 69.2 (C3a), 69.4, 69.5 (C3c, C3c'), 70.2 (C2b), 70.6 (C2e, C2e'), 70.9 (C5a), 71.0 (C8f, C8f'), 72.0, 72.1 (C3d, C3d'), 72.4 (C3e, C3e'), 72.9 (C5c'), 73.5, 73.6 (C5c, C5e, C5e', C6f, C6f'), 74.3 (C5b), 74.3 (C5d, C5d'), 76.2 (C2c'), 76.4 (C2c), 77.9 (C4a), 80.5 (C3b), 80.7 (C4d, C4d'), 97.0 (C1c'), 99.3 (C1c), 99.4 (C1d, C1d'), 100.0 (C1a), 100.4 (C1b), 103.6 (C1e, C1e'), 169.2 (C1f, C1f'), 174.6, 175.0 (4 x *C*OCH_3); LRMS (ESI^+): *m/z* 2122 ($M{\cdot}H^+$, 11), 1082 ($M{\cdot}2Na^+$, 32), 1072 ($M{\cdot}Na^+{\cdot}H^+$, 66), 1061 ($M{\cdot}2H^+$, 100%); HRMS (ESI^+) ion series calculated for $C_{80}H_{133}N_7O_{54}Na_2S_2$ 1082.85511, 1083.35679, 1083.85847, 1084.36013, 1084.86061; observed: 1082.85349, 1083.35500, 1083.85610, 1084.35610, 1084.85775, 1085.35378.

Decasaccharide lactol bearing SSPy groups 5d

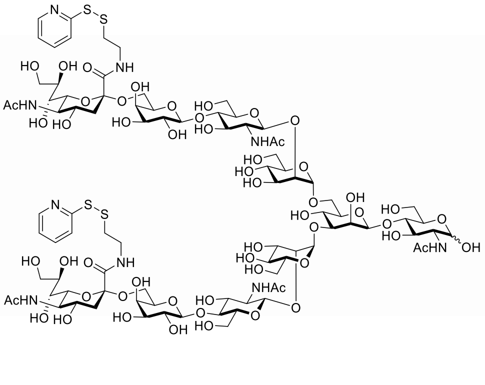

Lactol 4 (2.3 mg, 1.14 μmol) was dissolved in an aqueous solution of 2-(pyridine-2-yldisulfanyl)ethan-1-amine hydrochloride (5.07 mg, 0.4 M solution). 4-(4,6-Dimethoxy-1,3,5-triazin-2-yl)-4-methylmorpholinium chloride (4.72 mg, 17.13 μmol) was added and the reaction warmed to 50 ºC. After 16 h t.l.c. analysis (IPA:1 M ammonium acetate, 3:2) indicated complete consumption of the diacid starting material (R_f 0.60) and formation of a major product (R_f 0.67). The reaction mixture was cooled and the reaction mixture placed in a centrifugal filter unit (0.22 μm membrane) and subjected to centrifugation (13,000 g, 1 min.) to remove the white precipitate. The cake was twice washed with water (30 μL) and centrifuged. The reaction mixture was subjected to PD MiniTrap G10 purification, eluting with water. Fractions containing glycan product were collected and lyophilised to afford 5d as a white solid, 1.8 mg, 67%; ν_{max} (single crystal) 3331, 2935, 1621, 1548 cm^{-1}; δ_H (500 MHz, D_2O, diagnostic resonances) 1.76 (2H, t, *J* 12.2 Hz, H3f_{ax}, H3f'$_{ax}$), 1.93, 1.94, 1.95, 1.97 (15H, 4 x s, 5 x CH_3), 2.59 (2H, dd, $J_{3eq\text{-}4}$ 4.9 Hz, $J_{3eq\text{-}3ax}$ 12.6 Hz, H3f_{eq}, H3f'$_{eq}$), 2.91 (4H, t, *J* 6.1 Hz, 2 x CH_2S), 4.34 (2H, d, $J_{1\text{-}2}$ 7.8 Hz, H1e, H1e'), 4.50 (2H, d, $J_{1\text{-}2}$ 7.0 Hz, H1d, H1d'), 4.53 (0.3H, d, H1a(β)), 4.70 (1H, d, H1b), 4.85 (1H, s, H1c'), 5.04 (1H, s, H1c), 5.12 (0.7H, d, $J_{1\text{-}2}$ 3.2 Hz, H1a(α)), 7.22-7.25 (2H, m, 2 x ArH), 7.77 (4H, d, *J* 3.8 Hz, 4 x ArH), 8.33 (2H, d, *J* 4.9 Hz, 2 x ArH); δ_C (125.8 MHz, D_2O, from HSQC, diagnostic resonances) 21.9, 22.0, 22.3, 22.4, 22.4 (5 x CH_3), 35.4 (CH_2S), 37.1 (CH_2CH_2S), 40.0 (C3f, C3f'), 90.4 (C1a(α)), 94.9 (C1a(β)), 96.9 (C1c'), 99.2, 99.3 (C1d, C1d'), 99.5 (C1c), 100.1, 100.1 (C2f, C2f'), 100.4 (C1b), 103.5 (C1e, C1e'), 121.5 (Ar-C), 121.8 (Ar-C), 138.6 (Ar-C), 149.3 (Ar-C); LRMS (ESI^+): *m/z* 1198 ($M\cdot2Na^+$, 59), 1179 ($M\cdot2H^+$, 100%); HRMS (ESI^+) ion series calculated for $C_{90}H_{141}N_9Na_2O_{55}S_4$ 1200.85901, 1201.36048, 1201.86095, 1202.36162, 1202.86191, 1203.36241, 1203.86251, observed: 1200.86410, 1201.36596, 1201.86748, 1202.36918, 1202.87064, 1203.37034, 1203.87243.

Decasaccharide oxazoline bearing SSPy groups ***6d***

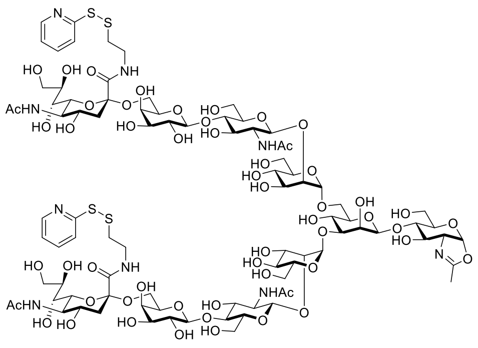

Lactol **5d** (1.8 mg, 764 nmol) was dissolved in water (30.5 μL). CDMBI (1.66 mg, 7.64 μmol) was added, followed by potassium phosphate tribasic (30.5 μL of a 0.75 M solution). The mixture was vortexed and the shaken at 4 °C for 4.5 hours. After this time ESI-MS analysis indicated complete consumption of lactol and formation of oxazoline product. The mixture was filtered through a centrifugal spin filter (0.2 μm membrane) and applied to a PD Miditrap G25 column. The product was eluted with 0.01% ammonia solution; pure fractions containing glycan were collected and lyophilised to afford **6d** as a white fluffy solid, 1.5 mg

(84%), $[\alpha]_D^{25}$ = -1.7° (c = 0.06, H_2O); ν_{max} (single crystal) 3183, 1656, cm^{-1}; δ_H (500 MHz, D_2O, diagnostic resonances) 1.71 (2H, t, *J* 12.2 Hz, $H3f_{ax}$, $H3f'_{ax}$), 1.84, 1.89, 1.90, 1.92, 1.93 (15H, 5 x s, 5 x CH_3), 2.55 (2H, dd, $J_{3eq\text{-}4}$ 3.9 Hz, $J_{3eq\text{-}3ax}$ 12.8 Hz, $H3f_{eq}$, $H3f'_{eq}$), 2.85 (4H, t, *J* 5.9 Hz, 2 x CH_2S), 4.29 (2H, m, H1e, H1e'), 4.45 (2H, br, H1d, H1d'), 4.60 (1H, d, H1b), 4.81 (1H, s, H1c'), 4.98 (1H, s, H1c), 5.94 (1H, d, $J_{1\text{-}2}$ 7.2 Hz, H1a), 7.16-7.18 (2H, m, 2 x ArH), 7.71 (4H, d, *J* 3.9 Hz, 4 x ArH), 8.27 (2H, d, *J* 4.5 Hz, 2 x ArH); δ_C (125.8 MHz, D_2O, diagnostic resonances) 12.9 (C=N($\underline{C}H_3$)), 21.9, 22.0, 22.3, 22.4, 22.4 (5 x CH_3), 35.4 (CH_2S), 37.1 ($C\mathit{H_2}CH_2S$), 40.0 (C3f, C3f'), 97.0 (C1c'), 99.4, 99.9 (C1d, C1d', C1c), 100.4 (C2f, C2f'), 101.4 (C1b), 103.6 (C1e, C1e'), 121.6, 122.1, 138.7, 149.3, 158.2 (5 x Ar-C), 138.6 (Ar-C), 168.5, 169.2 (C1f, C1f'), 173.2, 174.6, 175.0 (4 x *C*OCH_3); LRMS (ESI^+): *m/z* 2340 (MH^+, 2), 1070 ($M{\cdot}2H^+$, 100%); HRMS (ESI^+) ion series calculated for $C_{90}H_{140}N_9O_{54}NaS_4$ 1180.86276, 1181.36444, 1181.86611, 1182.36234, 1182.86401, 1183.36566; observed: 1180.86658, 1181.36765, 1181.86727, 1182.36738.

Decasaccharide lactol bearing iodoaryl groups ***5e***

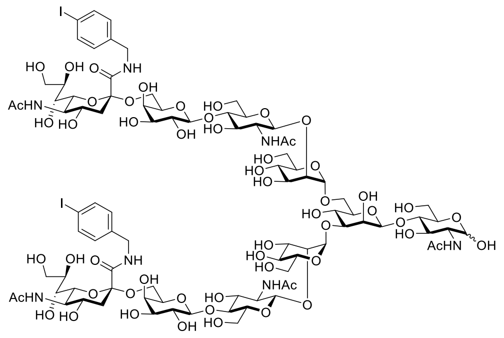

Lactol 4 (4.0 mg, 1.98 µmol) was dissolved in an aqueous solution of *para*-iodobenzylamine hydrochloride (10.7 mg, 0.32 M solution in 3:1 water-methanol). 4-(4,6-Dimethoxy-1,3,5-triazin-2-yl)-4-methylmorpholinium chloride (9.67 mg, 34.9 µmol) was added and the reaction warmed to 50 ºC. After 16 h t.l.c. analysis (IPA:1 M ammonium acetate, 3:2) indicated complete consumption of the diacid starting material (R_f 0.60) and formation of a major product (R_f 0.84). The reaction mixture was cooled and the reaction mixture placed in a centrifugal filter unit (0.22 µm membrane) and subjected to centrifugation (13,000 g, 1 min.) to remove the white precipitate. The cake was twice washed with water (30 µL) and centrifuged. The reaction mixture was subjected to PD MiniTrap G10 purification, eluting with water. Fractions containing glycan product were collected and lyophilised to afford 5e as a white solid, 3.27 mg, 67%; ν_{max} (single crystal) 3296, 2928, 1642, 1555 cm^{-1}; δ_H (500 MHz, D_2O/acetone-D_6) 1.76 (2H, t, *J* 12.3 Hz, $H3f_{ax}$, $H3f'_{ax}$), 1.94, 1.95, 1.98 (15H, 3 x s, 5 x CH_3), 2.60 (2H, dd, $J_{3eq\text{-}4}$ 4.4 Hz, $J_{3eq\text{-}3ax}$ 12.9 Hz, $H3f_{eq}$, $H3f'_{eq}$), 3.39-3.48 (6H, m, H4c, H4c', H2e, H2e', H6e, H6e'), 3.48-3.59 (14H, m, H5b, H5c', H6c, H6c', H4d, H4d', H5d, H5d', H3e, H3e', H7f, H7f', H9f, H9f'), 3.61-3.77 (28H, m, H3a, H4a, H6a, H6'a, H3b, H4b, H6b, H5c, H2d, H2d', H3d, H3d', H6d, H6d', H4e, H4e', H5e, H5e', H4f, H4f', H5f, H5f', H6f, H6f', H8f, H8f', H9'f, H9'f'), 3.77-3.90 (11H, m, H2a, H5a, H6'b, H3c, H3c', H6'c, H6'c', H6'd, H6'd', H6'e, H6'e'), 4.04 (1H, s, H2c'), 4.11 (1H, s, H2c), 4.18 (1H, s, H2b), 4.25-4.34 (6H,

m, H1e, H1e’, 2 x $ArCH_2$), 4.51 (2H, d, J_{1-2} 7.4 Hz, H1d, H1d’), 4.64 (0.3H, d, H1a(β)), 4.69 (1H, d, J 4.3 Hz, H1b), 4.86 (1H, s, H1c’), 5.05 (1H, s, H1c), 5.14 (0.7H, d, J_{1-2} 3.1 Hz, H1a(α)), 7.07 (4H, d, J 8.2 Hz, 4 x ArH (meta to I)), 7.71 (4H, d, J 8.2 Hz, 4 x ArH (ortho to I)); δ_C (125.8 MHz, D_2O/acetone-D_6) 21.9, 22.0, 22.4 (5 x CH_3), 38.0 (C3f, C3f’), 42.8, 43.2 (2 x $ArCH_2$), 51.7 (C5f, C5f’), 53.2 (C2a(α)), 54.5 (C2d, C2d’), 57.0 (C2a(β)), 60.0 (C6d, C6d’), 60.1 (C6a), 61.6, 61.7 (C6c, C6c’), 62.0, 62.9 (C9f, C9f’), 63.9 (C6e, C6e’), 66.9 (C4b), 67.3 (C6b), 67.7 (C4c, C4c’, C4f, C4f’), 68.2 (C4e, C4e’), 69.1, 69.4 (C7f, C7f’), 69.9(C3c, C3c’), 70.2 (C2b), 70.6 (C2e, C2e’), 71.0 (C8f, C8f’), 72.0 (C3d, C3d’), 72.4 (C3e, C3e’), 72.8 (C3a), 73.4 (C5c, C5c’, C6f, C6f’), 73.5 (C5e, C5e’), 73.5 (C5a, C5b), 74.3 (C5d, C5d’), 76.2 (C2c’), 76.3 (C2c), 80.2 (C4a), 80.4 (C2b), 80.7, 80.8 (C4d, C4d’), 90.5 (C1a(α)), 92.6 (ArCI), 94.9 (C1a(β)), 96.9 (C1c’), 99.2, 99.3, 99.3 (C1d, C1d’, C2f, C2f’), 99.5 (C1c), 100.4 (C1b), 103.5 (C1e, C1e’), 129.9 (4 x ArC), 137.3 (2 x ArC), 137.8 (4 x ArC), 169.2, 169.3 (C1f, C1f’), 174.5, 174.6, 175.0 (5 x C=O); LRMS (ESI^+): *m/z* 2474 ($M·Na^+$, 15), 2451 ($M·H^+$, 15), 1278 ($M·Na^+·H^+$, 45), 1226 ($M·2H^+$, 100%); HRMS (ESI^+) ion series calculated for $C_{90}H_{138}I_2N_7O_{55}$ 2450.63006, 2451.63343, 2452.63676, 2453.64004, 2454.64116, observed: 2450.64076, 2451.64418, 2452.64270, 2453.64983, 2454.65455.

*Decasaccharide oxazoline bearing iodoaryl groups **6e***

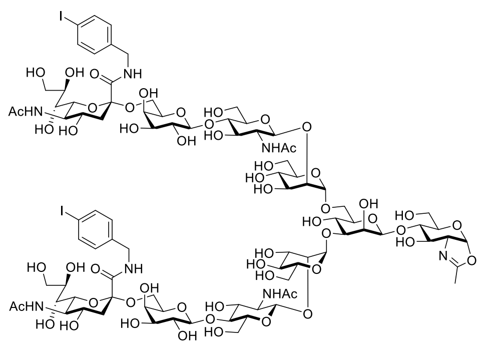

Lactol **5e** (1.53 mg, 624 nmol) was dissolved in water (25 μL). CDMBI (1.36 mg, 6.24 μmol) was added, followed by potassium phosphate tribasic (25 μL of a 0.75 M solution). The mixture was vortexed and the shaken at 4 °C for 4.5 hours. After this time ESI-MS analysis indicated incomplete consumption of lactol so further CDMBI and base were added. After a further 18 h analysis indicated formation of oxazoline product. The mixture was filtered through a centrifugal spin filter (0.2 μm membrane) and applied to a PD Miditrap G25 column. The product was eluted with 0.01% ammonia solution; pure fractions containing glycan were collected and lyophilised to afford **6e** as a white fluffy solid, 1.15 mg (75%); $[\alpha]_D^{25}$ = - 0.22° (c = 0.06, H_2O); ν_{max} (single crystal) 3183, 1656, 1554 cm^{-1}; δ_H (500 MHz, D_2O, diagnostic resonances) 1.75 (2H, t, J 12.3 Hz, $H3f_{ax}$, $H3f'_{ax}$), 1.87, 1.89, 1.93, 1.94, 1.98 (15H, 5 x s, 5 x CH_3), 2.59 (2H, dd, J_{3eq-4} 4.4 Hz, $J_{3eq-3ax}$ 12.9 Hz, $H3f_{eq}$, $H3f'_{eq}$), 4.03 (1H, s, H2c’), 4.10 (1H, s, H2c), 4.24-4.33 (7H, m, H2b, H1e, H1e’, 2 x $ArCH_2$), 4.50 (2H, br d, J_{1-2} 4.0 Hz, H1d, H1d’), 4.68 (1H, br s, H1b), 4.86 (1H, s, H1c’), 5.04 (1H, s, H1c), 6.00 (1H, d, J_{1-2} 7.3 Hz, H1a), 7.06 (4H, d, J 8.1 Hz, 4 x ArH (meta to I)), 7.69 (4H, d, J 8.2 Hz, 4 x ArH (ortho to I)); δ_C (125.8 MHz, D_2O, diagnostic resonances) 22.0, 22.4 (5 x CH_3), 38.0 (C3f,

C3f'), 42.8, 43.2 (2 x $ArCH_2$), 92.6 (ArCI), 96.9 (C1c'), 99.2, 99.3, 99.4 (C1c, C1d, C1d'), 100.4, 100.6 (C2f, C2f'), 101.4 (C1b), 103.6 (C1e, C1e'), 129.9 (4 x ArC), 137.3 (2 x ArC), 137.8 (4 x ArC), 169.2 (C1f, C1f'), 174.6, 175.0 (5 x C=O); HRMS (ESI^+) ion series calculated for $C_{90}H_{136}N_7I_2O_{54}Na$ 1227.80436, 1228.30604, 1228.80771, 1229.30935, 1229.80991, 1230.31154; observed: 1227.80306, 1228.30494, 1228.80702, 1229.30925, 1229.78793, 1230.28991.

2.2.3 NMR data

SGAsn-Fmoc

1H NMR, 700 MHz, D_2O/acetone-D_6

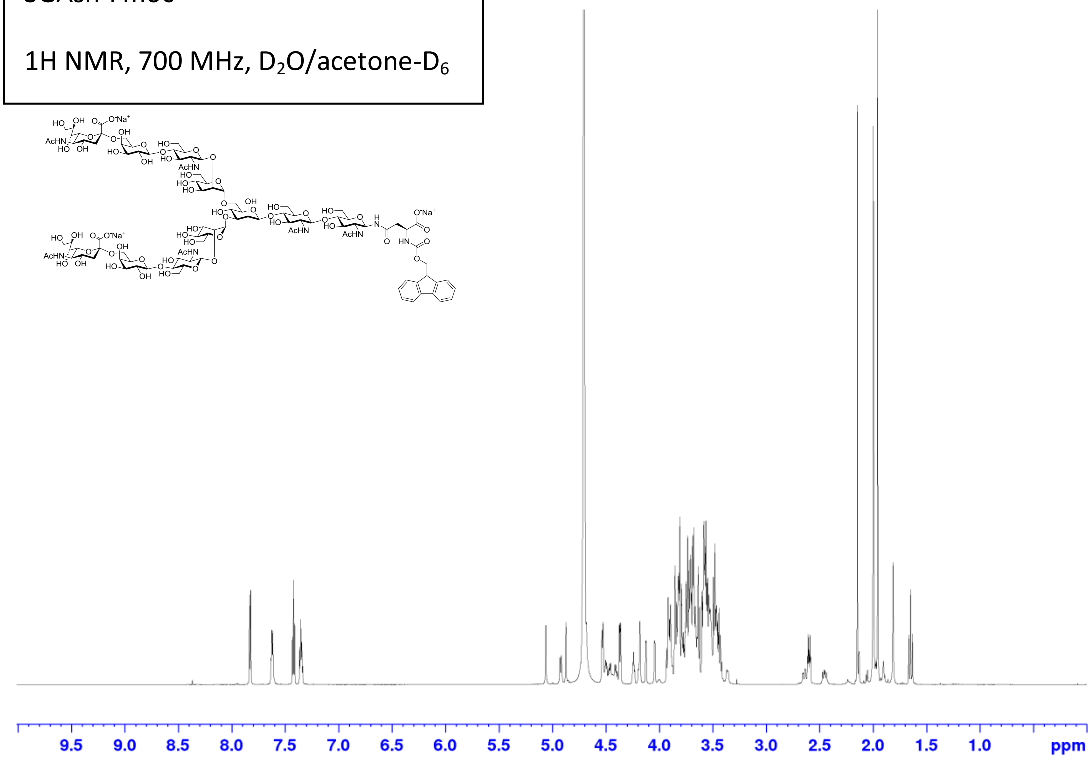

SGAsn-Fmoc

13C NMR, 176 MHz, D_2O/acetone-D_6

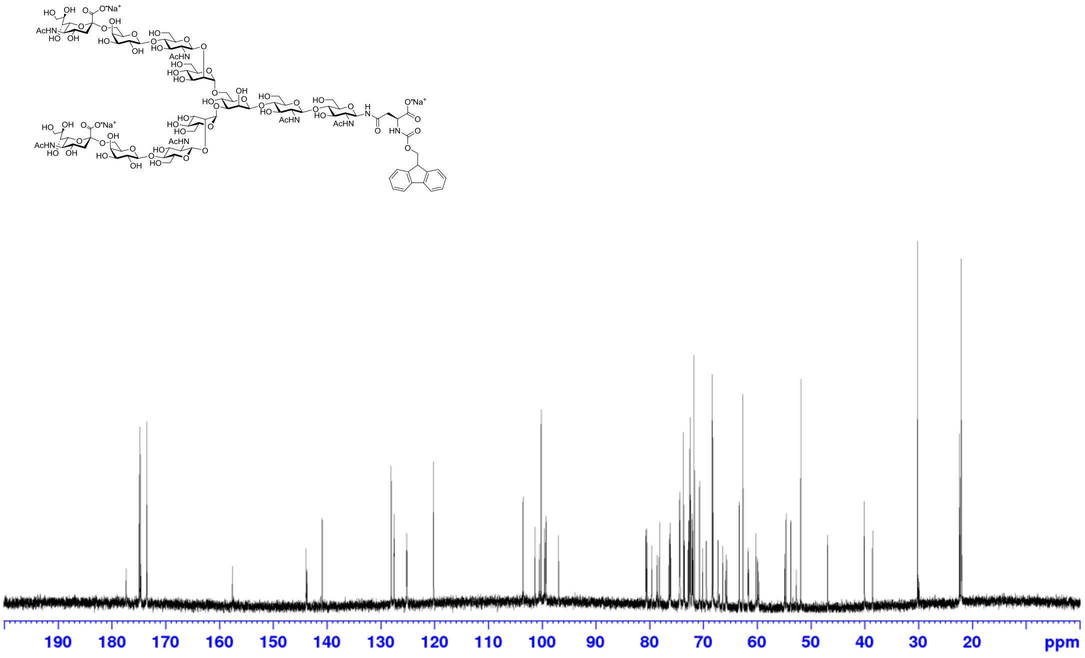

Diacid Lactol **4**

1H NMR, 400 MHz, D_2O

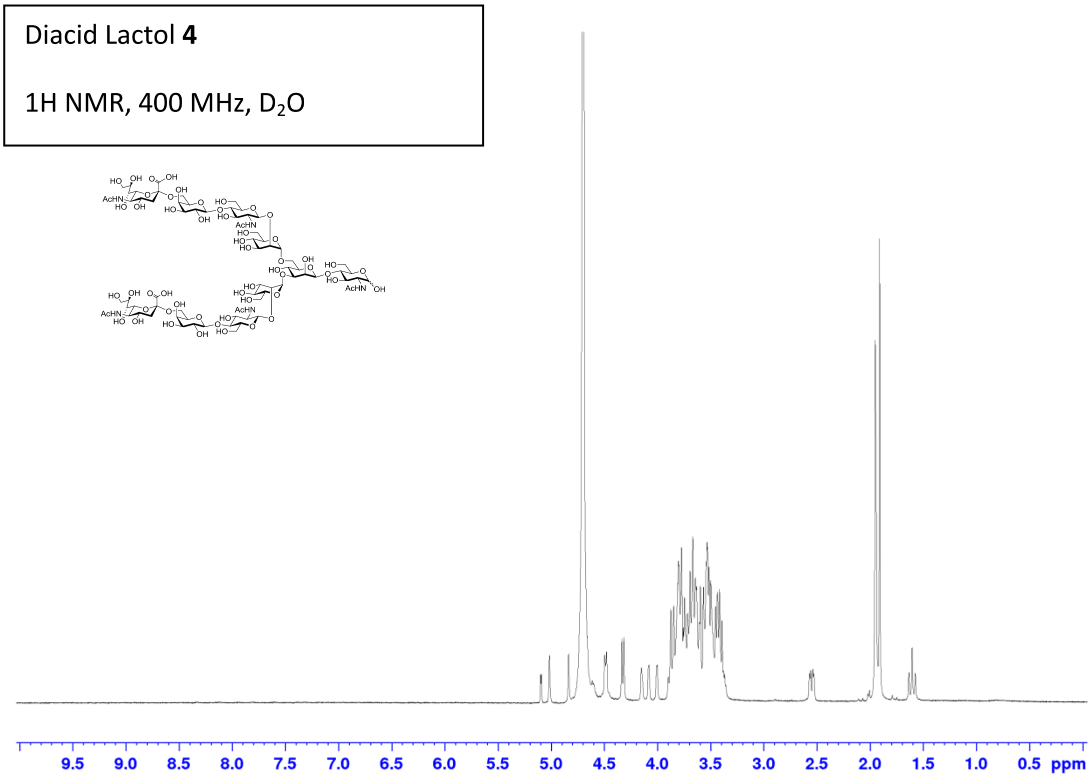

Diacid Lactol **4**

13C NMR, 125 MHz, D_2O

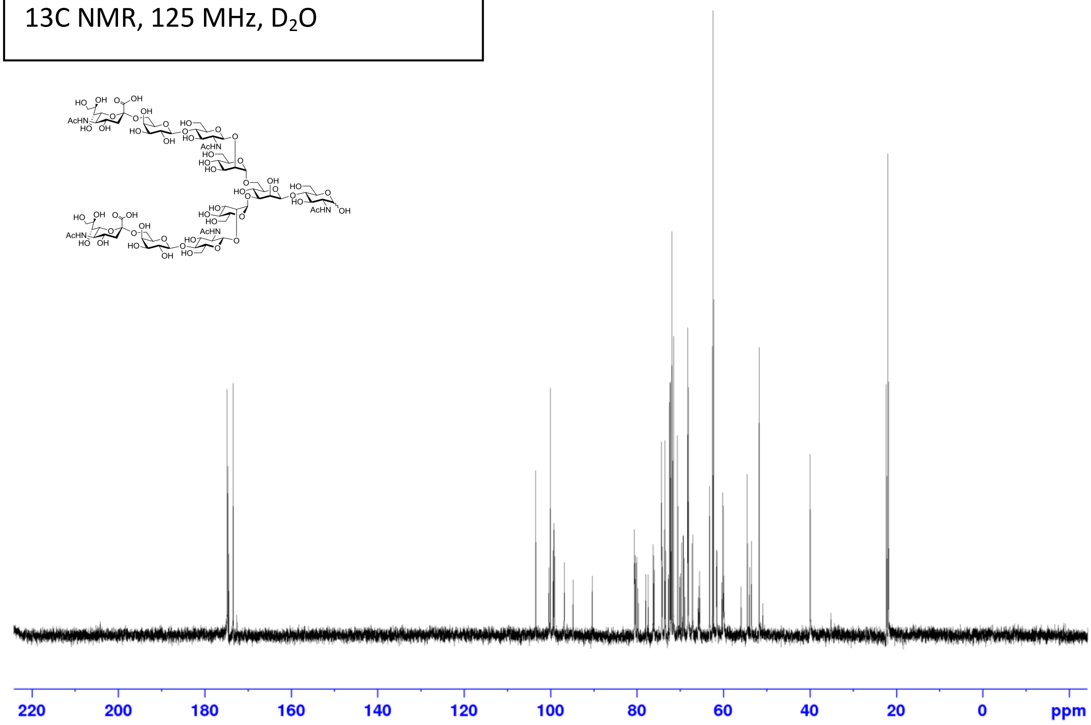

Diacid oxazoline **2**

1H NMR, 700 MHz, D_2O

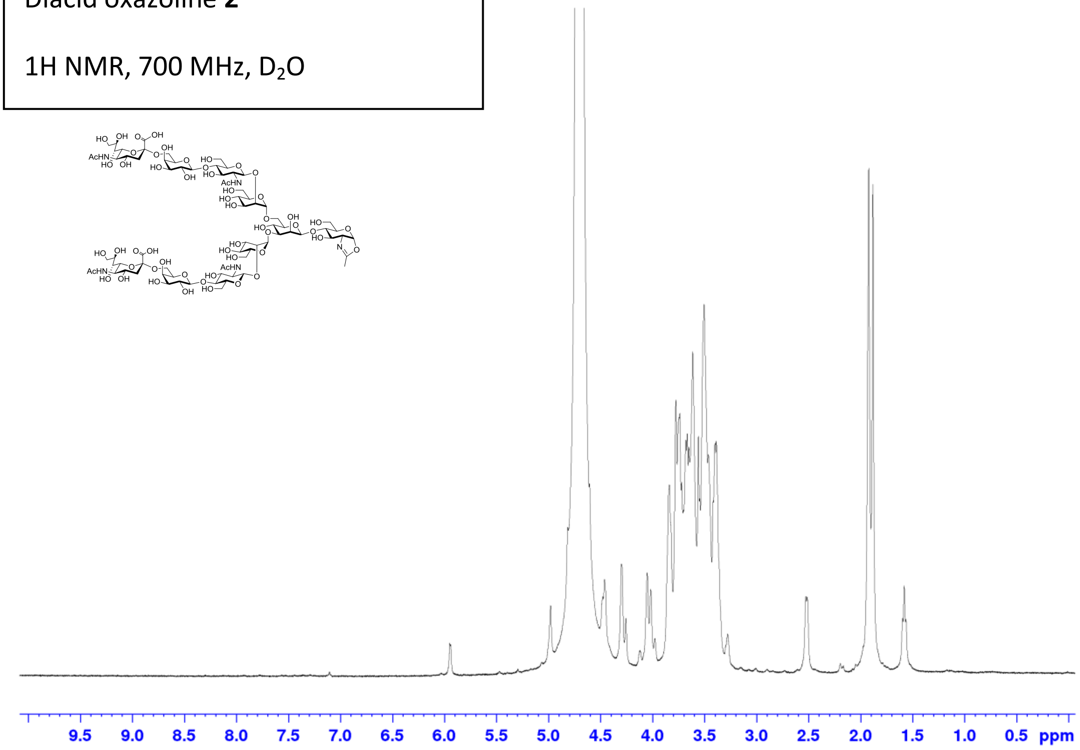

Diacid oxazoline **2**

HSQC, 700 MHz, D_2O

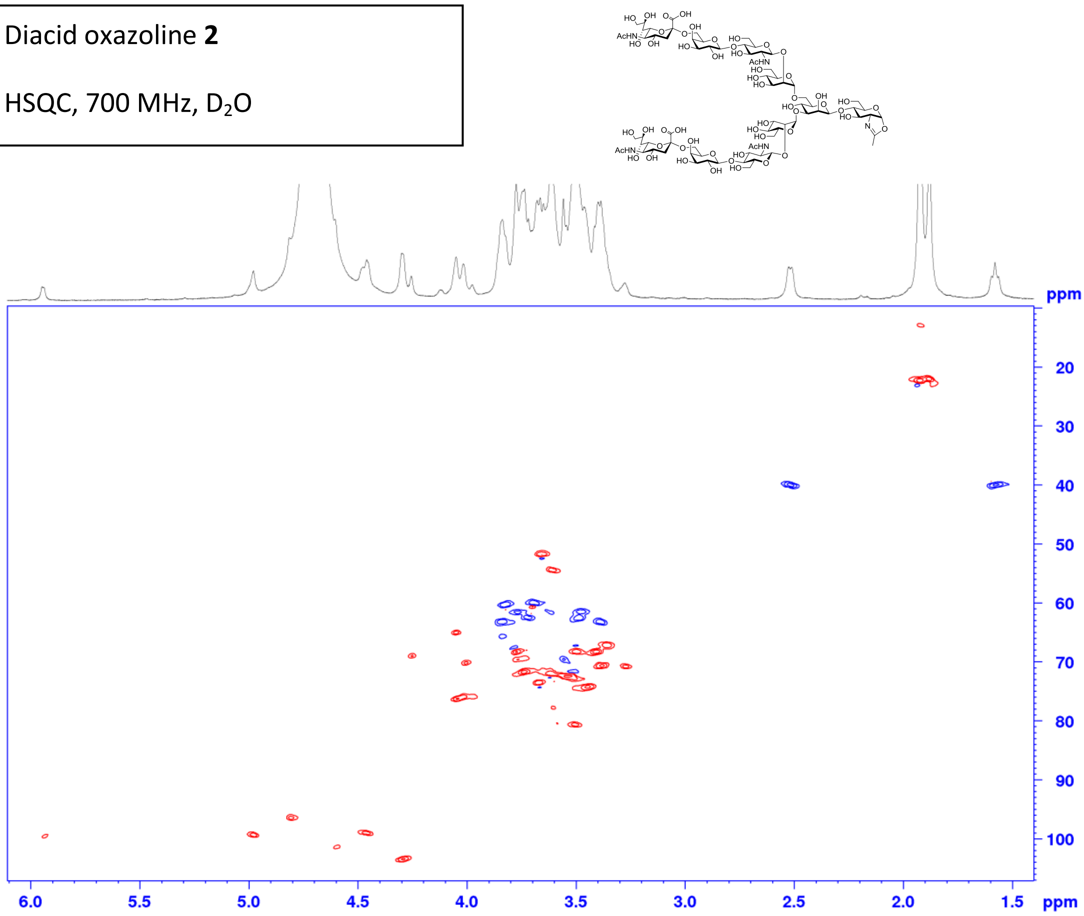

Alkyne lactol **5a**

1H NMR, 700 MHz, D_2O

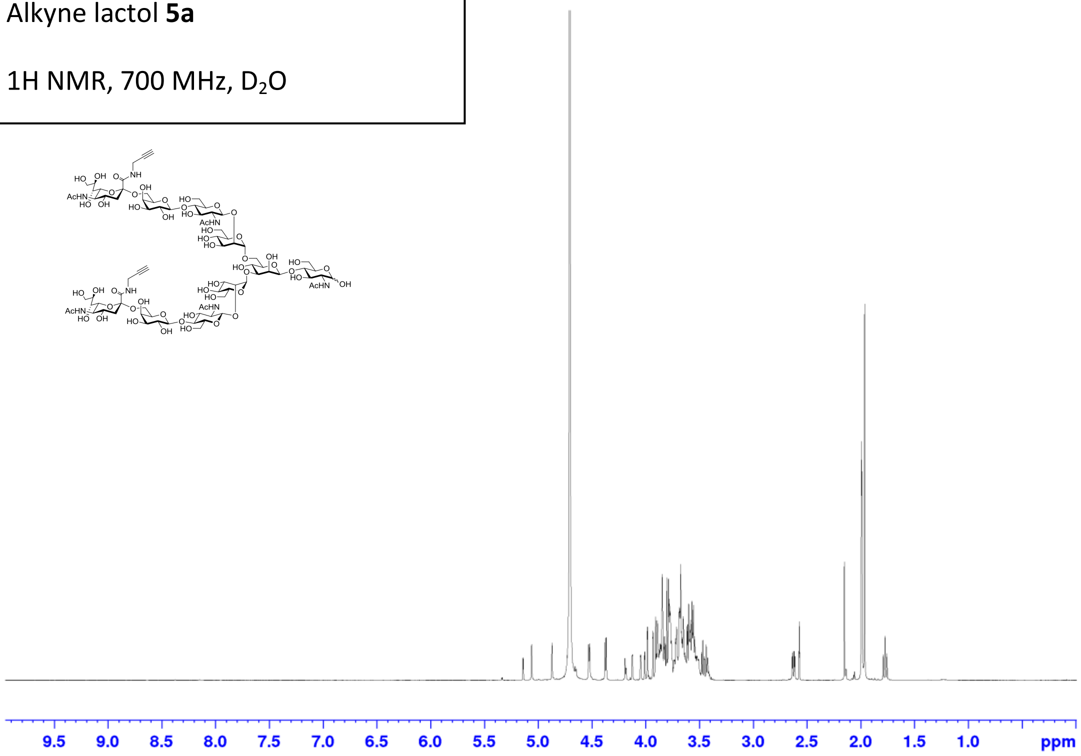

Alkyne lactol **5a**

13C NMR, 175 MHz, D_2O

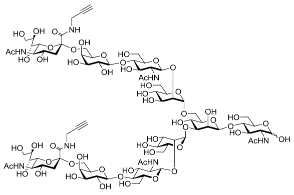

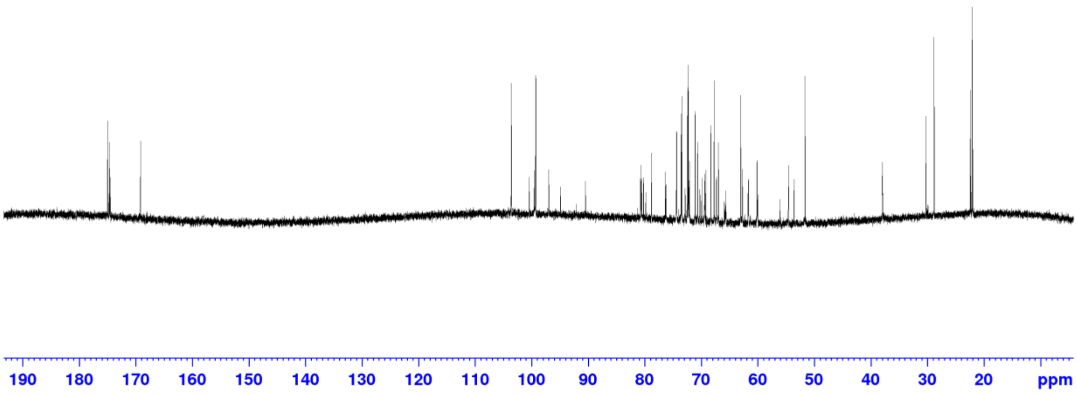

Alkyne oxazoline **6a**

1H NMR, 600 MHz, D_2O

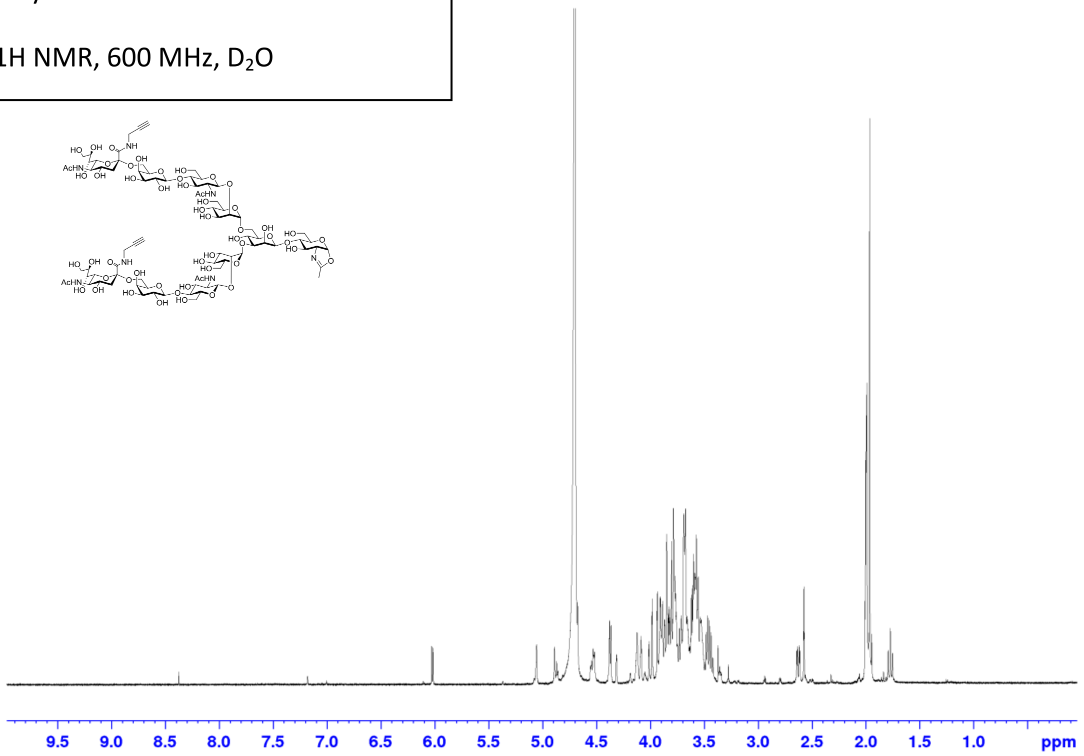

Alkyne oxazoline **6a**

13C NMR, 125 MHz, D_2O

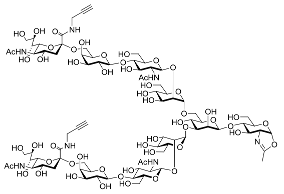

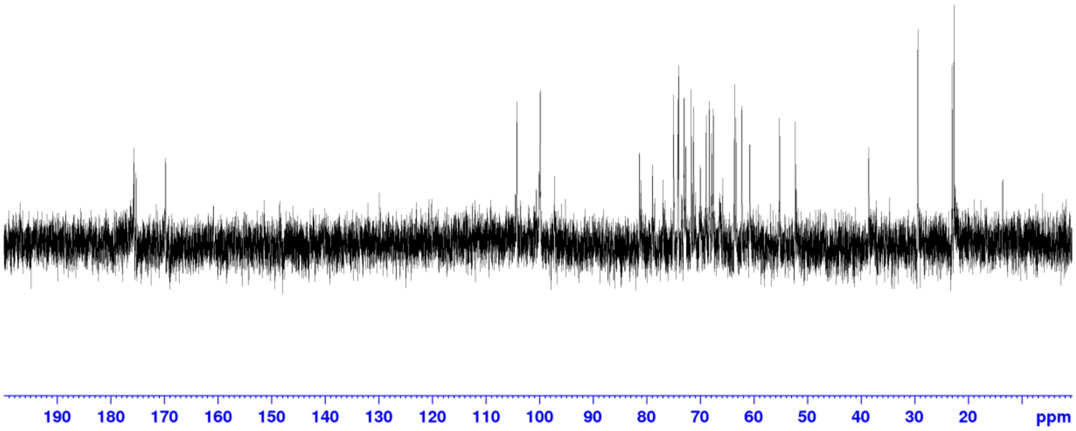

Azide lactol **5b**

1H NMR, 500 MHz, D_2O

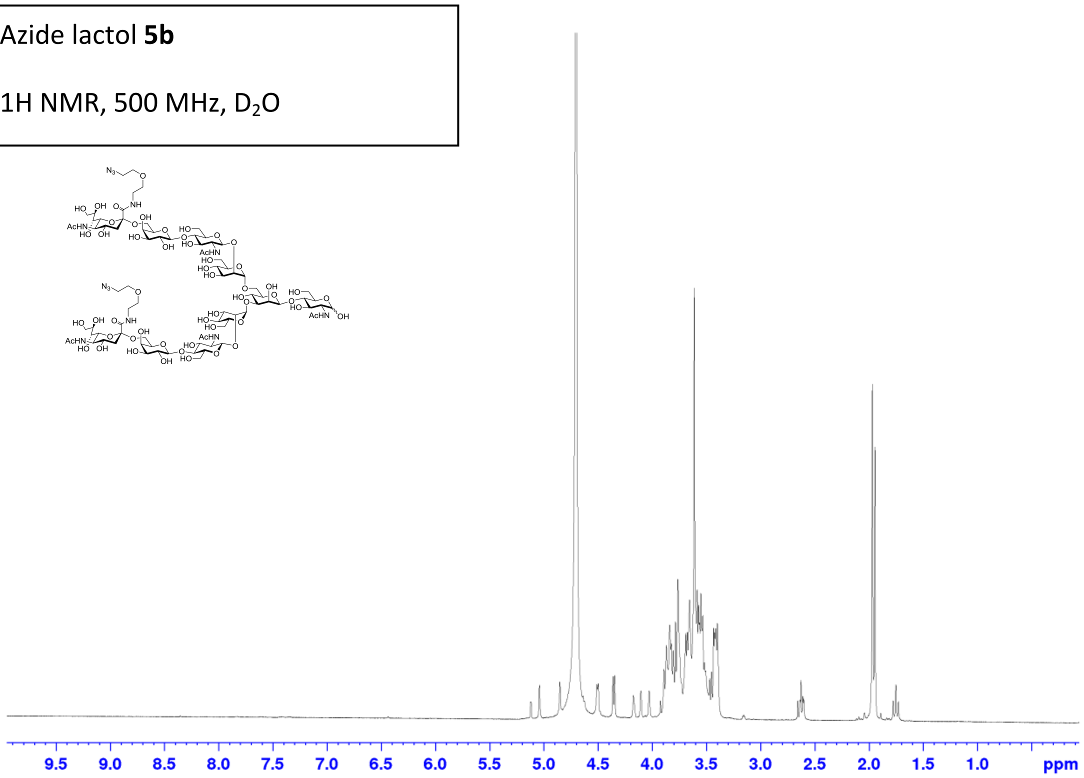

Azide lactol **5b**

13C NMR, 125 MHz, D_2O

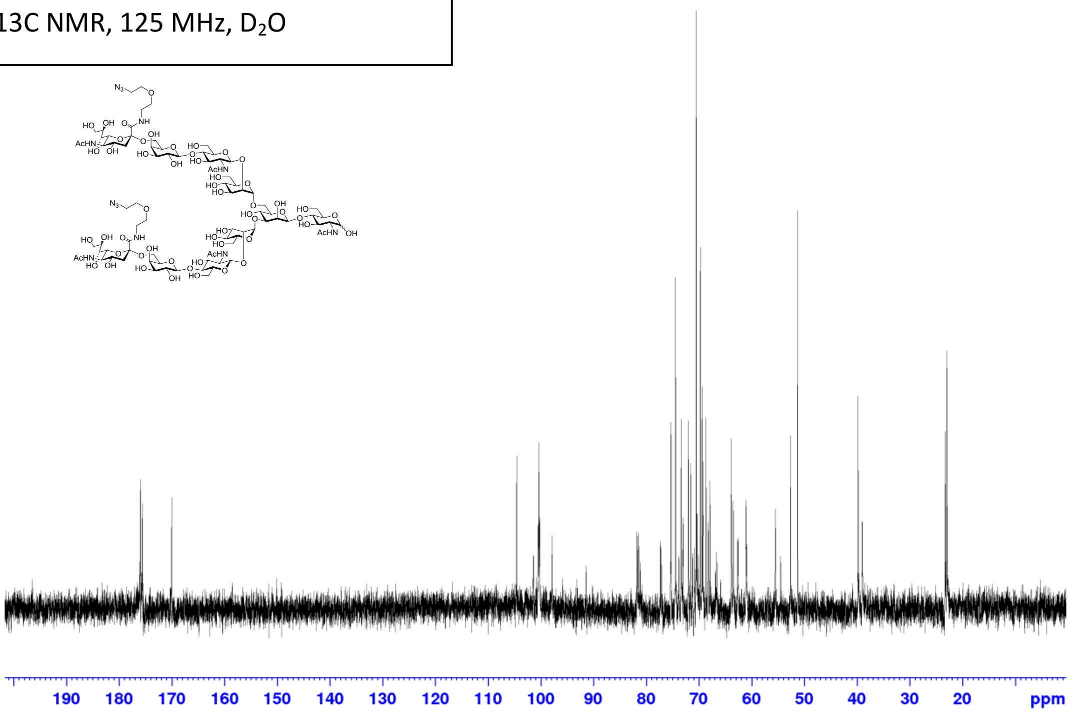

Azide oxazoline **6b**

1H NMR, 500 MHz, D_2O

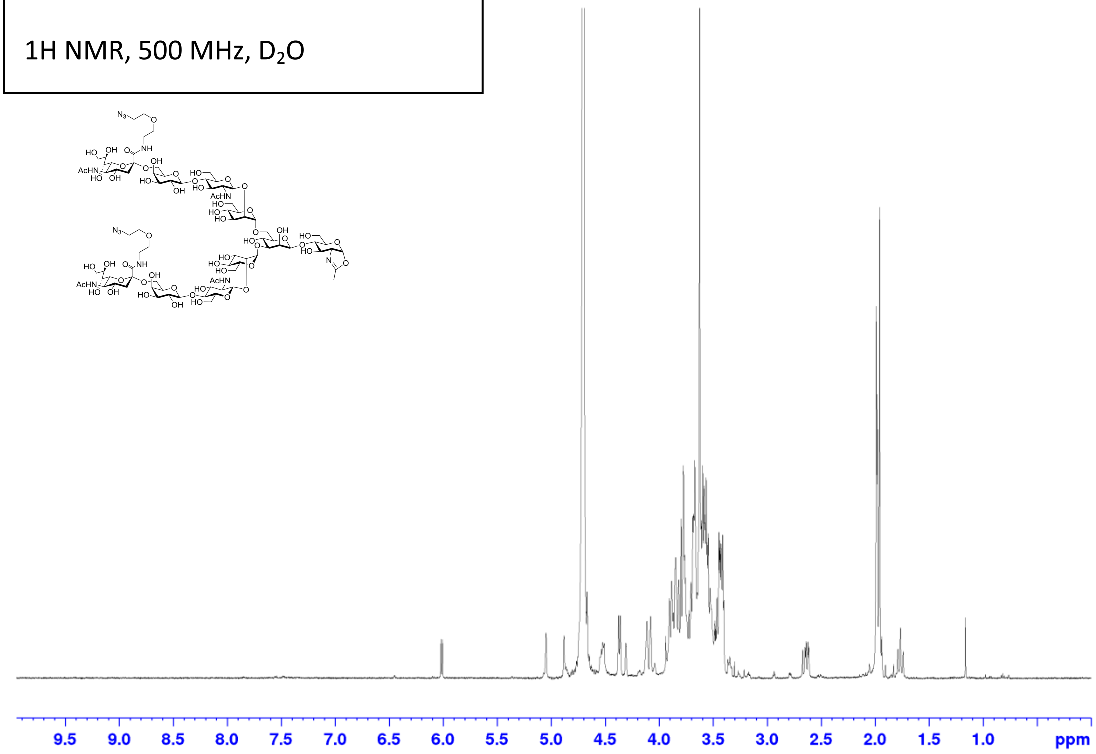

Azide oxazoline **6b**

13C NMR, 125 MHz, D_2O

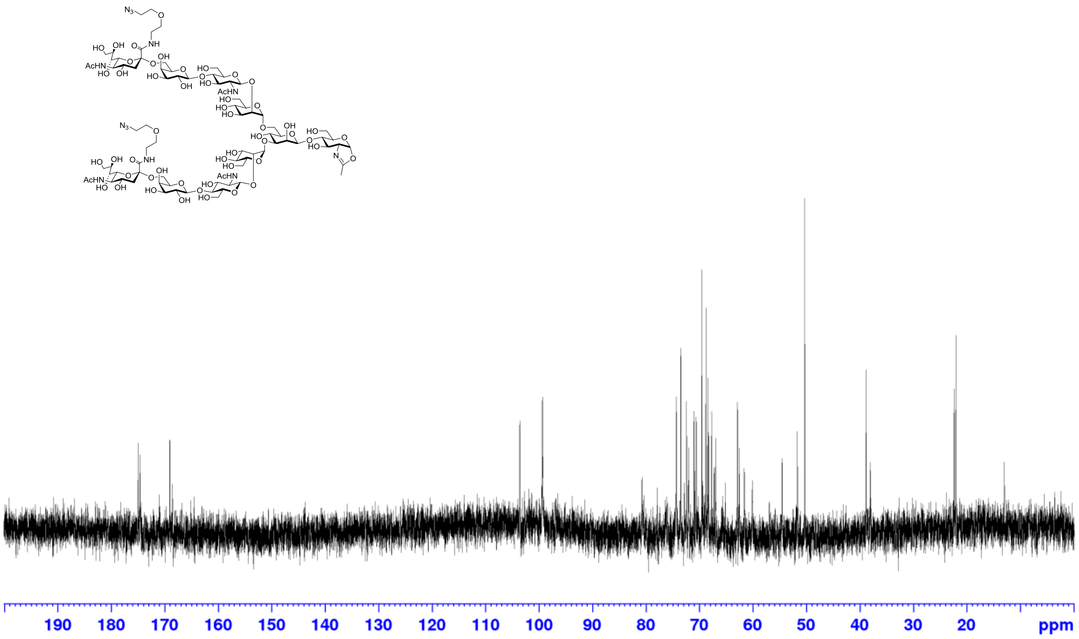

SSMe lactol **5c**

1H NMR, 500 MHz, D_2O

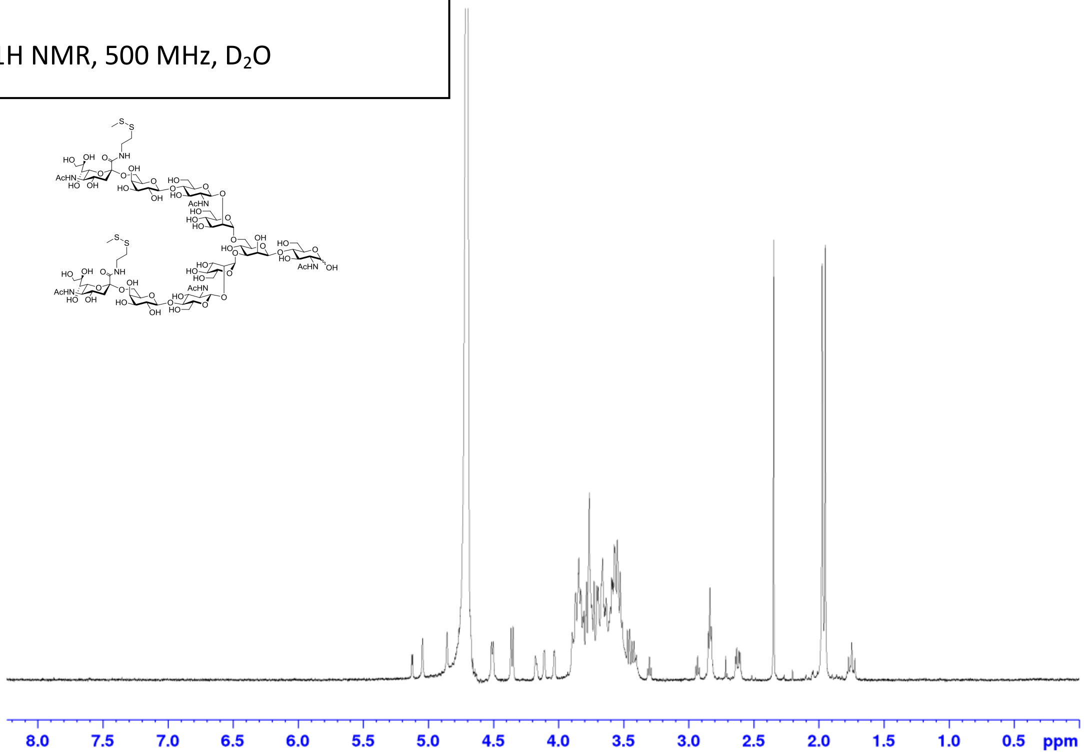

SSMe lactol **5c**

13C NMR, 125 MHz, D_2O

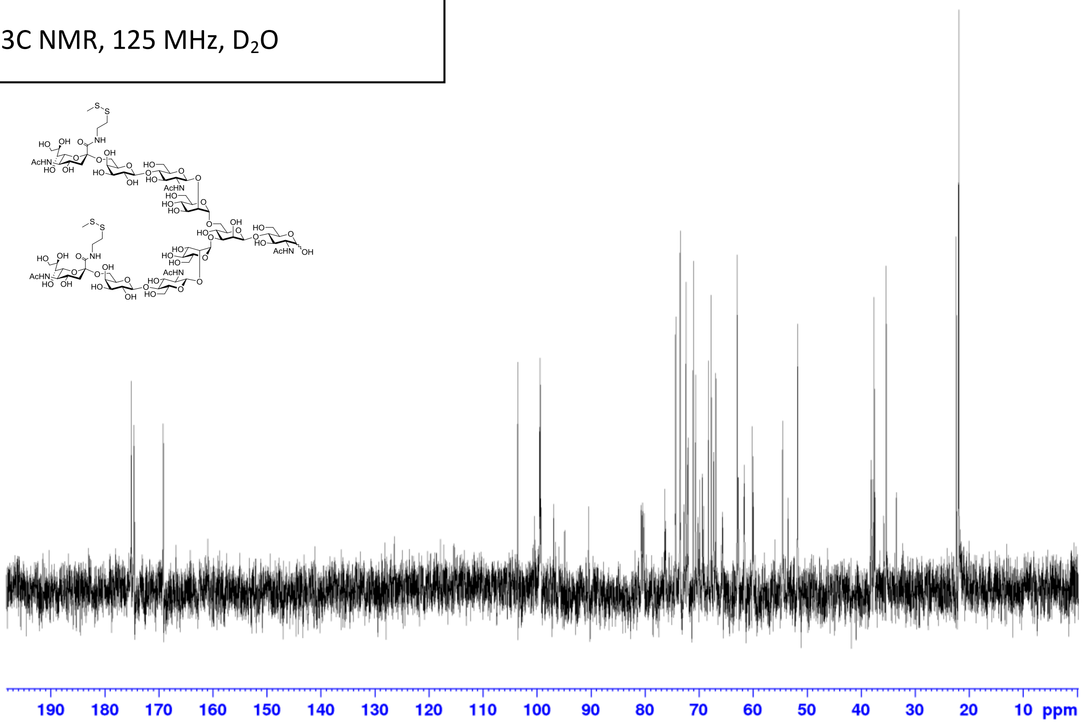

SH oxazoline **6c**

1H NMR, 500 MHz, D_2O

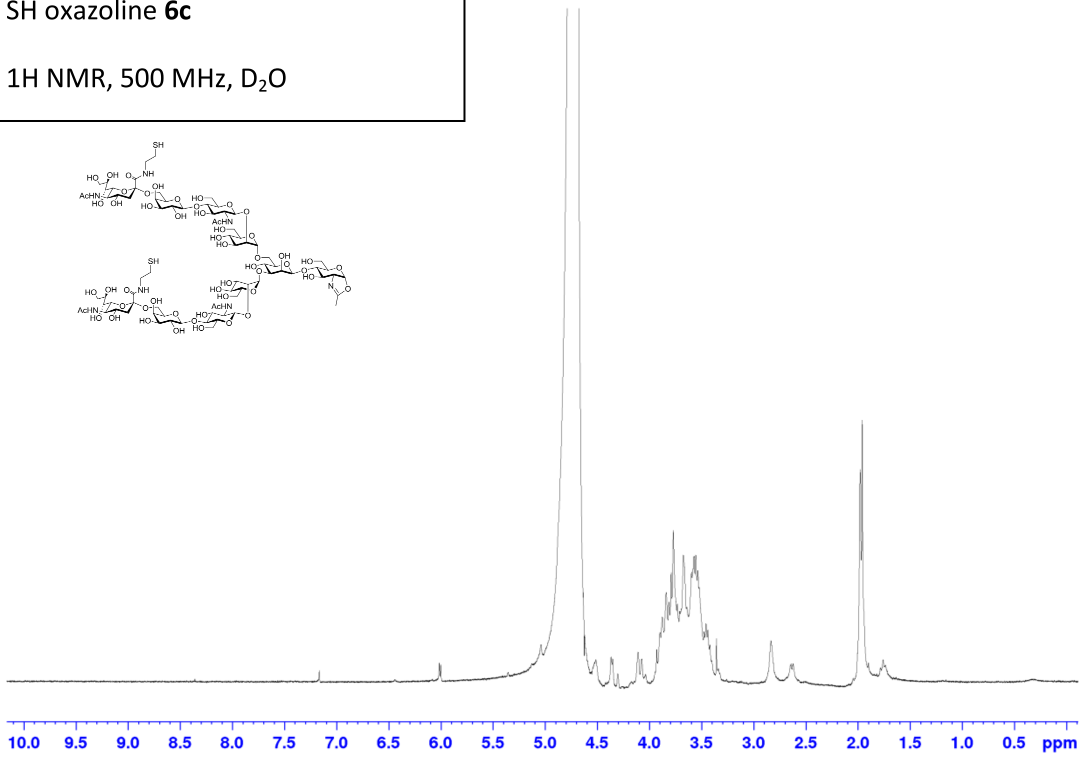

SH oxazoline **6c**

13C NMR, 125 MHz, D_2O

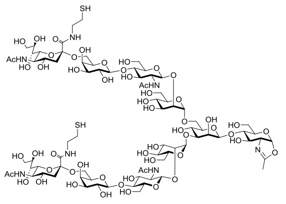

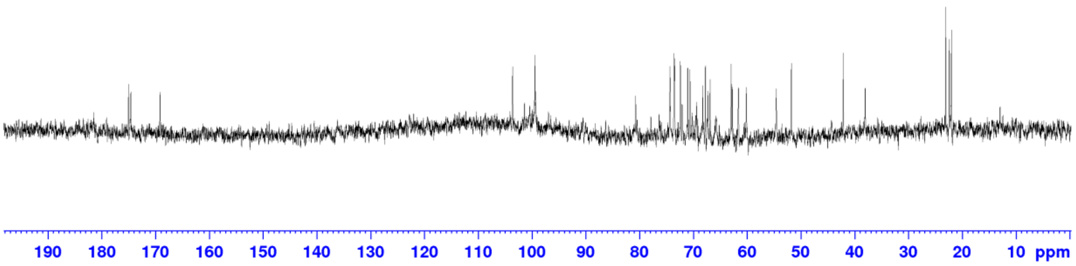

SSPy lactol **5d**

1H NMR, 500 MHz, D_2O

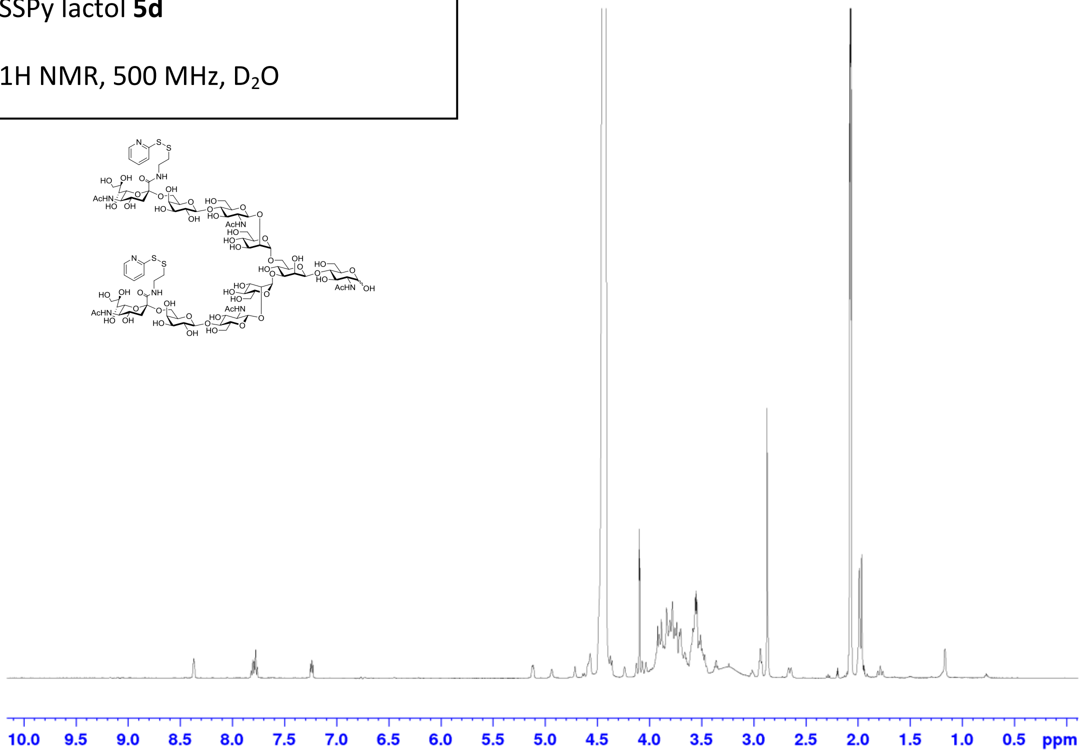

SSPy lactol **5d**

HSQC NMR, 600 MHz, D_2O

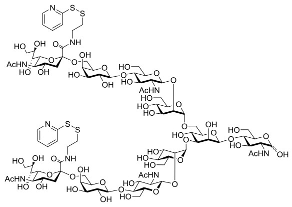

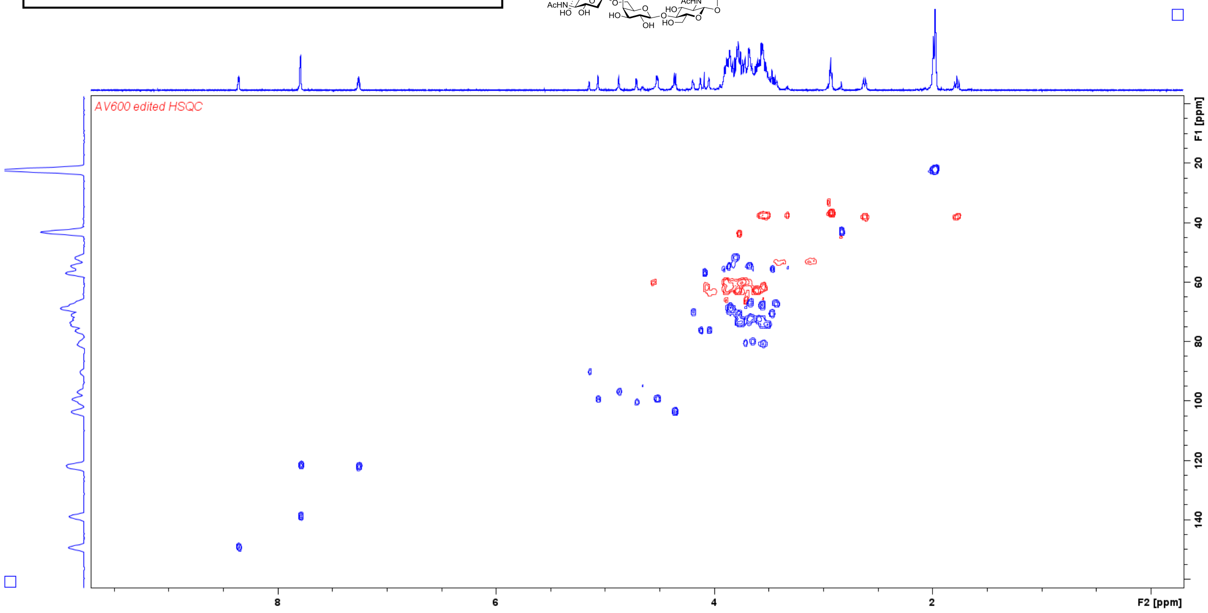

SSPy oxazoline **6d**

1H NMR, 700 MHz, D_2O

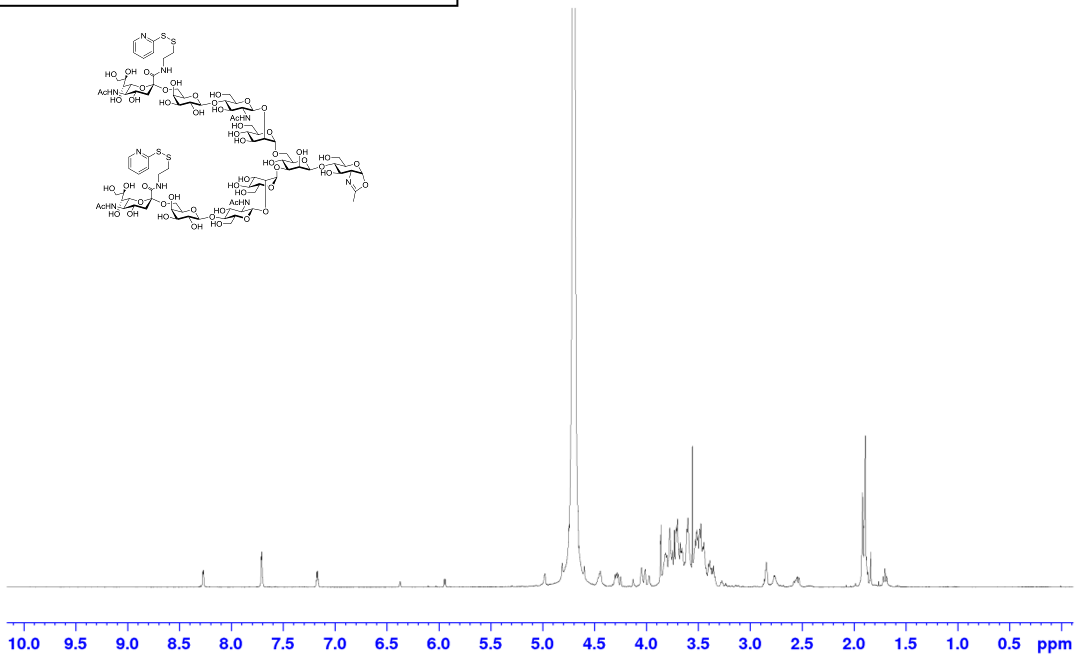

SSPy oxazoline **6d**

13C NMR, 125 MHz, D_2O

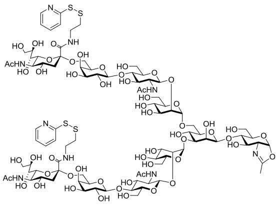

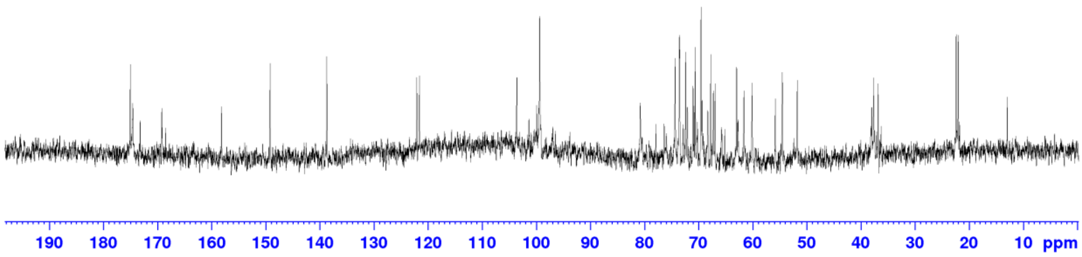

Iodoaryl lactol **5e**

1H NMR, 500 MHz, D_2O

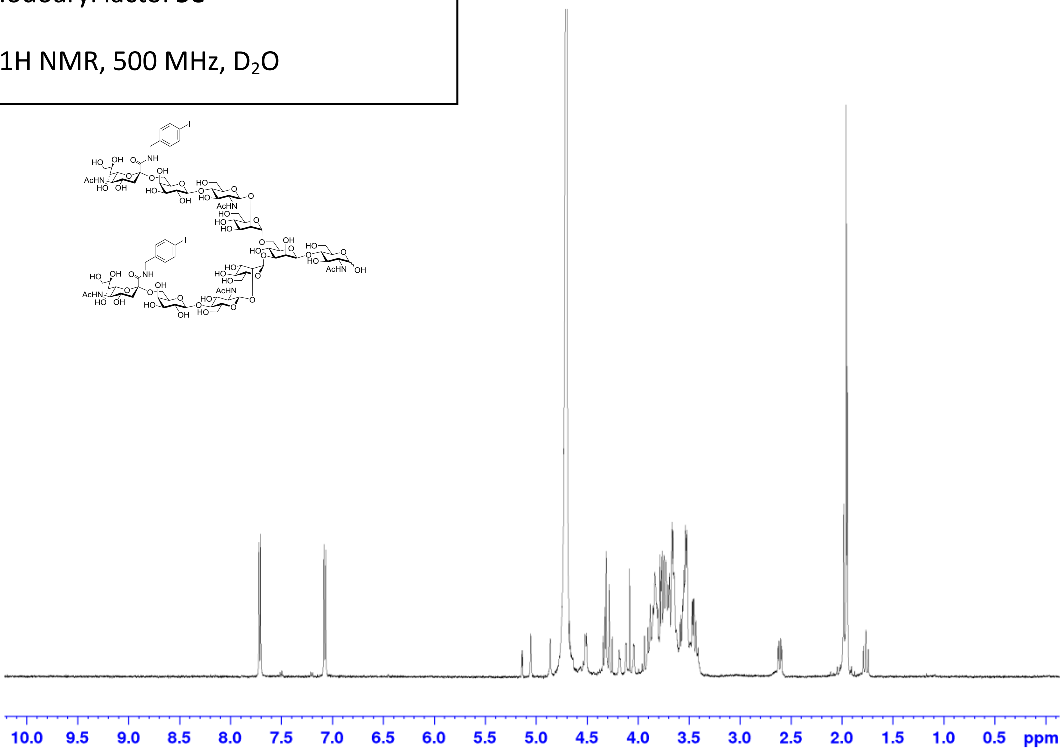

Iodoaryl lactol **5e**

13C NMR, 125 MHz, D_2O

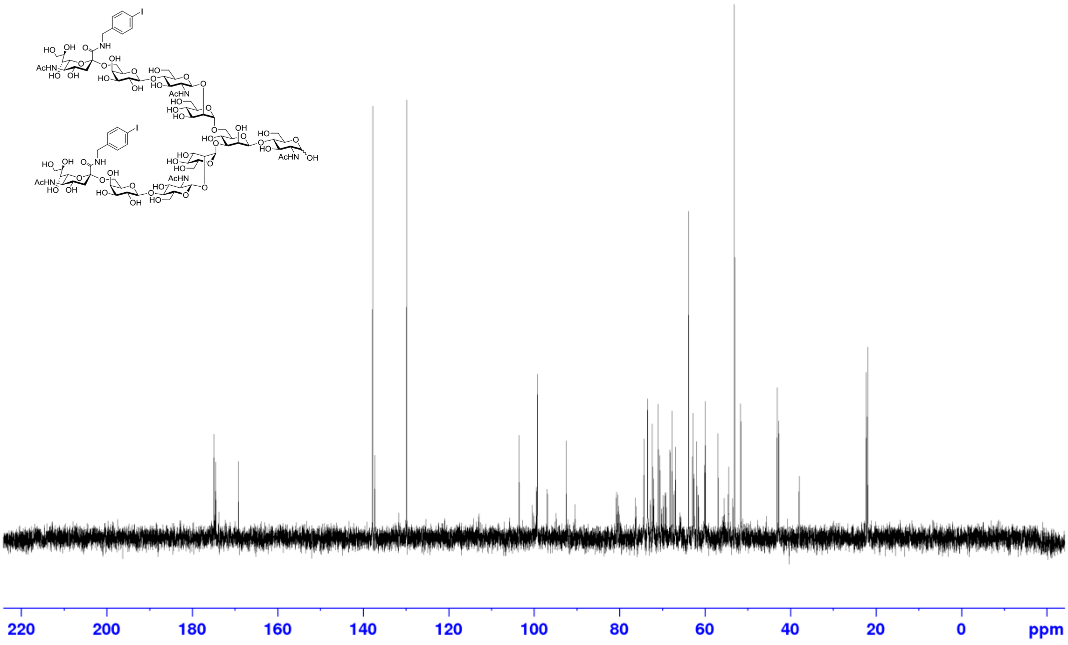

Iodoaryl oxazoline **6e**

1H NMR, 500 MHz, D_2O

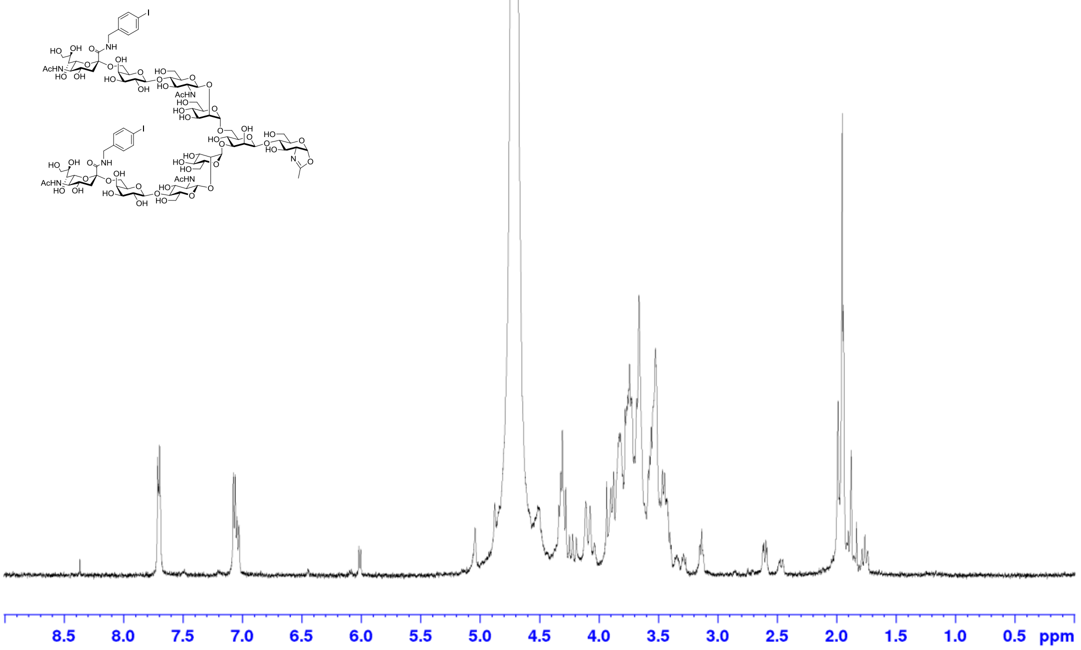

Iodoaryl oxazoline **6e**

13C NMR, 125 MHz, D_2O

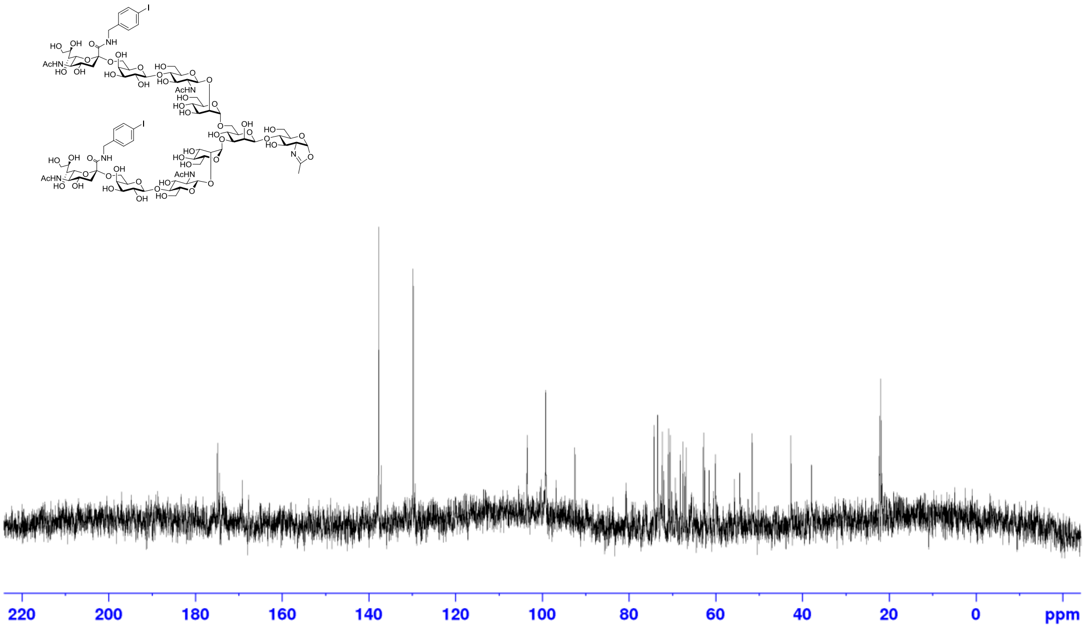

2.2.4 Synthesis of Rhodamine Cargo Molecule ***8a***

N-(9-(2-(4-((((1R,8S,9s)-Bicyclo[6.1.0]non-4-yn-9-yl)methoxy)carbonyl)piperazine-1-carbonyl)phenyl)-6-(diethylamino)-3H-xanthen-3-ylidene)-N-ethylethanaminium

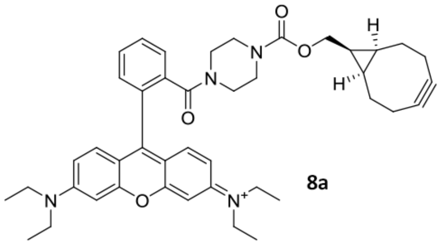

8a

((1R,8S,9s)-Bicyclo[6.1.0]non-4-yn-9-yl)methyl (2,5-dioxopyrrolidin-1-yl) carbonate (5.2 mg, 17.9 μmol) was dissolved in DCM (0.3 mL). *N*-(6-(Diethylamino)-9-(2-(piperazine-1-carbonyl)phenyl)-3H-xanthen-3-ylidene)-*N*-ethylethanaminium (rhodamine derivative, 12.7 mg, 23.2 μmol) was added, followed by trimethylamine (6.2 μ, 44.6 μmol). The reaction was stirred at room temperature for 2.5 hours after which time t.l.c. analysis (DCM-methanol, 9:1) indicated complete consumption of carbonate (Rf 0.90) and formation of a major product (Rf 0.44). The solvent was removed *in vacuo* and the residue purified by flash column chromatography on silica gel, eluting with DCM-methanol, 19:1, to afford **8a** (11.0 mg, 89%) as a metallic purple solid,

ν_{max} (neat) 3384, 2922, 2852, 2605, 2498, 2360, 1693, 1631, 1587, 1528, 1413 cm-1; LRMS (ESI+): *m/z* 687 (M+, 100%). The absorbance spectrum was recorded over the range 220-820 nm and showed absorbance below 300 nm and a λ_{max} at 565 nm (spectrum recorded at 100 μM; at 1 mM the spectrometer reached saturation point).

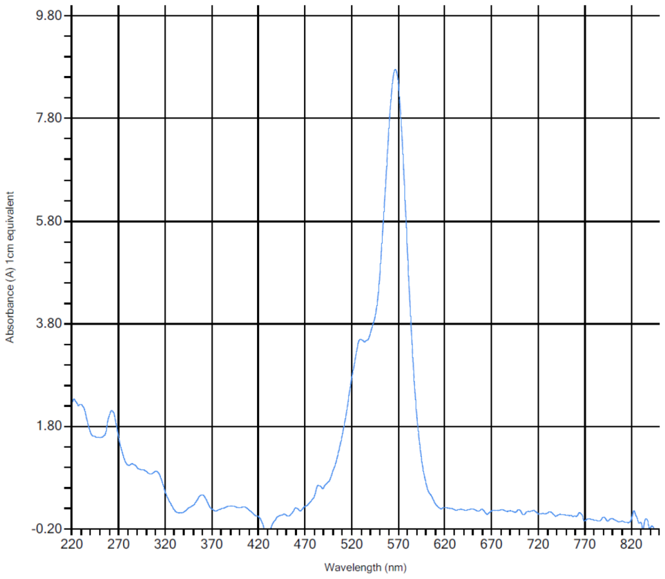

2.2.5 Synthesis of Cemadotin Cargo Molecule ***8b***

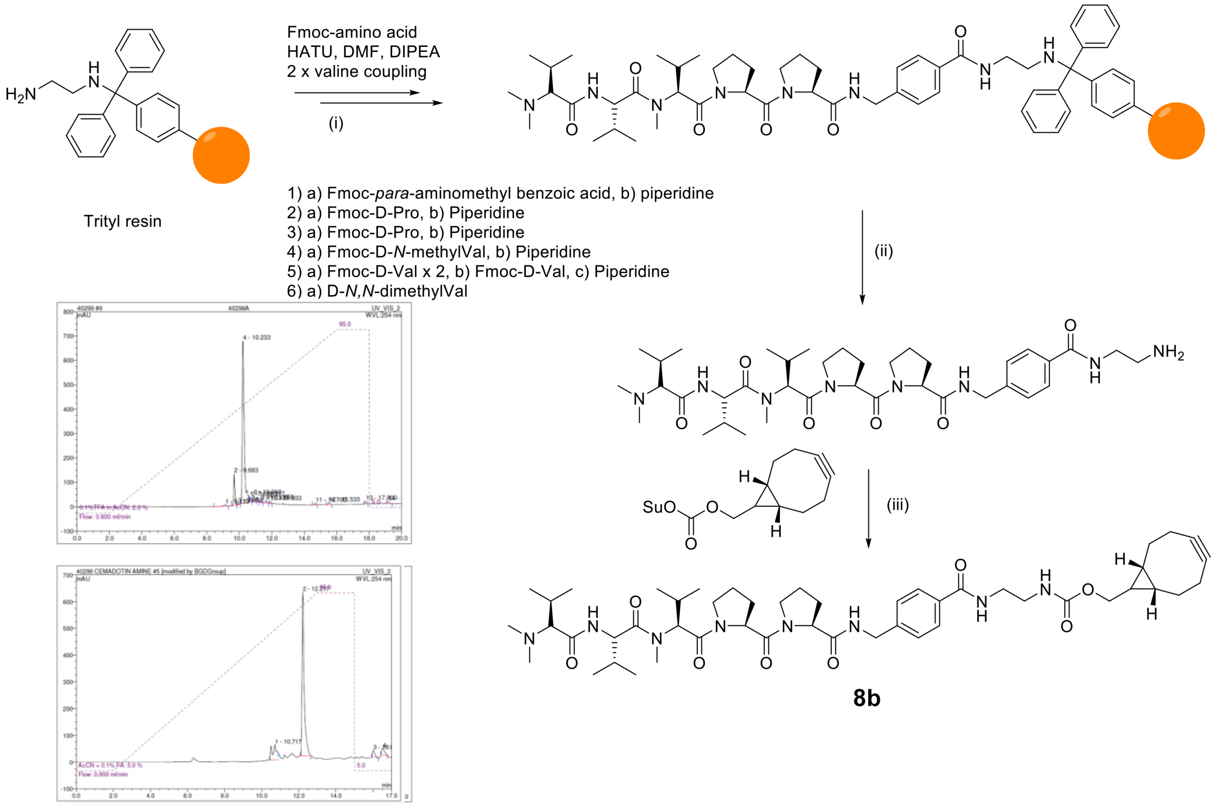

Synthesis of cemadotin derivative **8b**. (i) a) HATU, Fmoc-amino acid, DMF, 2 h, RT b) piperidine, DMF, 15 mins, RT; sequence as indicated; (ii) HFIP, DCM, RT, 30 mins, repeat once, 61% crude yield; (iii) DCM, Et_3N, purify by HPLC, 38% overall yield.

Trityl resin (1.2-1.7 mmol/g, 150 mg, 0.18-0.255 mm) was placed in a solid phase reaction vessel (Telos) equipped with a filtration frit and with stoppers for both ends. In a fresh flask appropriate amino acid was combined with HATU (1 equiv.) and the mixture suspended in DMF (2 mL). DIPEA (171 µL, 1.0 mmol) was added and the mixture shaken for two minutes, during which time all the components dissolved and the mixture turned yellow. This pre-activated mixture of carboxylate was then added to the trityl resin, the vessel sealed and stirred end-over-end for 16 hours. After this time the solvent was removed by filtration, the resin washed with DMF (3 x 6 mL), DCM (3 x 6 mL) and diethyl ether (3 x 6 mL). The resin was dried by flushing excess solvent from the vessel using a syringe.

To the coupled resin was added a freshly mixed DMF/piperidine mixture (3 mL, 4:1 DMF:piperidine). The reaction vessel was sealed and stirred end-over-end for 15 minutes. After this time the solvent was removed by filtration, the resin washed with DMF (3 x 6 mL), DCM (3 x 6 mL) and diethyl ether (3 x 6 mL) and dried by flushing as described previously.

Sequences of coupling and deprotection was repeated in this way using the following amino acids: 1) Fmoc-*para*-aminomethyl benzoic acid (187 mg, 0.500 mmol); 2) Fmoc-(L)-Pro (169 mg, 0.500 mmol); 3) Fmoc-(L)-Pro (169 mg, 0.500 mmol); 4) Fmoc-(L)-(*N*-Me)Val

(177 mg, 0.500 mmol); 5) Fmoc-(L)-Val (170 mg, 0.500 mmol) [N.B. this coupling procedure was repeated twice before deprotection, to ensure that the *N*-methyl valine had successfully coupled]; 6) *N*Me$_2$-(L)-Val (73 mg, 0.500 mmol). Couplings were conducted for two hours, deprotections for 15-20 minutes. After each step the resin was washed sequentially with DMF (3 x 6 mL), DCM (3 x 6 mL) and diethyl ether (3 x 6 mL). When it was necessary to leave the peptide overnight it was stored at -20 °C in an Fmoc-protected state.

Cleavage from resin

The resin-bound peptide was treated with a solution of hexfluoroisopropanol in DCM (1:4 mixture, 4 mL) for 30 minutes with end-over-end stirring. The solvent containing free peptide was removed by filtration and the resin washed with DCM (2 x 5 mL). The combined organics were concentrated *in vacuo* to afford a pale yellow oil (70 mg, 44%). Mass spectrometric analysis indicated the presence of the expected peptide (*m/z* 749; M·Na$^+$). The cleavage process was repeated once to afford a further 17% of the desired peptide.

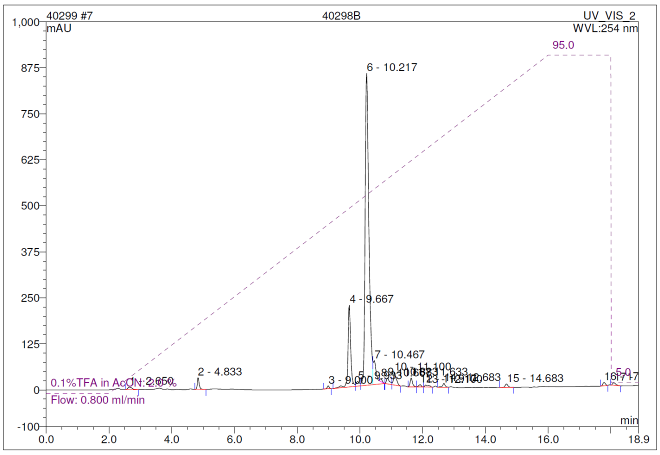

*Coupling to strained alkyne to give **8b***

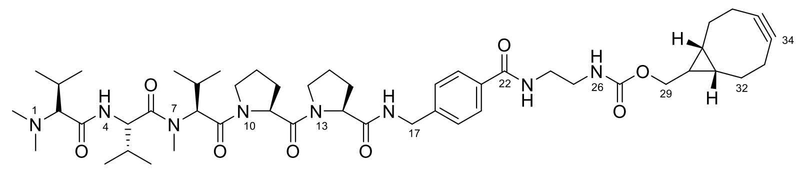

A portion of peptide (2.5 mg, 3.44 μmol) was dissolved in DCM (100 μL). Strained activated succinyl ester alkyne derivative (SXA-1028, 2.0 mg, 6.88 μmol) was added followed by trimethylamine (5 μL). The mixture was stirred for 1.5 hours at RT. Analysis by HPLC indicated formation of a major product. Mass spectrometric analysis (Water open access) indicated the presence of the expected product (*m/z* 904, $C_{49}H_{75}N_8O_8Na$ M·H$^+$ 904). The reaction mixture was analysed by HPLC using a dionex 3000 HPLC system connected to a Jupiter 4 μ Proteo 4.6 x 250 mm 4 micron analytical column equipped with a guard column (both from Phenomenex). Solvent A was H_2O containing 0.1 % formic acid. Solvent B was

MeCN containing 0.1% formic acid. The sample was eluted from the column using the following gradient: 2 minutes equilibration at 5 % B, a gradient between 2 and 18 minutes from 5-95% B, a wash at 95 % B from 18 to 22 minutes, followed by re-equilibration of the column at 5% B. Detection of compounds was achieved by UV absorbance at 214, 254, 280 and 300 nm. The absorbance at 254 nm was the most informative. Samples were filtered (20 μm centrifugal spin filter) prior to analysis.

δ_H (500 MHz, C_6-DMSO) 8.43 (1H, t, *J* 5.3 Hz, NH-23/26), 8.34 (1H, t, *J* 6.0 Hz, NH-16), 8.08 (1H, d, *J* 8.3 Hz, NH-4), 7.77 (2H, d, *J* 8.1 Hz, 2 x Ar-H), 7.30 (2H, d, *J* 8.2 Hz, 2 x Ar-H), 7.21 (1H, t, *J* 5.6 Hz, NH-23/26), 4.98 (1H, d, *J* 10.9 Hz, H-2/8), 4.54 (1H, dd, *J* 5.3, 8.2 Hz, H-11/14), 4.512 (1H, t, *J* 8.7 Hz, H-5), 4.30 (dABq, *J* 6.0 Hz, 15.9 Hz, H-17), 4.33 (1H, dd, *J* 4.1, 8.6 Hz, H-11/14), 4.04 (1H, d, *J* 8.0 Hz, H-29),3.79-3.51 (4H, m, 2 x proline-NCH_2), 3.30 (2H, dd, *J* 6.1, 12.0 Hz, H-24/25), 3.15 (2H, dd, *J* 6.2, 12.3 Hz, H-24/25), 3.07 (3H, s, N-7 CH_3), 2.64 (1H, d, *J* 10 Hz, H-2/8),2.22-2.05 (9H, m, CH(valine), 2 x H-32', 2 x H-33, 2 x H-33', 2 x CH_2 (proline)), 2.20 (3H, s, N-1 CH_3), 2.00-1.87 (5H, m, 2 x CH(valine), 3 x CH_2 (proline)), 1.83-1.77 (2H, m, 2 x CH_2 (proline)), 1.76-1.70 (1H, m, 1 x CH_2 (proline)), 1.51 (2H, dd, *J* 12.0 Hz, 19.1 Hz, 2 x H-32), 1.27 (1H, apparent pentet, *J* 8.6 Hz, H-30), 0.93 (3H, d, *J* 6.7 Hz, CH_3), 0.88-0.86 (8H, m, X x H-31, 2 x CH_3), 0.82 (3H, d, *J* 6.7 Hz, CH_3), 0.70 (3H, t, *J* 6.1 Hz, CH_3); δ_C (125.8 MHz, C_6-DMSO) 173.2, 172.2, 170.5, 170.3, 168.2, 166.6 164.0 (7 x s, 7 x C=O), 143.3, 133.3 (2 x s, 2 x Ar-C), 127.6, 127.1 (2 x d, 2 x Ar-C), 99.5 (s, C-34), 73.2 (d, C-2/8), 61.9 (t, C-29), 60.0 (d, C-11/14), 58.7 (d, C-2/8), 58.0 (d, C-11/14), 54.4 (d, C-5), 47.6, 47.1 (2 x t, 2 x proline-NCH_2), 42.1 (t, C-17), 41.8 (q, N-1 CH_3), 40.6, 40.4 (2 x t, C-24, C-25), 30.7 (q, N-7 CH_3), 30.3 (d, valine-CH), 29.6 (t, proline-CH_2), 29.0 (t, C-32), 28.5 (t, proline-CH_2), 27.3, 27.1 (2 x d, 2 x valine-CH), 25.1, 24.8 (2 x t, 2 x proline-CH_2), 21.3 (t, C-33), 20.1, 20.0, 19.7, 19.2, 18.6, 18.1 (6 x q, 6 x CH_3), 19.5 (2 x t, 2 x C-31); HRMS calculated for $C_{49}H_{75}O_8N_8$ ($M{\cdot}H^+$) 903.57133, observed 903.56989; ion series calculated: 903.57133, 904.57369, 905.57804, 906.58140, 907.58476; observed: 903.56989, 904.57303, 905.57653, 906.57972, 907.58397.

This reaction was repeated on 21 mg scale to afford the target peptide **8b** after purification by semi-prep HPLC. HPLC was carried out as described above except that the column used was a Jupiter Proteo (Phenomenex) 10 x 250 mm. Samples containing product were combined, concentrated *in vacuo* and lyophilised to afford **8b** as an off-white solid (9.8 mg, 38% overall yield).

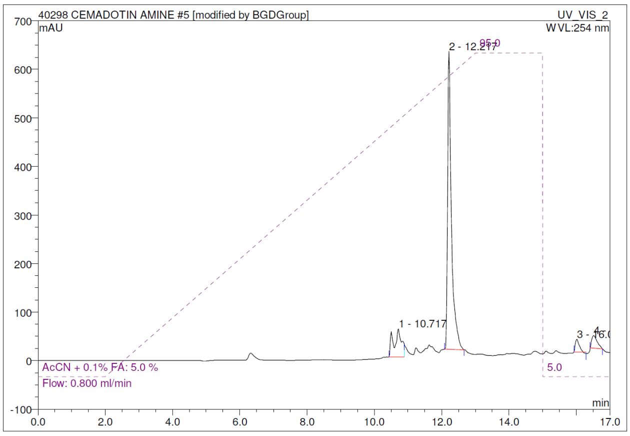

3.1 Antibody remodelling

3.1.1 General methods: biological

Sodium dodecyl sulfate – polyacrylamide gel electrophoresis (SDS-PAGE)

Aliquots (1-2 µg of antibody, typically 1 µL) were removed from the reaction mixture, diluted with water (typically 4 µL), snap frozen and stored at -20 ºC until analysis by SDS-PAGE. Upon thawing, aliquots were treated promptly with an equal volume (typically 5 µL) of SDS loading buffer (80 mM Tris-HCl buffer pH 6.8, 8% v/v glycerol, 40 µM bromophenol blue, 80 mM SDS, 2% β-mercaptoethanol). The solution was heated to 95 ºC for 3 min and then loaded into a well in a 4-12% bis-tris polyacrylamide gel. The gel was run at 200 V in MOPS running buffer for around 50 minutes. Visualisation was by the addition of Coomassie Instant Blue dye (Gentaur) and gentle stirring for 2-24 h at room temperature. Gels were de-stained by gentle stirring in water for 4-24 hours at room temperature.

To improve the separation of the heavy chain components carrying only GlcNAc-Fuc from those which had undergone glycosylation, gels were over-run by around 15 minutes (around 1 hour 5 minutes).

LC-MS

Aliquots (2 µg of antibody, typically 1 µL) were removed from the reaction mixture, diluted with water (typically 9 µL) and treated with DTT (typically 1 µL of an 80 mM solution). The mixture was heated to 60 ºC for three minutes then further diluted to a final antibody concentration of around 0.02 mg/mL before analysis by LC-MS.

LC-MS was carried out using one of two methods.

Method A

Protein samples were desalted by passing through a Chromolith RP-18e 5-2 mm HPLC guard cartridge (Merck) attached to a Waters 1525µ binary HPLC pump. Samples were eluted using buffers A (0.1% aqueous solution of formic acid) and B (acetonitrile). Prior to sample injection, the column was pre-treated as follows: 1 min 40% A, 0.6 mL/min followed by 20 seconds at 95% A, 0.4 mL/min followed by 40 seconds 95% A, 0.3 mL/min. Samples (5 µL) were injected onto the column *via* a Waters 2777C sample manager, and were eluted using a solvent gradient as follows: 30 seconds of 95% buffer A (flow rate 0.3 mL/min) followed by a 1 minute gradient to 40% buffer A (flow rate 0.3 mL/min) and hold at 40% A for a further 1.5 minutes. The flow rate was then increased to 0.75 mL/min for 30 seconds before a final wash using 2% A for 30 seconds. Eluted samples were injected into an LCT Premier XE mass spectrometer (Waters) operating in positive mode (ES^+) with the analyser in 'V' mode. The capillary was set at 3000V and sample cone at 100V. Desolvation temperature was 150 ºC. Collected data was analysed using MassLynx V4.1 software. Protein eluted between 2.0 and 2.5 minutes, and all protein products were integrated for analysis together. Ion envelopes were deconvoluted using MaxEnt software, using the uniform Gaussian model with

appropriate values for the peak width at half height (typically between 0.5 and 0.8 Da). Resolution was set to 0.5 Da. Minimum intensity ratios were set at 33% for both left and right. Deconvolution iterations were allowed to continue until convergance. Raw data was not subjected to subtraction, smoothing or centring prior to deconvolution.

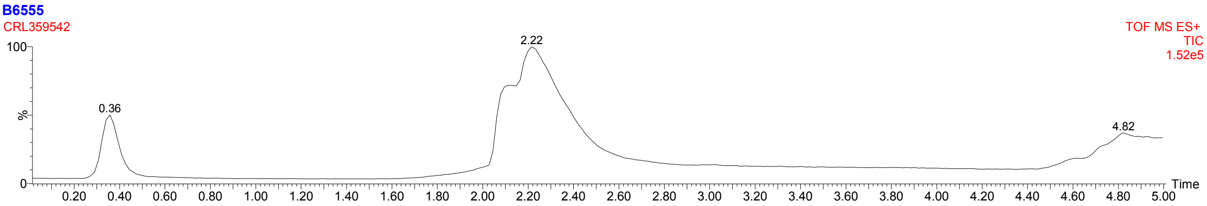

Typical chromatogram obtained after LC-MS (method A) of denatured and reduced IgG. Protein elutes between 2.0 and 2.5 minutes and the entire peak was integrated to give a mass spectrum containing all protein components in the mixture.

Method B

Protein samples were desalted by passing through a ProSwift RP-2H analytical 4.6 x 50 mm HPLC column (Thermo scientific) attached to a Shimadzu Prominence LC-20ad HPLC pump equipped with a Prominence DGU-20A5 degasser. Samples were eluted using buffers A (0.1% solution of formic acid in 95:5 water:acetonitrile) and B (0.1% solution of formic acid in 95:5 acetonitrile:water). Samples (3-15 μL) were injected onto the column *via* a Shimadzu Prominence SLI-20AC autosampler, and were eluted using a solvent gradient as follows: 1 minute of 100% buffer A followed by a 6 minute gradient to 100% buffer B, held at 100% B for 4 minutes. The flow was adjusted to 100% A and the column re-equilibrated with 100% A for 5 minutes. The flow rate was maintained at 0.4 mL/min throughout. Protein eluted at around 6.5 minutes and all protein products were integrated together. Eluted samples were injected into an LCT (micromass) mass spectrometer operating in positive mode (ES^+) with the analyser in ‘V’ mode. The capillary was set at 3000V and sample cone at 25V. Desolvation temperature was 400 ºC. Collected data was analysed as described above.

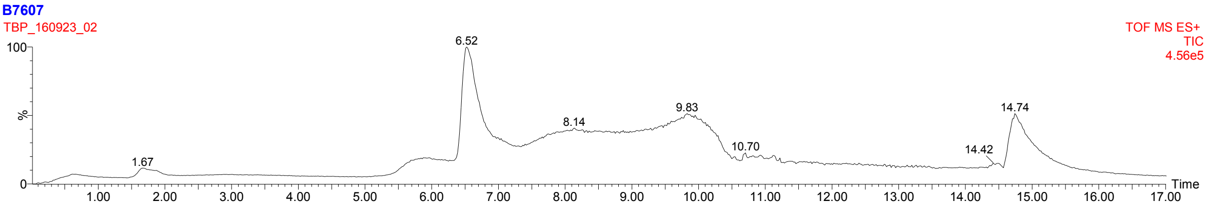

Typical chromatogram obtained after LC-MS (method B) of denatured and reduced IgG. Protein elutes between 6.5 and 6.8 minutes and the entire peak was integrated to give a mass spectrum containing all protein components in the mixture.

*3.1.2 Trimming of Herceptin to produce **1***

Herceptin (2.08 mL of an 8 mg/mL solution in PBS buffer; 16.6 mg total) was treated with WT EndoS_GST (14 μL of a 14.7 mg/mL solution in PBS). The mixture was incubated at rt.

An aliquot (10 μL) was taken after 4 h and analysed by SDS-PAGE under reducing conditions to confirm completion of reaction. The trimmed antibody was purified using four Protein A plus spin column (7 mg column binding capacity, Thermofisher) and gentle Ag/Ab binding and elution buffers. The columns were allowed to warm to rt and centrifuged (5000 x g, 1 min) before equilibration with binding buffer (2 x 400 μL, centrifuge 5000 x g, 1 min). The sample was applied (3 x 180 μL plus 20 μL wash) and the column incubated at rt for 15 min with end-over-end mixing. Centrifuge (5000 x g, 1 min), collect flow-through fractions (these can be re-applied to the column to recover any unbound IgG). The columns were washed with binding buffer (400 μL) and centrifuged (5000 x g, 1 min). This process was repeated a total of three times and the flow-through discarded. The columns were then eluted with elution buffer (400 μL) and centrifuged (5000 x g, 1 min) a total of six times and the flow-through analysed by nanodrop to confirm the quantity of protein present.

Fractions containing protein were pooled, subject to dialysis (snakeskin dialysis tubing) against TBS (2 L, 16 h) and PBS (2 x 2 L, 4 h), and concentrated (vivaspin 20, MWCO 30,000) to ~700 μL. To ensure complete removal of residual WT Endo S the product was subjected to a second round of purification as described above. Following dialysis and concentration **1** was obtained as a solution in PBS (1.76 mL, 8.5 mg/mL, 90%).

3.1.3 Transglycosylation activity of EndoS mutants

Typical glycosylation reaction protocol

Trimmed Herceptin **1** (35 μg in PBS buffer) and decasaccharide oxazoline (3.5 μL of a 10 mg/mL aqueous solution) were combined in sodium phosphate buffer (3.5 μL of a 500 mM solution, pH 6.5 final concentration 100 mM). EndoS D233Q (0.70 μL, of a 1.0 mg/mL solution in PBS buffer, 50:1 Ab:enzyme) was added. The mixture was vortexed, incubated at 30 °C in a water bath and aliquots (1 μL) taken for analysis at regular time intervals.

Similar reactions were conducted using the various Endo S mutants. Initial screening for transglycosylation activity ('T' in manuscript) was carried out using densitometry to semi-quantify level of antibody glycosylation. Densitometry analysis for glycosylation was more accurate than for hydrolysis, as the larger glycans attached in the product heavy chain enabled better band separation. However, separation is still not at the baseline level and so quantification is approximate.

Due to the high hydrolytic activity of the Y305F variant, this mutant was not included in our transglycosylation activity screening.

Endo S D233Q showed high transglycosylation activity, though at low enzyme loadings a small amount of over-reaction was apparent as a faint band at slightly higher MW than the heavy chain (figure S5). This did not become significant until 4 hours reaction time, giving a target maximum reaction time beyond which we would expect glycation to compete significantly. Under these conditions glycosylation levels reached a maximal level around 80% after 2 hours.

A)

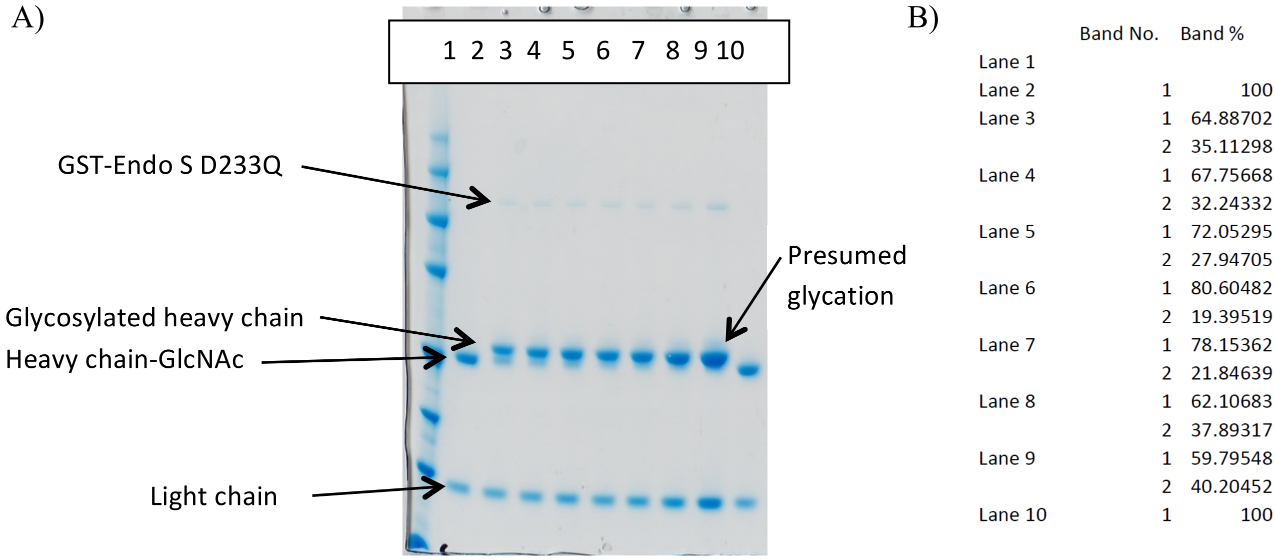

B)

	Band No.	Band %
Lane 1		
Lane 2	1	100
Lane 3	1	64.88702
	2	35.11298
Lane 4	1	67.75668
	2	32.24332
Lane 5	1	72.05295
	2	27.94705
Lane 6	1	80.60482
	2	19.39519
Lane 7	1	78.15362
	2	21.84639
Lane 8	1	62.10683
	2	37.89317
Lane 9	1	59.79548
	2	40.20452
Lane 10	1	100

Figure S5. A) SDS-PAGE analysis and B) densitometry data for glycosylation catalysed by Endo S D233Q. Lane 1: Marker. Lanes 2,10: trimmed Herceptin **1**. Lanes 3-9: glycosylation after 0.5, 1, 1.5, 2, 3, 4, 5 h.

Endo S D233E showed very fast transglycosylation and hydrolytic activity: analysis of the glycosylation reaction at 5 minute intervals indicated that hydrolysis rapidly competes with transglycosylation (figure S6A). Maximal conversion of 75% was observed after 20 mins, after which time product was rapidly hydrolysed. This rapid competing hydrolysis was expected to render purification of homogeneous glycosylated antibody difficult, as the standard purification protocols (e.g. affinity chromatography using Protein A or Protein G) require a minimum of 20 minutes between column loading and product elution. During this time the product would still be exposed to Endo S so we would anticipate considerable loss of target during the purification process.

A)

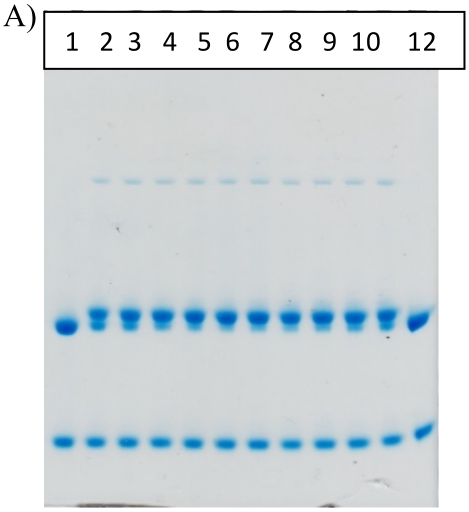

B)

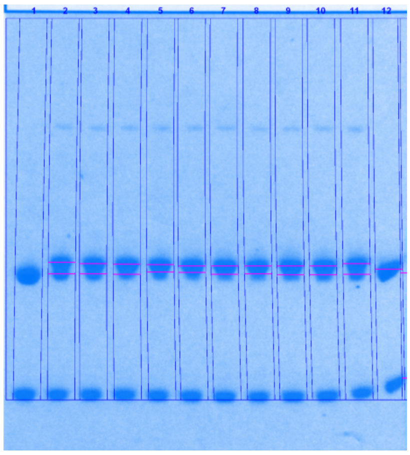

	Band No.	Band %
Lane 1		
Lane 2	1	55.86697
	2	44.13303
Lane 3	1	64.84311
	2	35.15689
Lane 4	1	72.37322
	2	27.62678
Lane 5	1	77.31252
	2	22.68748
Lane 6	1	74.66104
	2	25.33896
Lane 7	1	73.19917
	2	26.80083
Lane 8	1	70.01058
	2	29.98942
Lane 9	1	68.90595
	2	31.09405
Lane 10	1	65.05108
	2	34.94893
Lane 11	1	65.38056
	2	34.61944
Lane 12	1	100

Figure S6. A) SDS-PAGE analysis and B) densitometry data for glycosylation catalysed by Endo S D233E. Lanes 1,12: trimmed Herceptin **1**. Lanes 2-11: glycosylation after 5, 10, 15, 20, 30, 60, 90, 120, 180, 240 mins.

Endo S D233A showed low transglycosylation activity (figure S7A), reaching a maximum

conversion of 35% after 30 mins. Endo S D233A:Q303E showed modest activity, reaching around 65% conversion but only after 5 hours (figure S7B). Due to the requirement for relatively rapid reaction in order to minimise the competing glycation process, these two variants were deemed less appropriate for further method development than the D233Q mutant.

Taking the optimal transglycosylation conversion obtained in the initial screening trials as ‘T’, we obtained values as follows:

D233Q: 80% (2 hours)
D233A: 35% (30 mins)
D233A:Q303E: 65% (5 hours)
D233E: 75% (20 mins)
WT: 75% (30 min), 10% (2 hours)

And using these values along with those determined previously for relative hydrolytic activity (‘H’), we obtain the following ratios of activities:

D233Q: 80:20
D233E: 75:55
D233A:E303Q: 65:25
D233A: 35:30
WT: 10:100
Y305F: nd:100 (transglycosylation activity not determined).

These offer a semi-quantitative evaluation of the relative hydrolytic and transglycosylation activities of the various mutants. From these values it appears that the EndoS D233Q variant is the most promising, with the highest T:H ratio, and so was selected for further method optimisation studies.

A)

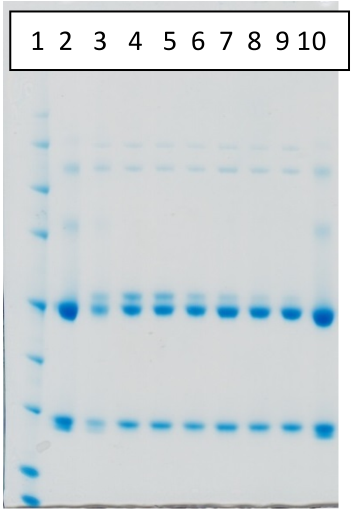

	Band No.	Band %
Lane 1		
Lane 2		
Lane 3	1	23.71646
	2	76.28354
Lane 4	1	33.35555
	2	66.64445
Lane 5	1	28.23059
	2	71.76942
Lane 6	1	18.84817
	2	81.15183
Lane 7	1	15.00711
	2	84.99289
Lane 8	1	16.8207
	2	83.1793
Lane 9	1	13.79062
	2	86.20938

B)

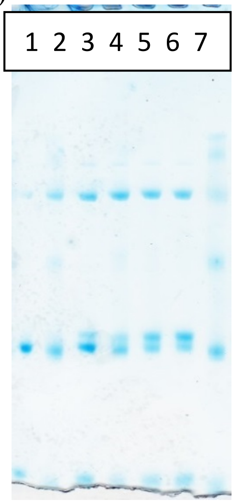

	Band No.	Band %
Lane 1		
Lane 2	1	100
Lane 3	1	17.34792
	2	82.65208
Lane 4	1	34.82855
	2	65.17145
Lane 5	1	58.12678
	2	41.87322
Lane 6	1	64.74765
	2	35.25235
Lane 7	1	100

Figure S7. A) SDS-PAGE analysis and densitometry data for glycosylation catalysed by Endo S D233A; lane 1: marker Lanes 2 and 10: trimmed Herceptin **1**. Lanes 3-9: glycosylation reaction progress after 15, 30, 45, 90, 135, 180, 240 mins. B) SDS-PAGE analysis & densitometry data for glycosylation catalysed by Endo S D233A:Q303E; lane 1: Herceptin. Lanes 2, 7: trimmed Herceptin **1**. Lanes 3-6: glycosylation after 1, 2, 3, 5 h.

Having established that EndoS D233Q was the preferred variant for further development, we undertook initial optimisation of the glycosylation conditions. We maintained pH at 6.5 and

temperature at 30 °C, increased enzyme concentration to 5% loading (20:1 wt:wt Ab:enzyme) and used two additions of 70 equivalents of oxazoline donor, added at 40 minute intervals. Under these conditions the glycosylation reaction appeared to proceed to completion around 90 mins to 2 hours after the second addition of donor. Analysis by LC-MS indicated that product distribution remained 'stable' for a sufficient period to enable purification by Protein A affinity chromatography without appreciable product hydrolysis (figure S9).

3.1.4 LC-MS analysis of commercial, trimmed and remodelled Herceptin

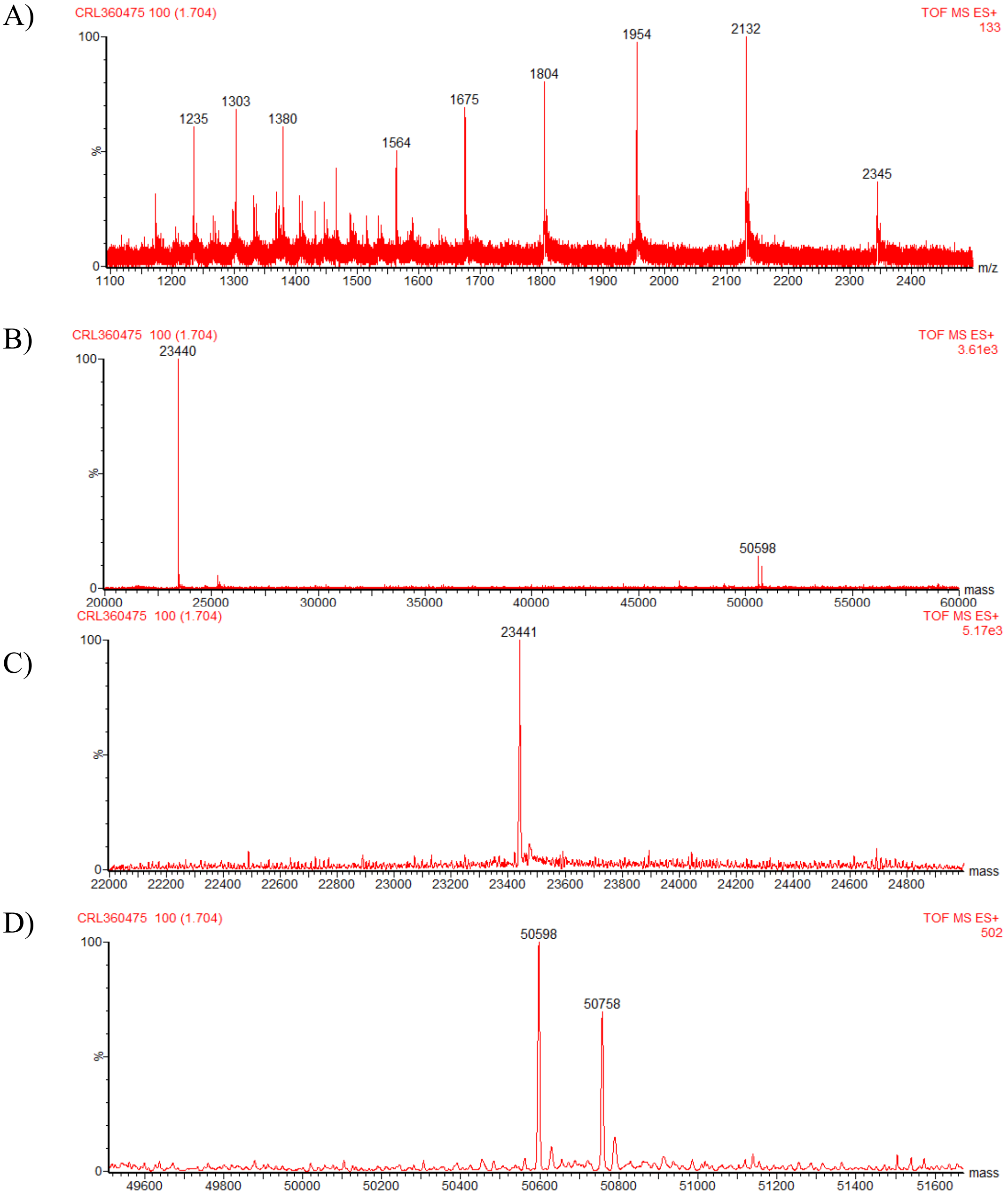

Figure S8. A) Raw and B) deconvoluted LC-MS data for commercial Herceptin along with magnifications of C) region of the spectrum corresponding to light chain and D) region of spectrum corresponding to heavy chain. Calculated mass of light chain: 23443, observed: 23441. Calculated mass of heavy chain with G0F glycan: 50600, observed 50598. Calculated mass of heavy chain with G1F glycan: 50762, observed 50758.

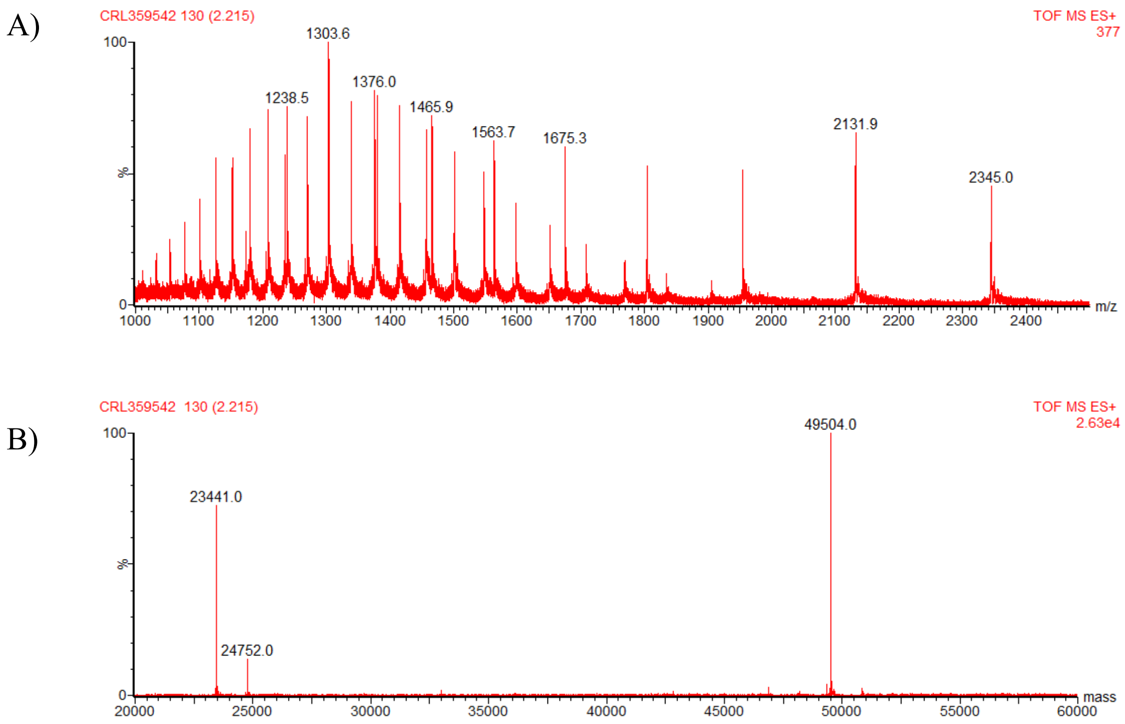

Figure S9. A) Raw and B) deconvoluted LC-MS data for trimmed Herceptin **1**. Calculated mass of light chain: 23443, observed: 23441. Calculated mass of heavy chain 49505, observed 49504. N.B. The peak at 24752 is half the mass of the heavy chain and is a consequence of the deconvolution process.

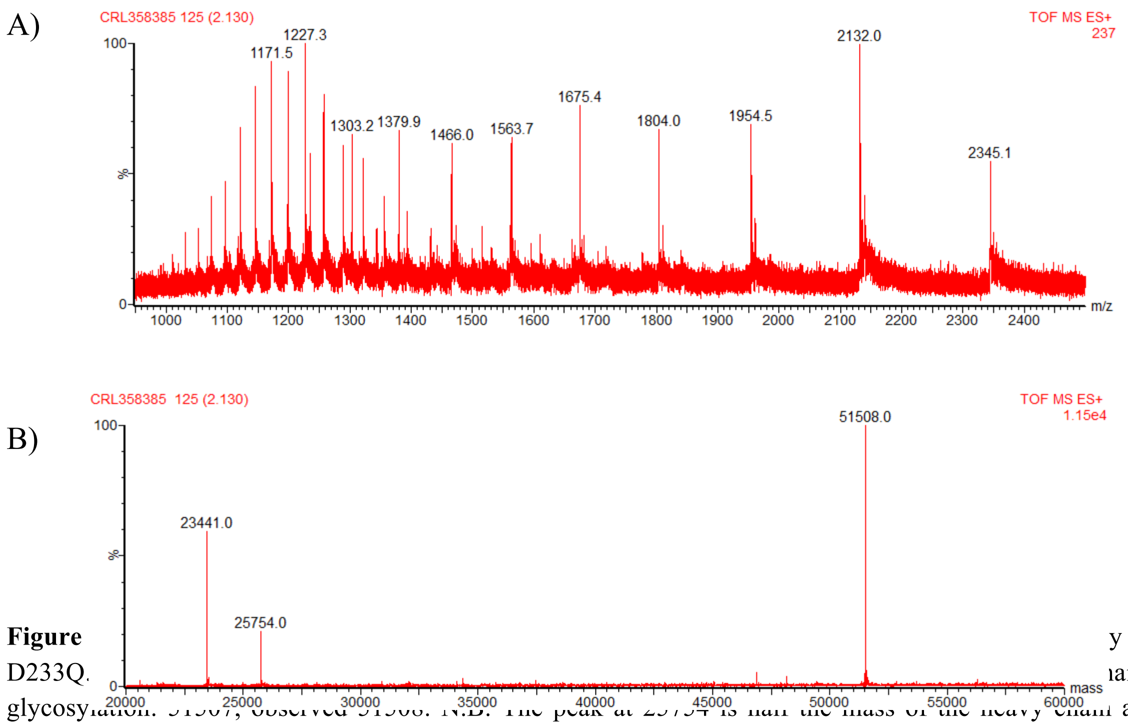

Figure [illegible] y Endo S D233Q. [illegible] ain after glycosy[illegible] and is a consequence of the deconvolution process.

3.2 Analysis of antibodies under native/non-denaturing MS conditions

3.2.1 Sample preparation

Commercial Herceptin was dialysed against ammonium bicarbonate (50 mM, pH 8.0) to remove stabilisers (histidine, polysorbate 20 and trehalose).

Trimmed Herceptin **1** was purified twice by Protein A affinity chromatography (as described above) and then buffer exchanged into either ammonium acetate (150 mM, pH 8.0) or ammonium bicarbonate (50 mM, pH 8.0). Buffer exchange was carried out either by exhaustive dialysis or using Micro BioSpin 6 (BioRad) columns. Two rounds of buffer exchange were required to remove non-volatile salts for native/non-denatured MS analysis.

Glycosylation reactions and PNGase F deglycosylation reactions were purified using Protein G columns according to the manufacturer's protocol, with elution of antibody using pH 2.7 buffer which was neutralised immediately using 1 M Tris-HCl, pH 8.5. The fractions containing protein were dialysed (slide-a-lyser, 10 kDa MWCO) twice against 50 mM ammonium bicarbonate, pH 8.0 (1 L) and subsequently concentrated (vivaspin 500, 30 kDa MWCO) to afford a solution of antibody at a concentration between 0.2 and 2 mg/mL (1-15 µM).

Prior to intact mass spectrometric analysis, all protein samples were adjusted to between 1.5 and 3 µM final concentration in 50 mM ammonium bicarbonate or 50 mM ammonium acetate, pH 8.0.

For analysis under reducing conditions, samples were first treated with DTT (8 mM) at 60 °C for three minutes before buffer exchange into 50 mM ammonium acetate, pH 8.0, using Biorad Micro BioSpin 6 columns. Buffer exchange was carried out according to the manufacturer's protocol, and each sample was subjected to two rounds of buffer exchange to ensure complete exchange.

Samples for analysis under reducing and denaturing conditions were treated with DTT as described above. They were subsequently purified by C_4 ZipTip and transferred into a spray solution comprising methanol/water/formic acid in the ratio 75/25/3. The ZipTip was washed with methanol (10 x 10 µL) and then with water containing 0.1% formic acid (10 x 10 µL). The sample was loaded onto the C_4 phase by aspirating around 20 times, followed by removal of the solvent. Salts and highly polar components were removed by washing with water containing 0.1% formic acid (10 x 10 µL). The protein was eluted by aspirating into the spray solution (10 µL) several times.

3.2.2 Sample analysis

Samples were prepared as described above and introduced into a Q Exactive^{TM} hybrid quadrupole-Orbitrap^{TM} mass spectrometer modified for the transmission and detection of high mass ions and operated in "Native Mode".[8] Briefly, ions were generated in the positive ion mode from a static nanospray source using gold-coated capillaries prepared in-house,

then passed through a temperature controlled transfer tube (40-60 C), RF-lens, injection flatapole and bent flatapole. After traversing the selection quadrupole, which was operated with a wide selection window (2,000-15,000 *m/z*), ions were trapped in the HCD cell before being transferred into the C-trap and Orbitrap mass analyser for detection. Transient times were 64 ms and AGC target was 1×10^6. Spectra were acquired with 10 microscans, averaged over 50-100 scans and with a noise level parameter set to 3, slightly lower than the default setting of 4.68. Efficient desolvation of intact antibodies was achieved through increased voltages applied in the HCD cell (150-200 V). The collision gas was either Nitrogen or Argon and pressure in the cell was maintained at around 1×10^{-9} mbar. Data was processed using Thermo Scientific™ Xcalibur™ 2.1 and masses calculated using in-house software.

Proportions of the components in the mixtures were calculated by summing the intensities of the three most abundant charge states for each component. These were then divided by the sum of all the intensities for the three major peaks for all the series combined.

Commercial Herceptin analysed under LC-MS conditions showed only two major glycoform components: G0F and G1F (figure S8). However, under high resolution Orbitrap nMS analysis, at least seven glycoforms could be resolved (figure S11). The difference in sensitivity of the techniques is presumed to be largely due to the great improvement in signal:noise ratio when analysing samples under native conditions. This arises due to the much smaller number of charge states produced under native conditions.[9]

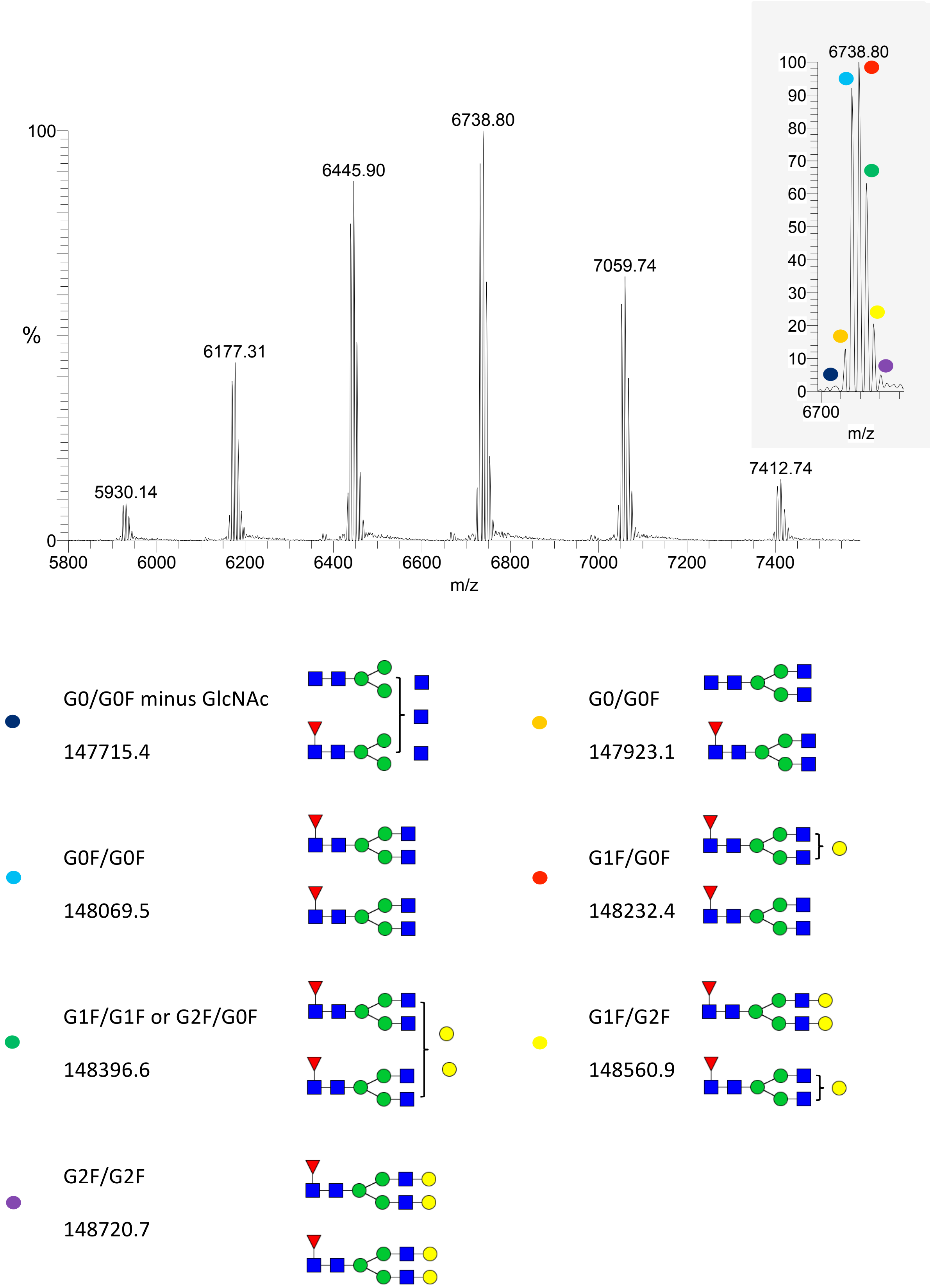

Figure S11. Native mass spectrum of commercial Herceptin. Inset is a close-up of the base peak (+22 charge state). Major glycoforms and intact mass corresponding to these are shown below the spectra. Commercial Herceptin was found to comprise at least seven major glycoforms, with the dominant components being the G0F and G1F.

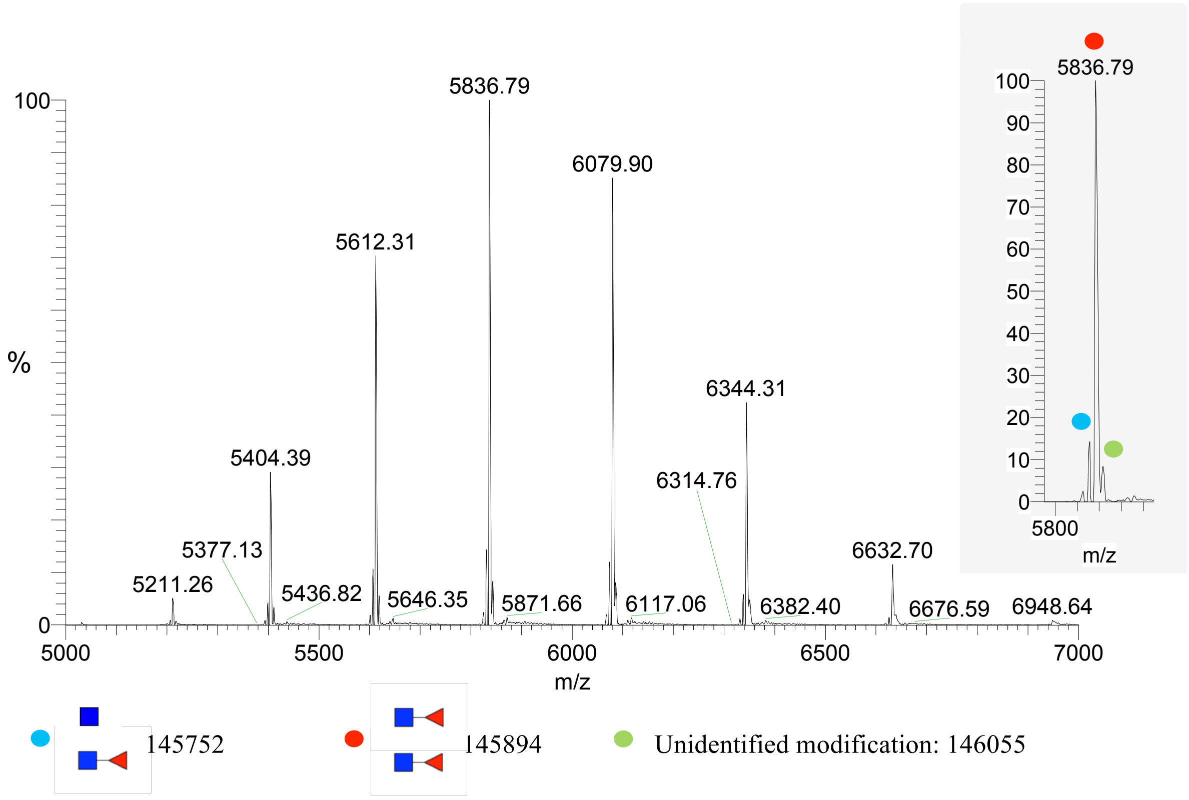

Figure S12. Native mass spectrum of trimmed Herceptin **1**. Inset is a close-up of the base peak (+25 charge state). Major glycoforms are indicated below the spectra.

Following Endo S catalyzed trimming of the Herceptin glycans, three major components remained. The primary component was the expected bis-fucosylated material, comprising around 85% of the material. Also present was around 10% mono-fucosylated material. We also identified a minor component (around 5%) which appeared to have a mass around 160 Da heavier than the expected mass for each species. We have not identified this minor component but it may be due to incomplete lysine trimming of the heavy chain, another modification of the protein backbone or possibly low levels of *O*-glycosylation in the hinge region of the antibody.

The levels of core fucosylation and of the minor component corresponding to +160 Da are not affected by Endo S treatment of antibody samples. Therefore during all glycoprotein remodeling work described here it is not possible to obtain 100% pure Herceptin products: all constructs obtained are expected to retain around 10% monofucosylation and around 5% of a +160 Da component.

Glycosylation of **1** using one addition of seventy equivalents of donor **2** at pH 6.5, with 10 % wt/wt enzyme loading gave rise to a mixture of products corresponding to attachment of between 0 and 3 glycans to the antibody (figure S13A). The use of two additions of seventy equivalents of donor gave a considerably cleaner product mixture (figure S13B).

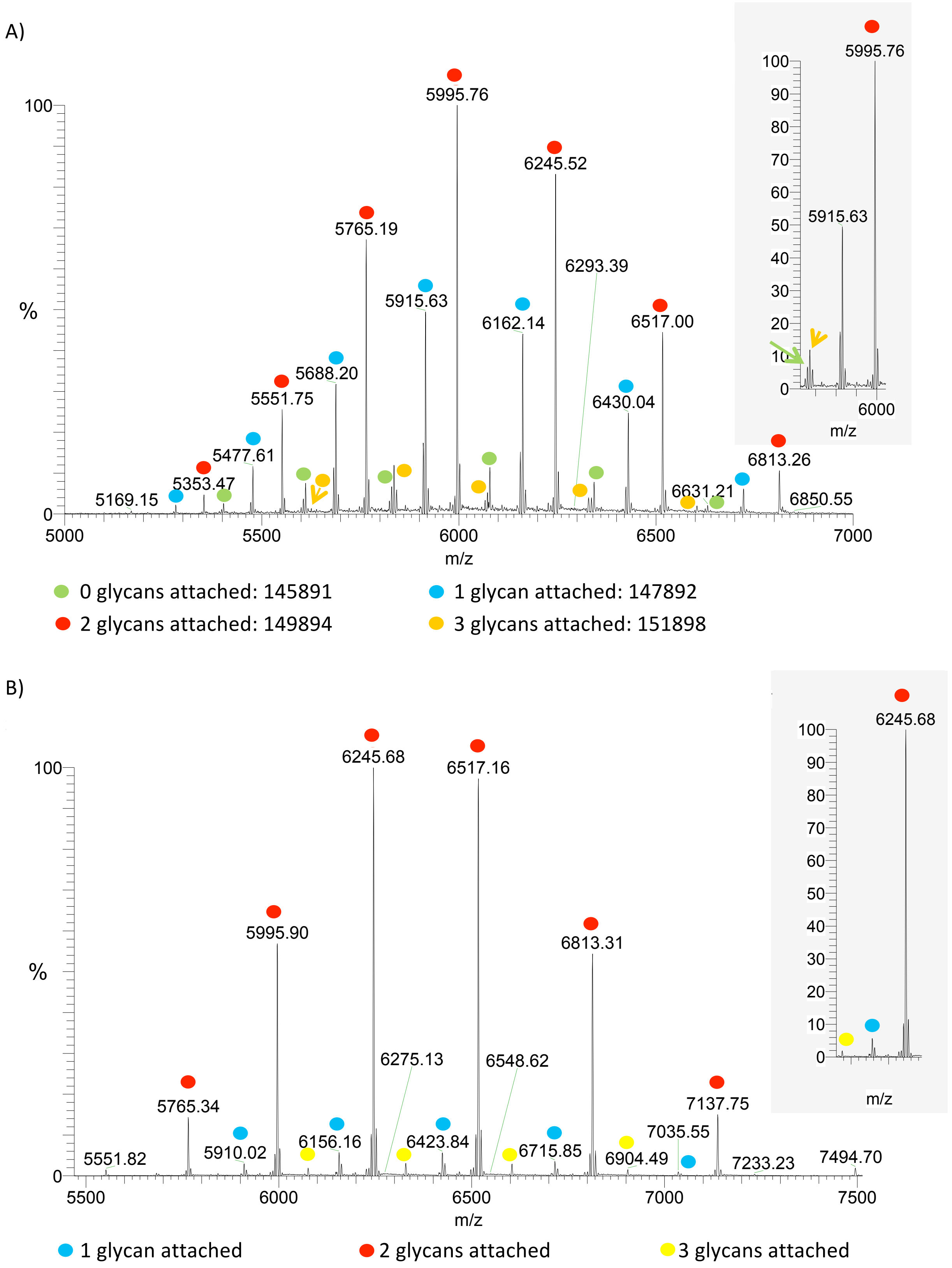

Figure S13. Native mass spectra of glycosylation of **1** using oxazoline **2** with inset close-up of the base peak. A) Seventy equivalents of donor **2** gave rise to considerable heterogeneity in the product (+25 charge state). B) Use of two additions of seventy equivalents of donor **2** afforded a much cleaner product distribution (+24 charge state).

Analysis of the glycosylation reaction conducted using two additions of seventy equivalents of donor **2** indicated that the bisglycosylated product comprised around 89% of the mixture, with the monoglycosylated 7% and the product carrying three glycans around 5% (figure S13B). Within these sets, monofucosylation levels varied somewhat, suggesting that the absence of a core fucose affects the activity of Endo S. In Endo S treated Herceptin **1** around 12% of the antibody carries only one fucose, 2% has no fucose and 7% has a mass around 180 Da higher. The remaining 79% was the expected trimmed material carrying two GlcNAc(Fuc) moieties. Following glycosylation, native MS indicated that 5-10% of the diglycosylated product possessed only one core fucose residue. However, the monoglycosylated material appears to comprise an approximately equal mixture of monofucosylated and difucosylated material.

3.3 PNGase F treatment of Herceptin

3.3.1 Typical procedure for PNGase F digestion of antibodies

A sample of antibody (40 μL of a 0.5 mg/mL solution) was dialysed into ammonium bicarbonate if necessary (50 mM, pH 8.0; slide-a-lyser dialysis cup, 20 kDa MWCO, 3 x 2 h) and then treated with PNGase F (0.2 μL, 500, 000 units/mL, 100 units) at 37 °C. After incubation for 16 hours the reaction was analysed by LC-MS to confirm that reaction was complete. The reaction mixture was subjected to purification by Protein G affinity chromatography using a Spin Column (Pierce scientific) according to the manufacturer's protocol. The fractions containing antibody were combined and dialysed into ammonium bicarbonate (50 mM, pH 8.0; slide-a-lyser, 20 kDa MWCO, 3 x 2 h). The resultant solution was concentrated to between 0.2 and 2 mg/mL using a centrifugal concentrator (vivaspin 500, 30 kDa MWCO). Concentrations were adjusted to between 0.4 and 0.5 mg/mL prior to analysis by nMS.

3.3.2 Deglycosylated herceptin

PNGase F digestion of Herceptin proceeded efficiently at 37 °C: LC-MS under reducing conditions indicated that removal of all sugars was complete after 16-24 hours. The antibody product was purified by Protein G, buffer exchanged into ammonium acetate (50 mM, pH 8) and analysed by LC-MS and nMS (figure S14). Native MS indicated that the minor peaks which we had assigned as being due to core afucosylated glycans were no longer present, supporting our hypothetical assignment. Furthermore, the minor impurity at around +176 Da was still present in the spectrum, indicating that this heterogeneity was not due to a minor glycoform but instead must be an alteration elsewhere in the protein sequence.

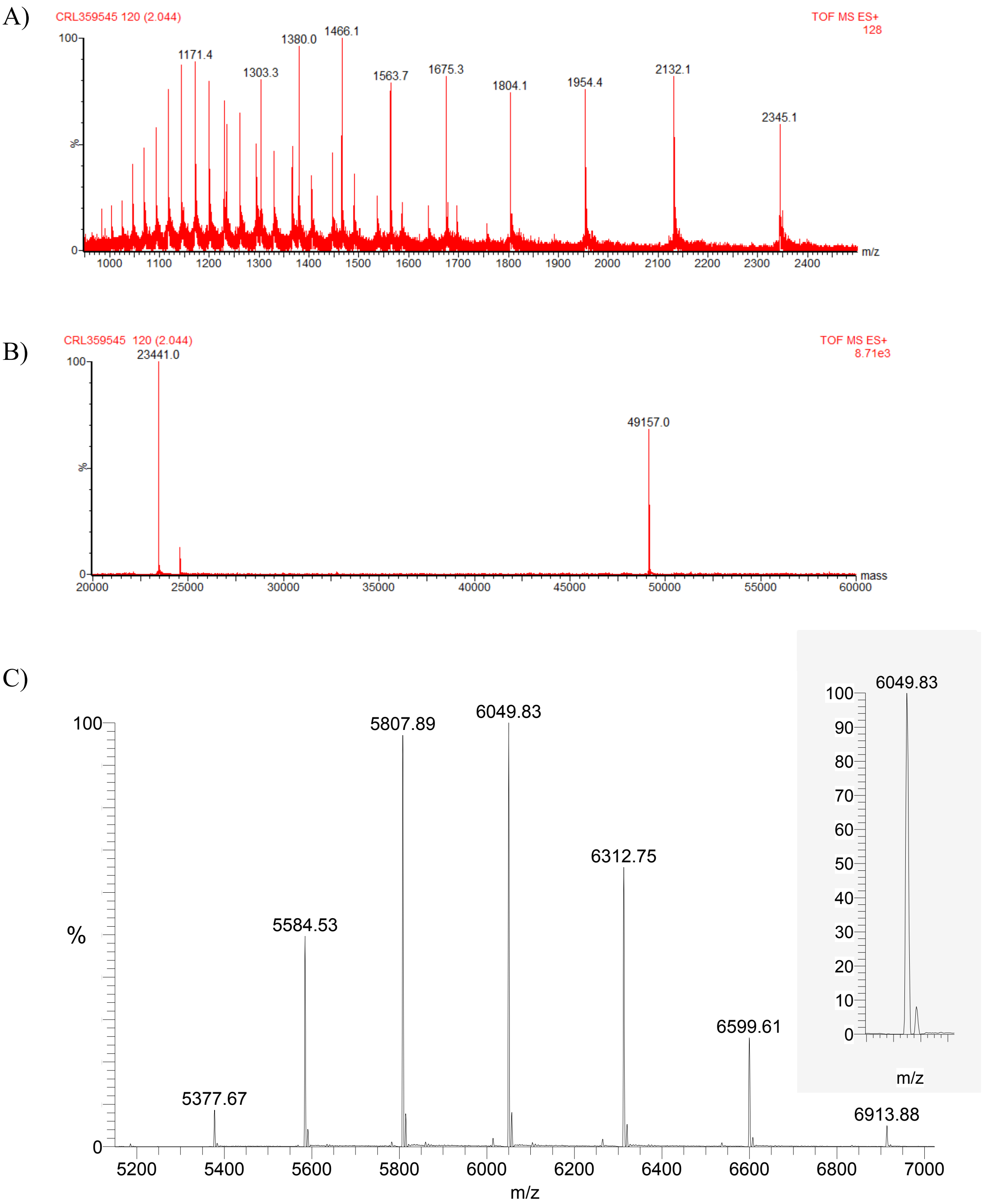

Deconvoluted mass: 145171 (expected: 145167)

Figure S14. A) Raw and B) deconvoluted LC-MS data for PNGase F treated Herceptin. Calculated mass of light chain: 23443, observed: 23441. Calculated mass of heavy chain 49156, observed 49156. N.B. The peak at 24578 is half the mass of the heavy chain and is a consequence of the deconvolution process. C) Native mass spectra of deglycosylated Herceptin following Protein G purification with inset close-up of the base peak (+24 charge state).

3.4 Glycation control reactions

*3.4.1 Glycation control reaction using activated sugar donor **2***

To test for background glycation reaction, Ab was combined with activated donor sugar **2** in the absence of enzyme, at various pH. The results indicate that at pH 7.4 significant glycation occurs within just one hour. This is substantially reduced at pH 7 and 6.5 but can still become significant after prolonged reaction time (figure S15). Notably the glycation process appears to occur on both the light and heavy chains of the Ab. Glycation was found to be concentration and pH dependent. The pH dependence is presumably due to a combination of greater deprotonation of lysine residues in the protein backbone and lower activity of Endo S (the endohexosaminidase enzymes typically have activity optima in the range pH 4-8).[10] Subsequently all glycosylation reactions were carried out at pH 6.5; at this pH glycation only reached significant levels after around 4 hours reaction time, whereas at pH 7.0 and 7.4 glycation was observed after just one hour.

Notably the bands arising from non-specific attachment of glycans to the antibody appear more rapidly and reach considerably higher concentrations on the heavy chain than the light chain.

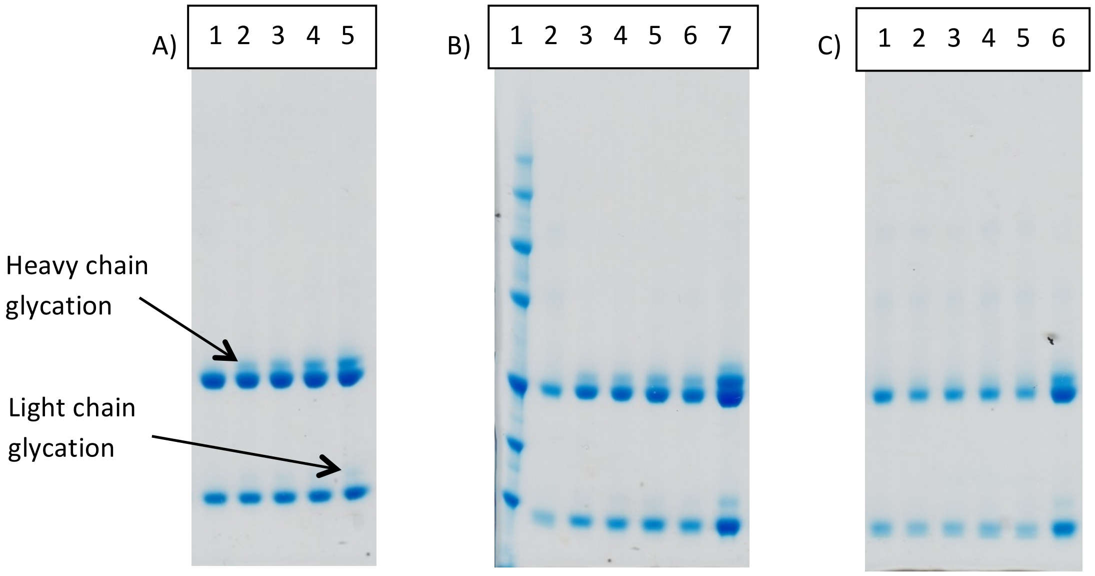

Figure S15. A) Antibody glycation at pH 7.4. Lane 1: Herceptin-GlcNAc. Lanes 2-5: reaction mixture at 30, 60, 120, 180 mins. B) Antibody glycation at pH 7.0. Lane 1: Protein marker. Lane 2: Herceptin-GlcNAc. Lanes 3-7: reaction mixture at 1, 2, 4, 8, 22 hours. C) Antibody glycation at pH 6.5. Lane 1: Herceptin-GlcNAc. Lanes 2-6: reaction mixture at 1, 2, 4, 8, 22 hours. Note the faint bands arising from non-specific attachment of glycans to the antibody.

Native MS indicated that at pH 7.4 the majority of the antibody had undergone some glycation after 4 hours reaction time, while at pH 6.5 the level of glycation was substantially lower (figure S16).

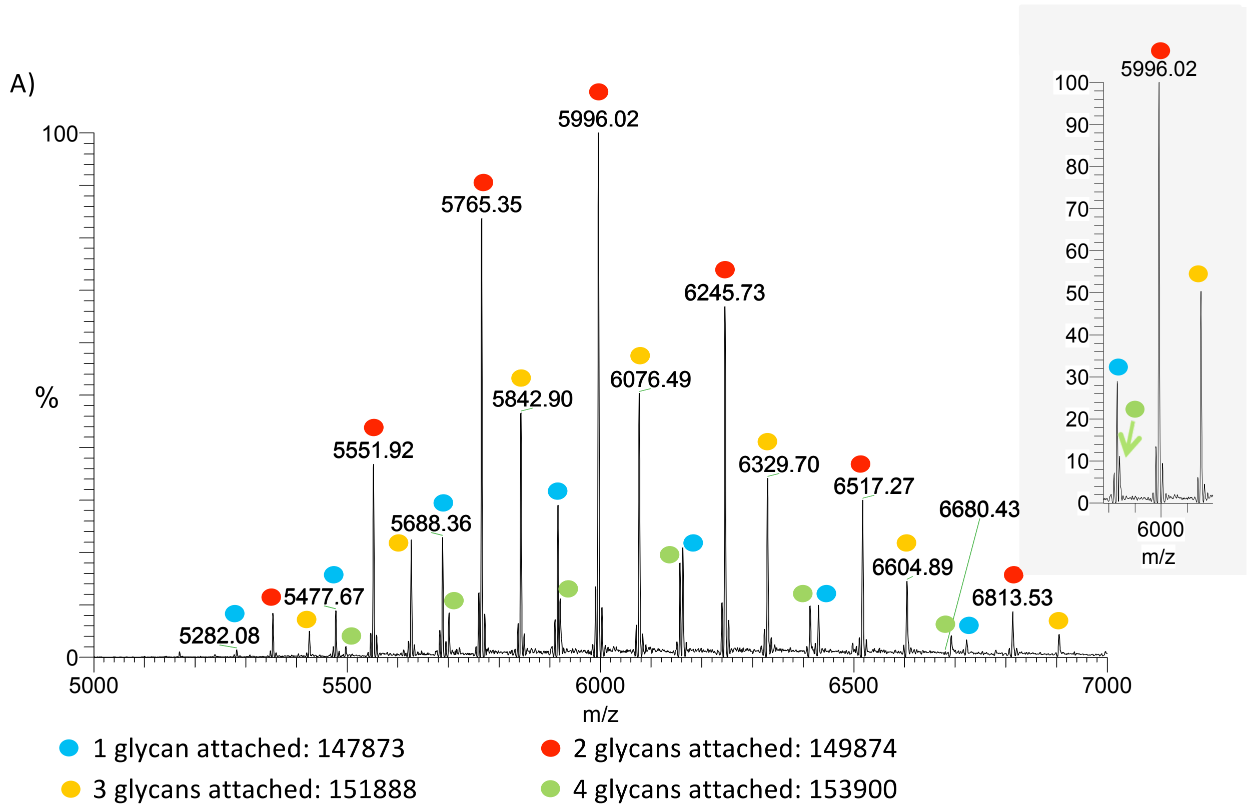

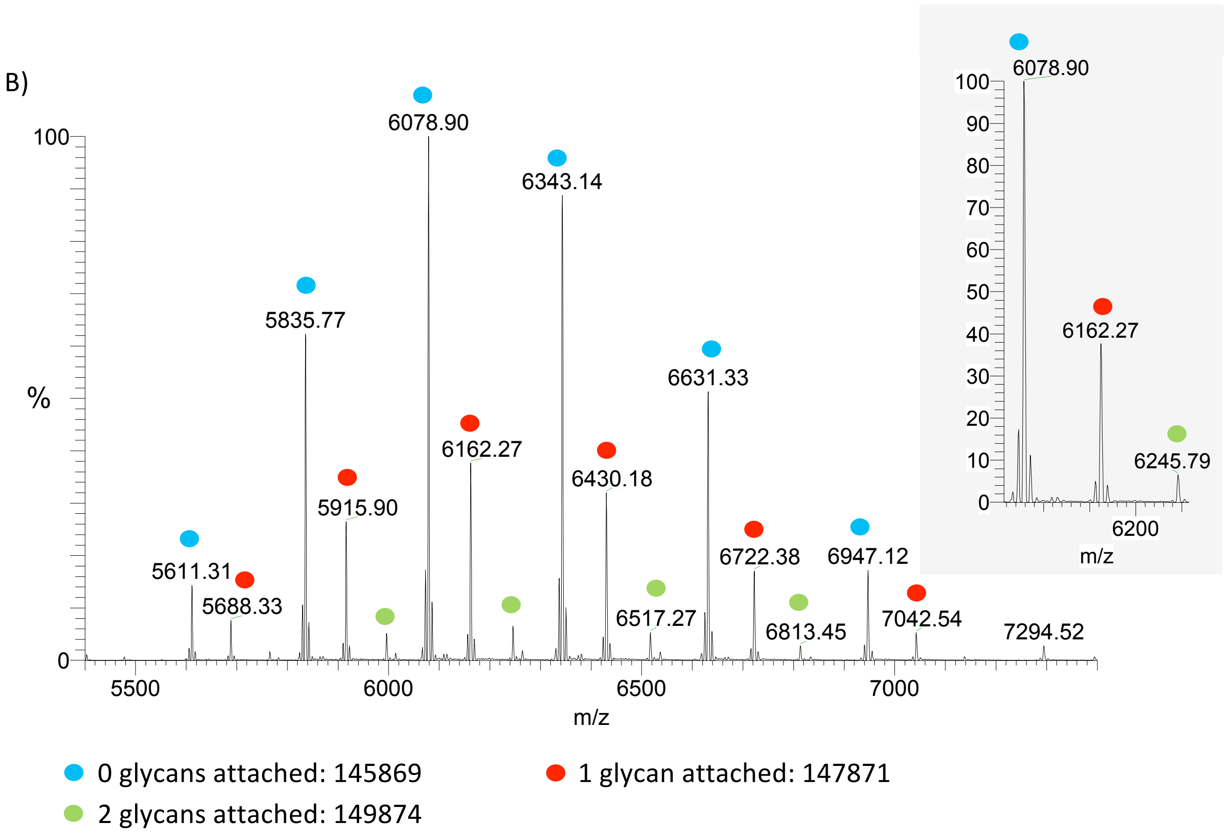

Figure S16. Native mass spectra of glycation of **1** using oxazoline **2** in the absence of enzyme, with inset close-up of the base peak; A) at pH 7.4 (+25 charge state inset). B) at pH 6.5 (+24 charge state inset). Deconvoluted masses indicated below the spectra.

Typical glycation control reaction using sugar oxazoline

Decasaccharide oxazoline **2** (2 x 50 μg, 2 x 70 equivalents) and antibody (50 μg) were combined in phosphate buffer (pH as required, final concentration of 100 mM, final enzyme concentration typically 2 mg/mL) in the absence of enzyme. Aliquots were removed from the reaction mixture, snap-frozen and stored at -20 °C. Subsequent analysis by SDS-PAGE under reducing conditions indicated that glycation levels varied depending on pH and reaction concentration.

*3.4.2 Glycation control reaction using non-activated sugar **4***

We considered the possibility that the apparent over-glycosylation of Herceptin may be due to non-covalent interaction between the sugar and protein during native MS analysis. To investigate this possibility we mixed reducing sugar lactol **4** with antibody **1** in the absence of enzyme. Aliquots were analysed by SDS-PAGE and after 4 hours incubation at 30 °C the reaction mixture was subjected either to two rounds of buffer exchange into 50 mM ammonium bicarbonate using Micro Bio-Spin P6 columns (Bio-Rad) or to purification by Protein G column. In the latter case, the fractions containing antibody were dialysed twice (slide-a-lyser, 10 kDa MWCO) against 50 mM ammonium bicarbonate. The concentration of the samples was subsequently adjusted to between 1.5 and 3 μM final concentration in 50 mM ammonium bicarbonate, pH 8.0. Analysis by SDS-PAGE indicated only the presence of the expected trimmed antibody **1**, with no indication of any glycation occurring (figure S17, lanes 5-10).

Surprisingly, native MS analysis of the same antibody-sugar mixture which had been subjected to two rounds of size exclusion chromatography using Micro Biospin 6 columns showed small amounts (< 5%) of an adduct between the sugar and the protein(figure S18A). Native MS analysis of samples which had been purified by Protein G showed no sugars present in the mixture (figure S18B). This indicates that non-covalent adducts *can* form between the protein and the large sugars used here, and that size exclusion using Micro BioSpin 6 columns does not efficiently remove all the sugars (MW around 2000 Da) from the protein (MW around 150000). Presumably this is due to the occurrence of relatively stable non-covalent bonding between the sugar and the protein. However, all glycosylation samples analysed herein were subjected to protein G purification and two or three rounds of dialysis prior to native MS analysis and so we conclude that non-covalent adducts are not the cause of the apparent over-glycosylation observed upon native MS of glycosylation reaction products.

*Typical glycation control reaction using lactol **4***

Decasaccharide lactol **4**, i.e. non-activated donor (50 μg, 70 equilvalents) and antibody (50 μg) were combined in phosphate buffer (pH 6.5, final concentration of 100 mM) in the absence of enzyme. Aliquots were removed from the reaction mixture, snap-frozen and stored at -20 °C. Subsequent analysis by SDS-PAGE under reducing conditions indicated that no glycation occurred using this sugar.

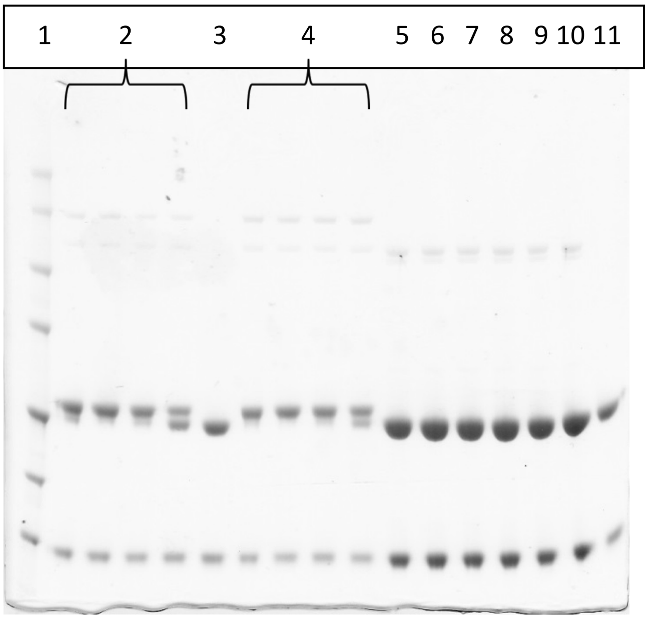

Figure S17. Lane 1: protein marker. Lanes 2 and 4: trial glycosylation reactions. Lane 3: Herceptin-GlcNAc. Lanes 5-10: reaction mixture at 1, 2, 3, 4, 6, 22 hours. Lane 11: Herceptin-GlcNAc.

A)

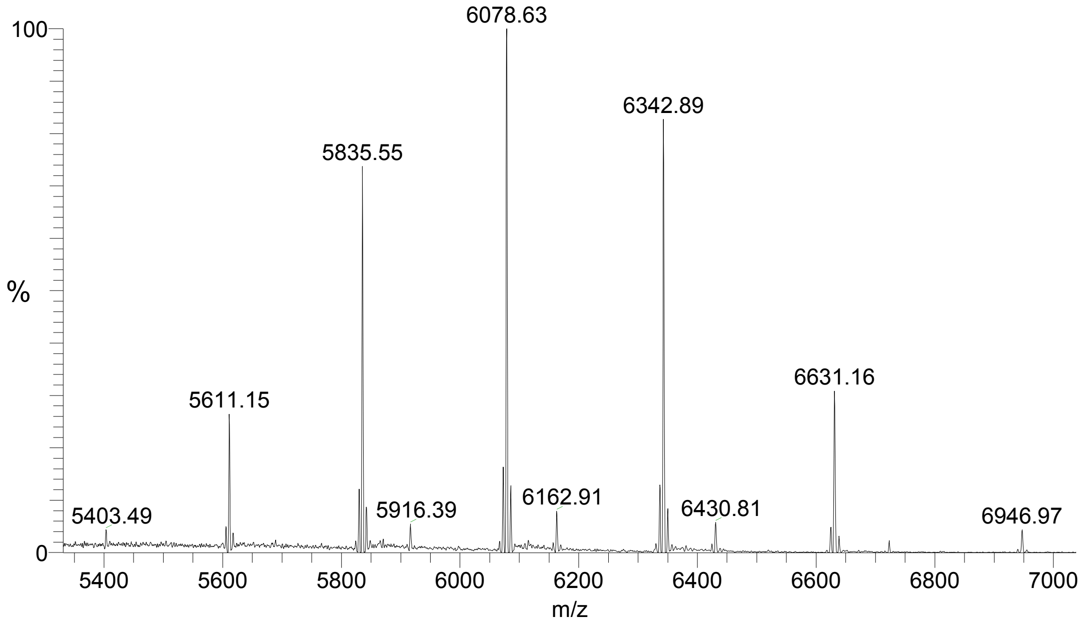

B)

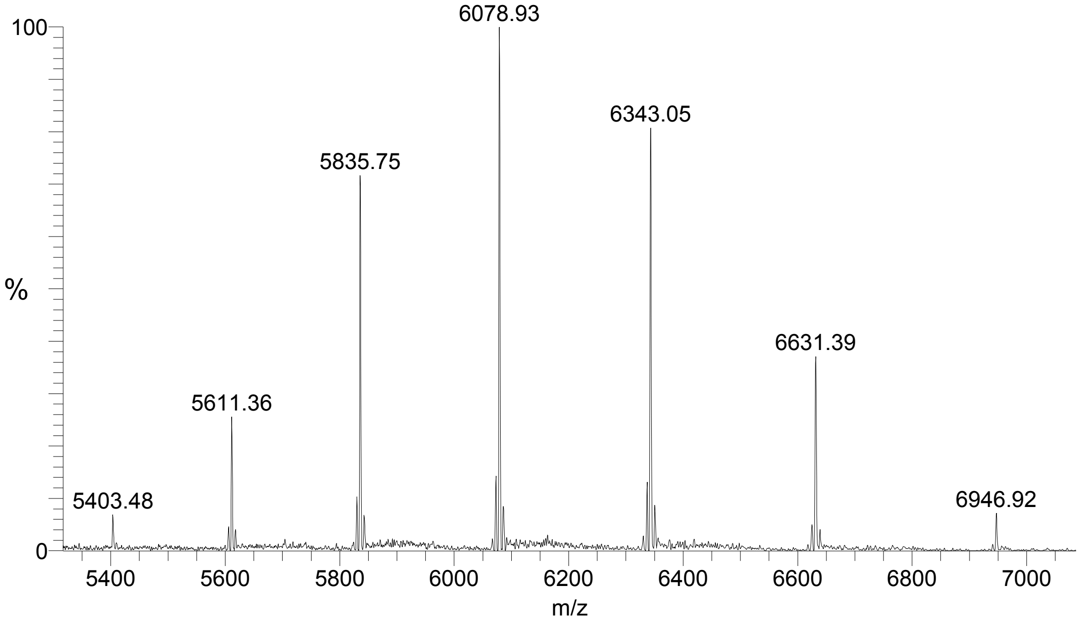

Figure S18. Native mass spectra of Herceptin after treatment with lactol **4** and subsequent purification by either A) two rounds of size exclusion chromatography using Micro BioSpin 6 columns or B) one round of Protein G affinity chromatography.

3.5 Optimized glycosylation

The glycosylation of **1** using **2** under optimized conditions (7 x 15 equivalents of donor, 10% wt loading of enzyme) was carried out several times at scales between 50 and 200 µg. Products were analysed by nMS to confirm the reproducibility of the reaction process. Typically levels of monoglycosylation were below 10% and levels of overglycosylation/glycation were below 1% (figure S19).

A)

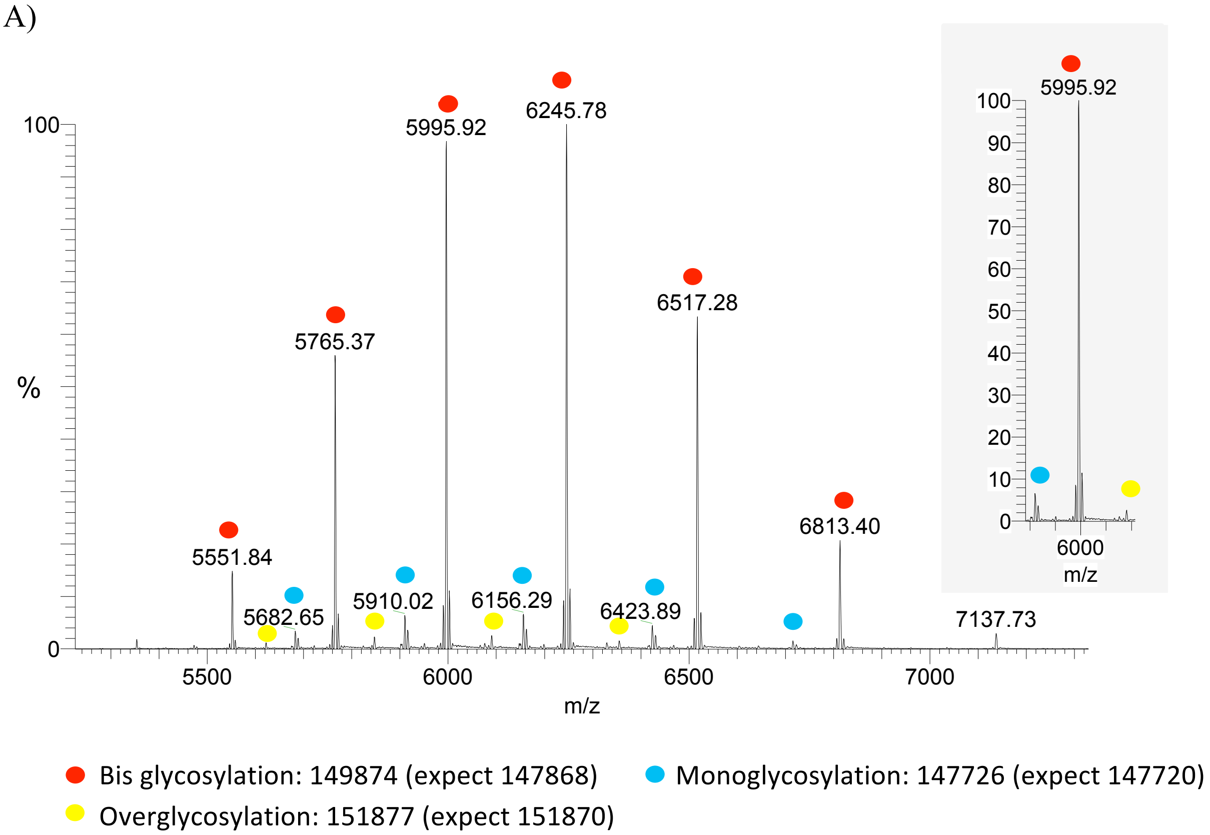

B)

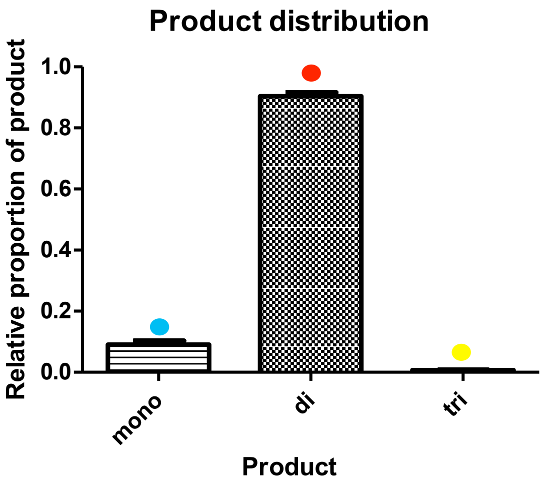

Figure S19. A) Typical native mass spectra for glycosylation of **1** using oxazoline **2** at pH 6.5 under optimized conditions, with inset close-up of the +24 charge state. Deconvoluted masses are shown beneath the spectrum. Note that the major peak for the monoglycosylated material corresponds to only one core Fucose on the antibody glycans. B) Mean product distribution obtained for this glycosylation reaction, obtained from five individual reactions.

Higher enzyme loading than 10% led to both very rapid glycosylation and subsequent hydrolysis of the newly formed glycosidic linkages. Thus, although such an approach would further decrease the glycation to negligible level, we were unable to purify the reaction mixture sufficiently rapidly to prevent significant loss of product to hydrolysis.

Five replicates of the 'optimised' conditions were investigated. These gave an overall average of <1% over-glycosylation, >90% product and 9% monoglycosylation. (0.7:90.4:9.1)

Notably the monoglycosylation product ions showed a change in pattern. Most peaks appear in groups of three which represent the major F2 (i.e. carrying two core fucose moieties) glycoform (around 86%), the minor F1 (carrying only one core fucose) glycoform (around 6%) and the minor unidentified component, as discussed above (8%). Following glycosylation the major peak of the mono-glycosylated product is the F1 glycoform. This may be due to either slower glycosylation of this species OR faster hydrolysis of the product (i.e. bisglycosylated) F1 glycoform, OR faster hydrolysis of the monoglycosylated F2 glycoform. It has previously been noted that Endo D has greated hydrolytic activity in the presence of core fucose but faster in transglycosylation in absence of core fucose. It may be that some steric effect is acting, with either a relief in steric clash favouring hydrolysis for core fucosylated glycoforms and the lower steric clash of afucosylated glycoforms leading to an increase in the rate of the glycosylation process.[11] Notably the activities of Endo F_1 and F_3 are also known to be affected by presence of core fucose.[12]

Immunoglobulin which has undergone glycation is only present in low quantities but this also appears to retain the expected 5-10% monofucosylation level.

3.6 Glycosylation using 'tagged' non-natural sugars

3.6.1 Typical glycosylation reaction using tagged sugar oxazoline donors

Decasaccharide oxazoline (2 x 70 equivalents) and antibody (1 equivalent) were combined in phosphate buffer (pH 6.5, final concentration of 100 mM, final enzyme concentration typically 2 mg/mL). Endo S D233Q (5 wt %) was added and the mixture incubated at 30 °C. Aliquots were removed from the reaction mixture, snap-frozen and stored at -20 °C until analysis by SDS-PAGE and/or LC-MS under reducing conditions.

*3.6.2 Glycosylation using alkyne-tagged glycan **6a***

Trimmed Herceptin **1** (600 µg, 70.5 µL of an 8.5 mg/mL solution in PBS, 4 nmol) and decasaccharide oxazoline **6a** (600 µg, 60 µL of a 10 mg/mL aqueous solution, 280 nmol) were combined in PBS (30 µL) and phosphate buffer (pH 6.5, 41 µL of a 500 mM solution). Endo S D233Q (30 µL of a 1 mg/mL solution in PBS, 30 µg, 5 wt %) was added and the mixture incubated at 30 °C. After 40 minutes a second bolus of oxazoline (600 µg) was added, to give a final reaction mixture concentration of 100 mM with respect to phosphate buffer and 2 mg/mL with respect to antibody. The mixture was incubated for a further 100 mins, at which point LC-MS analysis indicated complete conversion to glycosylated product. The reaction mixture was loaded onto a Protein A spin column and purified according to the manufacturer's instructions. Fractions containing antibody were combined and dialysed against twice against 1 L TBS/NaCl (50 mM Tris-HCl, 150 mM NaCl, pH 7.4) using a slide-a-lyser, 20 kD MWCO and twice against PBS. The solution was then concentrated using a vivaspin 500 (10 kDa MWCO) to give 350 µg of antibody as a 2.2 mg/mL solution (58% protein recovery).

For subsequent native MS analysis, an aliquot of the product was buffer exchanged twice into ammonium bicarbonate (50 mM, pH 8.0) using two Micro BioSpin 6 columns.

Analysis by LC-MS under reducing conditions indicated the presence of only one heavy chain component, that corresponding to the expected glycosylation process. However, nMS analysis indicated that minor components were also present, corresponding to attachment of one or three glycans to the intact antibody (figure S20). Analysis of the relative intensities of the product peaks indicated that the mixture comprised around 74% desired bisglycosylated product, 22% monoglycosylated and 4% material which carried three glycans.

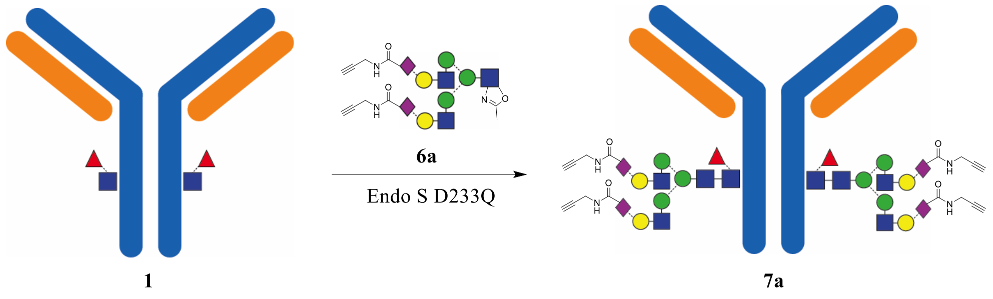

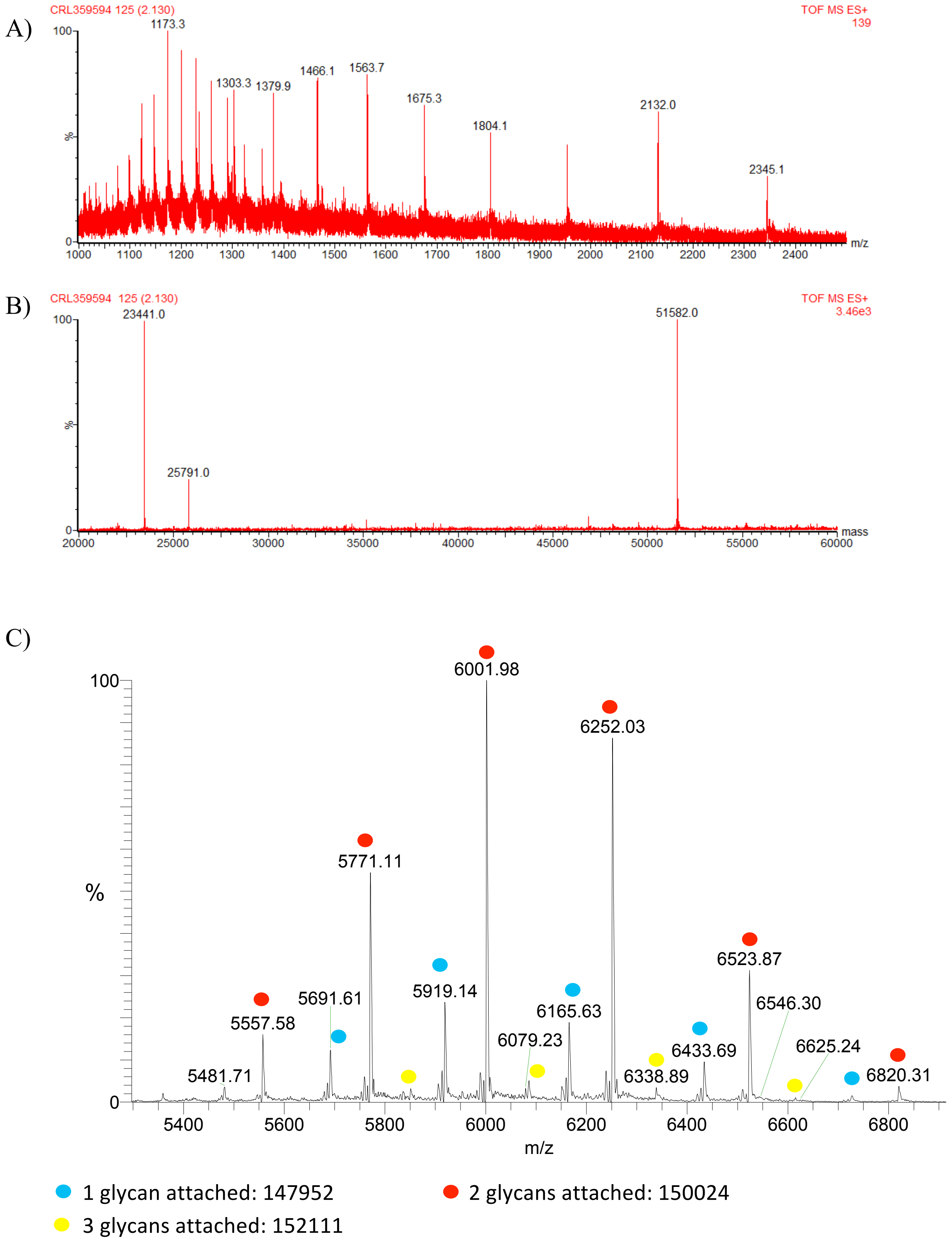

Figure S20. A) Raw and B) deconvoluted LC-MS data for glycosylation of **1** using alkyne-tagged glycan **6a**. Calculated mass of light chain: 23443, observed: 23441. Calculated mass of heavy chain 51581, observed 51582. N.B. The peak at 25791 is half the mass of the heavy chain and is a consequence of the deconvolution process. C) Native mass spectra of the product of glycosylation of **1** with **6a** after Protein A purification. Deconvoluted masses indicated below the spectra.

*3.6.3 Glycosylation using azide-tagged glycan **6b***

Trimmed Herceptin **1** (200 µg, 25 µL of an 8 mg/mL solution in PBS, 1.3 nmol) and decasaccharide oxazoline **6b** (212 µg, 21.2 µL of a 10 mg/mL aqueous solution, 93 nmol) were combined in PBS (7 µL) and phosphate buffer (pH 6.5, 20 µL of a 500 mM solution). Endo S D233Q (11.5 µL of a 1.1 mg/mL solution in PBS, 10 µg, 5 wt %) was added and the mixture incubated at 30 °C. After 40 minutes a second bolus of oxazoline (212 µg) was added, to give a final reaction mixture concentration of 100 mM with respect to phosphate buffer and 2 mg/mL with respect to antibody. The mixture was incubated for a further 2 hours, at which point LC-MS analysis indicated complete conversion to glycosylated product. The reaction mixture was loaded onto a Protein A spin column and purified according to the manufacturer's instructions. Fractions containing antibody were combined and dialysed against twice against 1 L TBS/NaCl (50 mM Tris-HCl, 150 mM NaCl, pH 7.4) using a slide-a-lyser, 20 kD MWCO and twice against PBS. The solution was then concentrated using a vivaspin 500 (10 kDa MWCO) to give 57 µL of a 2.15 mg/mL solution (61% protein recovery).

For subsequent native MS analysis, an aliquot of the product was buffer exchanged twice into ammonium bicarbonate (50 mM, pH 8.0) using two Micro BioSpin 6 columns.

Analysis by LC-MS under reducing conditions indicated the presence of only one heavy chain component, that corresponding to the expected glycosylation process. However, nMS analysis indicated that minor components were also present, corresponding to attachment of one or three glycans to the intact antibody (figure S21). Analysis of the relative intensities of the product peaks indicated that the mixture comprised around 65% desired bisglycosylated product, 27% monoglycosylated and 9% material which carried three glycans.

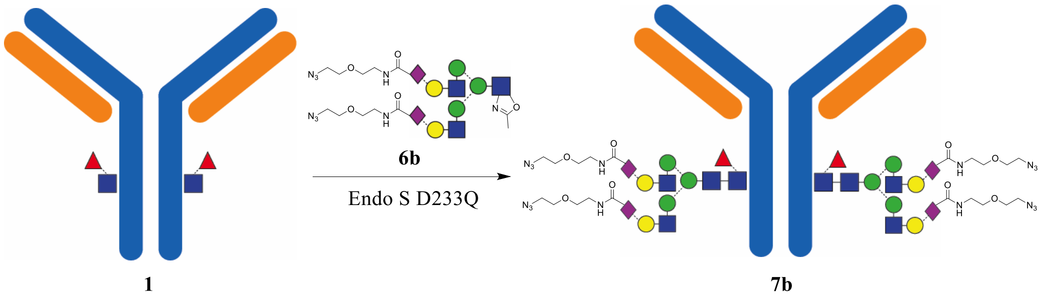

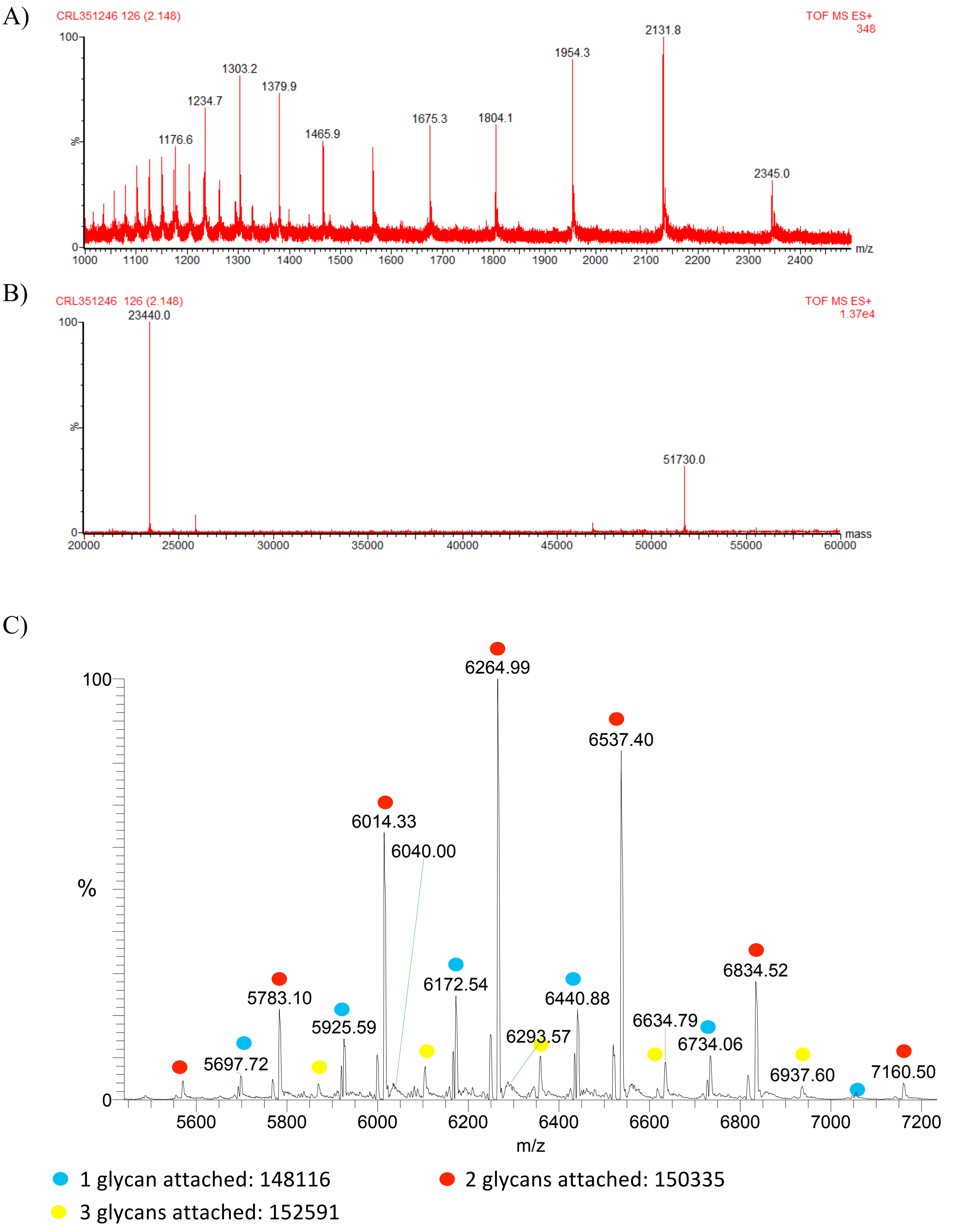

Figure S21. A) Raw and B) deconvoluted LC-MS data for glycosylation of **1** using azide-tagged glycan **6b**. Calculated mass of light chain: 23443, observed: 23440. Calculated mass of heavy chain 51731, observed 51730. N.B. The peak at 25865 is half the mass of the heavy chain and is a consequence of the deconvolution process. C) Native mass spectra of the product of glycosylation of **1** with **6b** after Protein A purification. Deconvoluted masses indicated below the spectra.

*3.6.4 Glycosylation using thiol-tagged glycan **6c***

Trimmed Herceptin **1** (100 µg, 15.4 µL of a 6.5 mg/mL solution in PBS, 0.68 nmol) and decasaccharide oxazoline **6c** (101 µg, 10.4 µL of a 10 mg/mL aqueous solution, 47.6 nmol) were combined in phosphate buffer (pH 6.5, 10.4 µL of a 500 mM solution). Endo S D233Q (5.7 µL of a 0.9 mg/mL solution in PBS, 5 µg, 5 wt %) was added and the mixture incubated at 30 °C. After 40 minutes a second bolus of oxazoline (101 µg) was added, to give a final reaction mixture concentration of 100 mM with respect to phosphate buffer and 2 mg/mL with respect to antibody. The mixture was incubated for a further 105 minutes, at which point LC-MS analysis indicated complete conversion to glycosylated product. The reaction mixture was loaded onto a Protein A spin column and purified according to the manufacturer's instructions. Fractions containing antibody were combined and dialysed against twice against 1 L TBS/NaCl (50 mM Tris-HCl, 150 mM NaCl, pH 7.4) using a slide-a-lyser, 20 kD MWCO and twice against PBS. The solution was then concentrated using a vivaspin 500 (10 kDa MWCO) to give 45 µL of a 0.82 mg/mL solution (50% protein recovery).

For subsequent native MS analysis, an aliquot of the product was buffer exchanged twice into ammonium bicarbonate (50 mM, pH 8.0) using two Micro BioSpin 6 columns.

Analysis by LC-MS under reducing conditions indicated the presence of only one heavy chain component, that corresponding to the expected glycosylation process. However, nMS analysis indicated that minor components were also present, corresponding to attachment of zero, one or three glycans to the intact antibody (figure S22). Analysis of the relative intensities of the product peaks indicated that the mixture comprised around 70% desired bisglycosylated product, 12% monoglycosylated and 13% material which carried three glycans. Around 5% of the antibody carried no glycans at all. The broader product distribution and the presence of unglycosylated antibody suggests that Endo S cannot process this non-natural substrate as efficiently as the azido- or alkynyl-tagged analogues.

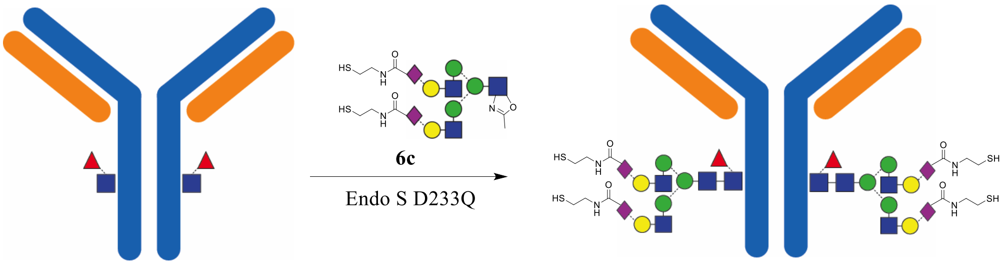

1 **7c**

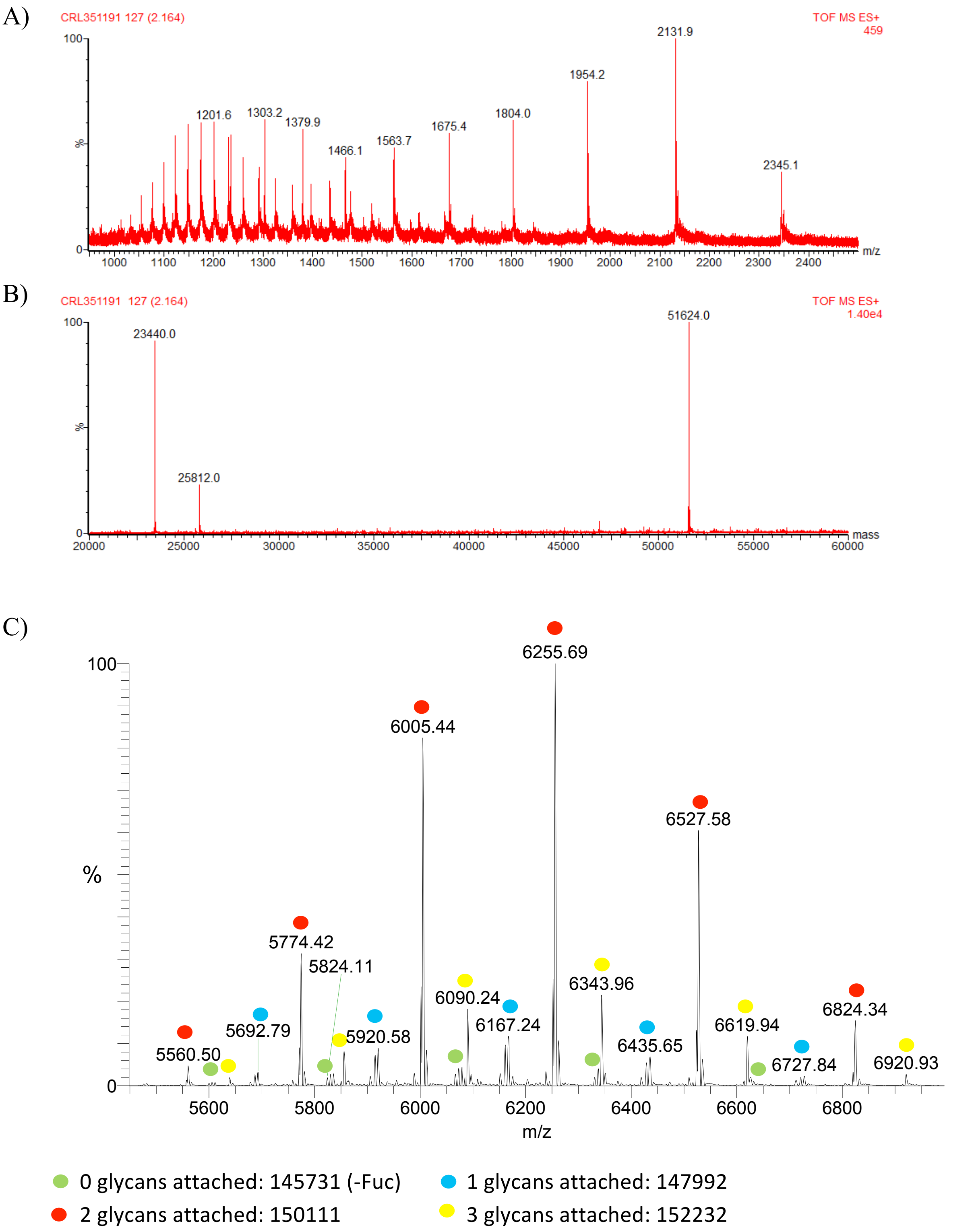

Figure S22. A) Raw and B) deconvoluted LC-MS data for glycosylation of **1** using thiol-tagged glycan **6c**. Calculated mass of light chain: 23443, observed: 23440. Calculated mass of heavy chain 51625, observed 51624. N.B. The peak at 25812 is half the mass of the heavy chain and is a consequence of the deconvolution process. C) Native mass spectra of the product of glycosylation of **1** with **6c** after Protein A purification. Deconvoluted masses indicated below the spectra.

3.6.5 Glycosylation using pyridyl disulfide-tagged glycan ***6d***

Glycosylation using donor **6d** required three additions of oxazoline in order to drive the reaction to condition. This indicates lower efficiency in the glycosylation reaction which may reflect an inherent preference by Endo S for more 'natural' sugar substrates, or may be due to the lower solubility of this substrate compared to those carrying smaller 'tags'.

Trimmed Herceptin **1** (150 µg, 24.2 µL of a 6.5 mg/mL solution in PBS, 1.0 nmol) and decasaccharide oxazoline **6d** (165 µg, 16.5 µL of a 10 mg/mL aqueous solution, 70 nmol) were combined in phosphate buffer (pH 6.5, 15.5 µL of a 500 mM solution). Endo S D233Q (8.3 µL of a 0.9 mg/mL solution in PBS, 7.5 µg, 5 wt %) was added and the mixture incubated at 30 °C. After 40 and 80 minutes further boluses of oxazoline (165 µg) were added. The mixture was incubated for a further 60 minutes, at which point LC-MS analysis indicated complete conversion to glycosylated product. The reaction mixture was loaded onto a Protein A spin column and purified according to the manufacturer's instructions. Fractions containing antibody were combined and dialysed against twice against 1 L TBS/NaCl (50 mM Tris-HCl, 150 mM NaCl, pH 7.4) using a slide-a-lyser, 20 kD MWCO and twice against PBS. The solution was then concentrated using a vivaspin 500 (10 kDa MWCO) to give 22 µL of a 3.85 mg/mL solution (60% protein recovery).

For subsequent native MS analysis, an aliquot of the product was buffer exchanged twice into ammonium bicarbonate (50 mM, pH 8.0) using two Micro BioSpin 6 columns.

Analysis by LC-MS under reducing conditions indicated the presence of only one heavy chain component, that corresponding to the expected glycosylation process. However, nMS analysis indicated that minor components were also present, corresponding to attachment of zero, one or three glycans to the intact antibody (figure S23). Analysis of the relative intensities of the product peaks indicated that the mixture comprised around 40% desired bisglycosylated product, 27% monoglycosylated and 22% material which carried three glycans. Around 11% of the antibody carried no glycans at all. The native MS of this reaction mixture showed a very high level of heterogeneity, which we presume to be due to some reduction of the disulfide bonds present in the glycans with corresponding loss of thiopyridyl groups.

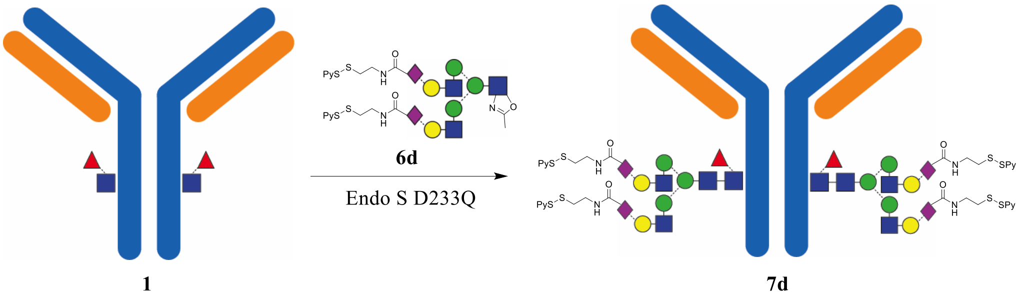

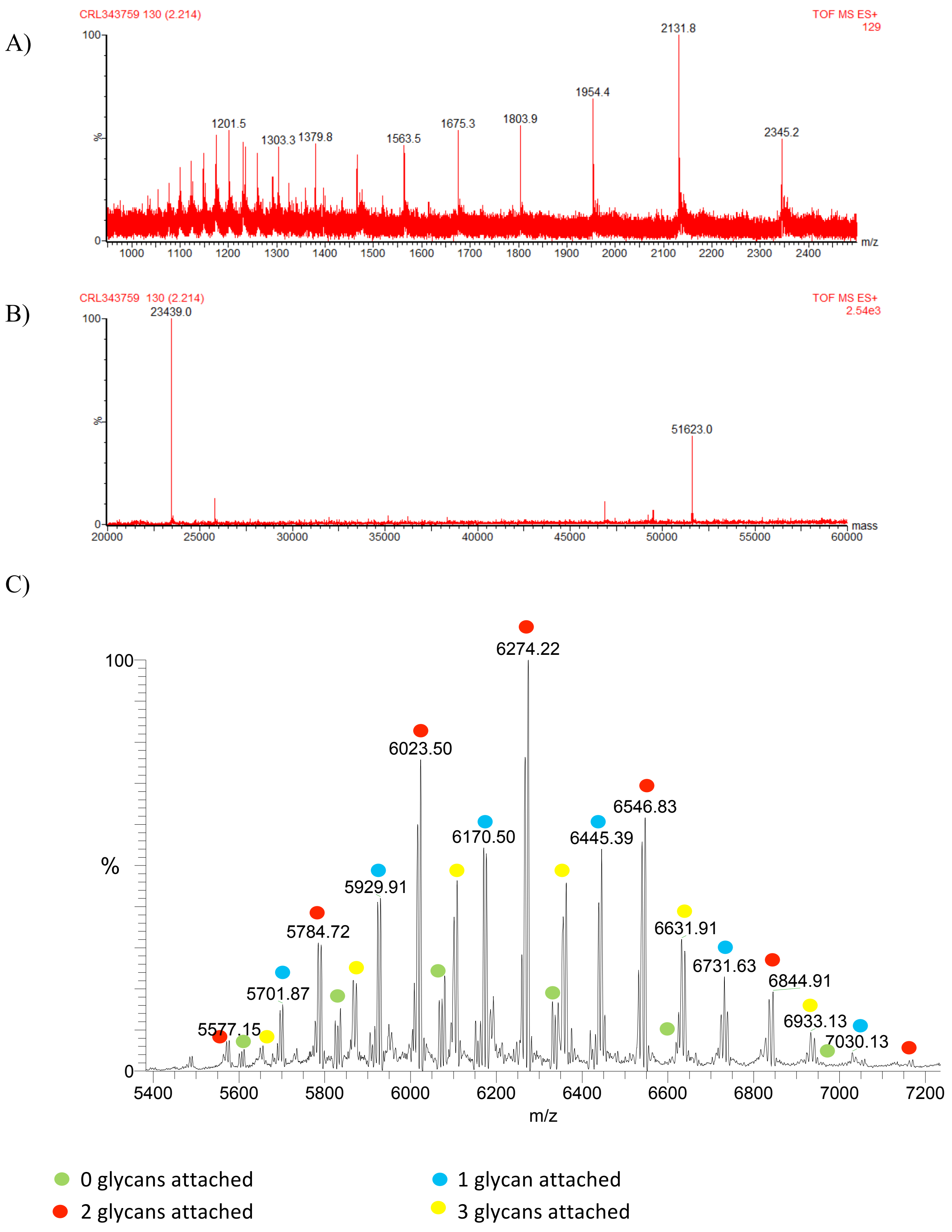

Figure S23. A) Raw and B) deconvoluted LC-MS data for glycosylation of **1** using dithiopyridyl-tagged glycan **6d**. Calculated mass of light chain: 23443, observed: 23439. Calculated mass of heavy chain 51625, observed 51623 (LC-MS is after reduction of the disulfide bonds so any heterogeneity due to loss of thiopyridyl groups would not be observed under these conditions). N.B. The peak at 25812 is half the mass of the heavy chain and is a consequence of the deconvolution process. C) Native mass spectra of the product of glycosylation of **1** with **6d** after Protein A purification.

*3.6.6 Glycosylation using iodoaryl-tagged glycan **6e***

Glycosylation using donor **6e** required three additions of oxazoline in order to drive the reaction to condition. This indicates lower efficiency in the glycosylation reaction which may reflect an inherent preference by Endo S for more ‘natural’ sugar substrates, or may be due to the lower solubility of this substrate compared to those carrying smaller ‘tags’.

Trimmed Herceptin **1** (155 µg, 22.6 µL of a 6.5 mg/mL solution in PBS, 1.05 nmol) and decasaccharide oxazoline **6e** (170 µg, 17 µL of a 10 mg/mL aqueous solution, 70 nmol) were combined in phosphate buffer (pH 6.5, 16.3 µL of a 500 mM solution). Endo S D233Q (8.6 µL of a 0.9 mg/mL solution in PBS, 7.75 µg, 5 wt %) was added and the mixture incubated at 30 °C. After 40 and 80 minutes further boluses of oxazoline (165 µg) were added. The mixture was incubated for a further 90 minutes, at which point LC-MS analysis indicated around 95% conversion to glycosylated product. The reaction mixture was loaded onto a Protein A spin column and purified according to the manufacturer’s instructions. Fractions containing antibody were combined and dialysed against twice against 1 L TBS/NaCl (50 mM Tris-HCl, 150 mM NaCl, pH 7.4) using a slide-a-lyser, 20 kD MWCO and twice against PBS. The solution was then concentrated using a vivaspin 500 (10 kDa MWCO) to give 25 µL of a 4.3 mg/mL solution (69% protein recovery).

For subsequent native MS analysis, an aliquot of the product was buffer exchanged twice into ammonium bicarbonate (50 mM, pH 8.0) using two Micro BioSpin 6 columns.

Analysis by LC-MS under reducing conditions indicated the presence of only one heavy chain component, that corresponding to the expected glycosylation process. However, nMS analysis indicated that minor components were also present, corresponding to attachment of zero, one or three glycans to the intact antibody (figure S24). Analysis of the relative intensities of the product peaks indicated that the mixture comprised around 59% desired bisglycosylated product and 35% monoglycosylated. Around 6% of the antibody carried no glycans at all and no material was observed which carried three glycans.

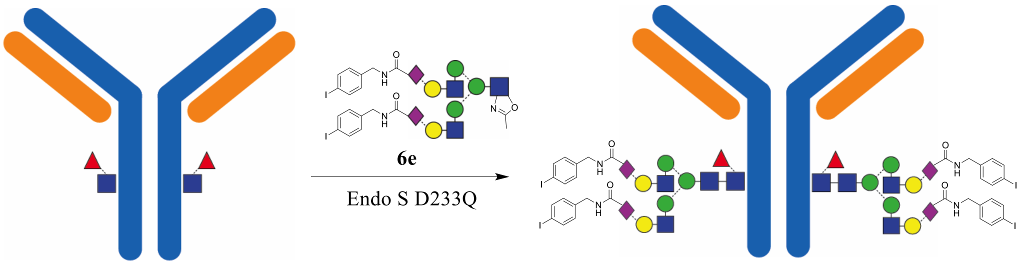

1 **7e**

A)

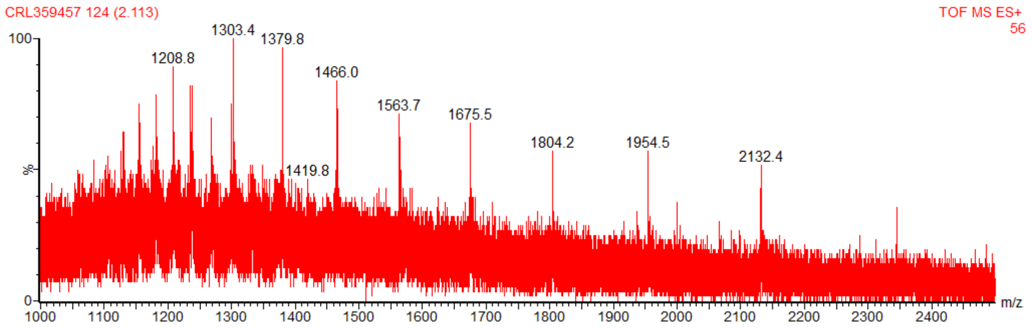

B)

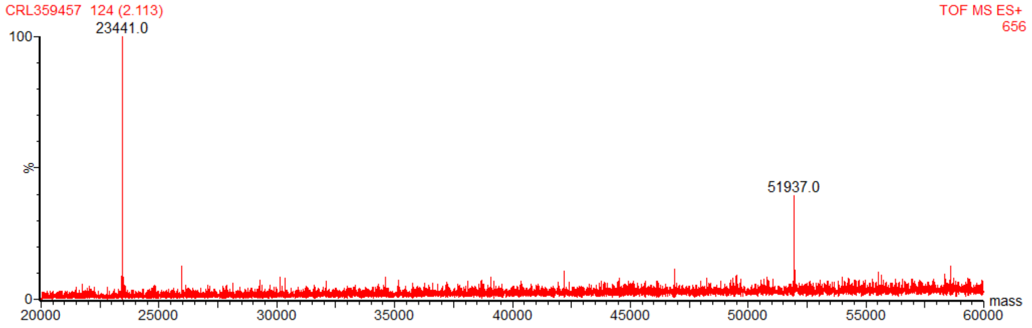

C)

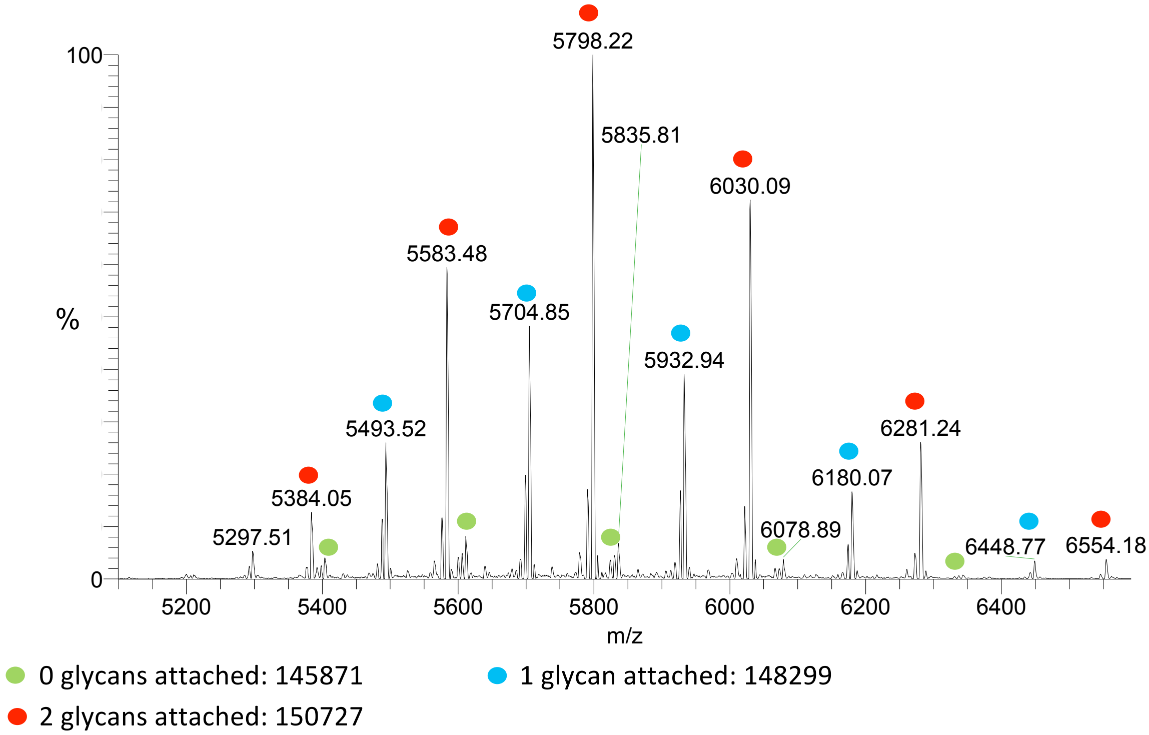

Figure S24. A) Raw and B) deconvoluted LC-MS data for glycosylation of **1** using iodoaryl-tagged glycan **6e**. Calculated mass of light chain: 23443, observed: 23441. Calculated mass of heavy chain 51937, observed 51937 C) Native mass spectra of the product of glycosylation of **1** with **6e** after Protein A purification.

Table S1. Proportions of products obtained following enzymatic glycosylation of **1** using various non-natural sugar donors.[a]

Glycan donor		Number of glycans attached to Ab (%)[b]				Target Ab	
		0	1	**2**	3		
**6a**	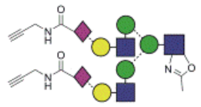	nd	21.9	**74.0**	4.1	**7a**	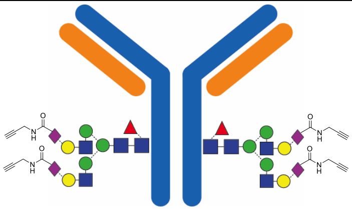
**6b**	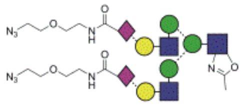	nd	26.7	**64.6**	8.8	**7b**	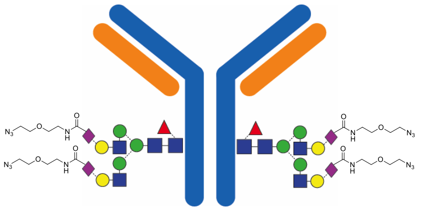
**6c**	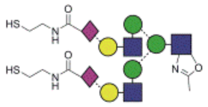	5.3	11.5	**70.1**	13.2	**7c**	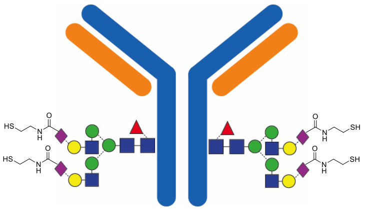
**6d**	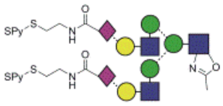	11.1	27.2	**39.7**	22.0	**7d**	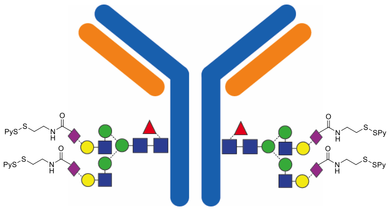
**6e**	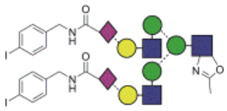	6.4	34.9	**58.7**	nd	**7e**	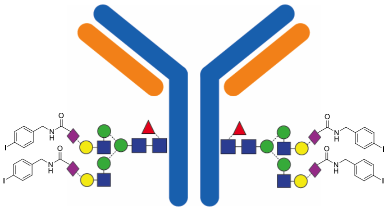

[a] Proportions were calculated by examination of the ratios of the products using the three most intense charge state peaks from the native MS. [b] nd: not detected.

The proportions of the various products obtained after glycosylation using non-natural donors are summarised in table S1. The percentage of the target remodelled antibody, i.e. that carrying two glycans, is shown in bold for each entry.

3.7 PNGase F treatment of glycated antibodies

To further confirm the non-enzymatic nature of the overglycosylation (see above), we treated samples which displayed triple glycosylation with PNGase F. This will efficiently remove *N*-linked glycans from Herceptin, but would be ineffectual at removing glyans attached chemically, i.e. during glycation. Analysis of these antibody samples after PNGase F treatment indicated that although the majority of glycans were removed, a proportion of glycans remained in place. Comparison with PNGase F treatment of commercial Herceptin which proceeded efficiently strongly suggests that the overglycosylation occurs in a non-enzymatic manner at alternative acceptor sites.

PNGase F treated Herceptin samples were subjected to two rounds of buffer exchange using Micro BioSpin columns. Analysis indicated complete removal of glycans from commercial Herceptin and from remodelled Herceptin which carried natural complex biantennary glycans. However, in the case of remodelled antibody samples bearing glycans with biorthogonal tags, removal of the glycans was not complete. Remodelled 'tagged' Abs produced by glycosylation under optimised conditions retained very low levels of glycan after PNGase F treatment, indicating low levels of glycation. Those Abs bearing non-natural tags which were introduced under non-optimised glycosylation conditions retained significant quantities of glycan even after PNGase F digestion and size exclusion purification. This indicates that significant glycation had occurred during the glycosylation reaction.

It should be noted that during glycosylation, the antibody is treated with >100 equivalents of sugar, and complete removal of the excess sugar appears to require Protein G or A affinity purification rather than two rounds of size exclusion chromatography. However, treatment of antibodies with PNGase F will necessarily lead to a maximum 2-3 equivalents of free sugar. In this case it appears that two rounds of size exclusion chromatography is sufficient to efficiently remove all free sugars from the antibody. Nevertheless, to ensure that there was no contamination of antibody samples with non-specifically bound sugar, when analysing samples for glycation we undertook Protein G purification after PNGase F treatment.

3.7.1 Native MS analysis of PNGase-treated glycation products

A portion of antibody carrying native glycans, **3**, (see figure S13A) was treated with PNGase F at 37 °C for 16 hours. We have observed that under these conditions, Herceptin is completely deglycosylated. However, PNGase F removes the core GlcNAc(Fuc) in **1** much less readily than the extended glycans in commercial or remodelled Herceptin. Treatment of **3** with PNGase F led to incomplete removal of the glycans, suggesting that some glycans were attached at sites other than the GlcNAc(Fuc), supporting our hypothesis that non-specific glycation has occurred (figure S25).

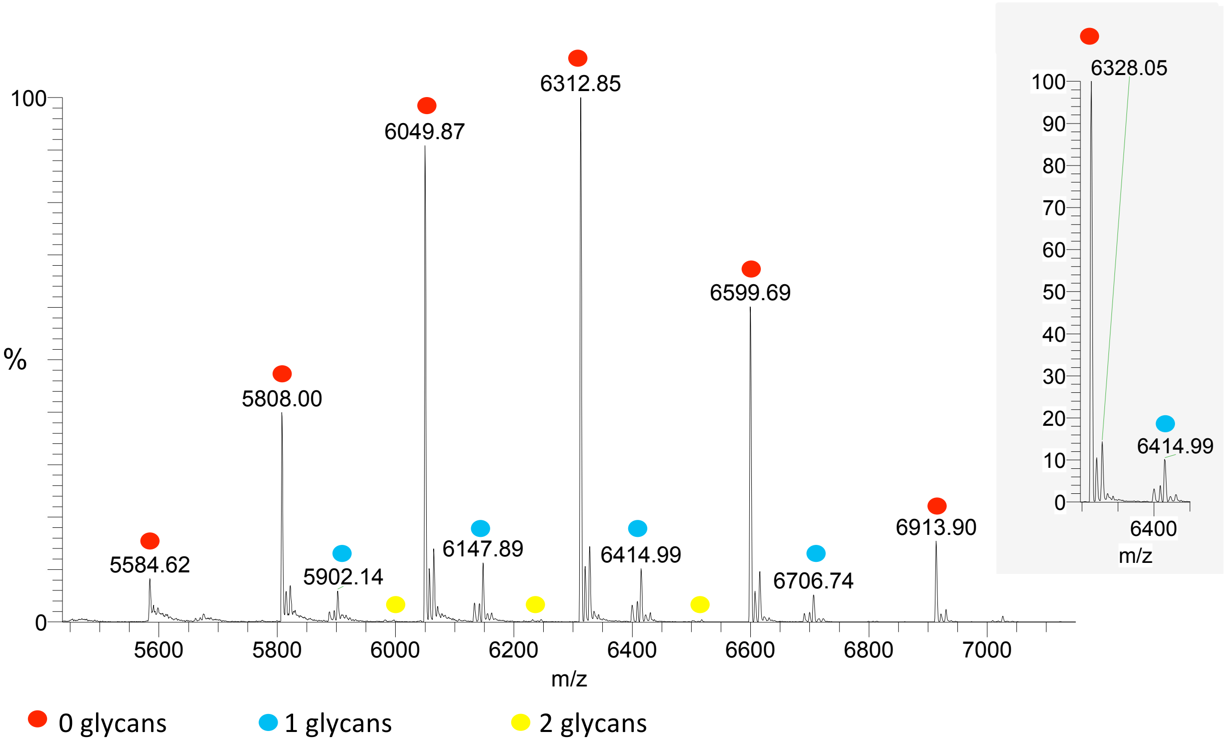

Figure S25. Native mass spectra obtained after PNGase F treatment of antibody **3**. A mixture of Abs carrying between 0 and 3 glycans indicates that non-specific glycation occurred during the glycosylation reaction preceding PNGase F digestion.

Similarly, treatment of thiol-tagged antibody (see figure S22) with PNGase F gave rise to incomplete removal of the glycans, suggesting that some non-specific glycan attachment had occurred (figure S26).

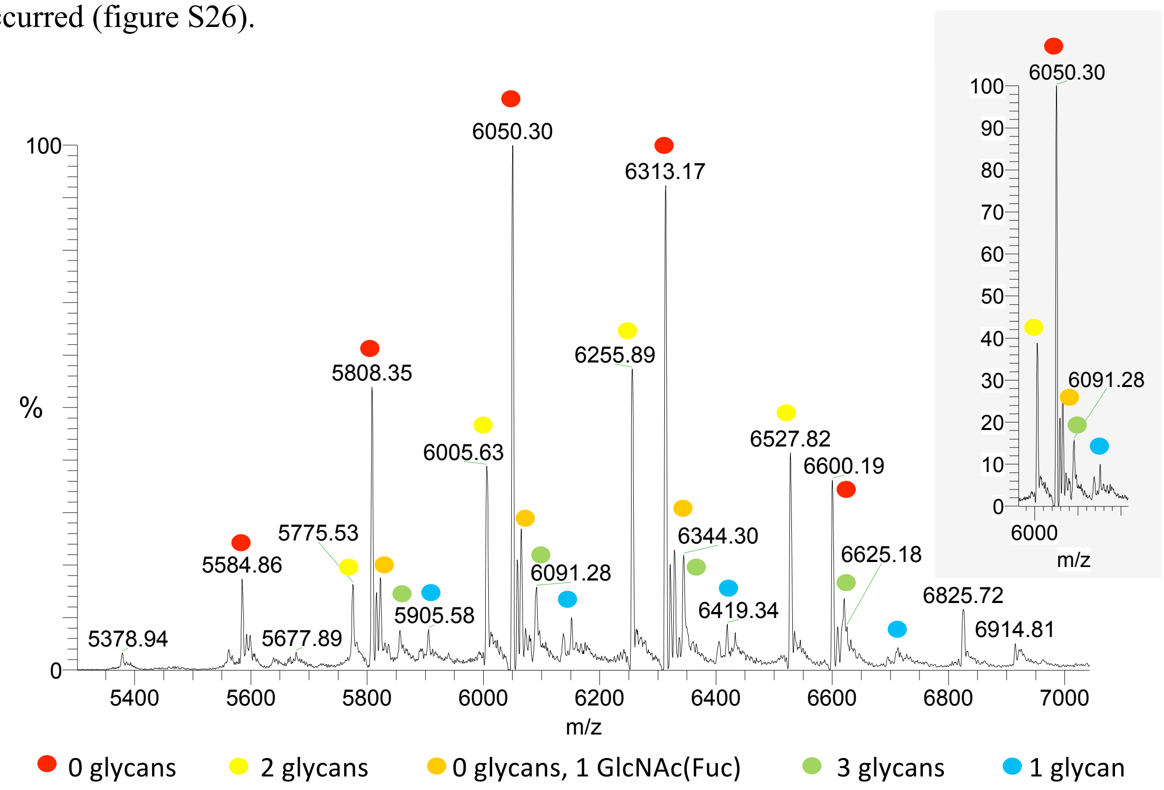

Figure S26. Native mass spectra obtained after PNGase F treatment of thiol-tagged antibody. A mixture of Abs carrying between 0 and 3 glycans indicates that non-specific glycation occurred during the glycosylation reaction preceding PNGase F digestion.

Similarly, treatment of dithiopyridyl tagged antibody (see figure S23) with PNGase F gave rise to a mixture of incompletely deglycosylated Ab products, again indicating that non-specific glycation occurred during the preceding glycosylation step (figure S27).

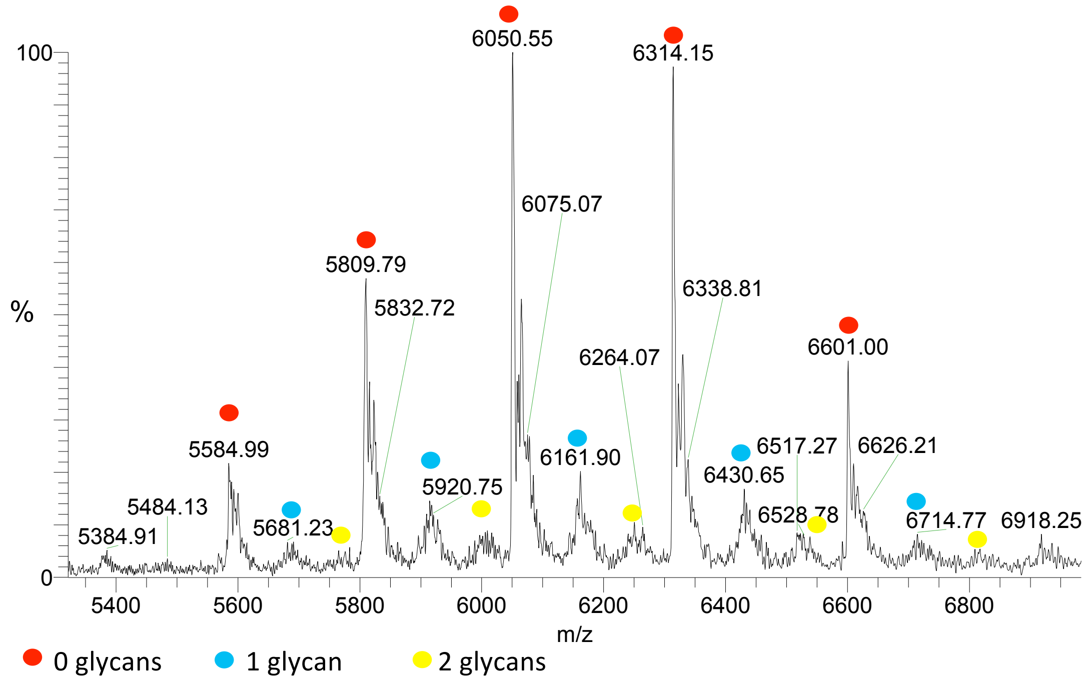

Figure S27. Native mass spectra obtained after PNGase F treatment of thiopyridyl-tagged antibody. A mixture of Abs carrying between 0 and 2 glycans indicates that non-specific glycation occurred during the glycosylation reaction preceding PNGase F digestion.

3.7.2 Denaturing MS analysis of PNGase F-treated glycation products

Analysis of trimmed Herceptin **1** which had undergone glycation with donor **2** was also carried out under denaturing and reducing conditions. Analysis using an LCT-Premier and LC-MS protocol did not show any glycation products. However, analysis using a Q-Exactive and direct infusion showed clearly that glycation had occurred mainly on the heavy chain, with very low levels apparent on the light chain (figures 3 and S28). The lysine-clipped heavy chain of Herceptin contains 31 lysines, 10 histidines and 13 arginines, whereas the light chain contains 13 lysines, 3 histidines and 7 arginine residues. As we would logically expect glycation to be occurring on nucleophilic residues such as there, it is unsurprising that the majority of glycation appears in the heavy chain.

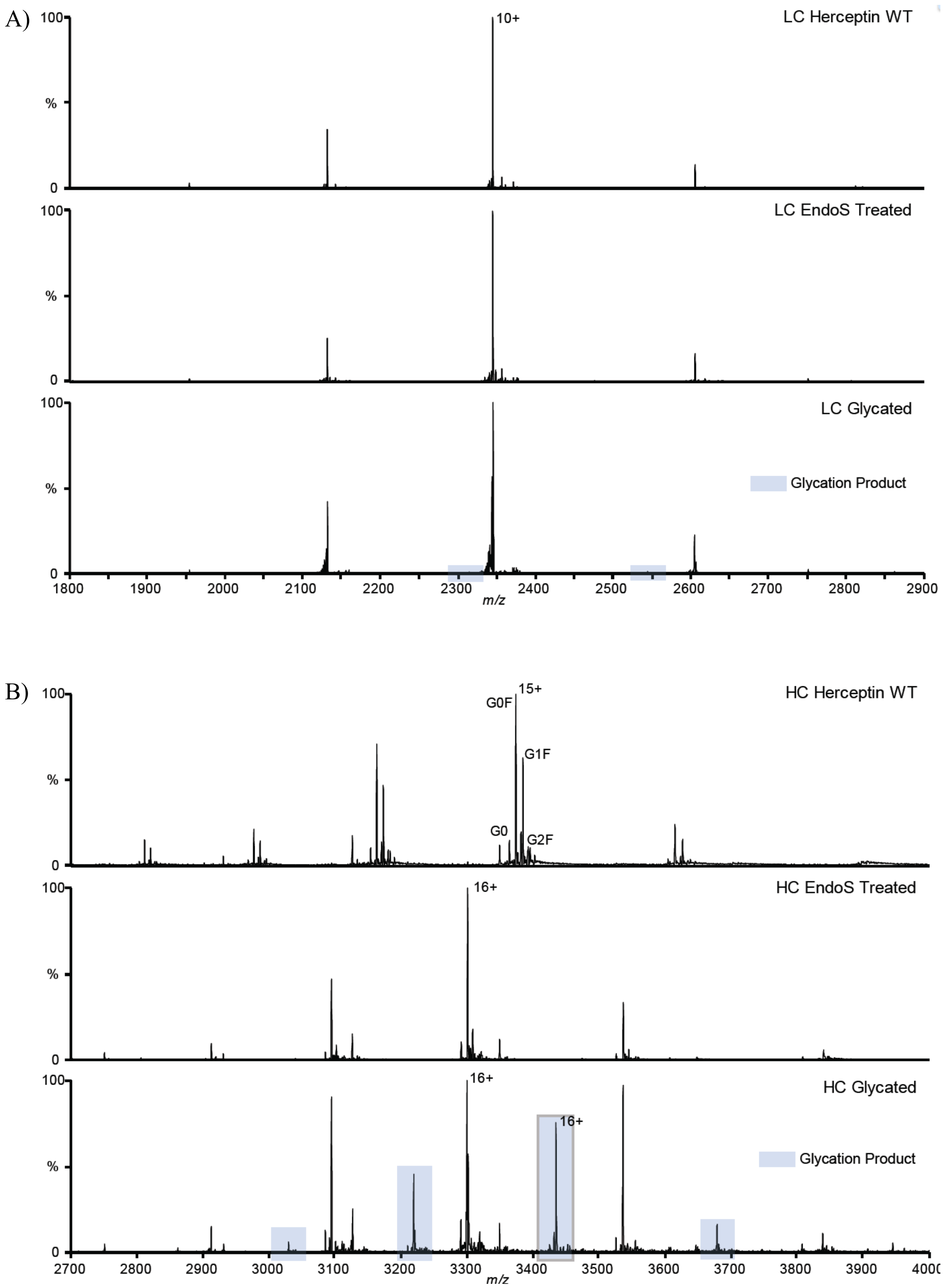

Figure S28. Reduced and denaturing mass spectra obtained using Q-Exactive for commercial Herceptin, EndoS treated Herceptin and glycated **1**. A) Light chain region of spectrum and B) heavy chain region of spectrum.

We attempted to determine more precisely the location of glycation on the antibody. The presence of three glycans on some of the glycosylated Ab products indicated that there is some susceptibility of the antibody to undergo glycation but this could occur either on nucleophilic residues of the protein backbone (e.g. lysine, histidine) or on alcohol groups of the glycans. Similarly we considered that the glycation of trimmed Herceptin **1** could be occurring on the sugar 'stub' of the Fc.

Commercial Herceptin, trimmed Herceptin **1** and PNGase F-treated Herceptin which carries no residual core sugar were all subjected to glycation using donor **2** at pH 7.4. In all cases glycation was clearly visible upon analysis by reducing SDS-PAGE gel (figure S29). Densitometry analysis indicated slight differences between the levels of glycation, with values around 29% for commercial Herceptin, 37% for **1** and 32% for PNGase F treated Herceptin. This may indicate some protective effect of the glycans on Herceptin due to e.g. blocking of possible glycation sites. It may also indicate that the GlcNAc(Fuc) core may itself be somewhat susceptible to non-specific glycation, although it appears that the glycan on commercial Herceptin is not itself substantially subject to glycation. Overall, the relatively small difference between levels of heavy chain glycation means we cannot conclude definitively that the presence of glycans on the antibody Fc has a large effect on non-specific glycation.

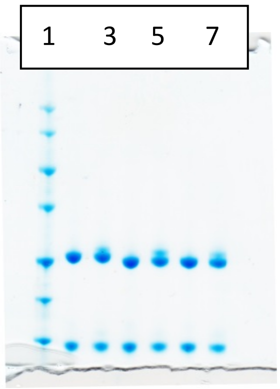

Figure S29. Reducing SDS-PAGE analysis of glycation reaction. Lane 1: protein marker. Lane 2: commercial Herceptin. Lane 3: glycation of commercial Herceptin, 4 hours. Lane 4: trimmed Herceptin **1**. Lane 5: glycation of **1**. Lane 6: PNGase F treated Herceptin. Lane 7: glycation of PNGase F treated Herceptin.

We attempted to determine more precisely the location of glycation reactions on the antibody. Digestion of antibodies using papain leads to release of the Fab from the Fc.[13] Analysis of glycated Ab samples which had been treated with papain showed the anticipated Fab fragment (MW 47638, observed 47637), along with minor peaks which may be due to incompletely digested antibody, either intact or minus one Fab domain (figure S30). Interestingly we were unable to identify the removed Fc domain, possibly due to further digestion by papain. The Fab fragment was accompanied by a species of molecular weight 2004 Da greater, which corresponds very closely to the expected mass of a Fab carrying one glycan. The Fab does not bear the *N*-glycosylation site, and so this observation indicates

clearly that glycation cannot solely be occurring at the remaining core sugar after EndoS trimming.

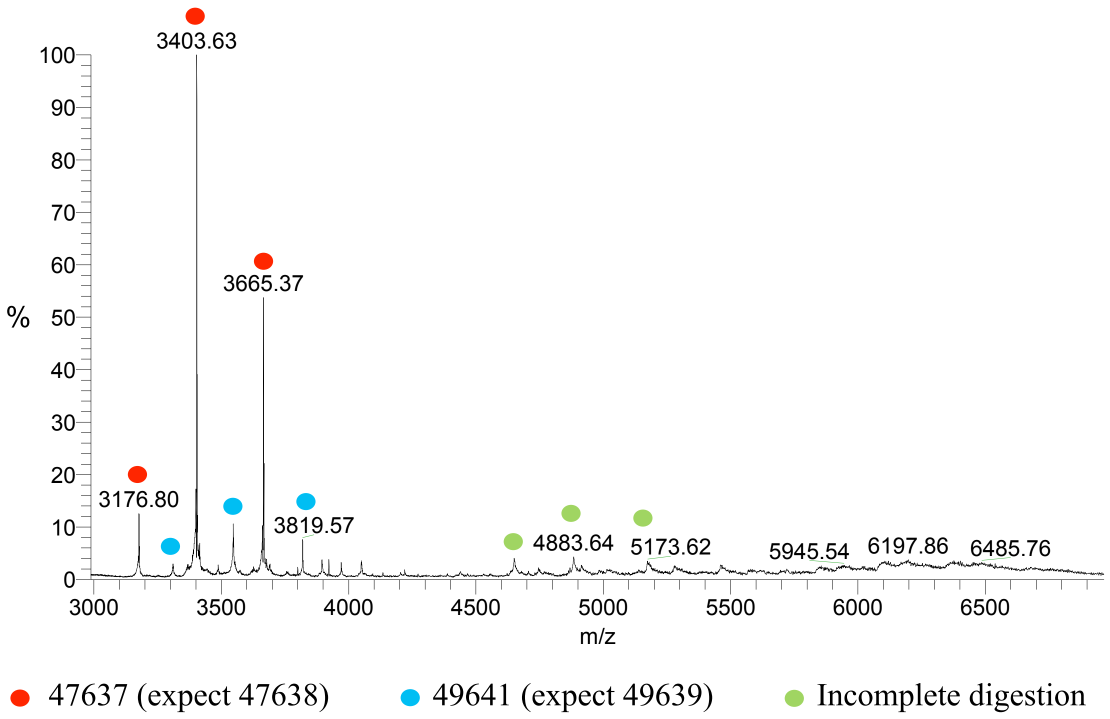

Figure S30. Native MS following papain digestion of glycated antibody sample.

3.7.3 Table summarising data obtained from nMS analysis of antibody samples

Entry	Sample	Proposed structure	Expected MW	Deconvoluted MW	%	Sugar MW
1	Commercial Herceptin	G0/G0F	147924	147923	4.9	
		G0F/G0F	148070	148084	30.9	
		G0F/G1F	148232	148246	35.4	
		G1F/G1F	148397	148406	20.1	
		G1F/G2F	148561	148570	6.6	
		G2F/G2F	148721	148732	2.0	
2	Trimmed Herceptin **1**	GlcNAc(Fuc) x 2	145865	145877	85	
		GlcNAc/GlcNAc(Fuc)	145719	145750	10	
		GlcNAc(Fuc) x 2 plus minor modification		146053	5	
3	Glycosylation with 2 x 70 equivalents of **2**	Monoglycosylation minus Fuc	147722	147725	6.5	2001.69
		Diglycosylation (**3**)	149870	149871	88.9	
		Diglycosylation + 1 x glycation	151873	151876	4.6	
4	Glycation at pH 7.4	1 glycan attached	147867	147873	15.5	2001.69
		2 glycans attached	149869	149874	52.4	
		3 glycans attached	151871	151888	26.3	
		4 glycans attached	153872	153900	5.8	
5	Glycation at pH 6.5	0 glycans attached	145865	145869	69.5	2001.69
		1 glycan attached	147867	147871	25.9	
		2 glycans attached	149868	149874	4.5	
6	Glycosylation with 7 x 15 equivalents of **2**	1 glycosylation, 1 core Fuc	147726	147721	9.5	2001.69
		2 glycosylations (**3**)	149873	149868	90.5	

Entry	Sample	Proposed structure	Expected MW	Deconvoluted MW	%	Sugar MW
7	Glycosylation with 2 x 70 equivalents of **6a** (alkyne tagged)	Diglycosylation (**7a**)	150017	150024	74	2075.76
		Monoglycosylation	147941	147952	21.9	
		Diglycosylation + 1 x glycation	152092	152111	4.1	
8	Glycosylation with 2 x 70 equivalents of **6b** (azide tagged)	Diglycosylation (**7b**)	150317	150335	73.0	2225.84
		Monoglycosylation	148091	148116	19.0	
		Diglycosylation + 1 x glycation	152543	152591	8.1	
9	Glycosylation with 2 x 70 equivalents of **6c** (thiol tagged)	Diglycosylation (**7c**)	150104	150111	70.1	2119.7
		Monoglycosylation	147985	147992	11.5	
		Diglycosylation + 1 x glycation	152224	152232	13.2	
		Starting material (**1**) -Fuc	145723	145731	5.3	
10	Glycosylation with 2 x 70 equivalents of **6d** (SSPy tagged)	Diglycosylation + 1 x glycation – Fuc	152733	152710	22.0	2337.9
		Diglycosylation (**7d**)	150541	150567	39.7	
		Monoglycosylation –Fuc	148203	148246	27.2	
		Starting material (**1**) -Fuc	145723	145749	11.1	
11	Glycosylation with 2 x 70 equivalents of **6e** (iodoaryl tagged)	Diglycosylation (**7e**)	150728	150727	58.7	2431.6
		Monoglycosylation	148297	148299	34.9	
		Starting material (**1**)	145865	145871	6.4	
12	PNGase F of **3** synthesised under unoptimised glycosylation conditions	Monoglycosylation - Fuc	147721	147735	6.5	
		Product (**3**)	149868	149878	88.9	
		(**3**) + 1 x glycation	151870	151880	4.6	

Entry	Sample	Proposed structure	Expected MW	Deconvoluted MW	%	Sugar MW
13	Glycosylation with 7 x 15 equivalents of **6b** (azide tagged)	Starting Material (**1**)	145865	145762	2.6	2225.84
		Monoglycosylation	148091	148111	18.2	
		Diglycosylation (**7b**)	150317	150332	76.2	
		Diglycosylation + 1 x glycation	152543	152561	3	
14	Glycosylation with 20 x 5 equivalents of **6b** (azide tagged)	Starting Material (**1**) – 2 x Fuc	145573	145568	<1	2225.84
		Monoglycosylation	148091	148097	8.9	
		Diglycosylation (**7b**)	150317	150318	89	
		Diglycosylation + 1 x glycation	152543	152540	<1	

Table S2. Details of expected and deconvoluted masses obtained after native MS analysis of various antibody samples discussed in this work.

3.8 Loading of Cargo Molecules

3.8.1 Loading of Rhodamine

*Synthesis of Her bearing rhodamine from azido-Her **7b***

Antibody **7b** (4 µL of a 2.5 mg/mL PBS solution, 10 µg protein) was treated with rhodamine derivative **8a** (0.25 µL of a 10 mM solution in acetonitrile; 40 equivalents). The mixture was incubated at room temperature with shaking for between 4 and 18 hours. The reaction mixture was analysed by LC-MS at various time points by treating aliquots with DTT at 60 °C for three minutes and subsequently diluting to around 0.03 mg/mL. The reaction mixture was purified by various means (all according to manufacturer's protocol with adjustment for low volumes of sample, e.g. using only one half of the column resin), including dialysis using slide-a-lyser (100 µL volumne, 10 kDa MWCO), protein A spin columns, dye removal spin columns, PD spintrap G25 spin columns. Purified reaction products were analysed by LC-MS (reducing conditions as described previously, Figure S31): Light chain: calculated: 23443; found 23440. Heavy chain carrying two rhodamine molecules calculated: 53108; found: 53101 = 4 molecules per Ab. SDS-PAGE, Bradford assay, BCA assay, nanodrop analysis, UV absorbance and fluorescence emission (Figure S32). Results indicated successful attachment of dye to the antibody, specifically to the heavy chain. The loading was quantified as being between ~4 molecules of fluorophore per antibody. A sample of the construct (4.5 µg) was treated with PNGase F (0.25 µL) and incubated at 37 °C for 24 hours. After this time analysis by SDS-PAGE gel indicated >90% removal of the glycans. Fluorescence analysis indicated that fluorophore had been removed from the antibody construct.

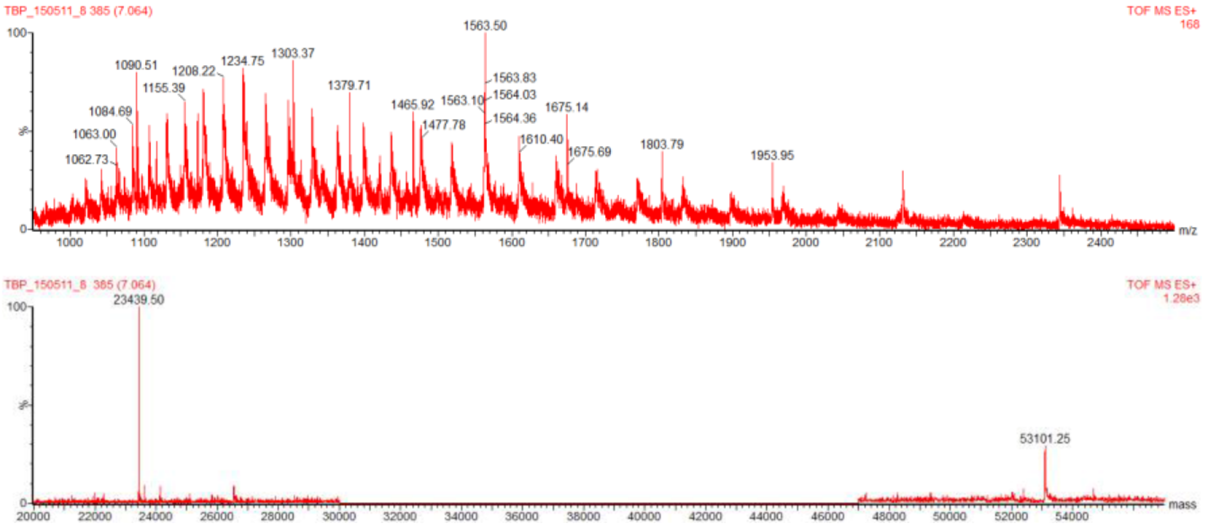

Figure S31. rMS Analysis of the loading of rhodamine cargo using **8a** on **7b.** Light chain: calculated: 23443; found 23440. Heavy chain carrying two rhodamine molecules calculated: 53108; found: 53101 = 4 molecules per Ab.

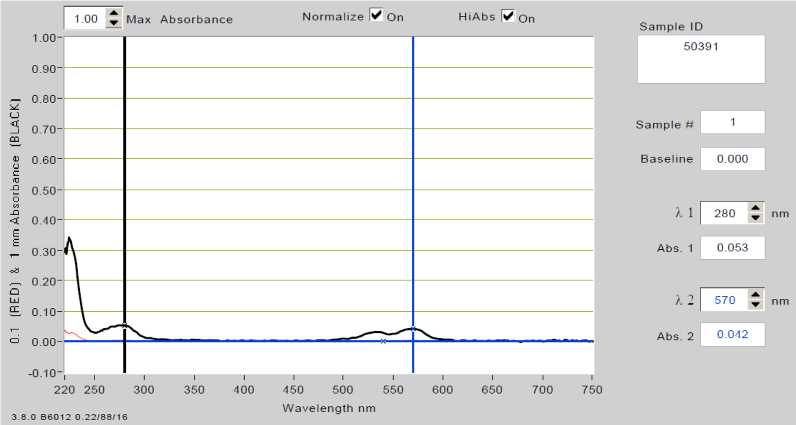

Figure S32. Fluorescence Emission Spectrum of the Loading of rhodamine cargo using **8a** on **7b**

Flow Cytometry

Tissue culture media was removed from a T75 flask and cells were washed with PBS (without calcium or magnesium, pH 7.4) before addition of PBS (2 mL supplemented with 1 mM EDTA). The flasks were incubated at 37°C until cells were fully detached (5-7 minutes). For Trypsin treatment the cells from a separate flask were washed with PBS and then incubated at 37 °C in the presence of Trypsin (1 mL supplemented with 1 mM EDTA) until cells were fully detached (3-4 minutes). Cells treated with PBS/EDTA were resuspended in PBS supplemented with 0.1 mM $CaCl_2$ and 0.5 mM $MgCl_2$ (8 mL, pH 7.4) and the cells treated with trypsin were resuspended in complete media (9 mL) to quench the trypsin. Cells were washed with ice-cold PBS twice (5 minutes, 500 x g, 4 °C), counted and $5\cdot10^5$ cells were resuspended in 100 µL for staining. Cells were stained with the rhodamine-conjugated Herceptin using 0.25, 1 and 5 µg of protein (concentrations based on nanodrop measurement and subsequent dilution) and incubated for 30 minutes in the dark at 4 °C. The cells were washed 3 times (5 minutes, 500 x g, 4°C) with ice-cold PBS (1 mL) before fixing them with formaldehyde (300 µL of a 1% solution in PBS, pH 7.4). The cells were incubated at 4 °C for 1 hour in the dark before analsis. The cells were analysed on a BD FACSCalibur and the data was processed using FlowJo version 10.0.

3.8.1 Loading of Cemadotin

Synthesis of Her bearing cemadotin from azido-Her ***7b***

Herceptin derivative **7b** (10 µg in ammonium bicarbonate, 50 mM, 2.15 mg/mL solution) was treated with cemadotin derivative **8b** (varying equivalents, added as a 10 mM solution in 1:1 MeCN:H_2O) at either RT or 37 °C. Aliquots were taken at time intervals, snap-frozen and stored at -20 °C until analysis. Samples were analysed by SDS-PAGE under reducing conditions. The gel was over-run by around 15 minutes to maximize separation of the heavy chain components on the gel. Analysis suggested that reaction was rapid and essentially

quantitative. As a control, when only partially glycosylated antibody was used (around 90% glycosylated) it was possible to distinguish a difference in migration ('gel shift') between deglycosylated starting material, glycosylated starting material and drug-conjugated antibody (Figure S33). Consistent with the azide tag being found in the glycan and reaction with that azide, the deglycosylated 'spike' did not alter its migration and so does not react non-specifically. Alternatively, reaction mixtures were analyzed directly by LC-MS (Figure S34): Light chain: calculated: 23443; found 23440. Heavy chain carrying two cemadotin molecules calculated: 53538; found: 53531. Reaction mixtures were purified by Protein G chromatography (spin columns), the fractions containing product combined, dialysed thrice against ammonium bicarbonate (50 mM aqueous solution, 10 kDa MWCO slide-a-lyser) and concerntrated to >0.5 mg/mL using vivaspin 500 centrifugal concentrators. CD analysis showed no gross perturbation to structure (Figure S35). To allow variation of drug-loading, hydrophobic interaction chromatography using a MabPAC-10 HIC column was employed (Figure S36).

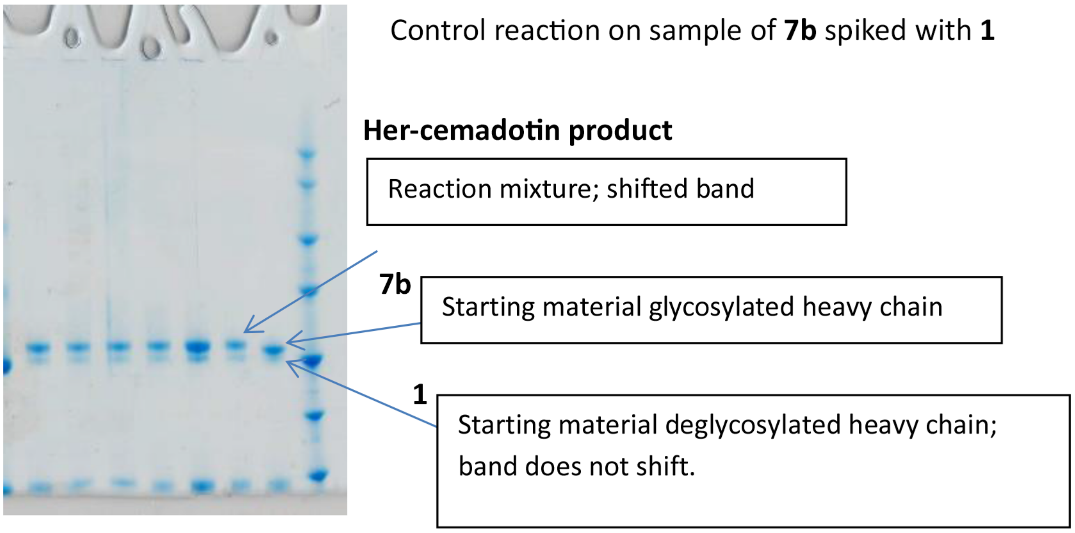

Figure S33. Polyacrylamide Gel Electrophoretic Analysis of the Reaction of **7b** with cemadotin reagent **8b.**

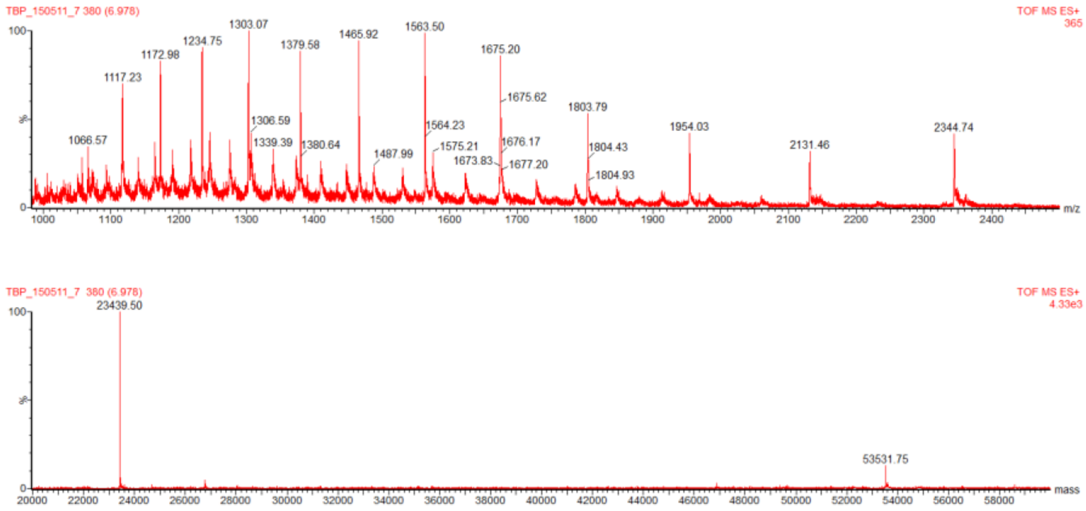

Figure S34. rMS Analysis of the loading of cemadotin cargo using **8b** on **7b.** Light chain: calculated: 23443; found 23440. Heavy chain carrying two cemadotin molecules calculated: 53538; found: 53531.

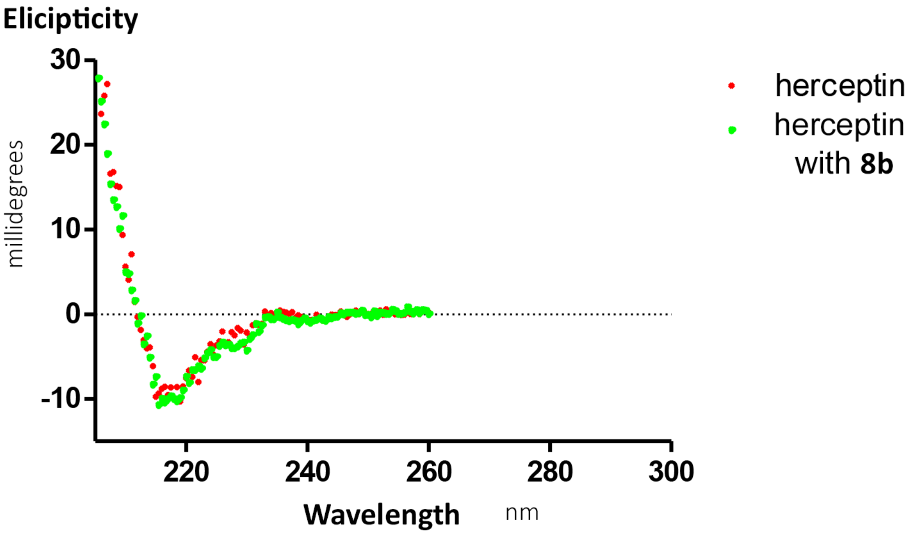

Figure S35. Circular Dichroism Analysis of the Loading of Cemadotin Cargo.

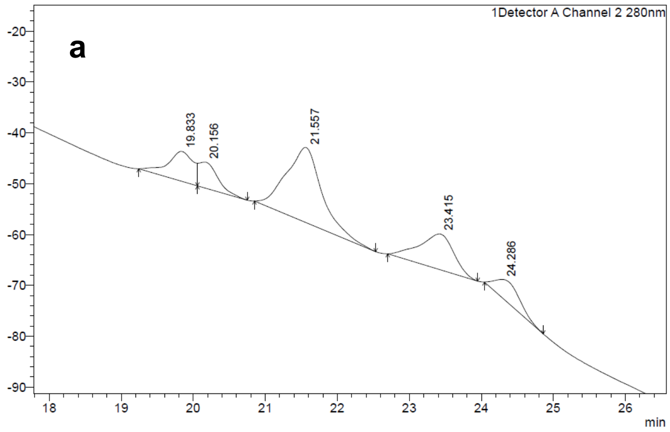

b

Peak#	Ret. Time	Area	Height	Conc.
1	19.844	2508382	57540	23.820
2	21.557	4687293	139523	44.511
3	22.654	320958	44396	3.048
4	23.430	1917140	61354	18.205
5	24.293	1096940	40354	10.417
Total		10530712	343167	

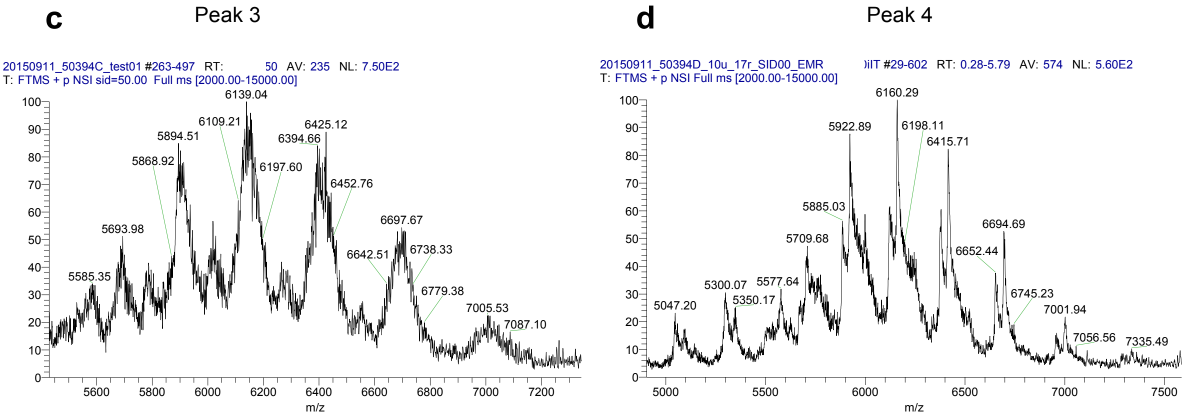

Figure S36. Hydrophobic interaction chromatography (panel **a**) using a MabPAC-10 HIC column was employed to vary the loading of cemadotin cargo (panel **b**). Panels (**c**) and (**d**) show illustrative nMS analysis of corresponding fractions 3 and 4 from (a,b), respectively.

Cytotoxcity

To analyse the effect of attachment of drug to Herceptin, we analyzed the cytotoxicity of the ADCs SK-BR-3 cells that overexpress the HER2 antigen. Cells were trypsinised and counted as described above. An aliquot of the cells was removed and the concentration adjusted to around 100 000 cells/mL by addition of extra media. The cells were plated into a 96 well plate to give around 20 000 cells/well. The plate was incubated overnight to allow the cells to adhere. Herceptin was added to the cells to give a final concentration in the range 100 µg/mL to 1.25 µg/mL. Negative control wells were included in the analysis. The cells were incubated for a further 3 days before treatment with PrestoBlue reagent. Cell viability was quantified by fluorescence emission (excitation λ 544 nm, emission λ 590 nm). SK-BR-3 cells exhibited lower viability when treated with Herceptin and even lower viability when treated with cemadotin conjugate. There was no effect from treatment with hIgG (Sigma). EC_{50} values were estimated using a non-linear, variable slope log[inhibitor] v. response equation (Prism).

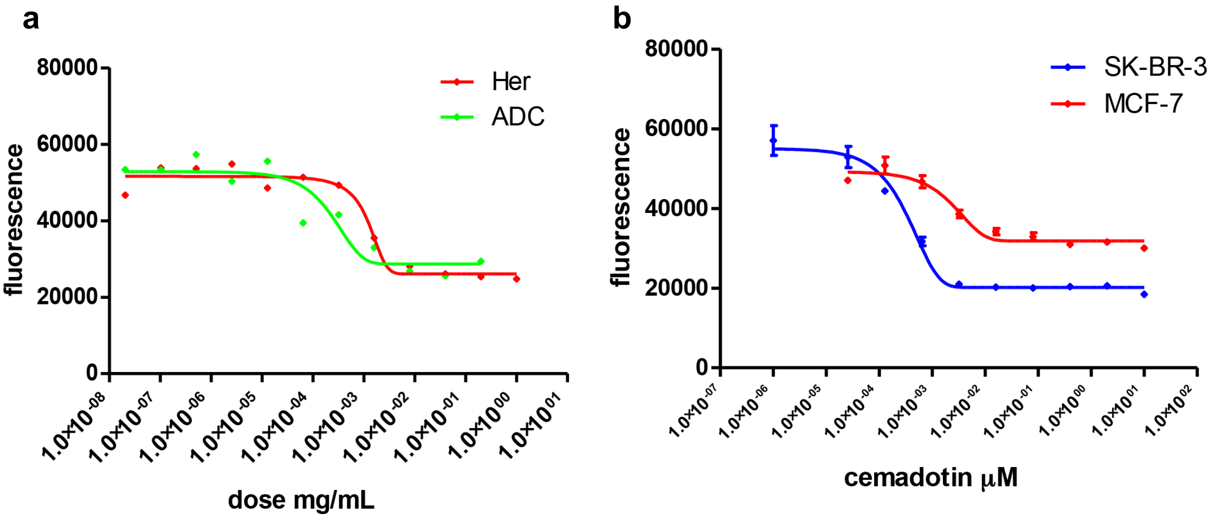

Figure S37. Cell viability dose-response comparison plots of (**a**) commercial Herceptin™ (red) with 'ADC' formed from loading of **7b** with cemadotin **8b** (green) against Her(+) SK-BR-3 cells; (**b**) ADC' formed from loading of **7b** with cemadotin **8b** against Her(+) SK-BR-3 (blue) and Her(-) MCF-7 (red) cells.

4.1 Elucidation of MS technique

Samples of commercial Herceptin were analysed by MS under various conditions. As described above, LC-MS under reducing conditions did not provide full detail of the complexity of the antibody glycoforms. We presume this is due to loss of fine detail caused by the low signal-to-noise ratio which results from the broad charge state envelope. LC-MS samples were run on an LCT Premier instrument.

We investigated the level of detail which could be obtained using a Q-Exactive Orbitrap instrument modified for the transmission of high mass ions.[8] Under denaturing conditions and using direct infusion nono-electrospray to introduce the samples, a very broad charge state envelope was again obtained (figure S38A). The envelope appeared to comprise two maxima and deconvolution gave the expected mass of intact Herceptin. The spectrum indicated the presence of multiple glycoforms but individual peaks were broadened and not particularly well resolved. Incomplete desalting of the sample also appeared to lead to significant asymmetry of the peaks.

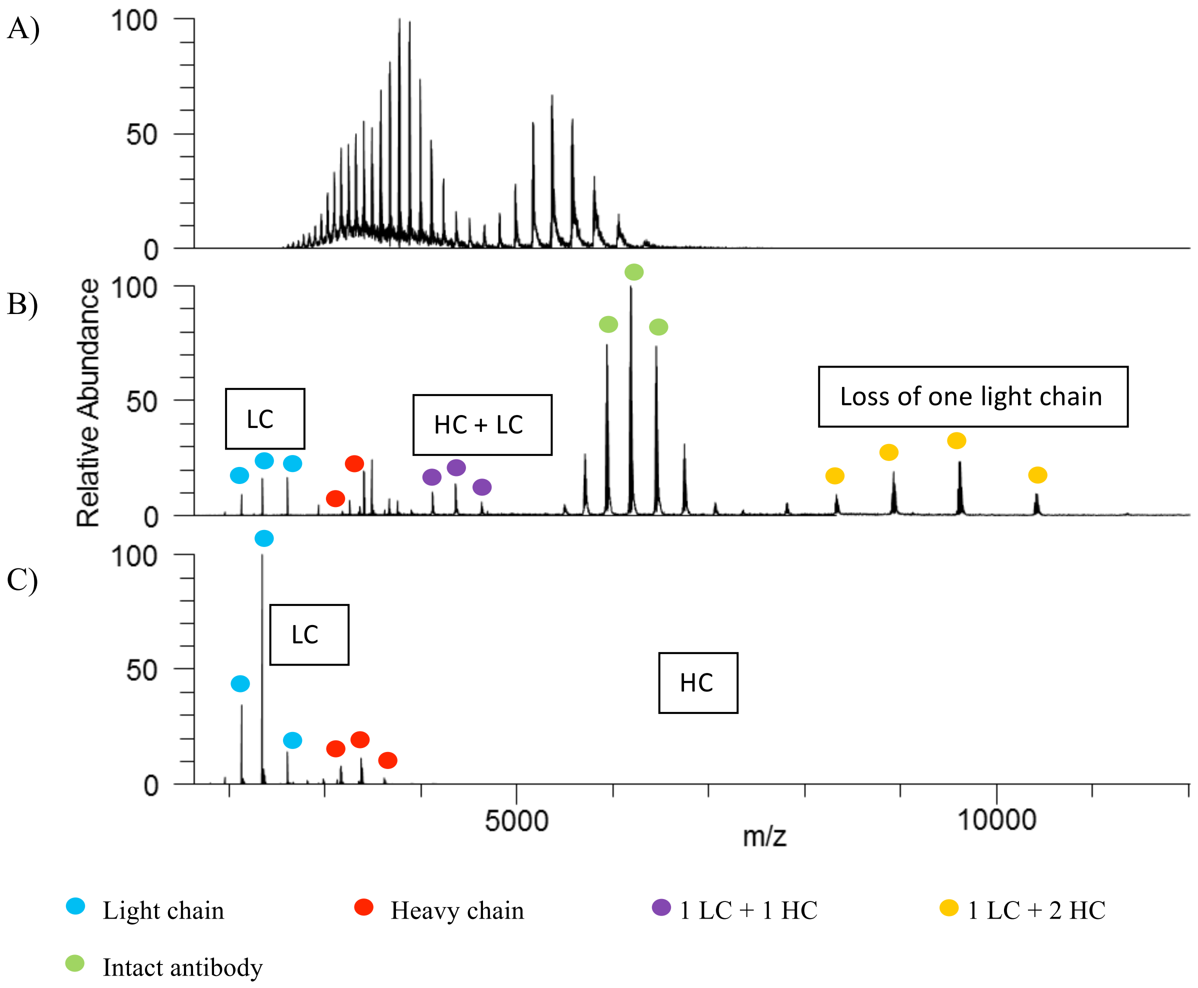

Figure S38. Commercial Herceptin was analysed using a Q-Exactive Orbitrap instrument under A) denaturing, B) reduced and C) reduced and denaturing conditions.

Analysis of reduced Herceptin under non-denaturing conditions gave rise to interesting spectra which appeared to show a small amount of fragmentation of the antibody into a mixture of heterotrimeric, hetero- and homo-dimeric and monomeric light and heavy chain proteins (figure S38B). The level of dissociation could be altered by varying the potential (collision energy) applied to the HCD cell. This indicates that the presence of the disulfide bonds is not required to hold the antibody structure together, but that they do significantly stabilise the heterotetrameric structure.[8a] Notably the application of higher levels of collisional activation to non-reduced antibody samples did not lead to any fragmentation (see also discussion below).

Analysis under reducing *and* denaturing conditions gave complete dissociation of the light and heavy chains, as expected, resulting in two separate ion envelopes (figure S38C).

We chose to analyse the glycosylation reactions using native conditions in order to maximise the detail we could obtain of the product distribution in the reaction mixtures. Similar benefits of high resolution native-MS have recently been reported for analysis of the ADC trastuzumab emtansine.[9] Having established that native conditions enabled the most detailed analysis of intact antibody samples, we briefly investigated the effect of varying desolvation energy on the spectral output (figure S39). The desolvation energy applied in the HCD cell must be carefully tuned in order to maximise spectral quality without dissociation of labile glycan moieties. Desolvation energies up to and including 125 V gave incomplete desolvation, leading to broad, poorly resolved peaks with low signal to noise. At voltages above this (i.e. 150-200 V) separate peaks corresponding to individual glycoforms could be resolved. Notably the higher desolvation energy did not lead to any visible fragmentation of the antibody, *via* backbone cleavage, disulfide cleavage or loss of glycans (the relative abundance of the various glycoforms remained consistent at 150-200V), indicating that these higher voltages were not detrimental to the stability of the samples. Therefore all spectra were collected at 150 V and voltages up to 200V were used when additional desolvation was required.

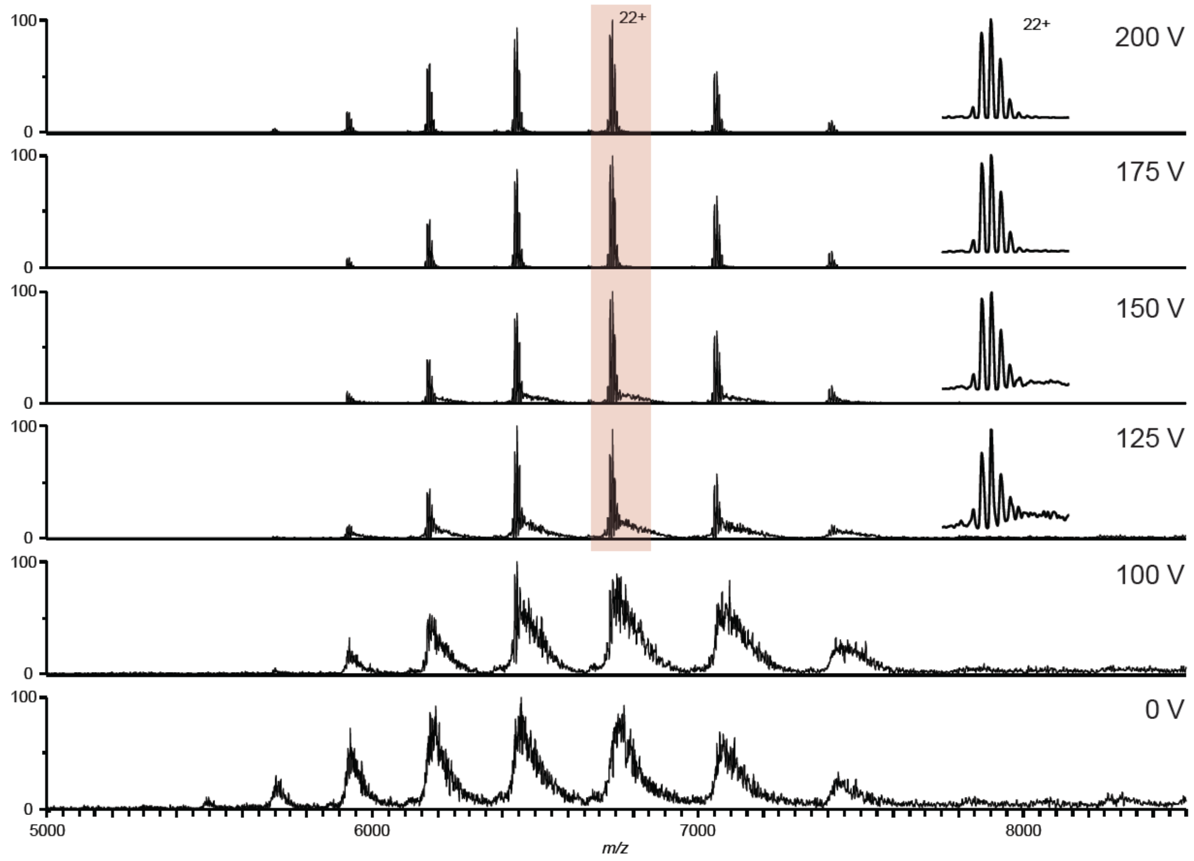

Figure S39. Commercial Herceptin was analysed under native conditions using different desolvation voltages.

4.2 Tryptic mapping of Herceptin

Commercial Herceptin was subjected to tryptic digestion and subsequent LC-MS/MS analysis. Results were processed using PEAKS 7.5. Data was searched against a concatenated database of Herceptin sequences and the common Repository of Adventitious Proteins, cRAP (downloaded 25/8/2015). The enzyme was Trypsin [D] [P] with up to 3 missed cleavages allowed. Carbamidomethylation of cysteine was added as a fixed modification and oxidation (M), deamination (N,Q) and Asn glycosylation (G0, G0F, G1, G1F, G2 or G2F) as variable modifications. Mass tolerances were 0.6 Da for the precursor and 0.2 Da for fragment ions. Results were filtered for peptides with an FDR of >1% and proteins with -10lgp>20 and at least one unique peptide.

Coverage of the light chain was 96% and heavy chain 94% (figure S40). The heavy chain appears to be entirely lysine clipped. Multiple modifications were observed for both the heavy and light chains, the most prevalent being deamination. Confident modifications (>5% ion intensity) are annotated above the amino acid sequence. Two glycan identifications, one on the HC [E296-K320] and one on the LC [V146-K169] were removed after manual validation.

There are some variations in the reported heavy chain sequence of Herceptin in the literature[14] and in the drug databank (http://www.drugbank.ca/drugs/DB00072). Our mapping analysis, together with intact and reduced MS data confirmed that the sequence matches well with that previously reported.[14]

The observation of variable levels of methionine oxidation and deamidation offers an explanation for the slight variability in precision of deconvoluted masses obtained when antibody samples were measured under native MS conditions. These modifications may vary depending on age of sample at time of analysis, and the precise conditions they have been exposed to. A full analysis of the effects of various treatments on these minor components is beyond the scope of the current work.

Further digestion using GluC in combination with trypsin, along with Titanium-enrichment experiments enabled the identification of putative sites of chemical glycation. A low level of glycation was observed on the peptide which bears the *N*-glycosylation site (figure S41A). This has presumably arisen due to nucleophilic attack of one of the alcohol groups of the GlcNAc(Fuc) core which remains after EndoS trimming. Two heavy chain residues, S17 and K30, were found to have undergone glycation (figure S41 B and C); both of these sites are in the variable heavy chain domain, and make up part of the Fab. In the light chain the peptide containing the K188, H189, K190 motif was also found to have been glycated (figure S41D), but in this case it was not possible to determine unequivocally which of these three sites carries the glycan. Indeed, it may be that any one of these three residues may undergo glycation.

Various other possible sites of glycation were found, but were discarded as the intensity of the ions arising from these peptides were of very low intensity. It is clear from this analysis that glycation can occur in a number of positions on the antibody backbone.

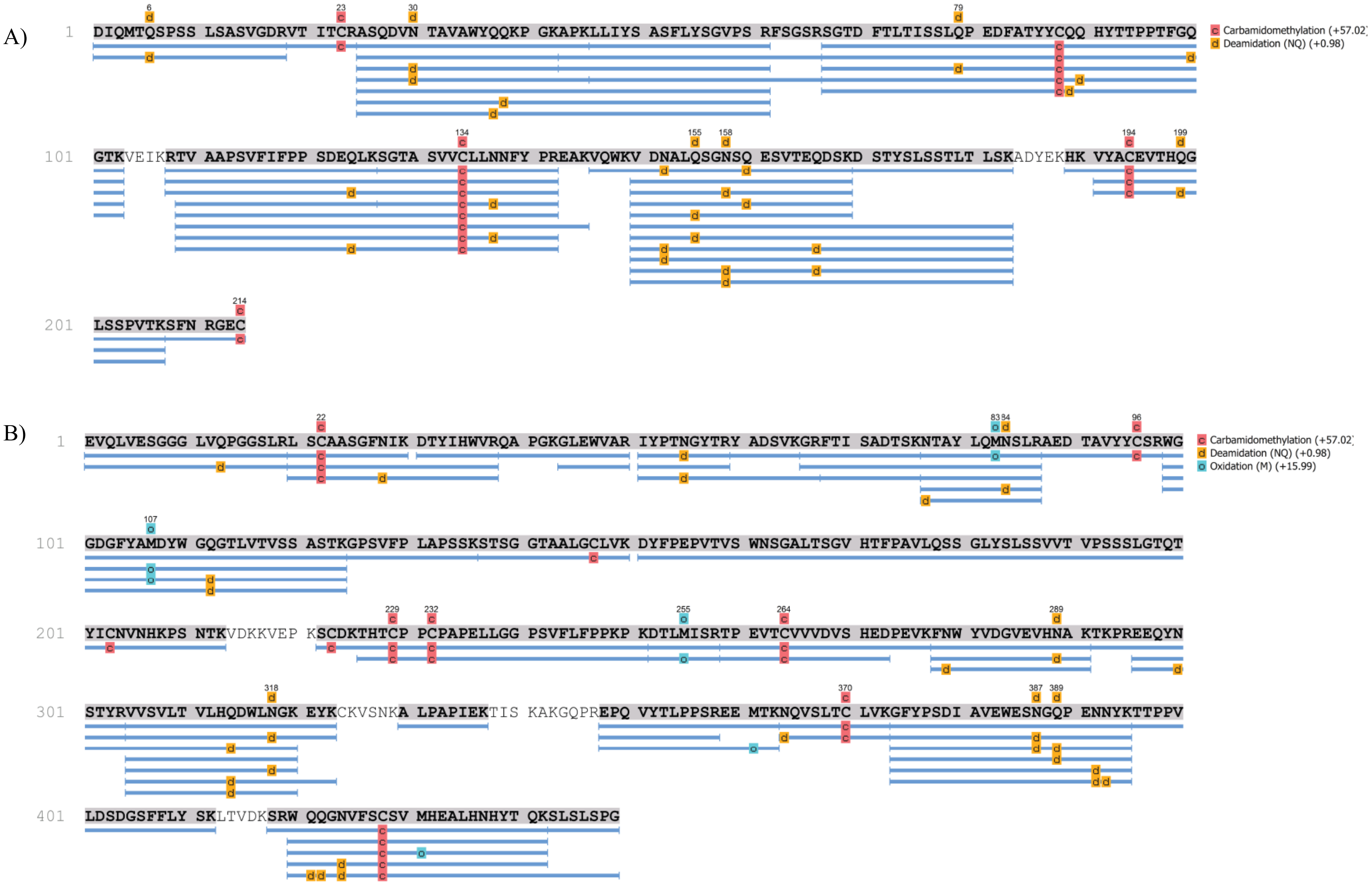

Figure S40. Commercial Herceptin was subjected to digestion by trypsin and subsequent MS/MS analysis.

A)

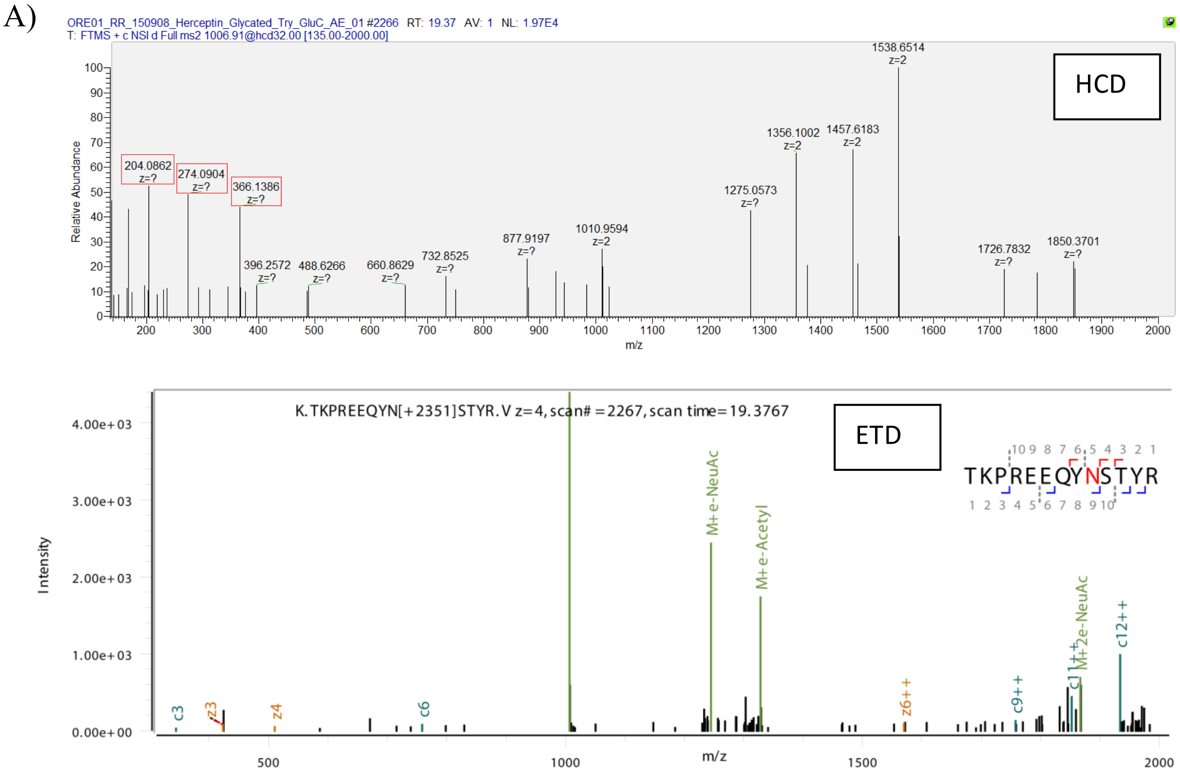
ORE01_RR_150908_Herceptin_Glycated_Try_GluC_AE_01 #2266 RT: 19.37 AV: 1 NL: 1.97E4
T: FTMS + c NSI d Full ms2 1006.91@hcd32.00 [135.00-2000.00]
HCD
Relative Abundance
m/z
204.0862 z=?
274.0904 z=?
366.1386 z=?
396.2572 z=?
488.6266 z=?
660.8629 z=?
732.8525 z=?
877.9197 z=?
1010.9594 z=2
1275.0573 z=?
1356.1002 z=2
1457.6183 z=2
1538.6514 z=2
1726.7832 z=?
1850.3701 z=?
K.TKPREEQYN[+2351]STYR.V z=4, scan# =2267, scan time=19.3767
ETD
Intensity
TKPREEQYNSTYR
M+e-NeuAc
M+e-Acetyl
M+2e-NeuAc
c3
z3
z4
c6
z6++
c9++
c11++
c12++

B)

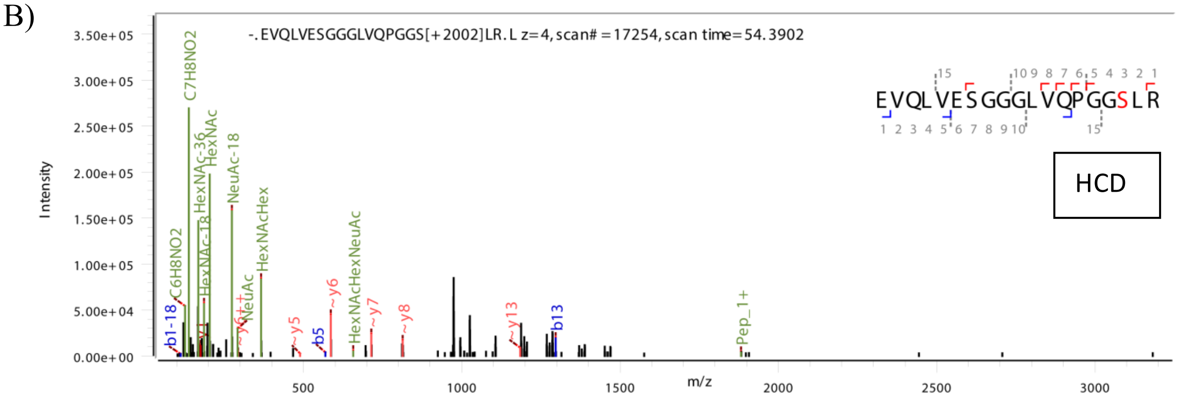
-.EVQLVESGGGLVQPGGS[+2002]LR.L z=4, scan# =17254, scan time=54.3902
HCD
Intensity
m/z
EVQLVESGGGLVQPGGSLR
C7H8NO2
C6H8NO2
HexNAc-36
HexNAc-18
HexNAc
NeuAc-18
NeuAc
HexNAcHex
HexNAcHexNeuAc
b1-18
b5
b13
~y5
~y6
~y7
~y8
~y13
Pep_1+

C)

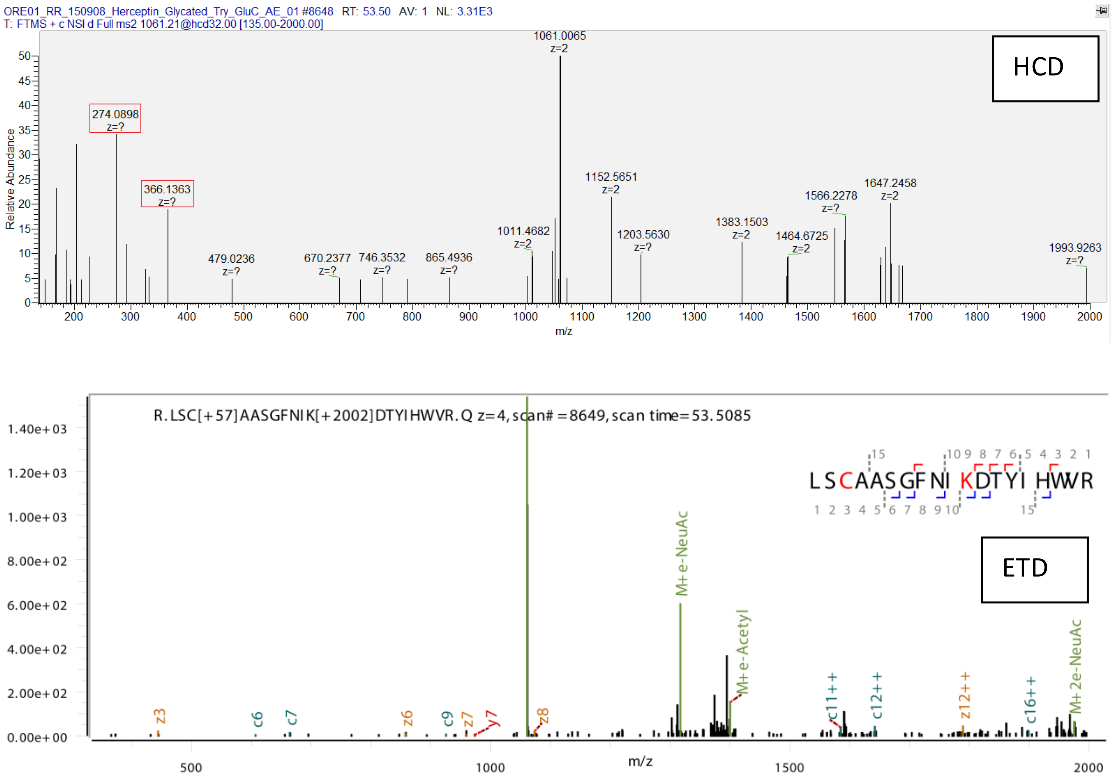
ORE01_RR_150908_Herceptin_Glycated_Try_GluC_AE_01 #8648 RT: 53.50 AV: 1 NL: 3.31E3
T: FTMS + c NSI d Full ms2 1061.21@hcd32.00 [135.00-2000.00]
HCD
Relative Abundance
m/z
1061.0065 z=2
274.0898 z=?
366.1363 z=?
1152.5651 z=2
1647.2458 z=2
1566.2278 z=?
1203.5630 z=?
1383.1503 z=2
1464.6725 z=2
1011.4682 z=2
1993.9263 z=?
479.0236 z=?
670.2377 z=?
746.3532 z=?
865.4936 z=?
R.LSC[+57]AASGFNIK[+2002]DTYIHWVR.Q z=4, scan# =8649, scan time=53.5085
LSCAASGFNIKDTYIHWR
ETD
Intensity
M+e-NeuAc
M+e-Acetyl
M+2e-NeuAc
z3
c6
c7
z6
c9
z7
y7
z8
c11++
c12++
z12++
c16++

D)

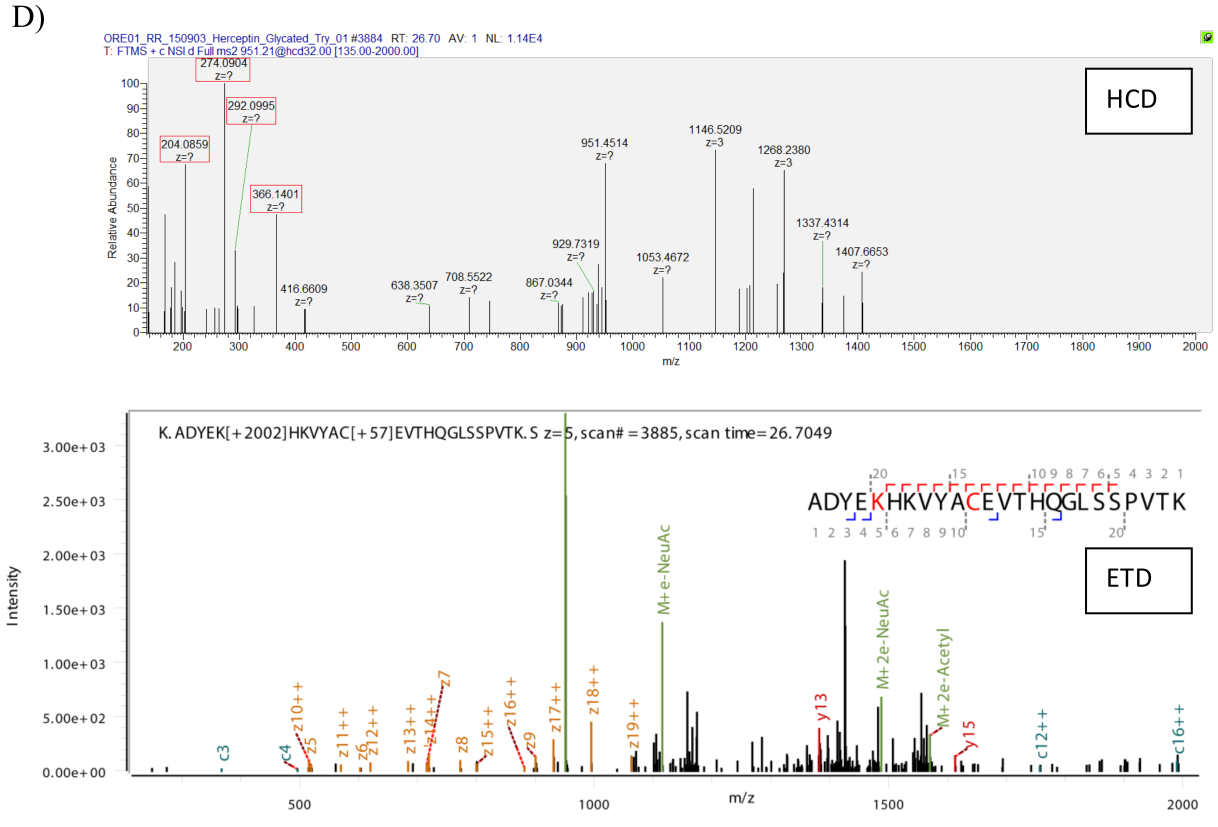
ORE01_RR_150903_Herceptin_Glycated_Try_01 #3884 RT: 26.70 AV: 1 NL: 1.14E4
T: FTMS + c NSI d Full ms2 951.21@hcd32.00 [135.00-2000.00]
HCD
Relative Abundance
m/z
274.0904 z=?
292.0995 z=?
204.0859 z=?
366.1401 z=?
1146.5209 z=3
951.4514 z=?
1268.2380 z=3
1337.4314 z=?
1407.6653 z=?
929.7319 z=?
1053.4672 z=?
416.6609 z=?
638.3507 z=?
708.5522 z=?
867.0344 z=?
K.ADYEK[+2002]HKVYAC[+57]EVTHQGLSSPVTK.S z=5, scan# =3885, scan time=26.7049
ADYEKHKVYACEVTHQGLSSPVTK
ETD
Intensity
M+e-NeuAc
M+2e-NeuAc
M+2e-Acetyl
c3
c4
z5
z10++
z11++
z6
z12++
z13++
z14++
z7
z8
z15++
z16++
z9
z17++
z18++
z19++
y13
y15
c12++
c16++

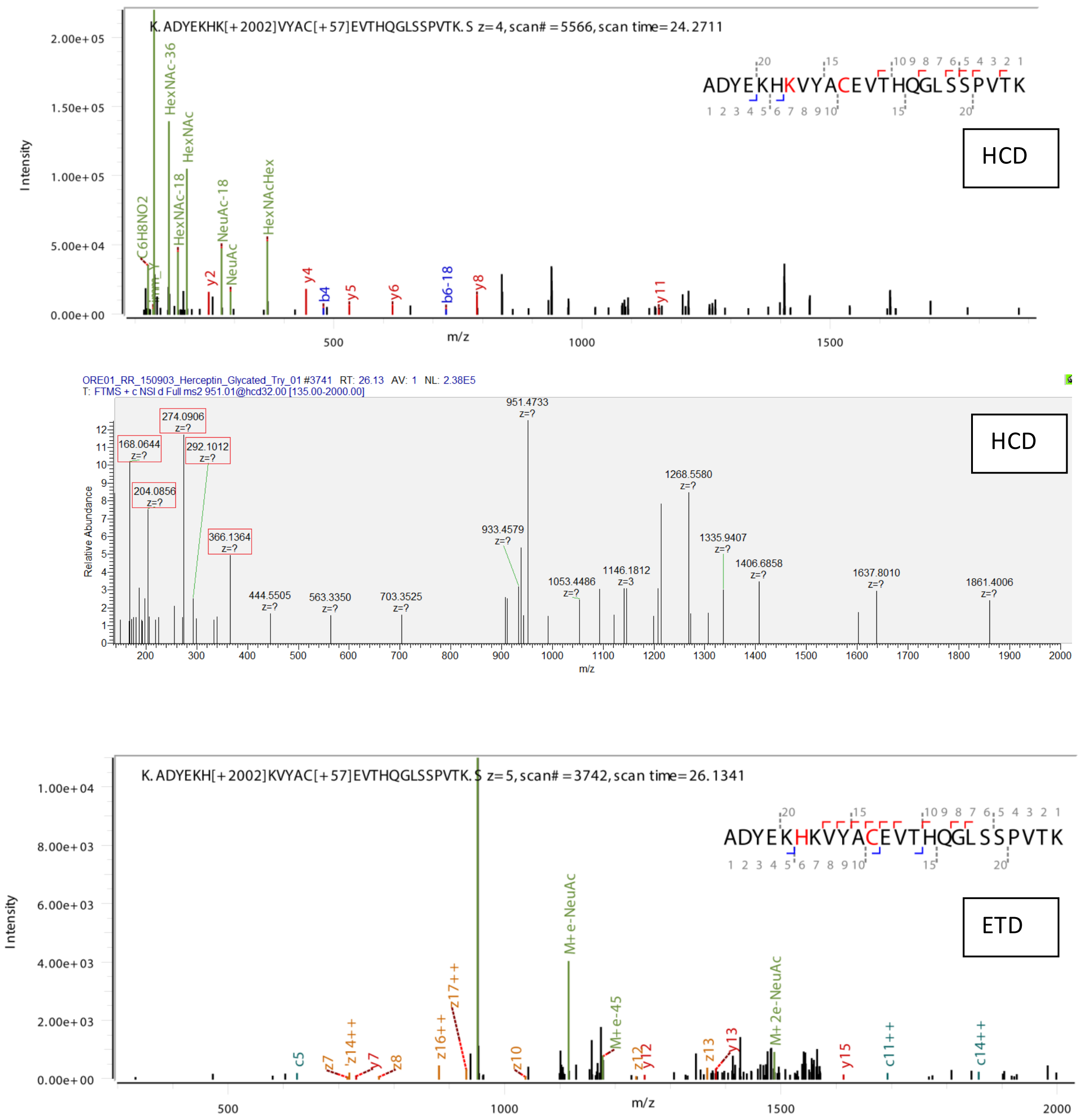

Figure S41. Location of glycation sites was determined by proteolytic digestion (trypsin or trypsin with Glu C) followed by LC-MS/MS analysis. LC-MS/MS ETD and HCD spectra indicate glycation at: A) heavy chain N-300; B) heavy chain S17 ('~' in the spectra annotation indicates neutral loss of glycan); C) heavy chain K30; D) light chain K188, H189 and/or K190. Signature peaks for glycan identification (denoted by red boxes in the HCD spectra): HexNAc (*m/z* 204.09), fragments of HexNAc (*m/z* 138.06 and 168.07), HexHexNAc (*m/z* 336.14), NeuAc (*m/z* 292.09) and NeuAc-H_2O (*m/z* 274.09).

4.3 LC-MS/MS analysis

10 µg each of wild type and modified (glycated) herceptin samples were dissolved in 50 µL of denaturing buffer (8 M Urea, 50 mM Ammonium Bicarbonate). The disulfide bonds were reduced (1 µL of 200 mM DTT solution in denaturing buffer) at 56 °C for 25 minutes, followed by alkylation (2 µL of 200 mM iodoacetamide solution in denaturing buffer) at room temperature for 30 minutes in the dark. Alkylation was quenched by further addition of 1 µL of DTT solution. The samples were diluted 4X with 50 mM Ammonium Bicarbonate solution. Trypsin was added to the sample in ratio of 1:50 Enzyme: Protein (w/w). The samples were incubated at 37 °C for 16 hours. Half of each digested sample was then transferred to a fresh eppendorf tube and Glu-C was added in the ratio 1:25 Enzyme: Protein (w/w). Digestion with Glu-C was performed at 37 °C for 4 hours. Following digestion, samples were stored at – 20 °C until further manipulation.

Peptides were separated on using EASY-nLC 1000 UHPLC system (Proxeon) and sprayed directly into an Orbitrap Elite mass spectrometer (Thermo Fisher Scientific) through an EASY-Spray nano-electrospray ion source (Thermo Fisher Scientific). The peptides were trapped on a guard column (75 µm i.d. x 20 mm, reprosil C18, 3µm, 120 Å, packed in-house) using solvent A (0.1% Formic Acid in water) at a pressure of 500 bar. The peptides were separated on an EASY-spray Acclaim PepMap® analytical column (75 µm i.d. × 500 mm, RSLC C18, 2 µm, 100 Å) using a linear gradient (length: 58 minutes, 8 % to 30 % solvent B (0.1% formic acid in acetonitrile), flow rate: 200 nL/min). The raw data was acquired on the mass spectrometer in a data-dependent mode using a HCD/ETD based method.[15] Full scan MS spectra (scan range 350-2000 m/z, resolution 30000, AGC target 1e6, maximum injection time 250 ms) and sub-sequent HCD MS/MS spectra (resolution 15000, AGC target 3e4, maximum injection time 500 ms) of the five most intense peaks were acquired in the Orbitrap. HCD fragmentation was performed at 32% of normalized collision energy and the signal intensity threshold was maintained at 500 counts. If peaks at any of *m/z* 138.0546, 168.0563 or 204.0867 were detected among top 20 most intense peak of a HCD spectrum, an ETD MS/MS spectrum of the precursor ion was triggered. ETD spectra (AGC cation target 5e3, AGC anion target 2e5, cation maximum injection time 100 ms, normalized collision energy 35%) were acquired in the Iontrap.

Samples were also analyzed on a Q Exactive mass spectrometer (Thermo Fischer Scientific) attached to a nLC-MS/MS system configured as described above. The peptides were separated using the same linear gradient. Full scan MS spectra were acquired in the Orbitrap (scan range 350-2000 m/z, resolution 70000, AGC target 3e6, maximum injection time 100 ms). After the MS scans, the 10 most intense peaks were selected for HCD fragmentation at 30% of normalised collision energy. HCD spectra were also acquired in the Orbitrap (resolution 17500, AGC target 5e4, maximum injection time 120 ms) with the first mass fixed at 100 m/z.

Digested peptides were further enriched for sialic acid containing peptides using Titanium dioxide as described previously.[16] The enrichment experiment was carried out using Titanium dioxide columns (TopTip Glygen, cat TT1TIO.96). Briefly, the titanium dioxide

columns were centrifuged at 2000 rpm for 30 seconds to compact loose column material at the bottom of the tip. The tips were then washed with 50 μL of elution buffer (25% aqueous NH_4OH) by centrifugation at 1000 rpm for 2 minutes. The tips were equilibrated three times with 65 μL of loading buffer (80% Acetonitrile, 5% TFA, 1 M glycolic acid) by centrifugation at 1500 rpm for 1 minute. Equal amount of double concentration loading buffer was added to 60 μL of digested samples (from aforementioned digestion experiment, around 3 μg of digested protein) to give the same final concentration as that of loading buffer. Samples were loaded onto the column in two batches and the column then centrifuged at 500 rpm for five minutes. The column was washed twice with 65 μL of wash buffer 1 (80% Acetonitrile, 1% TFA) by centrifugation at 1000 rpm for 2 minutes followed by washing with wash buffer 2 (20% Acetonitrile, 0.1% TFA) under the same conditions. The columns were then centrifuged at 2000 rpm for 2 minutes to remove residual buffer. The enriched peptides were eluted into fresh eppendorf tubes containing 20 μL of 5 % formic acid using 20 μL of elution buffer and centrifugation at 500 rpm for 5 minutes. The centrifugation speed was increased to 2000 rpm for 2 minutes to collect the residual buffer from the tip, if any. The enriched peptide samples were analyzed on an Orbitrap Elite mass spectrometer as described before.

The raw data was processed using Byonic™ software (Protein Metrics, version 2.2.9). The raw MS file was searched against the sequence of Herceptin using a decoy based search. Trypsin (and GluC in the case of the double digested samples) with a maximum number of 4 missed cleavages was selected as the protease. Carbamidomethylation (Cysteine), protein *N*-terminus acetylation and oxidation (Methionine) were set as variable modifications. The glycans HexNAc(1)Fuc(1) and HexNAc(4)Hex(5)Fuc(1)NeuAc(2) were sought as variable modifications at *N*-glycosylation sites (Aspargine) whereas the glycan HexNAc(3)Hex(5)NeuAc(2) was sought as a variable modification at *N*-glycosylation sites (Aspargine), *O*-glycosylation sites (Serine, Threonine) and at possible glycation sites (Cysteine, Histidine, Lysine, Arginine and Glutamine). Raw data from the Orbitrap Elite was analysed using the precursor mass tolerance set to 20 ppm with fragmentation type both HCD and ETD with mass tolerances set to 50 ppm and 0.8 Da respectively. Raw data from the Q Exactive instrument was analysed using the precursor mass tolerance set to 20 ppm with fragmentation type QTOF/HCD with mass tolerance 50 ppm. The protein false discovery rate (FDR) was set at 0.01. The spectra which identified glycated peptides were manually validated. Any glycated peptides which were also observed in the commercial Herceptin were discarded from the hit set.

5. Extended results, discussion and analyses

The following discussion was removed from the main text to comply with editorial requests for a shortened manuscript but should be read in the context of the foregoing discussions in the main text in order to provide proper background, context and analysis.

5.1 Extended Introduction

Protein glycosylation is the most common and varied post-translational modification, and critically influences numerous protein attributes and biological processes.[17] Glycoproteins are typically produced as heterogeneous mixtures of glycoforms, wherein a single protein chain is linked to various different glycan structures.[18] Control over the glycosylation pattern of recombinantly-produced glycoproteins is both a major goal and a considerable challenge for the scientific community. Current approaches[19] to access homogeneous glycoproteins include engineering cellular glycosylation pathways, total chemical (semi-)synthesis or *in vitro* glycoprotein remodelling, but no single general method yet exists which is appropriate or feasible for all glycoprotein targets. Amongst these strategies Endoglycosidase (ENGase or 'Endo')-catalyzed glycosylation using activated sugars as donors is an attractive technology that can enable direct synthetic access to glycoproteins and glycopeptides.[20] The initially limited scope of protein substrates for this methodology was recently expanded to include the biopharmaceutically-important class of antibody (Ab) substrates by using the enzyme EndoS,[21] a family 18 glycoside hydrolase capable of glycosylating immunoglobulins (Igs).[22]

Monoclonal Abs (mAbs) are a rapidly-growing class of therapeutics,[23] the highest-selling class of biologics[24] (US sales $24bn in 2012) and represented half of the top ten best selling drugs in 2014.[25] Their controllable antigen (and so target) specificity has led to direct use and also as carriers for potent drug molecules[26] (as well as dyes and radiolabels[27]) for both diagnostic and therapeutic applications. Over 30 Abs and antibody-drug conjugates (ADCs) are approved for clinical use, with many more under development.[23, 28] The critical functional roles of *N*-linked glycans in Abs have seen the understanding and control of glycosylation become a major focus of associated Ab (and biological) science.[29] They alter stability, modulate rate of clearance and hence pharmacokinetic profile.[30] They also critically affect aggregation and folding and hence immunogenicity.[31] Proper Ab glycosylation is essential for complement activation;[32] core fucosylation and bisecting GlcNAc alter binding to Fc receptors and hence influence Ab-dependent cell-mediated cytotoxicity[33] whilst the degree of galactosylation and sialylation affects Ab-mediated inflammation.[34] Thus, these sugars provide a potential 'switch-like' system in Abs for functional modulation and yet precise control of these glycosylation patterns is not yet possible. Ab are currently produced industrially as mixtures of glycoforms and even these display batch-to-batch inconsistency.[30b, 32a, 35]

Antibodies are all *N*-glycosylated (typically at Asn-297) in the Fc region of each heavy chain, so each Ab typically bears at least two *N*-glycans. Around 20% of Igs also possess *N*-glycosylation sites in the Fab domain. All therapeutic Abs are currently produced in mammalian (CHO) cell lines. These give rise to mixtures including variously galactosylated, core-fucosylated, biantennary glycoforms: the so-called G0F/G0F, G0F/G1F and G1F/G1F tend to predominate, however, > 20 different glycoforms have been identified.[36] Not only does this creation of mixtures of Abs bearing one subset of glycans

therefore represent a problem of purity and precision (see above) but also a missed opportunity to determine Ab function through synthesis (by exploiting glycan switching).

The ENGase method therefore represents a potentially unique approach for accessing pure glycoforms in a manner not possible using current culture methods. However, until now it has been generally assumed that this method will necessarily, cleanly give rise to homogeneous glycoforms by virtue of the direct reversal of selective enzymatic hydrolytic activity. Here we demonstrate that this is incorrect and not only do non-specific background chemical modifications compete in such reactions but we now reveal optimized methods that allow access to essentially homogenous (≥90% pure) glycoforms of a therapeutically important mAb.

We chose therapeutic mAb Herceptin™ as a highly representative Ab substrate; in combination with radio- or chemotherapy it is a leading treatment for early stage HER2(+) breast cancer, recurrent/metastatic breast or stomach cancer and has been used to treat late stage oesophageal cancer.[37]

5.2 Extended Conclusions

The increased control of the *natural* glycosylation pattern of Abs enabled by our method may allow new optimally efficacious Ab drugs as near-homogeneous glycoforms., e.g., by increasing receptor binding whilst decreasing inflammatory response. Here, a complex biantennary glycan has been attached to give a highly pure sialylated Ab glycoform. Fc glycan sialylation imparts anti-inflammatory properties to IgGs,[34a] and has led to the use of IVIg as an anti-inflammatory drug. However, only ~ 10% of total IgG content of IVIg carries sialylation, and therefore repeated high doses of IVIg are used for treatment of autoimmune disease.[38] Production of pure sialylated Abs (>90% sialylated as achieved here) may be a means to greatly improve the anti-inflammatory properties of IVIg and reduce dosage. Interestingly, circulating Abs in humans tend to carry between 1 and 5 glucosyl residues.[39] Aberrant protein glycation has also been linked to rheumatoid arthritis.[40] Our observations may therefore also have a more general bearing on the inherent reactivity and modification of Abs with sugars in human biology.

The incorporation of *unnatural* glycans into Abs via optimized EndoS-catalyzed glycosylation now also allows access to Abs with both a defined glycosylation pattern and reaction handles at specific positions. Future work will focus on their utility in the attachment of cargo. Current approaches to ADCs use backbone Lys and Cys residues as attachment sites and so give rise to heterogeneity, with concomitant issues in pharmacokinetics and reproducibility. Recently, other complementary approaches to ADCs have been suggested that make use of Fc *N*-glycosylation. Some require either complete removal[41] or significant truncation[22b, 42] of the glycans, which may adversely affect Ab stability and immunogenicity[30-31] as well as impacting on FcγR binding.[43] Alternatively, remodelling at the non-reducing termini[44], whilst partially reducing glycan heterogeneity, does not remove the variation arising from bisecting sugars[45] and/or hybrid or triantennary glycans.[46] The approach reported herein now enables attachment of a defined number of reaction handles at specified positions, whilst removing virtually all glycan heterogeneity. We note too that there may be distinct advantages to the *in vivo* attachment of cargoes.[47] Therefore, such access to ‘pure & tagged’ glycoproteins (using e.g. **6a-e**) is also anticipated to be of general utility beyond Abs for use in other glycoprotein therapeutics, diagnostics and as probes of organismal biology.

6.1 References

[1] C. D. Heidecke, T. B. Parsons, A. J. Fairbanks, *Carbohydr. Res.* **2009**, *344*, 2433-2438.

[2] M. Noguchi, T. Fujieda, W. C. Huang, M. Ishihara, A. Kobayashi, S.-i. Shoda, *Helvetica Chimica Acta* **2012**, *95*, 1928-1936.

[3] M. A. Azagarsamy, V. Yesilyurt, S. Thayumanavan, *Journal of the American Chemical Society* **2010**, *132*, 4550-4551.

[4] G. T. Zugates, D. G. Anderson, S. R. Little, I. E. B. Lawhorn, R. Langer, *Journal of the American Chemical Society* **2006**, *128*, 12726-12734.

[5] aM. Koketsu, A. Seko, L. R. Juneja, M. Kim, N. Kashimura, T. Yamamoto, *Journal of carbohydrate chemistry* **1995**, *14*, 833-841; bA. Seko, M. Koketsu, M. Nishizono, Y. Enoki, H. R. Ibrahim, L. R. Juneja, M. Kim, T. Yamamoto, *Biochim. Biophys. Acta* **1997**, *1335*, 23-32.

[6] Y. Kajihara, Y. Suzuki, N. Yamamoto, K. Sasaki, T. Sakakibara, L. R. Juneja, *Chem. Eur. J.* **2004**, *10*, 971-985.

[7] W. Huang, Q. Yang, M. Umekawa, K. Yamamoto, L.-X. Wang, *ChemBioChem* **2010**, *11*, 1350-1355.

[8] aA. Dyachenko, G. Wang, M. Belov, A. Makarov, R. N. de Jong, E. T. J. van den Bremer, P. W. H. I. Parren, A. J. R. Heck, *Analytical Chemistry* **2015**, *87*, 6095-6102; bJ. A. C. D. Joseph Gault, Idlir Liko, Jonathan T.S. Hopper, Kallol Gupta, Nicholas G. Housden, Weston Struwe, Michael T. Marty, Todd Mize, Cherine Bechara, Ya Zhu, Beili Wu, Colin Kleanthous, Eugen Damoc, Alexander Makarov & Carol V. Robinson, *Submitted to Nature Methods* **2015**.

[9] J. Marcoux, T. Champion, O. Colas, E. Wagner-Rousset, N. Corvaia, A. Van Dorsselaer, A. Beck, S. Cianferani, *Protein science : a publication of the Protein Society* **2015**, *24*, 1210-1223.

[10] aS. J. Williams, B. L. Mark, D. J. Vocadlo, M. N. G. James, S. G. Withers, *Journal of Biological Chemistry* **2002**, *277*, 40055-40065; bY. K. Sykulev, R. S. Nezlin, *Immunology Letters* **1982**, *5*, 121-126; cN. Çetinbaş, M. S. Macauley, K. A. Stubbs, R. Drapala, D. J. Vocadlo, *Biochemistry* **2006**, *45*, 3835-3844.

[11] S.-Q. Fan, W. Huang, L.-X. Wang, *J. Bio. Chem.* **2012**, *287*, 11272-11281.

[12] R. B. Trimble, A. L. Tarentino, *The Journal of biological chemistry* **1991**, *266*, 1646-1651.

[13] aP. S. a. D. Pressman, *J. Biol. Chem.* **1962**, *237*, 3679-3685; bT. A. Seldon, K. E. Hughes, D. J. Munster, D. Y. Chin, M. L. Jones, *Journal of Biomolecular Techniques : JBT* **2011**, *22*, 50-52.

[14] D. Nebija, H. Kopelent-Frank, E. Urban, C. R. Noe, B. Lachmann, *Journal of Pharmaceutical and Biomedical Analysis* **2011**, *56*, 684-691.

[15] aS. D. Julian Saba, Eric Hemenway, and Rosa Viner, *International Journal of Proteomics* **2012**, *2012*, 7; bC. Singh, C. G. Zampronio, A. J. Creese, H. J. Cooper, *Journal of Proteome Research* **2012**, *11*, 4517-4525.

[16] G. Palmisano, S. E. Lendal, K. Engholm-Keller, R. Leth-Larsen, B. L. Parker, M. R. Larsen, *Nat. Protocols* **2010**, *5*, 1974-1982.

[17] aA. Helenius, Aebi, Markus, *Science* **2001**, *291*, 2364-2369; bA. Varki, *Glycobiology* **1993**, *3*, 97-130.

[18] R. G. Spiro, *Glycobiology* **2002**, *12*, 43R-56R.

[19] D. P. Gamblin, E. M. Scanlan, B. G. Davis, *Chem. Rev.* **2009**, *109*, 131-163.

[20] aL.-X. Wang, J. V. Lomino, *ACS Chemical Biology* **2012**, *7*, 110-122; bJ. R. Rich, S. G. Withers, *Nat Chem Biol* **2009**, *5*, 206-215; cA. J. Fairbanks, *Comptes Rendus Chimie* **2011**, *14*, 44-58.

[21] M. Collin, A. Olsen, *EMBO J* **2001**, *20*, 3046-3055.

[22] aJ. J. Goodfellow, K. Baruah, K. Yamamoto, C. Bonomelli, B. Krishna, D. J. Harvey, M. Crispin, C. N. Scanlan, B. G. Davis, *J. Am. Chem. Soc.* **2012**, *134*, 8030-8033; bW. Huang, J. Giddens, S.-Q. Fan, C. Toonstra, L.-X. Wang, *J. Am. Chem. Soc.* **2012**, *134*, 12308-12318.

[23] J. M. Reichert, *MAbs* **2012**, *4*, 413-415.

[24] S. Aggarwal, *Nat Biotech* **2014**, *32*, 32-39.

[25] http://qz.com/349929/best-selling-drugs-in-the-world/

[26] R. V. J. Chari, M. L. Miller, W. C. Widdison, *Angew Chem Int Ed* **2014**, *53*, 3796-3827.
[27] J. Barbet, M. Bardies, M. Bourgeois, J. F. Chatal, M. Cherel, F. Davodeau, A. Faivre-Chauvet, J. F. Gestin, F. Kraeber-Bodere, *Methods Mol Biol* **2012**, *907*, 681-697.
[28] H. L. Perez, P. M. Cardarelli, S. Deshpande, S. Gangwar, G. M. Schroeder, G. D. Vite, R. M. Borzilleri, *Drug Discov Today* **2014**, *19*, 869-881.
[29] aM. Dalziel, M. Crispin, C. N. Scanlan, N. Zitzmann, R. A. Dwek, *Science* **2014**, *343*, 1235681; bR. M. Anthony, F. Wermeling, J. V. Ravetch, *Ann. N.Y. Acad. Sci.* **2012**, *1253*, 170-180.
[30] aH. Li, M. d'Anjou, *Curr. Opin. Biotechnol.* **2009**, *20*, 678-684; bL. Liu, *J. Pharm. Sci.* **2015**, *104*, 1866-1884; cT. A. Millward, M. Heitzmann, K. Bill, U. Längle, P. Schumacher, K. Forrer, *Biologicals* **2008**, *36*, 41-47.
[31] M. M. C. van Beers, M. Bardor, *Biotechnol. J.* **2012**, *7*, 1473-1484.
[32] aR. Jefferis, *Biotechnol Prog* **2005**, *21*, 11-16; bR. Jefferis, *Trends in Pharmacological Sciences* **2009**, *30*, 356-362.
[33] R. Jefferis, *Arch. Biochem. Biophys.* **2012**, *526*, 159-166.
[34] aY. Kaneko, F. Nimmerjahn, J. V. Ravetch, *Science* **2006**, *313*, 670-673; bF. Nimmerjahn, R. M. Anthony, J. V. Ravetch, *Proc Natl Acad Sci USA* **2007**, *104*, 8433-8437.
[35] aT. P. Patel, R. B. Parekh, B. J. Moellering, C. P. Prior, *Biochem. J.* **1992**, *285*, 839-845; bJ. Muthing, S. E. Kemminer, H. S. Conradt, D. Sagi, M. Nimtz, U. Karst, J. Peter-Katalinic, *Biotechnol Bioeng* **2003**, *83*, 321-334; cA. E. Schmelzer, W. M. Miller, *Biotechnol Prog* **2002**, *18*, 346-353; dJ. P. Kunkel, D. C. Jan, J. C. Jamieson, M. Butler, *J Biotechnol* **1998**, *62*, 55-71; eP. H. van Berkel, J. Gerritsen, G. Perdok, J. Valbjorn, T. Vink, J. G. van de Winkel, P. W. Parren, *Biotechnol Prog* **2009**, *25*, 244-251.
[36] S. Rosati, E. T. J. van den Bremer, J. Schuurman, P. W. H. I. Parren, J. P. Kamerling, A. J. R. Heck, *mAbs* **2013**, *5*, 917-924.
[37] aM. X. Sliwkowski, J. A. Lofgren, G. D. Lewis, T. E. Hotaling, B. M. Fendly, J. A. Fox, *Semin Oncol* **1999**, *26*, 60-70; bC. A. Hudis, *New Engl. J. Med.* **2007**, *357*, 39-51.
[38] aN. Ishii, T. Hashimoto, D. Zillikens, R. J. Ludwig, *Clin Rev Allergy Immunol* **2010**, *38*, 186-195; bS. Siberil, S. Elluru, S. Graff-Dubois, V. S. Negi, S. Delignat, L. Mouthon, S. Lacroix-Desmazes, M. D. Kazatchkine, J. Bayry, S. V. Kaveri, *Ann N Y Acad Sci* **2007**, *1110*, 497-506.
[39] A. Lapolla, D. Fedele, M. Garbeglio, L. Martano, R. Tonani, R. Seraglia, D. Favretto, M. A. Fedrigo, P. Traldi, *J Am Soc Mass Spectrom* **2000**, *11*, 153-159.
[40] aS. Ligier, P. R. Fortin, M. M. Newkirk, *Br J Rheumatol* **1998**, *37*, 1307-1314; bM. Takahashi, M. Suzuki, K. Kushida, S. Miyamoto, T. Inoue, *Br J Rheumatol* **1997**, *36*, 637-642.
[41] P. Dennler, A. Chiotellis, E. Fischer, D. Brégeon, C. Belmant, L. Gauthier, F. Lhospice, F. Romagne, R. Schibli, *Bioconj. Chem.* **2014**, *25*, 569-578.
[42] R. van Geel, M. A. Wijdeven, R. Heesbeen, J. M. M. Verkade, A. A. Wasiel, S. S. van Berkel, F. L. van Delft, *Bioconj. Chem.* **2015**.
[43] Y. Mimura, P. Sondermann, R. Ghirlando, J. Lund, S. P. Young, M. Goodall, R. Jefferis, *J. Biol. Chem.* **2001**, *276*, 45539-45547.
[44] aZ. Zhu, B. Ramakrishnan, J. Li, Y. Wang, Y. Feng, P. Prabakaran, S. Colantonio, M. A. Dyba, P. K. Qasba, D. S. Dimitrov, *mAbs* **2014**, *6*, 1190-1200; bB. M. Zeglis, C. B. Davis, D. Abdel-Atti, S. D. Carlin, A. Chen, R. Aggeler, B. J. Agnew, J. S. Lewis, *Bioconj. Chem.* **2014**, *25*, 2123-2128.
[45] R. Jefferis, *Nat Rev Drug Discov* **2009**, *8*, 226-234.
[46] J. Sjögren, E. F. J. Cosgrave, M. Allhorn, M. Nordgren, S. Björk, F. Olsson, S. Fredriksson, M. Collin, *Glycobiology* **2015**, *25*, 1053-1063.
[47] R. Rossin, P. Renart Verkerk, S. M. van den Bosch, R. C. M. Vulders, I. Verel, J. Lub, M. S. Robillard, *Angew Chem Int Ed* **2010**, *49*, 3375-3378.
